# Effect of a national mental health campaign on population mental resilience in the Netherlands: a retrospective longitudinal cohort analysis using a dynamical systems perspective

**DOI:** 10.1016/j.lanepe.2025.101434

**Published:** 2025-09-04

**Authors:** Junus M. van der Wal, Frank Pijpers, Gabriela Lunansky, Anja Lok, Mary Nicolaou, Anastasia Korezelidou, Karien Stronks, Claudi L. Bockting

**Affiliations:** a Centre for Urban Mental Health, University of Amsterdam, Amsterdam, the Netherlands; b Department of Psychiatry, Amsterdam UMC, Location AMC, Meibergdreef 5, 1105 AZ, Amsterdam, the Netherlands; c Department of Public and Occupational Health, Amsterdam UMC, University of Amsterdam, Van der Boechorststraat 7, 1081 BT, Amsterdam, the Netherlands; d Statistics Netherlands (CBS), Henri Faasdreef 312, The Hague, the Netherlands; e Korteweg-de Vries Institute for Mathematics, University of Amsterdam, Amsterdam, the Netherlands; f Amsterdam Public Health Institute, Amsterdam University Medical Center, the Netherlands; g Department of Epidemiology & Data Science, Amsterdam UMC, Location VU, De Boelelaan 1117, 1081 HV, Amsterdam, the Netherlands; h Department of General Practice and Health Services Research, University of Heidelberg, Im Neuenheimer Feld 130.3, D - 69120, Heidelberg, Germany

**Keywords:** Resilience, Dynamical systems, Network analysis, Depression, MDD, Public campaign

## Abstract

**Background:**

In 2016, the Dutch government initiated a nationwide campaign, aiming to increase mental resilience and reduce the prevalence of major depressive disorder (MDD) through boosting knowledge and awareness, improving attitudes towards talking about MDD, and encouraging people to seek/provide help. Utilizing a dynamical systems perspective, we aimed to examine its impact on mental resilience and subsequently on self-reported mental health and healthcare use, in the Dutch population.

**Methods:**

We used data from the Longitudinal Internet Studies for the Social Sciences (LISS) panel, an online panel representative of the Dutch adult population drawn from nationwide population registries, only excluding non-private households or households where no adult understood the Dutch language (2012–2022, n = 12,420). Yearly 5-item Mental Health Inventory (MHI-5) was used to estimate psychological networks from which population-level resilience proxies were derived (i.e., node threshold, connectivity, stability difference). We tested for change in resilience (i.e., stability difference) between 2012 versus 2019 and 2012 versus 2022 using 95% bias-corrected and accelerated (BCa) bootstrap intervals and descriptively examined changes in other resilience proxies. Interrupted time-series analysis was used to test for trend changes in MHI-5 scores, mental healthcare use, and medication use for depression and/or anxiety after (2016–2019) versus before (2012–2015) start of the campaign. Separate analyses were performed based on gender, social support, and urbanicity for mental resilience, as well as education and migration background for additional outcomes.

**Findings:**

A total of 12,420 participants were included between 5-11-2012 and 31-12-2022, of which 5807 participants were lost to attrition while 5873 participants entered through yearly refreshment samples. Mental resilience showed no improvement after initiation of the campaign (2012 versus 2019: difference −0.153, BCa-interval −0.359 to 0.0152, p = 0.07; 2012 versus 2022: difference −0.0631, BCa-interval −0.262 to 0.153, p = 0.57). There was a trend increase in self-reported mental health problems (2016–2019 versus 2012–2015, beta: 0.11, SE: 0.04, p-value = 0.002), a decrease in medication use (beta: −0.31, SE 0.12, p = 0.01), as well as differential trend changes between 2016 and 2019 versus 2012–2015 in mental healthcare use (decreasing trend change in medium versus low social support, beta: −0.31; SE: 0.15; p-value = 0.04; increasing trend change in non-Dutch with a LMIC background versus Dutch origin, beta: 0.67, SE: 0.25, p = 0.008).

**Interpretation:**

While the naturalistic setting refrains us from attributing results solely to the campaign, we also found no indication that a mental health campaign is able to combat the high burden of MDD through strengthening population-level mental resilience. There is a need for a stronger evidence base for effective and equitable population-level mental health interventions.

**Funding:**

Centre for Urban Mental Health, a research priority area of the 10.13039/501100001827University of Amsterdam.

**Research in context**

**Evidence before this study**

Searches on PubMed and Google Scholar were performed between 1-9-2023 and 31-1-2024, without language restrictions and including publications from inception up to the date of search, for longitudinal studies that examined the impact of mental health campaigns on population-level mental resilience and/or the prevalence or incidence of major depressive disorder (MDD). We used search terms such as “mental health campaign”, “anti-stigma campaign”, “public campaign”, or “awareness campaign”, combined with terms like “resilience” and “mental resilience” as well as “major depressive disorder”, “common mental disorders”, and “depression”. In addition, we added search terms to identify studies that also utilized a dynamical systems perspective on mental resilience, such as “dynamical system”, “complex system”, “dynamical network”, or “resilience landscape”. We found no longitudinal studies that directly measured the impact of a mental health campaign on population-level mental resilience, nor on incidence or prevalence of MDD. However, we did find several studies that evaluated the impact of mental health campaigns on other outcomes, particularly attitude towards mental illness and other stigma-related outcomes. These studies found mixed results, with some studies reporting changes in attitudes toward mental disorders, while other studies found positive impact on the likelihood to talk about mental illness with others or seek information about mental health disorders. Hence, there is a dearth of knowledge on how these campaigns impact mental resilience of the population, as well as how they contribute to combatting the burden of MDD.

**Added value of this study**

While the naturalistic setting of this study refrains us from attributing results solely to the campaign, we found no indication that a public campaign which aimed to reduce prevalence of MDD through strengthening resilience, was able to positively impact mental resilience at the population-level, also not in subgroups most vulnerable for MDD (i.e., females, those experiencing low social support, those residing in urban areas). This is the first study to utilize a dynamical systems perspective to examine the impact of a public mental health campaign on mental resilience. Doing so is in line with the increasingly popular notion that resilience constitutes a dynamical process of adaptation to life's circumstances, rather than a stable personality trait. Specifically, we examined changes in mental resilience in the Dutch population—before and after initiation of a nationwide mental health campaign—through different proxies derived from psychological networks, particularly resilience landscapes, which signify the tendency of these networks to be in either a healthy or unhealthy state (and switch between these states).

**Implications of all the available evidence**

There is an urgent need for future research on how macro-level interventions could positively impact mental resilience, especially in more vulnerable subgroups, in order to develop sustainable and equitable interventions that foster mental resilience and combat the high burden of MDD.

## Introduction

The burden of major depressive disorder (MDD) has not decreased over the past decades, despite high healthcare spending and the introduction of new treatments. More focus on prevention may help to curb this high burden.^1^ In this light, the Dutch Ministry of Health, Welfare and Sport launched a nationwide campaign in 2016 (‘*Dealing with depression*’) that aimed to combat the high MDD prevalence by strengthening mental resilience and fostering social support through (I) boosting knowledge and awareness, (II) improving attitudes towards talking to others about MDD, and (III) encouraging people to provide or seek (professional) help.^2^ The campaign is part of a depression prevention program, comprising a covenant between stakeholders (including health authorities, healthcare providers, schools and employers, and associations for people with lived experience) to commit to a reduction of MDD prevalence in the Netherlands of 30% in 2030, compared to 2017. Another important component of this program, alongside raising awareness, is the optimization of mental health service to specific high-risk groups and/or settings.^2^ In 2018, the campaign was renamed ‘*Hey, it's okay*’ and broadened its scope to also include anxiety disorders (from 2019 onward) and all other mental health disorders (from 2021 onward).^3^^,^^4^ This ongoing campaign, primarily targeted at the adult population (>18 years), is routinely promoted through ads on television, radio, in the cinema, billboards, and online. Key components of the campaign include showcasing the experiences of someone suffering from MDD, providing information on signs and symptoms of MDD and other mental disorders as well as their prevalence in the Dutch population, and encouraging those suffering from mental health problems to talk about their complaints as well as bystanders to starts a conversion about mental health (Supplementary Figure S1).^2^, ^3^, ^4^

Previous evaluations of this campaign found its reach in the general public lagged behind during the initial years (57–62%) compared to other government campaigns, yet increased in the following years (66–74%). Moreover, these evaluations found effects on intention to talk about—or seeking/providing help for—depression, but not attitude towards depression (i.e., whether people deemed it normal to talk about depression or seek/provide help), which is partly in line with studies in other countries.^3^, ^4^, ^5^ In Canada, awareness of the online campaign *In One Voice* was associated with increased likelihood to talk about—or seek information relating to—mental health issues, but not with changes in attitude.^6^ Similarly, a suicide awareness campaign in the Netherlands was associated with increased openness to seek help for mental health problems, yet no positive impact on stigma towards depression or suicide was found.^7^ In contrast, the campaigns *Time to Change* in England and *Optimizing suicide prevention programs and their implementation* (OSPI) in Germany, Hungary, Ireland, and Portugal, were linked to increasing positive attitudes towards mental illness and a decrease in MDD-related stigma, respectively.^8^^,^^9^ Whether these campaigns were associated with decreased burdens of MDD is unclear. Moreover, there is a growing body of research, including a recent review in Lancet Psychiatry, which advocates to broaden the evaluation of mental health interventions to include *mental resilience*, as examined from a dynamical systems perspective. As further detailed in Textbox 1, this is in line with the increasing understanding that resilience should not be regarded as a stable personality trait, but instead as the continues process of adaptation to external and internal stressors to preserve a healthy mental state.^10^^,^^14^^,^^20^^,^^23^ While positively impacting mental resilience may help to safeguard mental health in the population, it was not included as outcome in previous evaluations of mental health campaigns.

Textbox 1 **Mental resilience from a dynamical systems perspective.**

There is a growing body of research that is advocating a dynamical systems perspective on mental resilience, including theoretical papers,^10^, ^11^, ^12^, ^13^ but also simulation- and empirical studies.^14^, ^15^, ^16^, ^17^, ^18^, ^19^ From a dynamical systems perspective, resilience refers to the tendency of a system (e.g., an individual or a population) to transition between different stable attractor states, for example between a healthy state (i.e., no mental disorder) and a pathological state (e.g., a depressive episode) or vice versa. As such, a highly resilient system is able to remain in a certain attractor state, despite disturbances (i.e., perturbations) that could cause a transition. This conceptualization has been analogized as a ball-in-a-cup landscape, where a cup represent an attractor state, the ball represents the state of the system, and the landscape reflects the so-called stability of the different states.^10^^,^^20^ The network approach to psychopathology has been proposed as a method to study mental health as a dynamical system. It conceptualizes psychopathology as a network of causally interacting symptoms that, through dynamical interactions, give rise to a pathological attractor state of the system.^11^ Network analysis utilizes empirical data to estimate conditional dependencies between a set of symptoms (i.e., the network nodes) which can be represented in a network structure (i.e., connections between nodes). From a network perspective, resilience reflects the tendency of a network to shift and persist in a healthy or pathological state. Various proxies derived from such networks, either relating to their architecture (e.g., network connectivity or node threshold) or their tendency to be in either a healthy or pathological state (e.g., through estimating stability landscapes), have been used as proxies for resilience in past studies.^15^^,^^18^^,^^19^^,^^21^ It has been postulated that more densely connected networks are more prone to remain in a disordered state through hysteresis, as elevated symptoms continue to activate each other.^11^ Moreover, a lower threshold for a node to be active (and thus a symptom to be present) further encourages the spread of symptoms through the network.^15^ While underpinned by some influential early studies, reporting network connectivity to be related to vulnerability to—and course of—MDD, more recent studies have found mixed results.^14^, ^15^, ^16^, ^17^^,^^19^^,^^22^ Additional information on resilience can be garnered by estimating stability landscapes from cross-sectional networks, conceptually akin to the ball-in-cup analogy.^18^ From these landscapes, the stability of all possible attractor states and thus the tendency of the system to be in a healthy or pathological state can be derived.^18^ This method has been shown to discriminate between vulnerable (high symptom scores) and resilient (low symptom scores) subgroups, based on the estimated stability landscapes (n = 2436 participants of an online panel whom completed the Coronavirus Pandemic Anxiety Scale).^18^ Thus, estimating stability landscapes holds promise as a novel and complimentary method to infer population-level resilience from networks.

This study aims to examine changes in mental resilience in the Dutch adult population following the start of the abovementioned campaign in 2016 and explore differential impact among different subgroups (according to gender, social support, and urbanicity), using the Longitudinal Internet Studies for the Social Sciences (LISS) panel.^24^ Additionally, we will examine changes in self-reported mental health and mental healthcare and medication use for depression and/or anxiety in the same period. In line with the abovementioned insights, we conceptualize mental resilience from a dynamical systems perspective (Textbox 1).^10^^,^^20^^,^^23^ Based on the proposed campaign mechanisms and previous evaluations we hypothesize that (I) start of the campaign is followed by an increase in population-level mental resilience, (II) that those experiencing more versus less social support will see an increase in mental resilience and a decrease in self-reported mental health problems and mental healthcare use, and (III) that there is a stronger effect in women versus men, given their coping strategies more often involve social support and expressing emotions.^25^

## Methods

### Data source and measures

The LISS panel is a longitudinal internet panel representative of the Dutch general population (>16 years) and is administered and managed by the non-profit research institute Centerdata (Tilburg University, the Netherlands). Recruitment started in 2007 through true probability sampling of 10,150 independent, private households drawn from nationwide populations registries by Statistics Netherlands, of which 48% agreed to participate. The nature of this sampling procedure excluded individuals living in institutions or other non-private collective households, as well as households where no adult understood the Dutch language. A comprehensive description of the sampling procedure can be found elsewhere.^24^ Throughout the years, refreshment samples (either random or stratified) were drawn to compensate for the yearly 10% household attrition and to ensure representativeness of the sample. Participants are asked to complete yearly online questionnaires on topics including health and wellbeing, social support, and socioeconomic status.^24^ Comprehensive codebooks of all questionnaires used in the LISS panel can be accessed online and more details on the recruitment procedure and response rates can be found in the Appendix. All participant provided written informed consent before inclusion in the LISS panel. A data user statement was submitted to Centerdata (Tilburg University, the Netherlands) in which we confirmed agreement with the rules and conditions for using LISS panel data, after which access was granted on 7-12-2023.^26^ We retrieved data from the years 2012–2022 from participants aged 18 years and older. The health questionnaire was not administered in 2014. Gender was assessed through self-report; participants in the years 2012–2021 were only able to select ‘male’ or ‘female’ as response; in 2022 the response ‘other’ was added. In 2022, 20 participants reported to identify neither as male or female; given their small number, there were excluded from analyses. Ethnicity was assessed through self-reported country of birth of the participant and their parents, allowing for a distinction between those with Dutch origin and those who are of first- or second generation (non-)Western origin, in line with the definition of Statistics Netherlands.^27^ An overview of all retrieved variables, including how they were measured, is provided in Supplementary Table S1. The primary outcome, which is used to estimate group-level network models from which proxies for population-level resilience are derived, is the 5-item Mental Health Inventory (MHI-5) questionnaire, a validated questionnaire for mental health in the general population containing five questions on symptoms of depression and anxiety.^28^ Items are scored on a scale from 0 to 5, with higher scores indicating a more favourable mental health state and a cut-off sum score of >60% is indicative for being mentally healthy.^29^ Missing data was not imputed, though response rates were high for all years (approximately 80–85%, see Appendix).

### Statistical analysis

#### Mental resilience

Binarized MHI-5 scores from each yearly health questionnaire were used to estimate group-level Ising model networks in the period 2012–2022. In these networks, nodes represent symptoms and connections indicate the strength of conditional dependence between symptoms. More details on how Ising model networks are estimated are provided in the Appendix. The R packages IsingFit and qgraph were used for estimation and visualization of the networks.^30^^,^^31^

Three proxies for mental resilience were derived from the networks.

I. *Node thresholds*, provided in the Ising model output, express the autonomous disposition of nodes to be ‘active’. A positive threshold indicates that the symptom tends to be present, while a negative threshold indicates the opposite (i.e., more negative thresholds suggest higher resilience).^15^
II. *Connectivity* indicates how closely associated symptoms are, which may reflect resilience as symptoms ‘spread’ more easily in densely connected networks (i.e., lower connectivity suggests higher resilience). Connectivity was computed by calculating the sum of all edge-weights in the network.^15^
III. *Stability difference* estimated from Ising model networks provide information on their tendency to be in any of the possible states (from no symptoms to all symptoms active). This is displayed in a graph where the x-axis represents all possible summed states of the network (i.e., total number of symptoms present), while the y-axis represents how much (Hamiltonian) energy it costs for the network to be in any of the possible states, expressed as the generalized potential function *U* of that state. These graphs resemble a ball-in-cup landscape in which local minima represent an attractor state; the stability of that state is reflected by how ‘deep’ the local minimum is (i.e., how much energy it would cost the system to shift to a neighbouring state). Stability difference derived from these landscapes is a metric which reflects the tendency of the network as a whole to be in a healthy (positive value) or unhealthy (negative value) state (defined as three or more active symptoms, in line with the 60% cut-off for the continuous MHI-5 scale). It is calculated by subtracting the stability of the unhealthy state from the stability of the healthy state. A higher positive value of the stability difference reflects the healthy state is relatively more stable (and vice versa). The R package Isinglandr was used to estimate stability landscapes.^18^

**Fig. 1:**
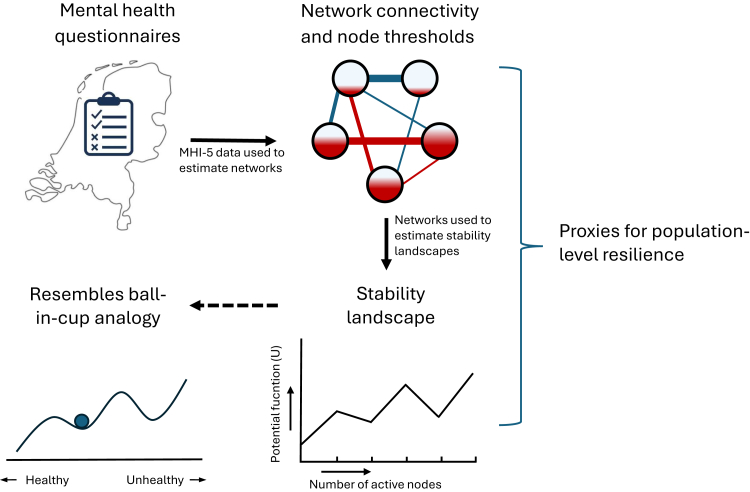
**Yearly 5-item Mental Health Inventory (MHI-5) questionnaires are used to estimate cross-section psychological networks for each respective year in which nodes resemble symptoms in the HMI-5 questionnaire that can either be present or absent and connections represent conditional dependence between the nodes. From these networks, overall connectivity and node threshold are calculated. Also, stability landscapes are estimated which plot the potential function *U* (i.e., how much Hamiltonian energy it costs for the network to have a certain number of symptoms active), which shows the stability of the attractor state(s) of the network, resembling the ball-in-cup analogy**.

Population-level resilience metrics were calculated for each year for the entire group, as well as separately according to gender, experienced social support, and urbanicity of the living environment. We tested for change in population-level resilience between the beginning (2012) versus the end of the study period, both with and without including the COVID period (i.e., 2019 and 2022), by calculating 95% bias-corrected and accelerated (BCa) bootstrap intervals for the difference in stability differences between these years. We opted to test for change over the entire study period, since it follows from dynamical systems theory that change in systems-level resilience is a slow-changing process.^10^ Changes in all resilience indicators will also be descriptively examined as proxies for population-level resilience over time. An overview of the different resilience proxies calculated in this study is provided in Fig. 1.

#### Mental health and healthcare-use

To test for changes in trends in self-reported mental health and mental healthcare use at the population level before versus after start of the campaign, we used interrupted time-series analysis.^32^ We compared trends in MHI-5 sum scores (using <60 as cut-off for mentally healthy/unhealthy, see Supplementary Table S1), mental healthcare use, and medication use for depression and/or anxiety, before (i.e., 2012–2015) and after (i.e., 2016–2019) the start of the campaign, using generalized linear mixed models. We estimated linear trend lines spanning the entire study period, after which an interaction term was added to the model to tests for differences in these trends before and after start of the campaign. The interrupted time-series models were corrected for macro-economic trends using the ‘business cycle tracer’ from Statistics Netherlands, which includes indicators like gross domestic product, consumer and producer confidence, and employment.^33^ The function glmer () from the package lme4 was used to estimate the generalized linear mixed models.^34^ A p-value of 0.05 was considered to indicate statistical significance in all abovementioned tests. A detailed description of these analyses is provided in the Appendix. All analyses were conducted in R, version 4.0.3. The full R-code of all analyses can be accessed online. This study was pre-registered on AsPredicted.

#### Role of the funding source

The funders had no role in the design and conduct of the study; collection, management, analysis, or interpretation of the data; preparation, review, or approval of the manuscript; or decision to submit the manuscript for publication.

## Results

### Baseline characteristics

A total of 12,420 participants were included between 5-11-2012 and 31-12-2022, of which 5807 participants were lost to attrition during the study period, while 5873 participants entered through yearly refreshment samples. Table 1 shows the baseline characteristics for the entire study population. Data from the health questionnaires was collected each year in the months of November and December, except for 2015 when data was collected in July and August. Note that not all participants are included for the entire span of 2012–2022, due to yearly household attrition of 10% and periodic refreshment samples to ensure representativeness.^35^ Supplementary Table S2 provides an overview of yearly sample attrition and refreshment, as well as the number of household members selected to complete the health questionnaire per year, the dates between which the data was collected, and the annual response and completion rates.

**Table 1:** Baseline characteristics entire group (2012–2022).

	Entire group (n = 12,420)
**Gender (No., %)**	
Male	5639, 45.40%
Female	6781, 54.60%
**Age**	
Median (IQR)	46 (31–61)
**Level of education (No., %)**	
Primary school	942, 7.58%
Intermediate secondary education	2487, 20.02%
Higher secondary education	1666, 13.41%
Intermediate vocational education	2896, 23.32%
Higher vocational education	2951, 23.76%
University	1445, 11.63%
*Missing*	33, 0.27%
**Experienced social support (No., %)**	
Low	2776, 22.35%
Middle	7046, 56.73%
High	2098, 16.89%
*Missing*	500, 4.03%
**Urbanicity of the living environment (No., %)**	
Low	4297, 34.60%
Middle	2675, 21.54%
High	5410, 43.56%
*Missing*	38, 0.31%
**Ethnic background (No., %)**	
Dutch origin	9714, 78.21%
Non-Dutch origin, HIC (1st + 2nd generation)	1146, 9.23%
Non-Dutch origin, LMIC (1st + 2nd generation)	1087, 8.75%
*Missing*	473, 4.81%

Abbreviations: No., number; IQR, inter-quartile range; HIC, high-income countries; LMIC, lower- and middle-income countries.

### Mental resilience

**Fig. 2:**
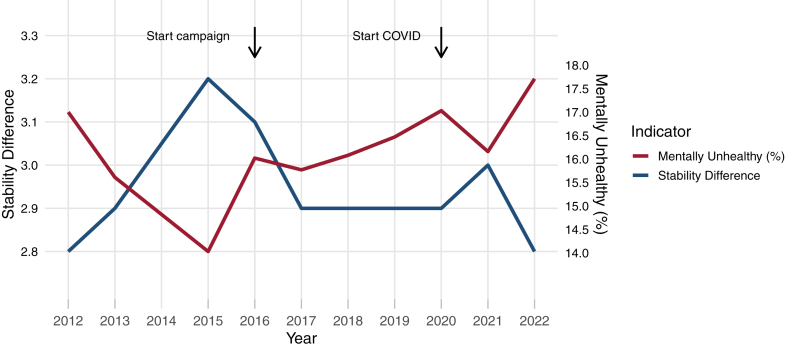
**Overview of the population-level stability difference and percentage of participants that was mentally unhealthy for each year (NB no data was collected in 2014). A higher value of the stability difference is indicative of higher likelihood of the population-level network to be in a healthy state**.

Mental resilience (i.e., stability difference) in the adult population did not significantly differ between the beginning versus the end of the study period (2012 versus 2019; difference in stability difference −0.153; BCa interval −0.359 to 0.0152, p = 0.07; 2012 versus 2022: difference in stability difference, −0.0631; BCa interval −0.262 to 0.153, p = 0.57). When descriptively examining changes over time as presented in Table 2, we observed some favourable changes in resilience indicators before the start of the campaign (2012–2015) in the entire group (node threshold decreased from −16.25 to −19.86, stability difference increased from 2.8 to 3.2), but relatively stable values afterwards, further indicating no increase in mental resilience after start of the campaign (see Fig. 2, Supplementary Figures S3 and S4 for changes in resilience proxies in relation to mental health). Notably, all stability landscapes showed a clear attractor basin at the healthy end of the spectrum (no symptoms present), with 16 instances (17.8%) with a second (less stable) attractor basin at the other end (all symptoms present). For illustration, Supplementary Figure S2 shows the HMI-5 network and stability landscape for 2012 for the entire group. All networks and stability landscapes are provided in the Appendix. Different sociodemographic groups (i.e., gender, social support, urbanicity of the living environment) showed roughly similar trends, except for mental resilience in those experiencing high social support and/or living in urban areas, which showed no improvement in the pre-campaign period (Supplementary Tables S3–S5). Resilience indicators in the entire group did not change after onset of the COVID pandemic, but there were some differential changes in accordance to experienced social support (decreases in those experiencing low support in 2021 and in those experiencing high support in 2022, see Appendix for a full description).

**Table 2:** Changes in resilience indicators the period 2012–2022.

	Year (n)	Node threshold (total, mean, SD)	Network connectivity	Stability difference	No., point-prevalence mentally unhealthy (MHI-5 < 60, scale 0–100)
Pre-campaign	2012 (n = 5644)	−16.25, −3.25 (0.97)	12.9361	2.8	958, 17.00%
	2013 (n = 5239)	−16.85, −3.37 (1.19)	13.2731	2.9	817, 15.61%
	2015 (n = 4438)	−19.86, −3.92 (1.68)	16.0506	3.2	622, 14.03%
Start campaign	2016 (n = 5276)	−18.67, −3.73 (1.43)	14.7956	3.1	844, 16.02%
	2017 (n = 5819)	−18.58, −3.72 (1.52)	14.9121	2.9	917, 15.77%
	2018 (n = 5365)	−18.58, −3.72 (1.43)	14.8091	2.9	861, 16.08%
	2019 (n = 5057)	−18.98, −3.79 (1.49)	15.3089	2.9	832, 16.47%
campaign/COVID	2020 (n = 5615)	−18.93, −3.79 (1.47)	15.3687	2.9	955, 17.03%
	2021 (n = 4986)	−18.92, −3.78 (1.47)	14.9628	3	805, 16.16%
	2022 (n = 5716)	−18.88, −3.78 (1.58)	15.2941	2.8	1011, 17.71%

Abbreviations: SD, standard deviation; No., number; HMI-5, 5-item Mental Health Inventory; COVID, coronavirus disease 2019.

### Trends in mental health and mental healthcare use

There was a small trend change in the percentage of self-reported mental ill-health before (decrease from 17.00% in 2012 to 14.03% in 2015) versus after (stabilizing values from 16.02% in 2016 to 16.47% in 2019) the start of the campaign (Table 2, beta: 0.11, SE: 0.04, p-value = 0.002; Fig. 2), but no differential effects in sociodemographic subgroups (Supplementary Tables S3–S5 and S10–S11). Also, we detected a negative trend change in medication use for depression and/or anxiety (Supplementary Table S6, beta: −0.31, SE 0.12, p = 0.01) and in mental healthcare use in those experiencing medium versus low social support levels (Supplementary Table S8, beta: −0.31; SE: 0.15; p-value = 0.04), yet an increasing trend change in mental healthcare use in participants of non-Dutch origin with a LMIC background versus participants of Dutch origin (Supplementary Table S11, beta: 0.67, SE: 0.25, p = 0.01). Output of all the interrupted time-series analyses is provided in the legends of the respective tables (Supplementary Tables S3–S11).

## Discussion

This study examined the impact of a nationwide mental health campaign in the Netherlands aiming to decrease the prevalence of MDD, on population-level mental resilience, as well as self-reported mental health and mental healthcare-use, in a longitudinal cohort representative of Dutch adults. Utilizing a dynamical systems perspective, we found no changes in mental resilience as derived from population-level psychological networks in the period after (2016–2019, additional comparison up to 2022) versus before (2012–2015) start of the campaign, also not in accordance to gender, social support, or urbanicity. Additionally, we detected a small increasing trend change in the percentage of participants reporting to be mentally unhealthy (beta: 0.11, SE: 0.04, p-value = 0.002), a decreasing trend change in medication use for depression and/or anxiety (beta: −0.31, SE 0.12, p = 0.01), as well as differential trend changes in mental healthcare use (medium versus low social support, beta: −0.31; SE: 0.15; p-value = 0.04; non-Dutch with a LMIC background versus Dutch origin, beta: 0.67, SE: 0.25, p = 0.01).

To our knowledge, this is the first study to use a dynamical systems perspective to examine the impact of a macro-level intervention on population-level mental resilience, in line with the increasing understanding of resilience as a dynamic concept rather than a stable trait, as well as recent calls to include resilience as outcome in mental health intervention studies.^10^^,^^20^^,^^23^ We viewed resilience as the inclination of group-level psychological networks to ‘withstand’ being in an unhealthy state (i.e., having a majority of symptoms active) and/or their tendency to shift back into a healthy state (Textbox 1).^12^^,^^18^ The finding that the campaign was not associated with an increase in population-level mental resilience could have several reasons. First, its working mechanism, emphasizing knowledge and awareness, and social support, may not be sufficient to achieve its goals in terms of decreasing prevalence of MDD. Indeed, prospective evidence that fostering social support prevents MDD onset is scarce.^36^^,^^37^ Moreover, talking about mental health problems may increase awareness of experiencing—or even deteriorate–internalizing symptoms (i.e., co-rumination) and increase self-reporting in questionnaires, which could in part explain the increase in self-reported mental health problems, as found in our study.^38^^,^^39^ Second, the reach and impact of the campaign itself may have been insufficient. The campaign had a below-average reach in the general public in its initial years (2016–2018) of around 60% compared to around 80% for other government campaigns, yet this increased in later years (2021–2022) to a reach of around 66–74%. Also, Previous evaluations of the campaign found that goals with regard to changing attitudes were not reached, while this proved to be an important aspect in successful campaigns in other countries.^3^, ^4^, ^5^, ^6^^,^^8^^,^^9^ This raises the question whether, even if the underlying mechanisms work, measurable impact can be expected. Third, the current time span may be too short to capture changes in mental resilience. From a dynamical systems perspective, while the campaign may impact reinforcing mechanisms (e.g., seeking support) which could prevent a shift into an unhealthy state, it may be that its impact on the tendency to be shifted in the first place takes longer.^10^^,^^20^ Moreover, sustainable improvement of mental resilience and population-level mental health may require a multi-level systems approach, addressing factors from the micro-to the macro-level, that ‘hit the system’ in several places.^40^

The lack of a larger effect in women versus men or in those experiencing high versus low social support is in contrast with our hypotheses. First, while women may use coping strategies involving social support and expressing emotions more often, there is also evidence that the protective effect of social support for MDD specifically is not gender-specific.^36^ Second, the absence of an increase in mental resilience in those experiencing high social support may be due to the small proportion that was mentally unhealthy in the first place (mean 7.52% for all years, versus 34.32% in the low support group). Notably, we did observe more favourable outcomes in mental resilience across all years in males versus females, those experiencing higher versus lower social support, and those residing in less versus more urbanized areas. Importantly, the notion that disparities within the different sociodemographic subgroups seemed unaddressed (and even possibly aggravated during the COVID-19 pandemic) raises questions on the equability of large-scale mental health campaigns. Moreover, while the upward trend in self-reported mental ill-health is line with other recent findings in the Netherlands of an increase in depressive symptoms and MDD prevalence, it is uncertain whether increases might have been higher without the campaign.^41^ In addition, the seemingly contradictory finding on an increase in self-reported mental health problems and a decrease in medication use for depression and/or anxiety may be explained by the fact that medication is not the primary choice of treatment for milder cases of these disorders in Dutch guidelines.^42^^,^^43^

This study has several limitations. Firstly, given the campaign was rolled out nationally, there was no control group. However, it has been proposed that longitudinal follow-up in the same group is more informative when studying resilience then than comparing separate groups.^32^ Moreover, interrupted time-series analysis is deemed a strong method for examining changes in trends when a control group is not available.^32^ Second, the chosen resilience metrics may not properly capture the systems dynamics concept of resilience. While some evidence links higher network node thresholds and lower connectivity to more favourable mental health outcomes, there are also studies that show no—or the opposite—association.^17^ Moreover, we find that, in a relatively healthy population, higher connectivity may reflect the tendency of symptoms to keep each other *deactivated* and thus the network in a healthy state.^19^^,^^44^ Third, we were unable to correct resilience parameters for macro-economic trends. This may have influenced results, as there may still have been impact of the global financial crisis and European debt crisis, especially in the pre-campaign period.^45^ However, self-reported mental health problems increased after start of the campaign (also corrected for macro-economic trends), which does not suggest a positive change in mental resilience. Fourth, seasonal mood fluctuations may have influenced the findings, as the 2015 health questionnaire was administered in July/August, as opposed to November/December in other years. However, a recent study in a Dutch general population sample showed no seasonal effect in self-reported mood and affect.^46^ Fifth, given the naturalistic setting, results cannot be solely attributed to the campaign, as we had no way of delineating possible impact of large societal events (e.g., COVID or economic developments) or other public campaign such as one on suicide and mental health (2017–2018) on mental resilience.^7^ Nonetheless, the interrupted time-series analyses that were corrected for macro-economic trends and did not include the COVID period also showed no improvement in mental health. Moreover, given the short duration of the other aforementioned campaign in comparison to the campaign examined in our study, we expect no major impact on our results. Lastly, future studies on this topic could benefit from collecting data that enables to examine the relationship between campaign awareness and resilience, as well as involving people with lived experience, to get more specific insights into the impact of the campaign.

In conclusion, while the naturalistic setting of this study refrains us from attributing results solely to the campaign, we also found no indication that a nationwide mental health campaign in the Netherlands improved population-level mental resilience from a dynamical systems perspective. There was also no improvement in self-reported mental health. Our findings underscore the need to strengthen the evidence-base for effective and equitable interventions for improving mental resilience and combatting MDD burden.

## Contributors

Concept and design: CL Bockting, F Pijpers, A Lok, M Nicolaou, K Stronks, JM van der Wal.

Acquisition, analysis, or interpretation of data: F Pijpers, JM van der Wal, G Lunansky, A Korezelidou.

Drafting of the manuscript: JM van der Wal, F Pijpers, A Lok, M Nicolaou, K Stronks, CL Bockting.

Critical review of the manuscript for important intellectual content: JM van der Wal, F Pijpers, G Lunansky, A Lok, M Nicolaou, A Korezelidou, K Stronks, CL Bockting.

Data access and verification: F Pijpers, JM van der Wal.

Statistical analysis: F Pijpers, JM van der Wal, G Lunansky.

Obtained funding: CL Bockting.

Supervision: F Pijpers, CL Bockting, K Stronks, A Lok, M Nicolaou.

Decision to submit: CL Bockting, F Pijpers, A Lok, M Nicolaou, K Stronks, JM van der Wal.

## Data sharing statement

The published LISS panel data are freely available for researchers under certain conditions, which can be found on their website. Per the rules and regulations, downloaded data is for personal use only and cannot be made available to third parties.

## Declaration of interests

Gabriela Lunansky received an internal postdoc grant from the Amsterdam University Medical Center.

## Appendix A Supplementary data

All networks and landscapes for LRH
