# Supplementary material for: Effect of a national mental health campaign on population mental resilience in the Netherlands: a retrospective longitudinal cohort analysis using a dynamical systems perspective
Source: Lancet Reg Health Eur. 2025 Sep 4;58:101434. doi: 10.1016/j.lanepe.2025.101434 (PMC12624795; doi:10.1016/j.lanepe.2025.101434)
Supplement: All networks and landscapes for LRH [file mmc1.pdf]

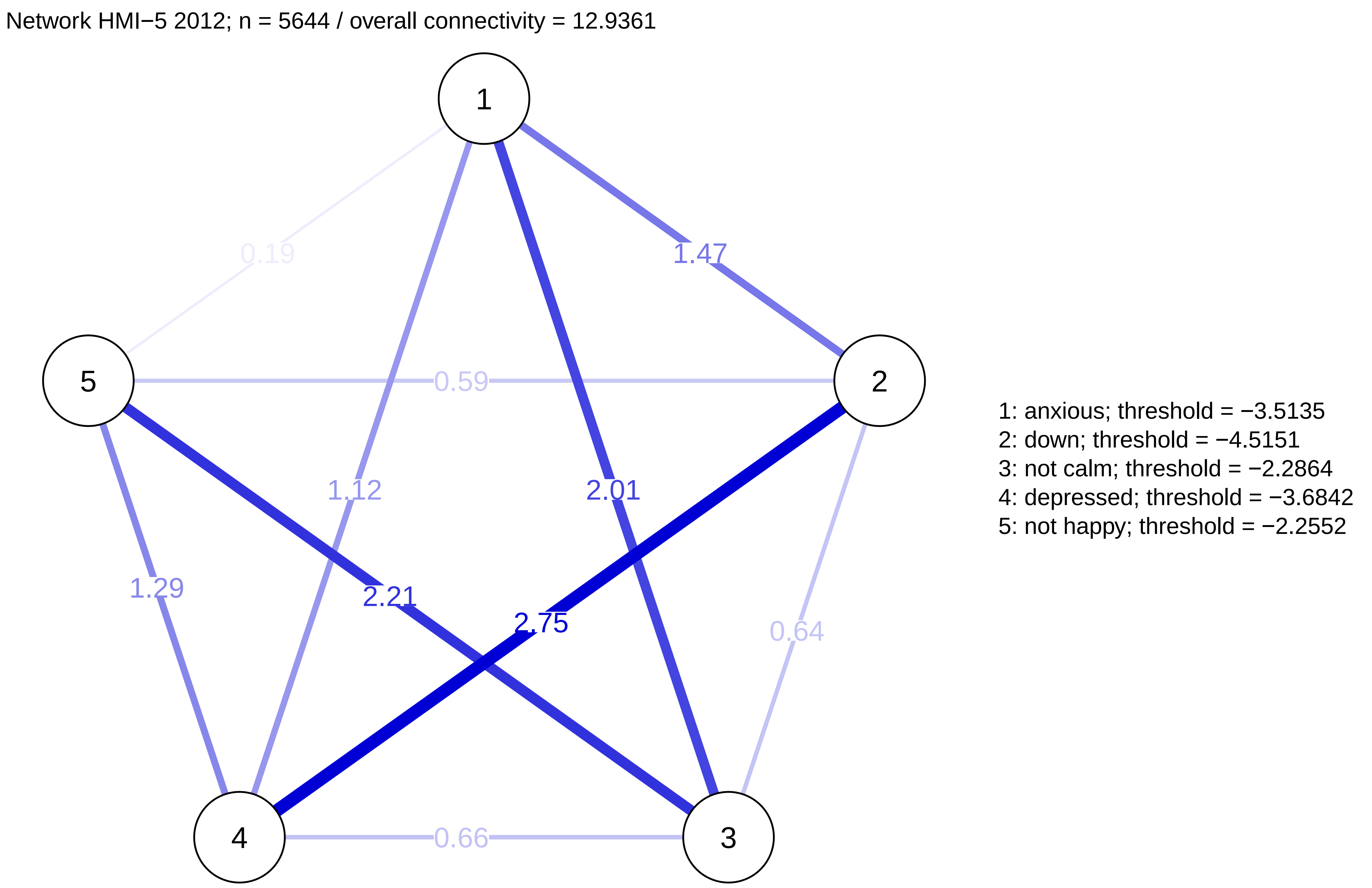
Network HMI−5 2012; n = 5644 / overall connectivity = 12.9361
1
2
3
4
5
0.19
1.47
0.59
1.12
2.01
1.29
2.21
2.75
0.64
0.66
1: anxious; threshold = −3.5135
2: down; threshold = −4.5151
3: not calm; threshold = −2.2864
4: depressed; threshold = −3.6842
5: not happy; threshold = −2.2552

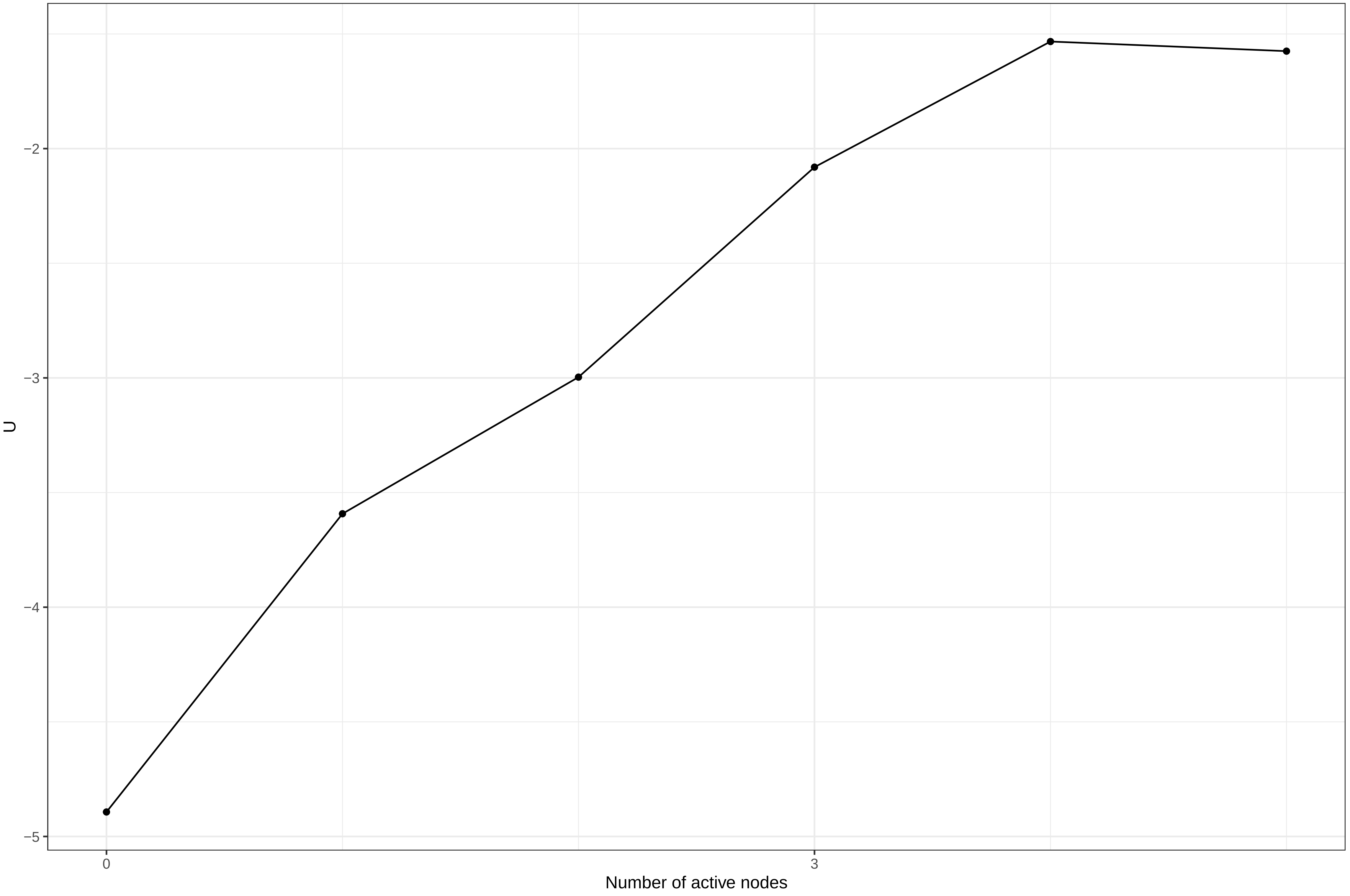

U
−2
−3
−4
−5
0
3
Number of active nodes

Network HMI−5 2013; n = 5239 / overall connectivity = 13.2731

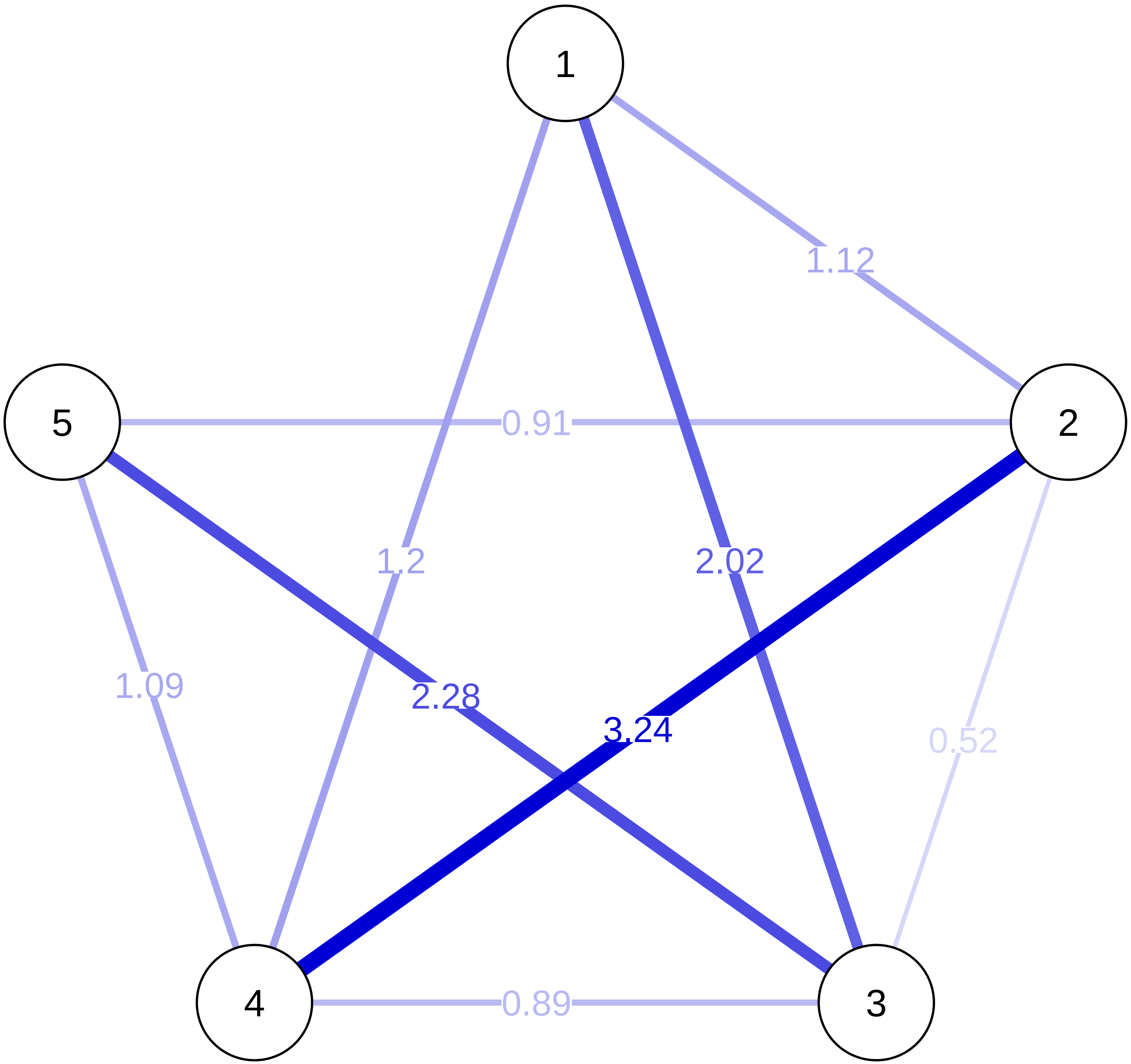

1: anxious; threshold = −3.7721
2: down; threshold = −4.8978
3: not calm; threshold = −2.2275
4: depressed; threshold = −3.8553
5: not happy; threshold = −2.1001

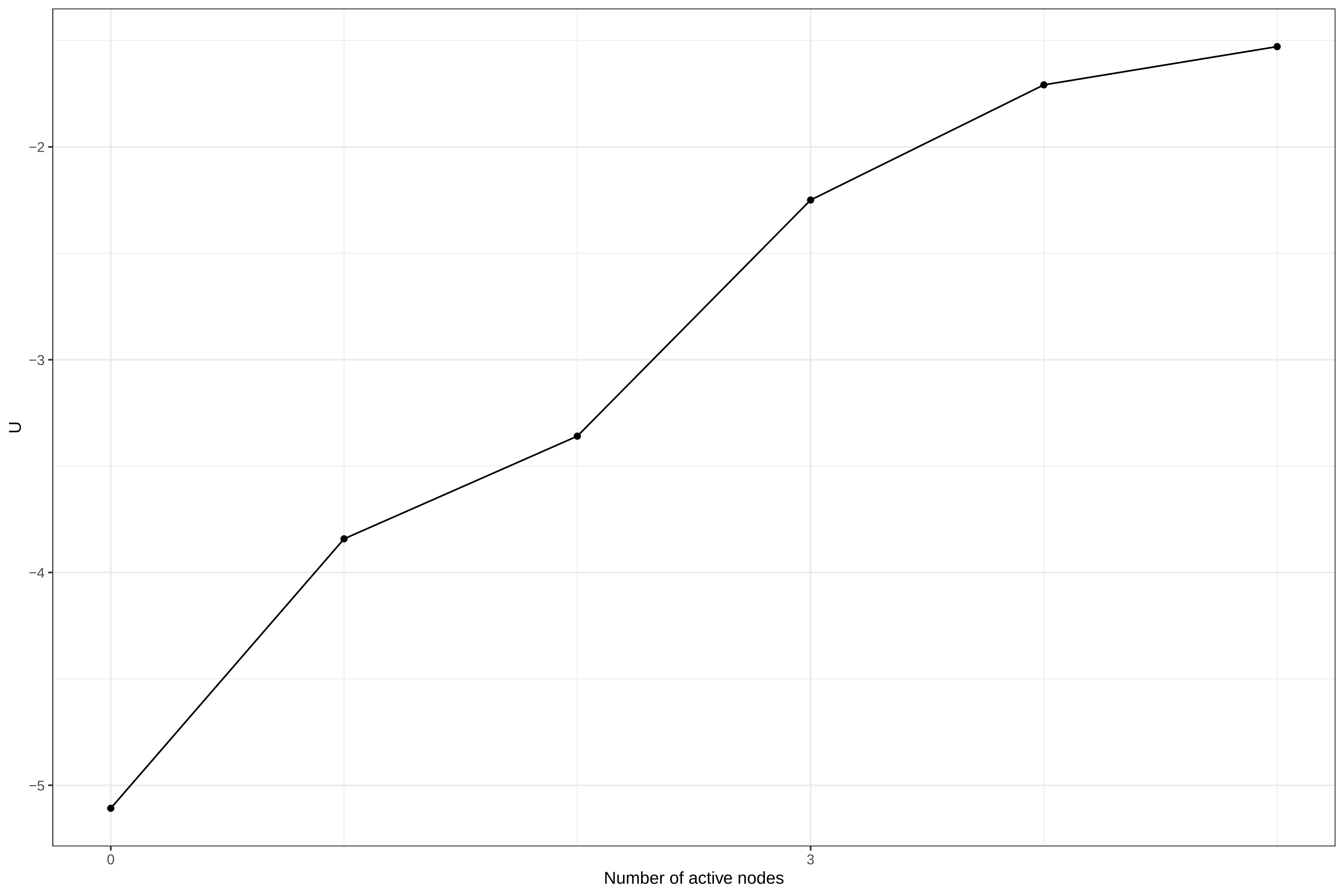

−2
−3
−4
−5
U
0
3
Number of active nodes

Network HMI−5 2015; n = 4438 / overall connectivity = 16.0506

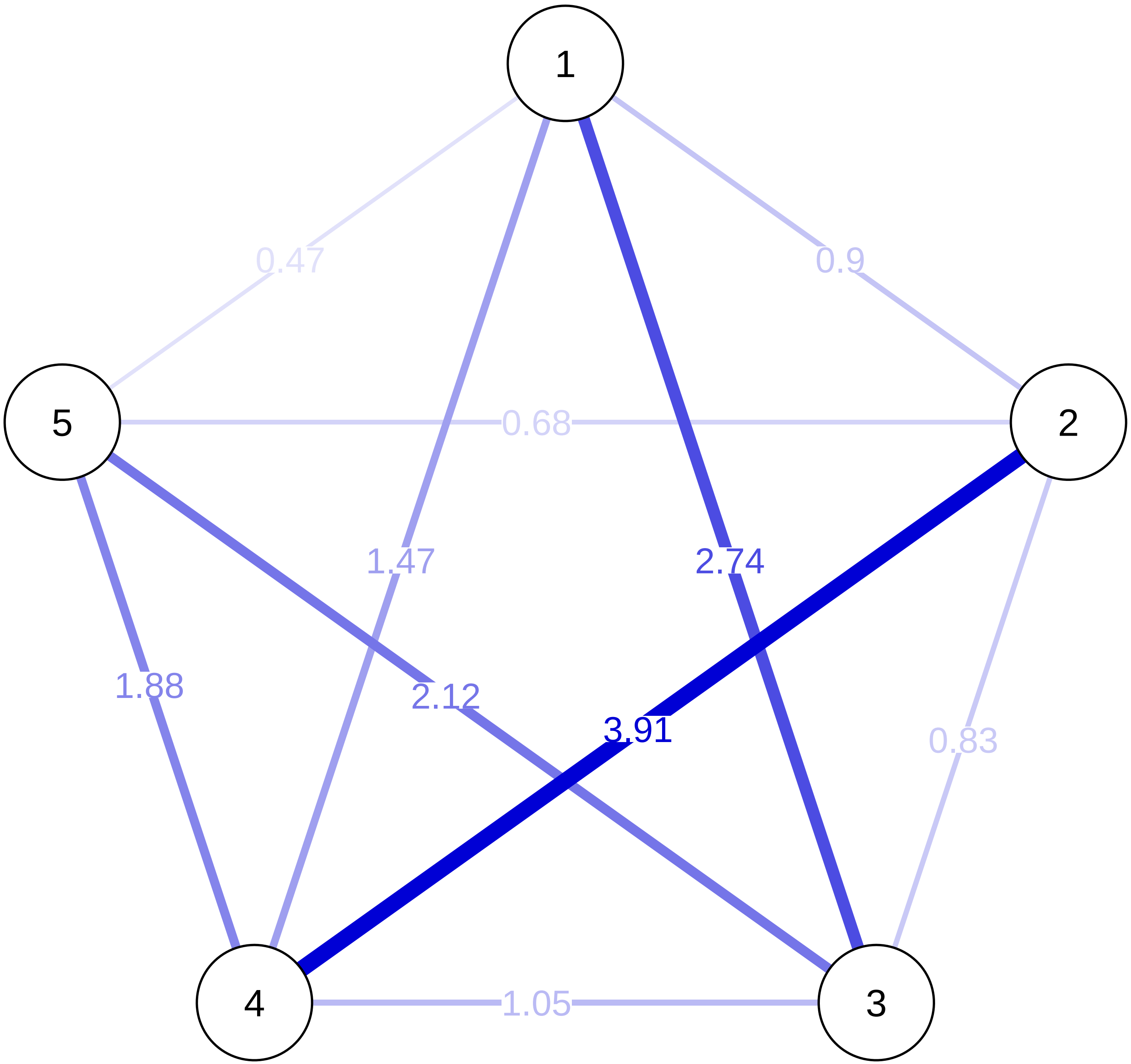

1: anxious; threshold = −4.8891
2: down; threshold = −5.6743
3: not calm; threshold = −2.4399
4: depressed; threshold = −4.9402
5: not happy; threshold = −1.9177

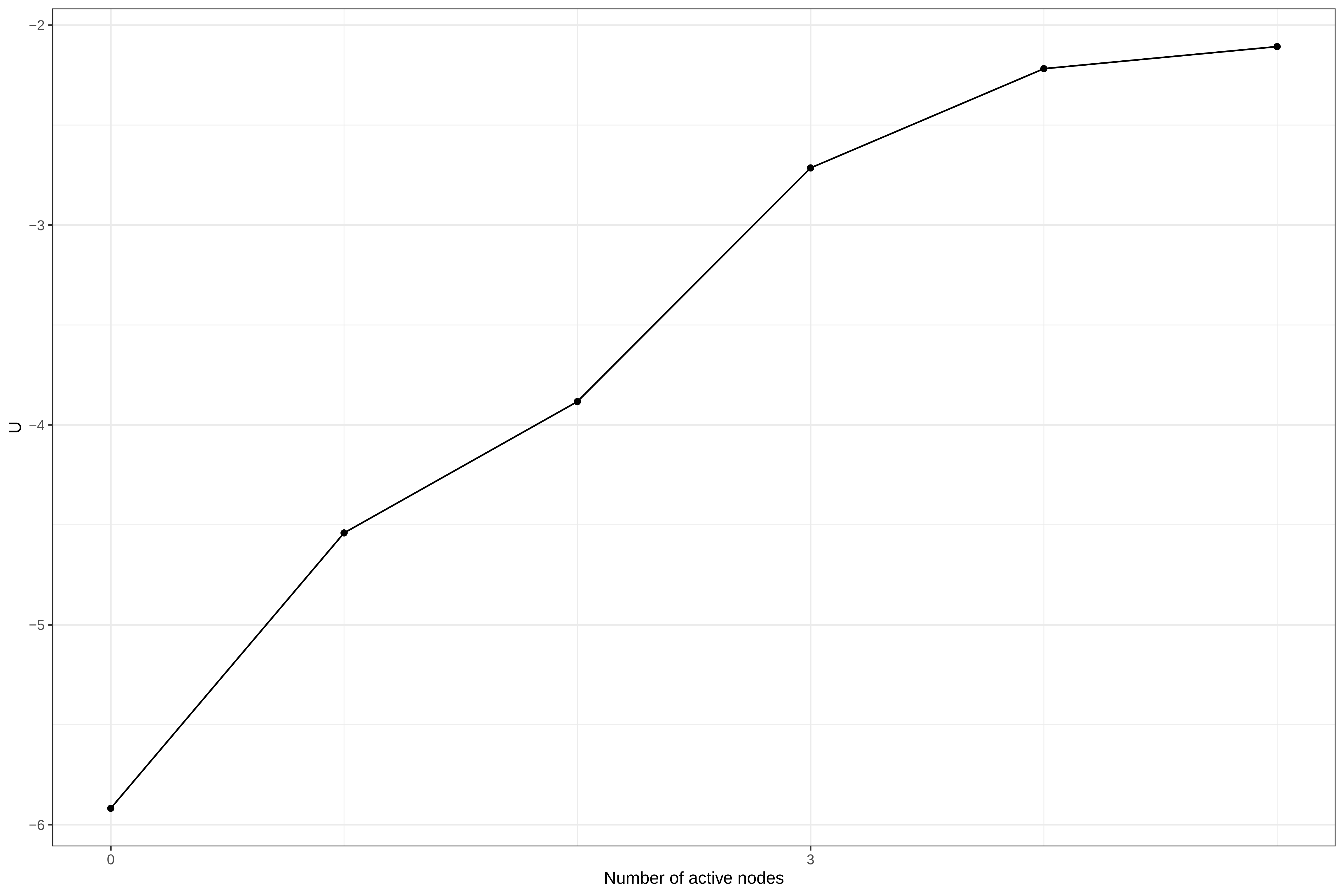

−2
−3
−4
−5
−6
U
0
3
Number of active nodes

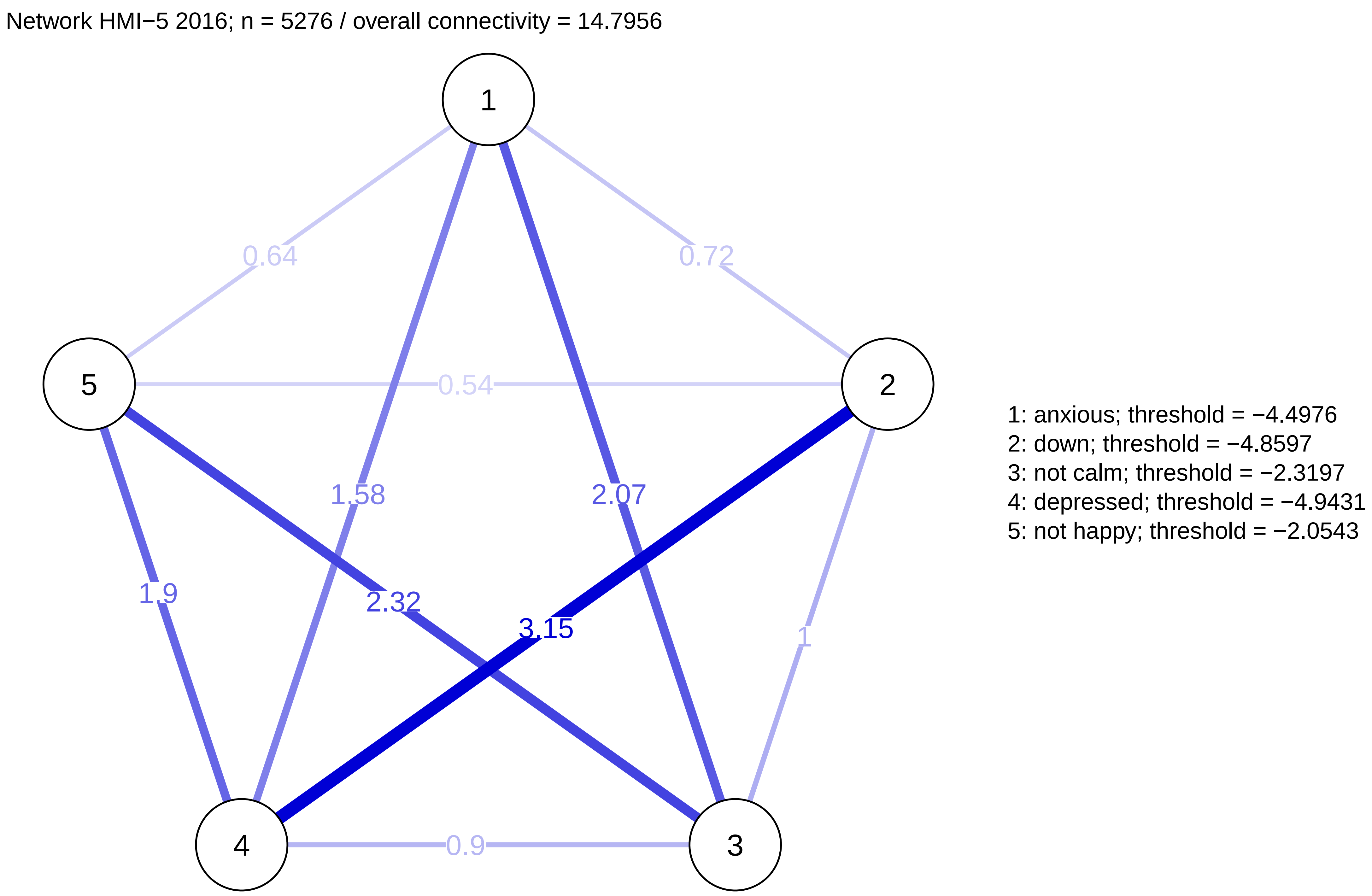
Network HMI−5 2016; n = 5276 / overall connectivity = 14.7956
1
2
3
4
5
0.64
0.72
0.54
1.58
2.07
1.9
2.32
3.15
1
0.9
1: anxious; threshold = −4.4976
2: down; threshold = −4.8597
3: not calm; threshold = −2.3197
4: depressed; threshold = −4.9431
5: not happy; threshold = −2.0543

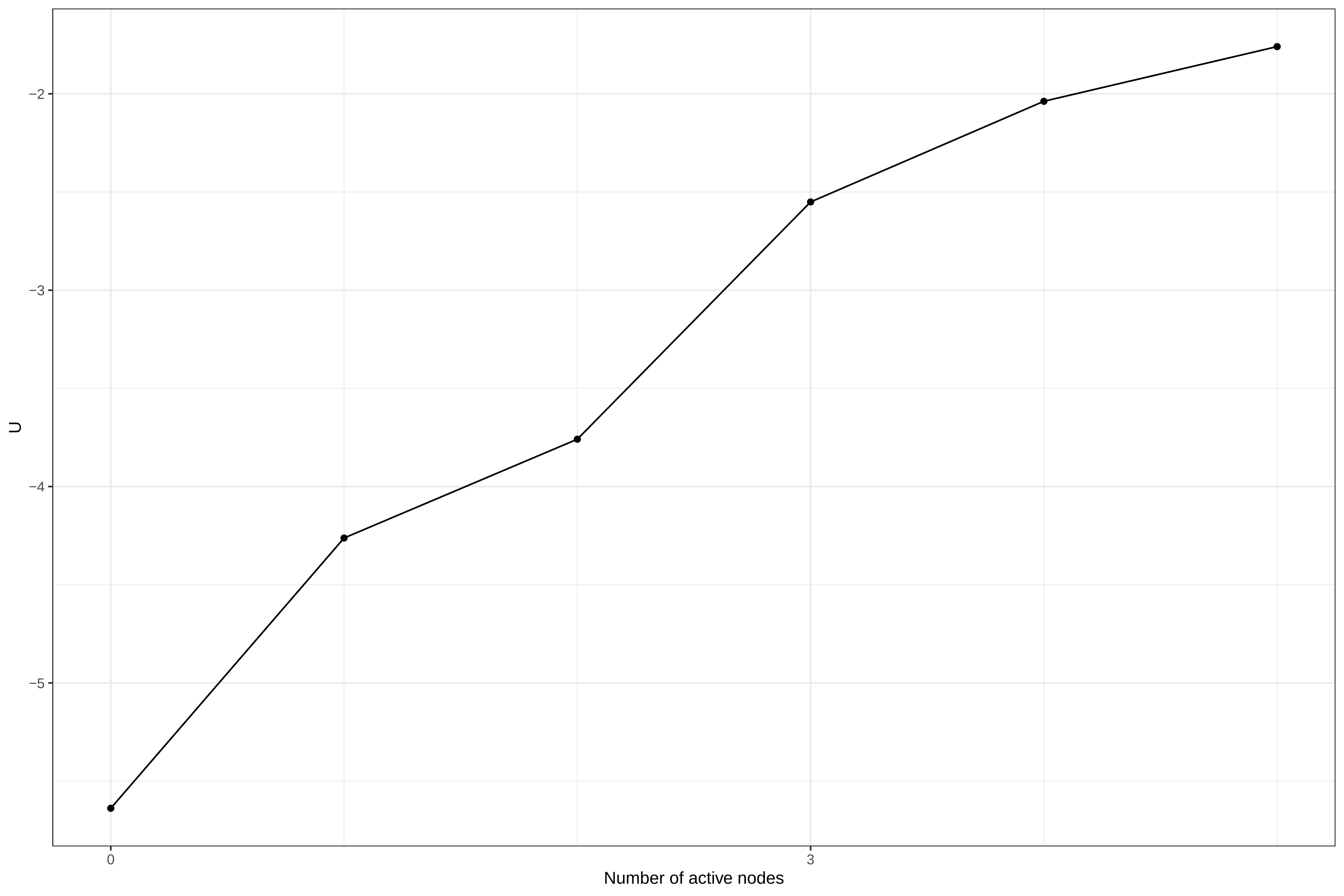

U
−2
−3
−4
−5
0
3
Number of active nodes

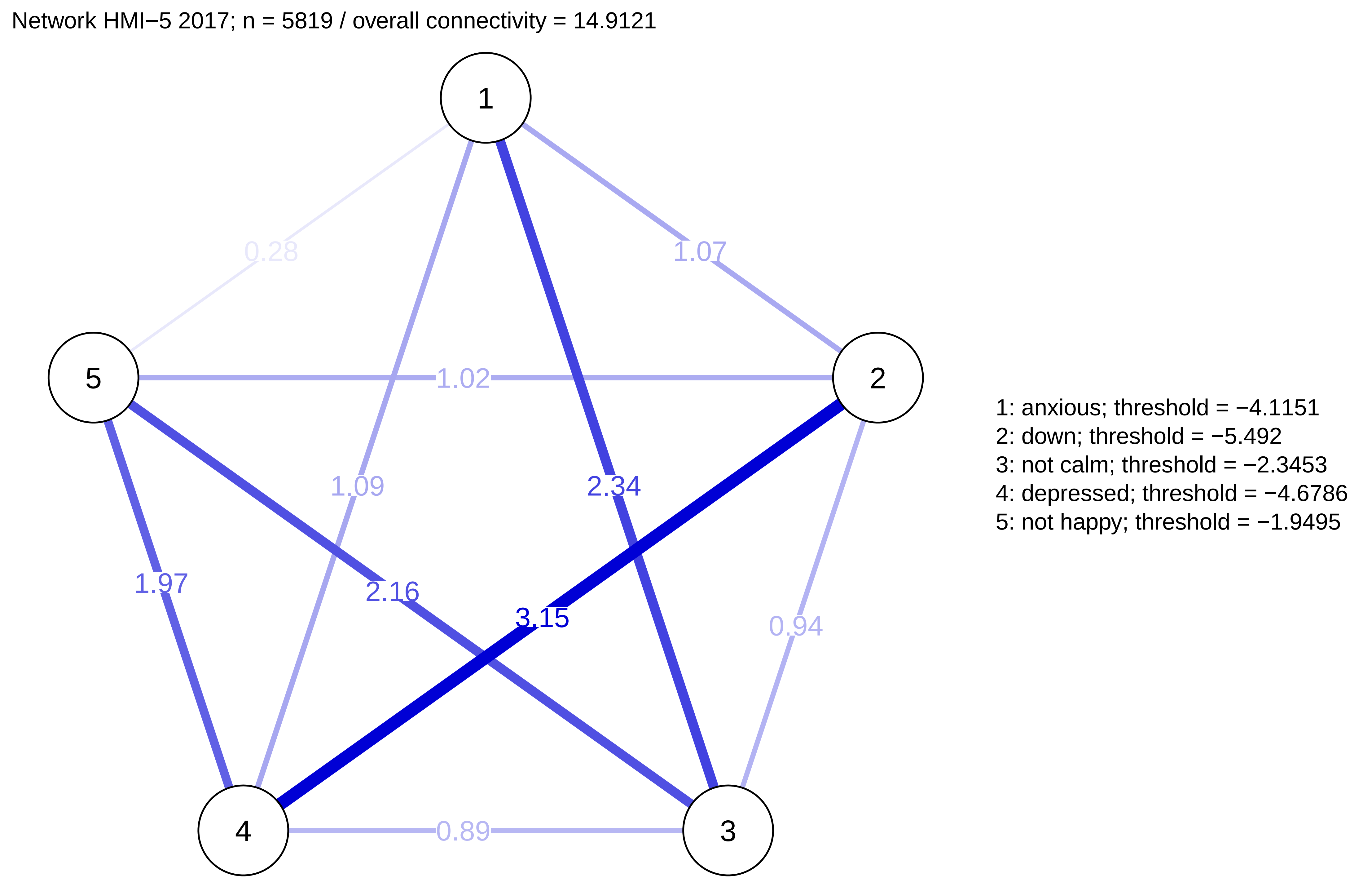

Network HMI−5 2017; n = 5819 / overall connectivity = 14.9121
1
2
3
4
5
0.28
1.07
1.02
1.09
2.34
1.97
2.16
3.15
0.94
0.89
1: anxious; threshold = −4.1151
2: down; threshold = −5.492
3: not calm; threshold = −2.3453
4: depressed; threshold = −4.6786
5: not happy; threshold = −1.9495

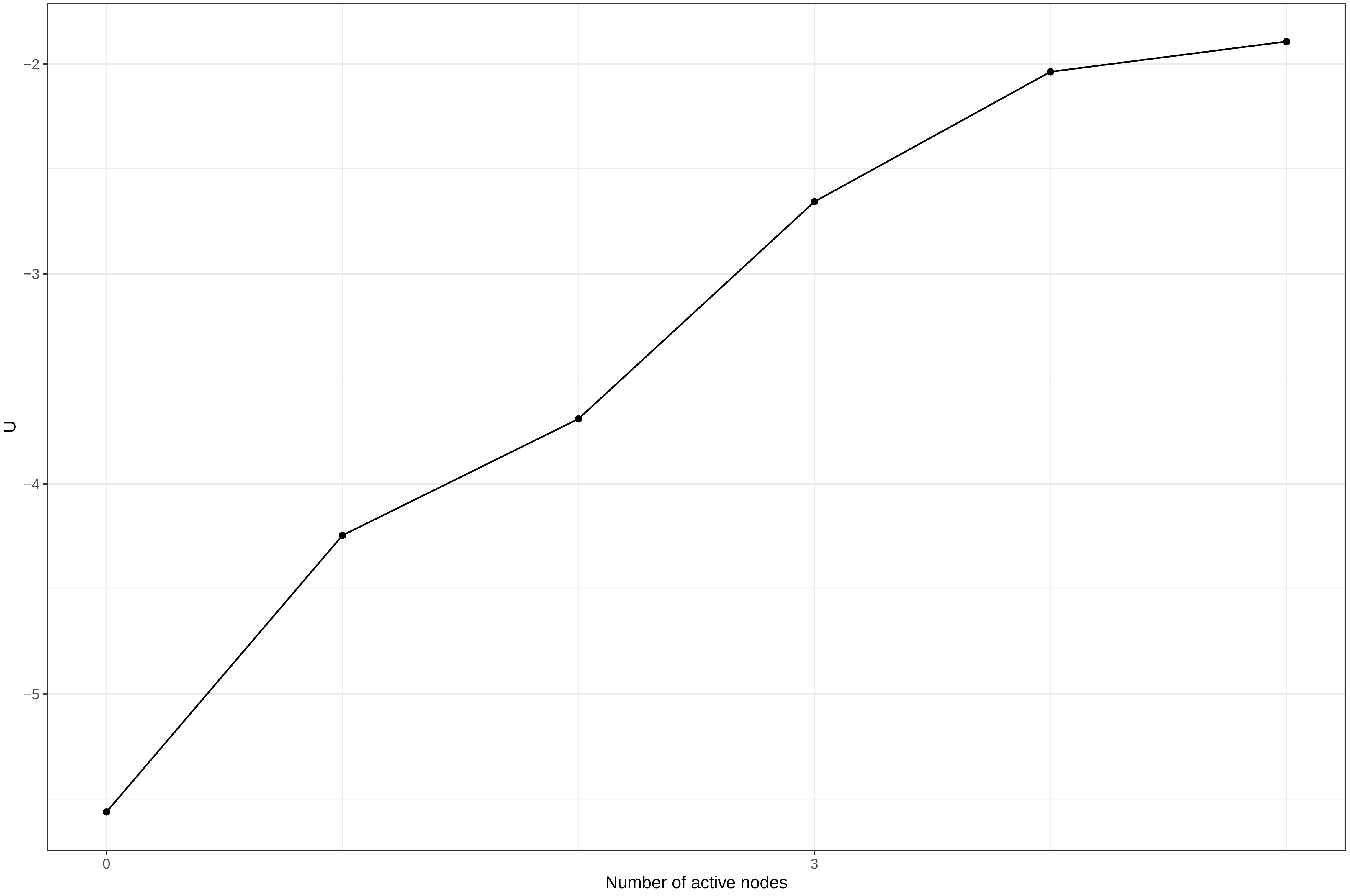

U
−2
−3
−4
−5
0
3
Number of active nodes

Network HMI−5 2018; n = 5365 / overall connectivity = 14.8091

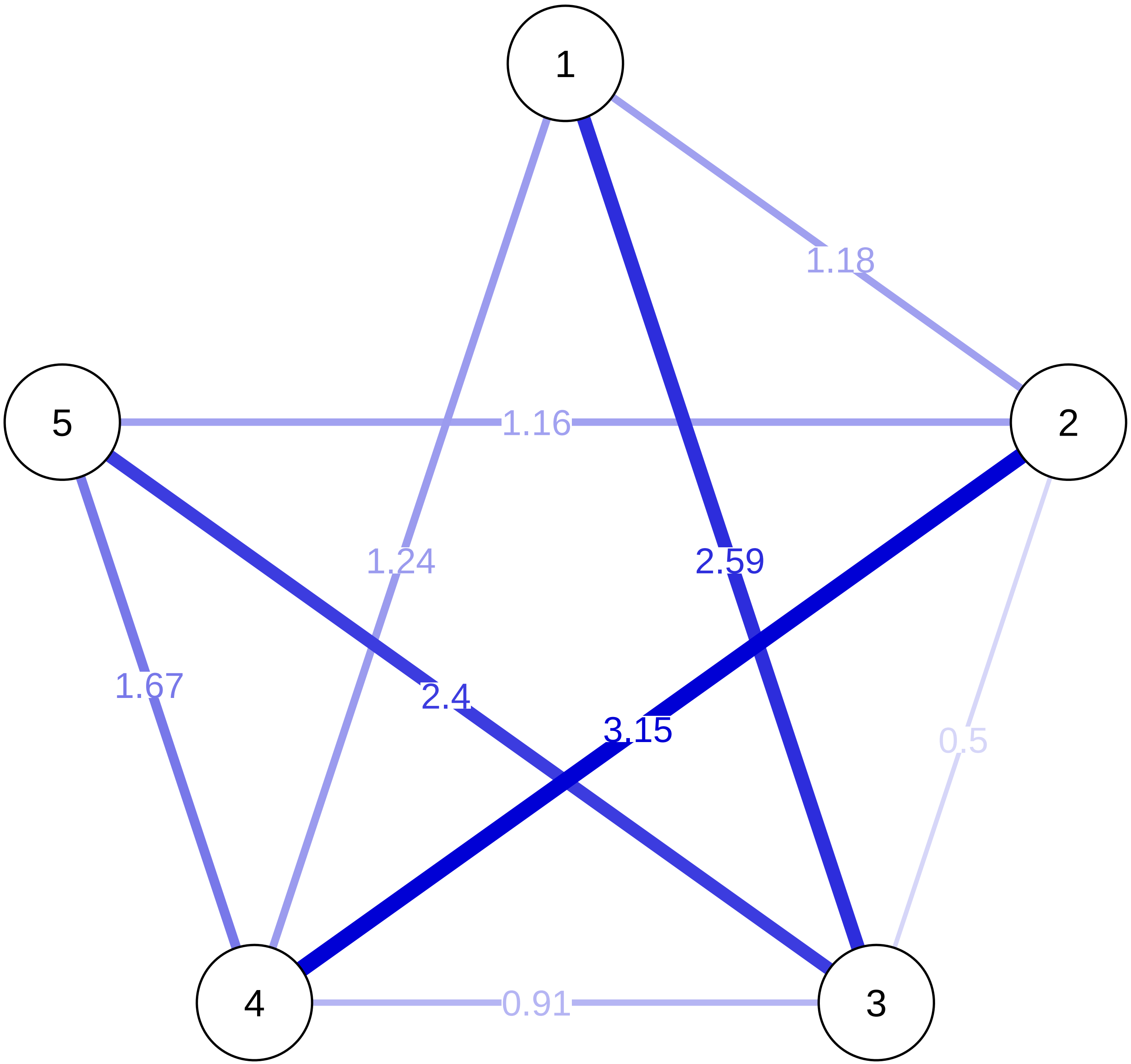

1: anxious; threshold = −3.9938
2: down; threshold = −5.4386
3: not calm; threshold = −2.4803
4: depressed; threshold = −4.6143
5: not happy; threshold = −2.0504

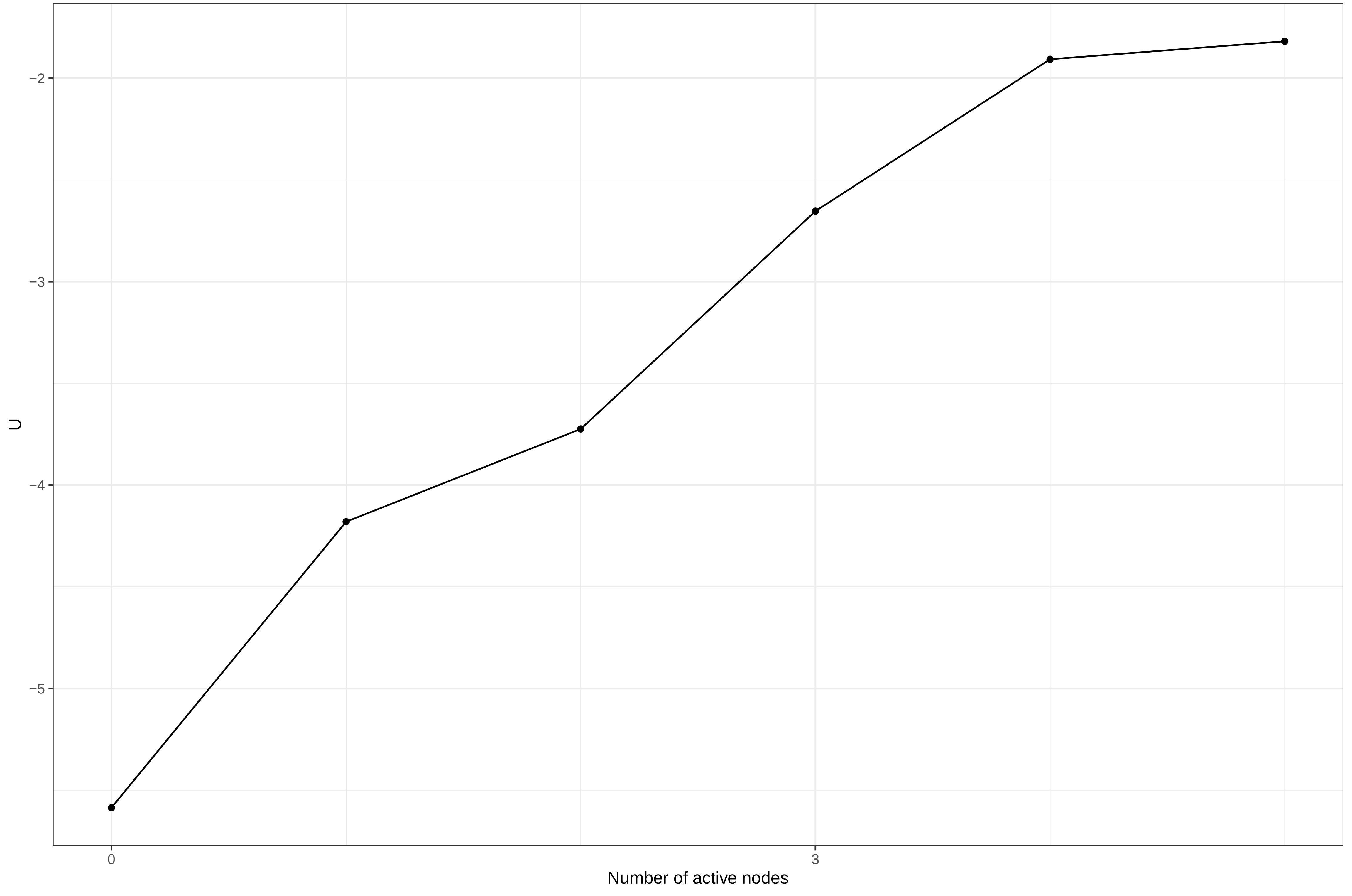

−2
−3
−4
−5
U
0
3
Number of active nodes

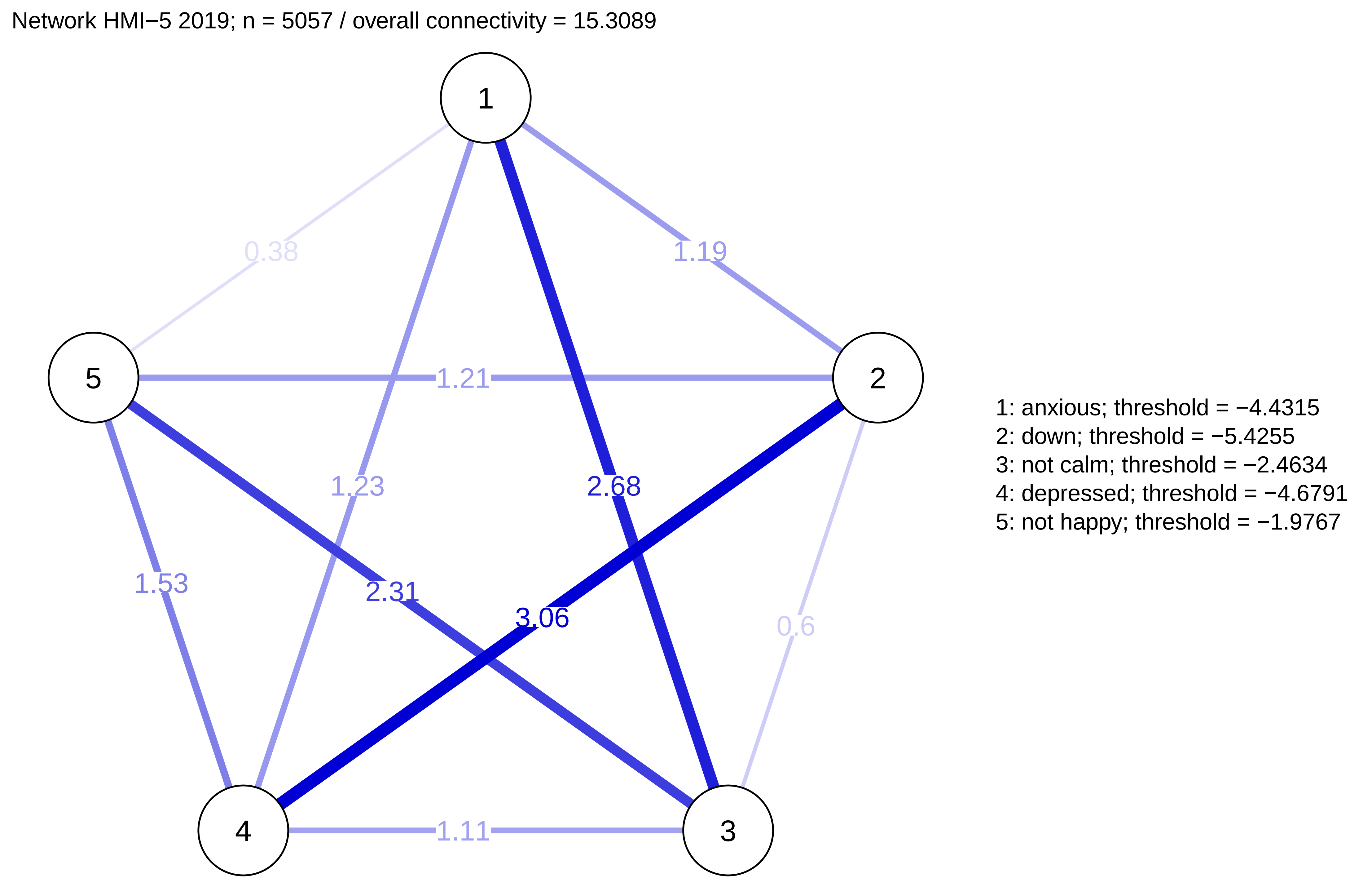
Network HMI−5 2019; n = 5057 / overall connectivity = 15.3089
1
2
3
4
5
0.38
1.19
1.21
1.23
2.68
1.53
2.31
3.06
0.6
1.11
1: anxious; threshold = −4.4315
2: down; threshold = −5.4255
3: not calm; threshold = −2.4634
4: depressed; threshold = −4.6791
5: not happy; threshold = −1.9767

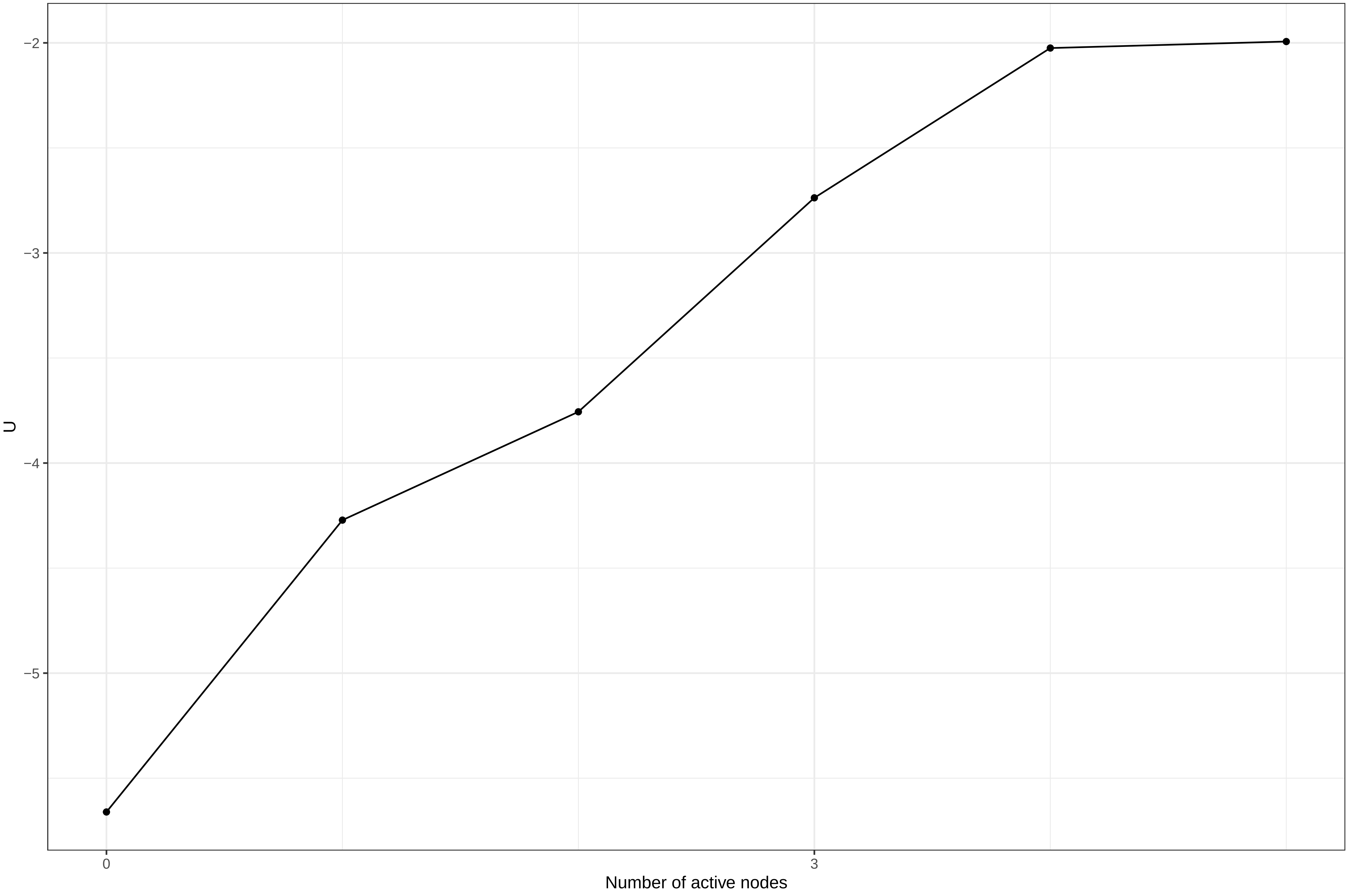

−2
−3
−4
−5
U
0
3
Number of active nodes

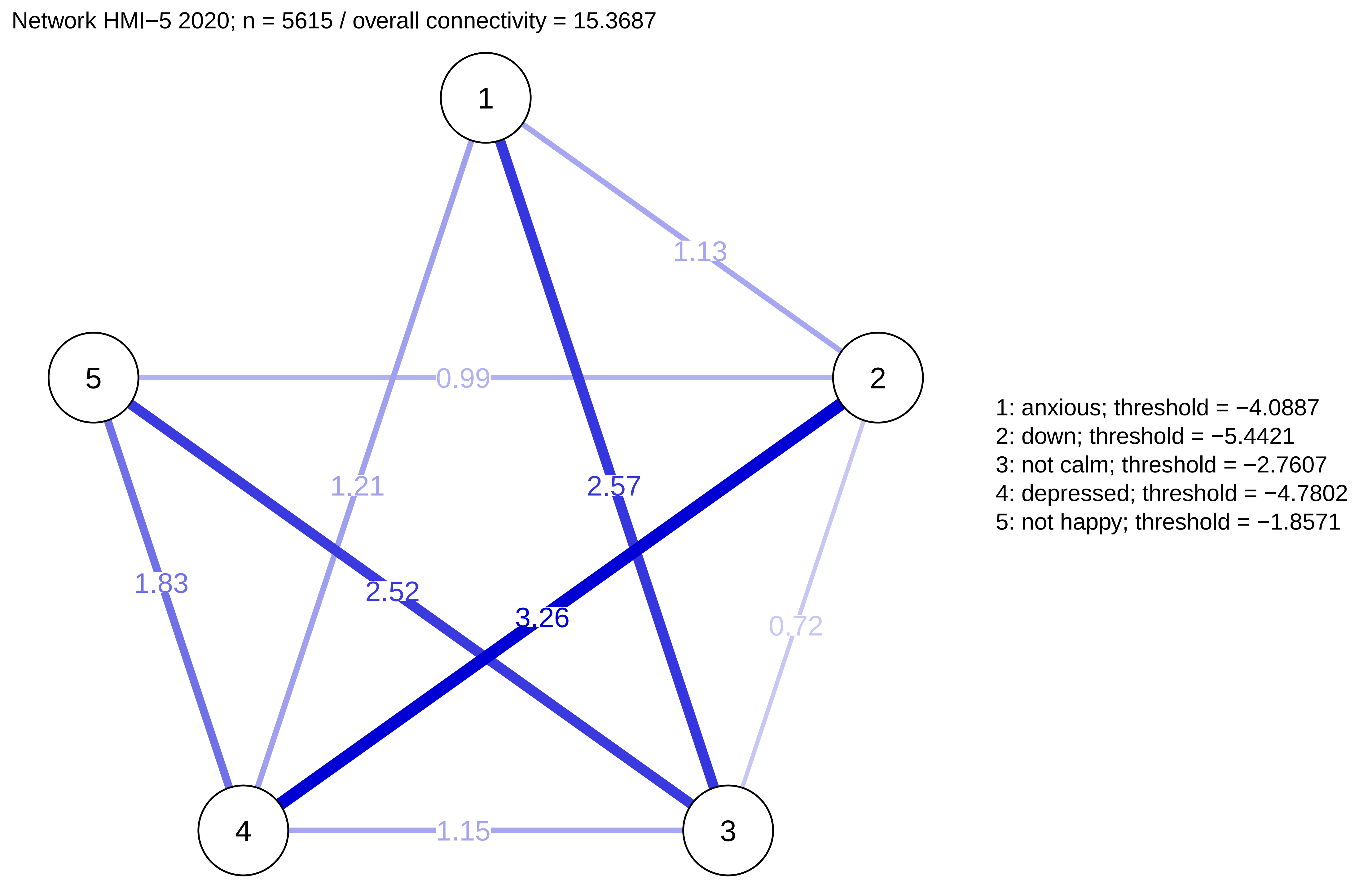

Network HMI−5 2020; n = 5615 / overall connectivity = 15.3687
1
2
3
4
5
1.13
0.99
1.21
2.57
1.83
2.52
3.26
0.72
1.15
1: anxious; threshold = −4.0887
2: down; threshold = −5.4421
3: not calm; threshold = −2.7607
4: depressed; threshold = −4.7802
5: not happy; threshold = −1.8571

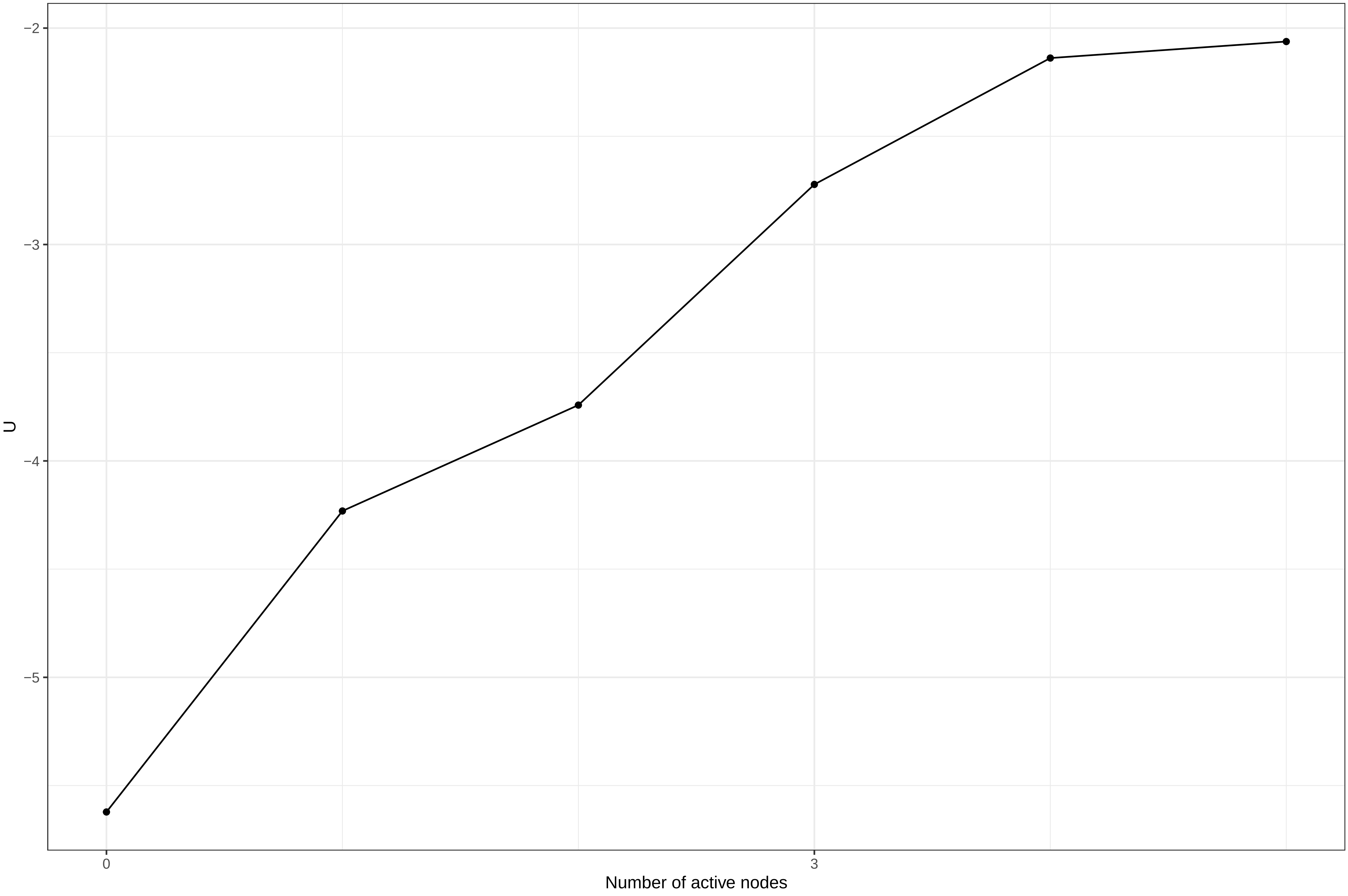

−2
−3
−4
−5
U
0
3
Number of active nodes

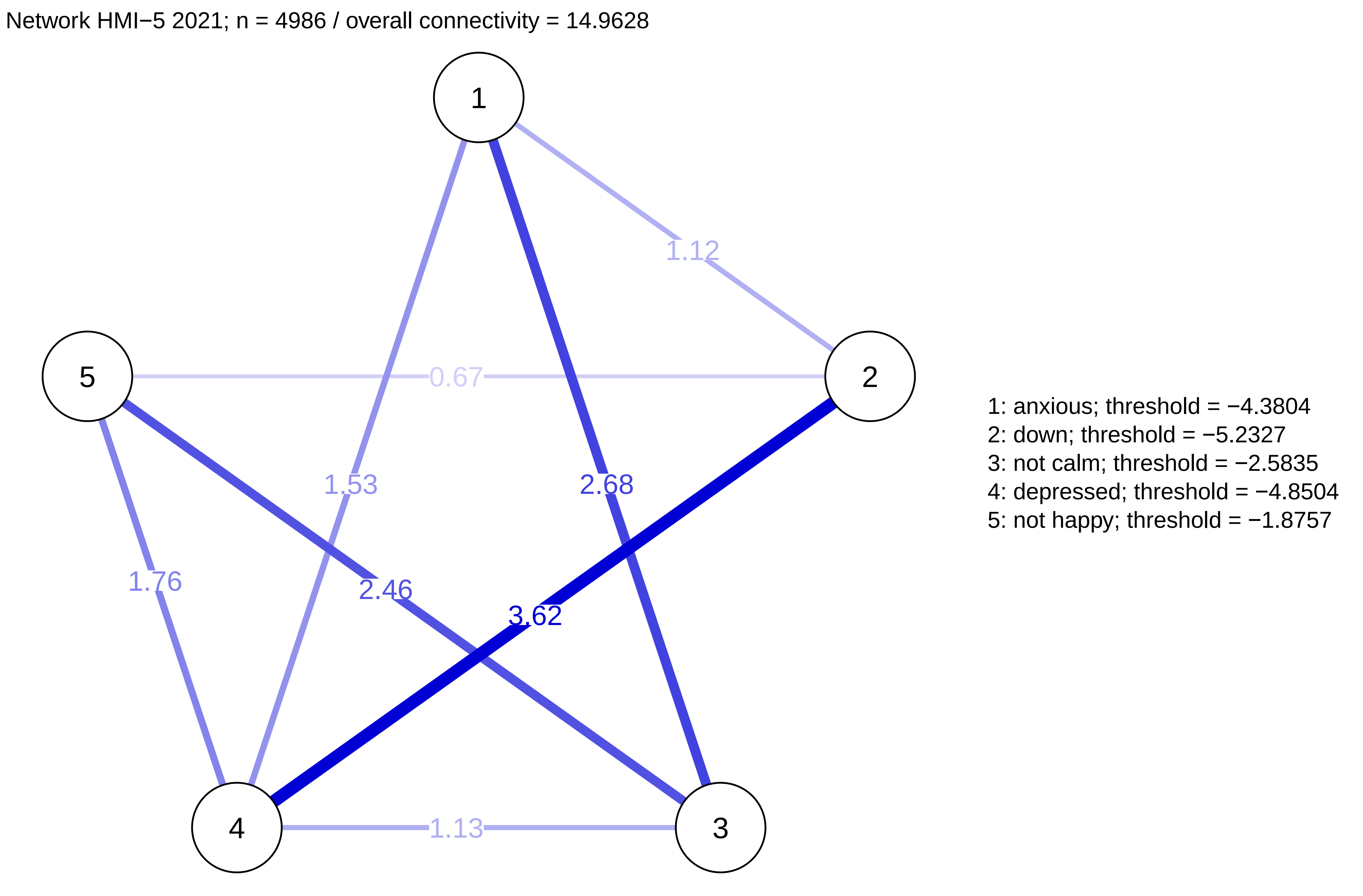

Network HMI−5 2021; n = 4986 / overall connectivity = 14.9628
1
2
3
4
5
1.12
0.67
1.53
2.68
1.76
2.46
3.62
1.13
1: anxious; threshold = −4.3804
2: down; threshold = −5.2327
3: not calm; threshold = −2.5835
4: depressed; threshold = −4.8504
5: not happy; threshold = −1.8757

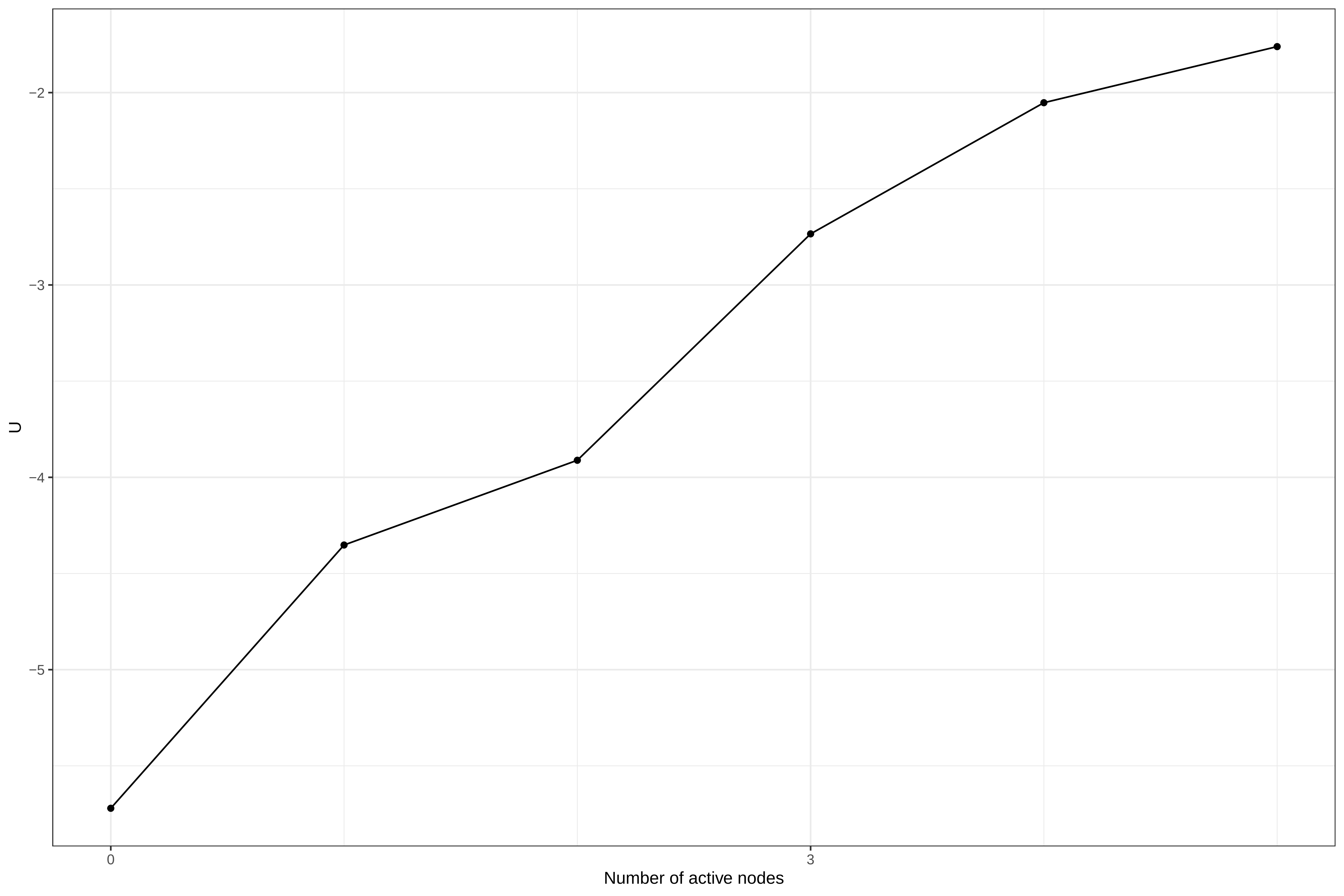

−2
−3
−4
−5
U
0
3
Number of active nodes

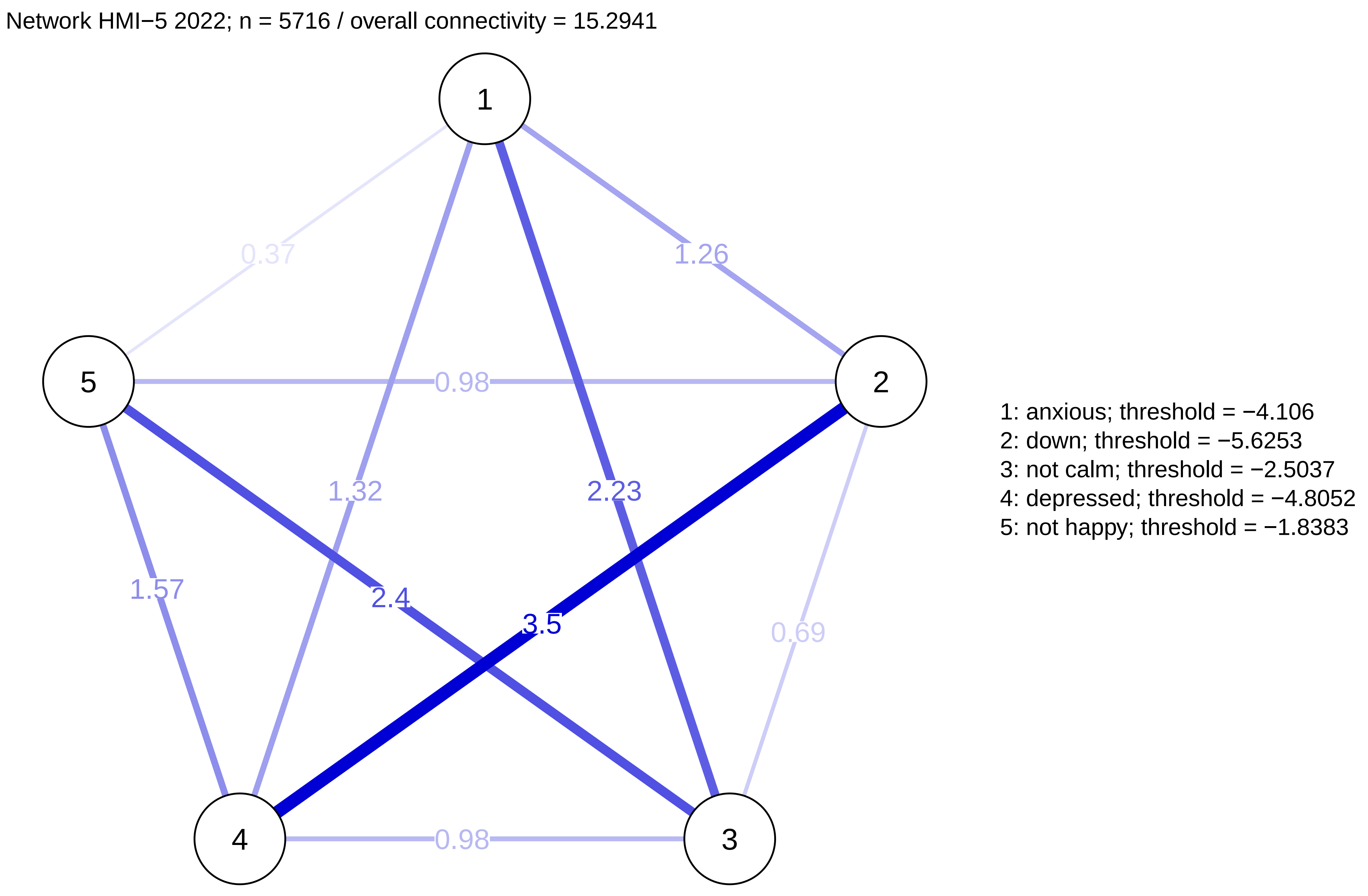
Network HMI−5 2022; n = 5716 / overall connectivity = 15.2941
1
2
3
4
5
0.37
1.26
0.98
1.32
2.23
1.57
2.4
3.5
0.69
0.98
1: anxious; threshold = −4.106
2: down; threshold = −5.6253
3: not calm; threshold = −2.5037
4: depressed; threshold = −4.8052
5: not happy; threshold = −1.8383

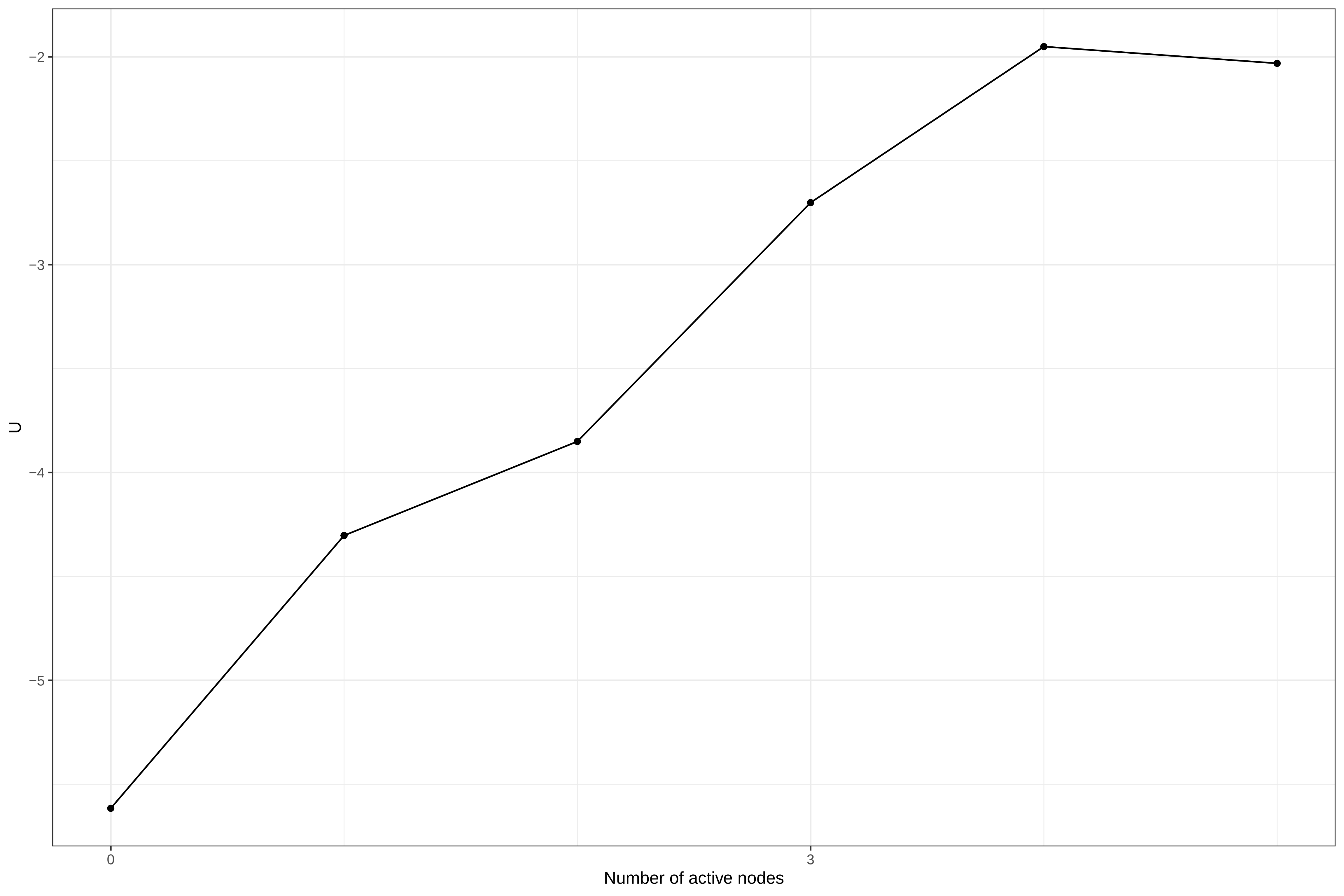

U
−2
−3
−4
−5
0
3
Number of active nodes

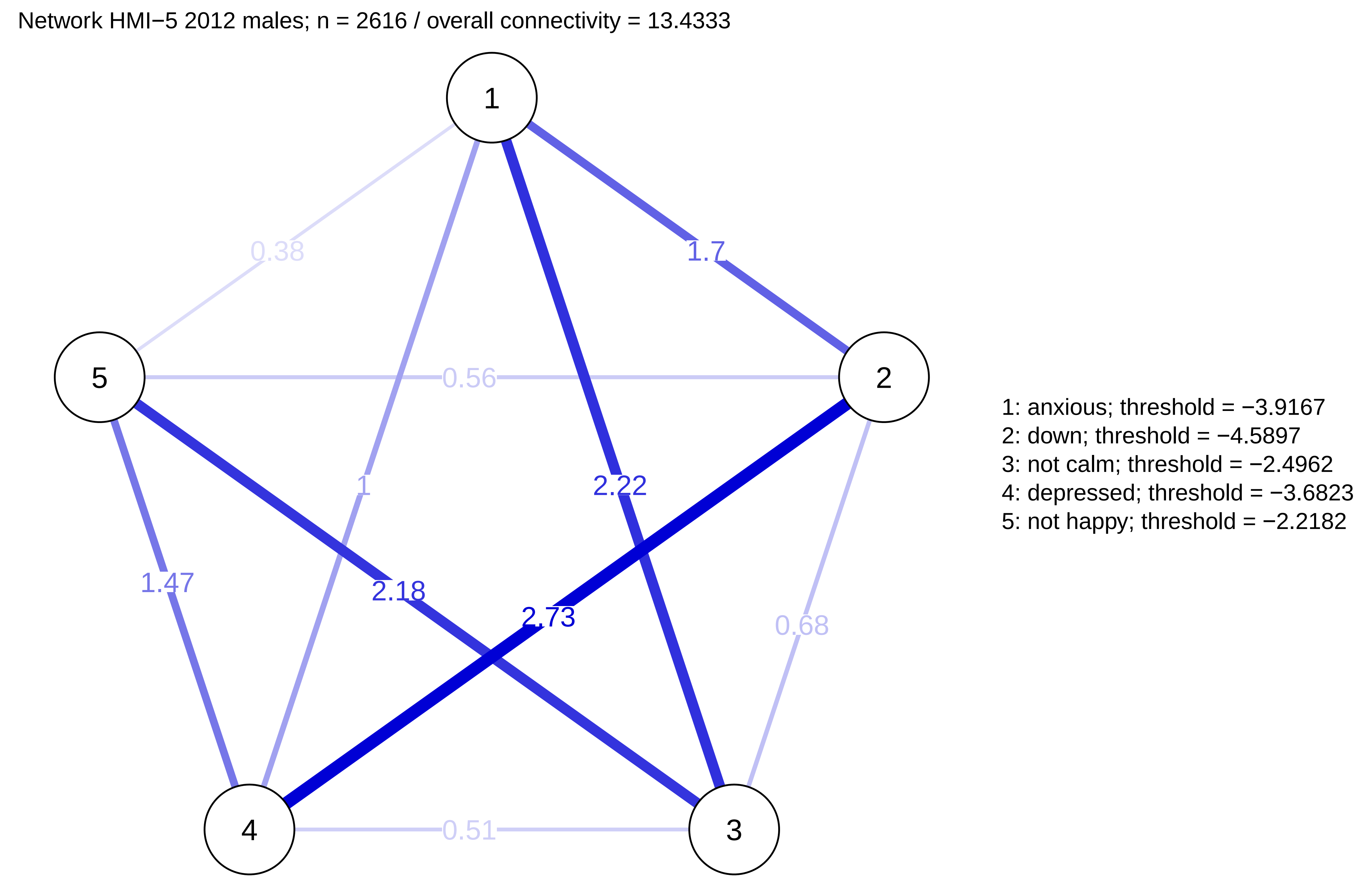
Network HMI−5 2012 males; n = 2616 / overall connectivity = 13.4333
1
2
3
4
5
0.38
1.7
0.56
1
2.22
1.47
2.18
2.73
0.68
0.51
1: anxious; threshold = −3.9167
2: down; threshold = −4.5897
3: not calm; threshold = −2.4962
4: depressed; threshold = −3.6823
5: not happy; threshold = −2.2182

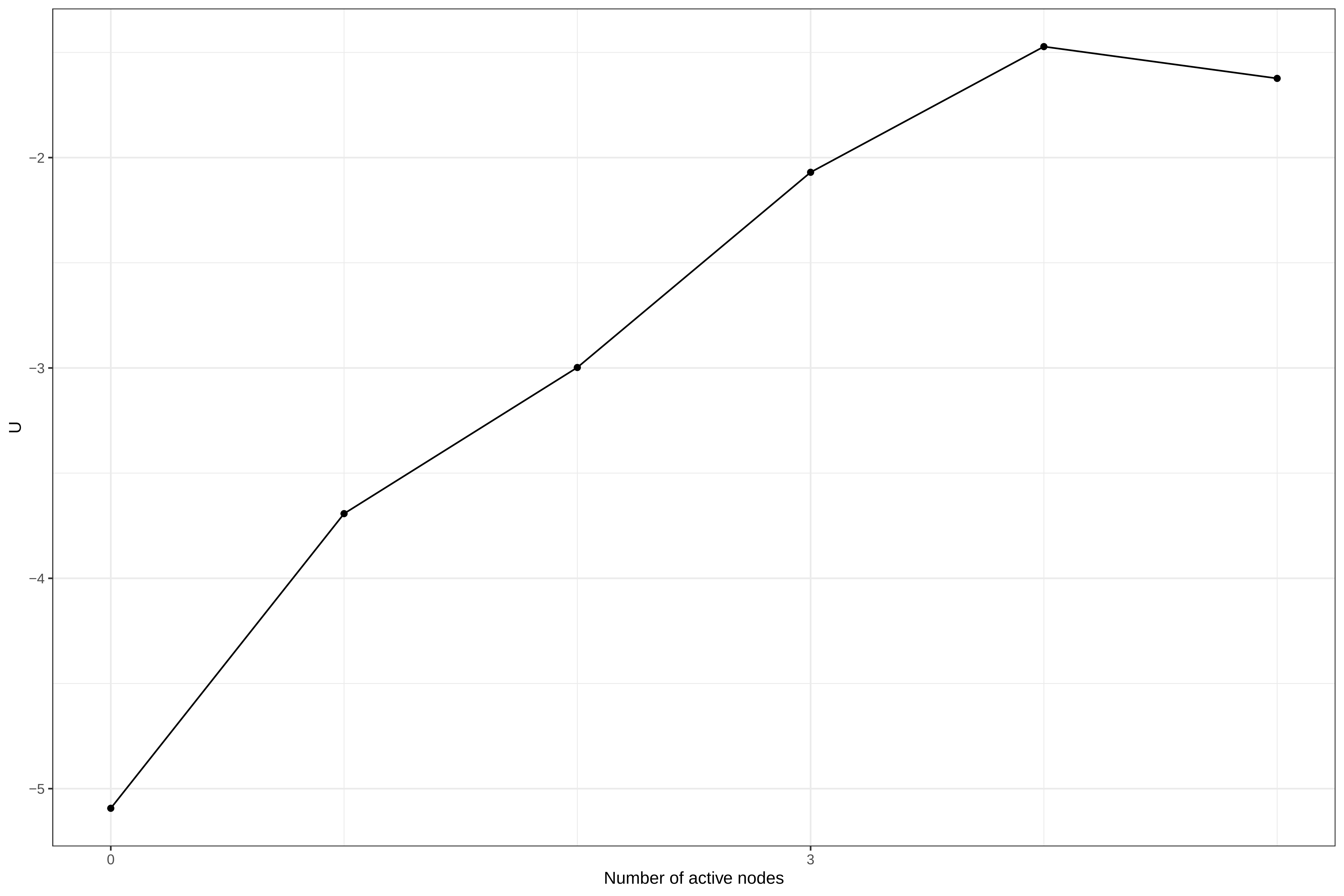

−2
−3
−4
−5
U
0
3
Number of active nodes

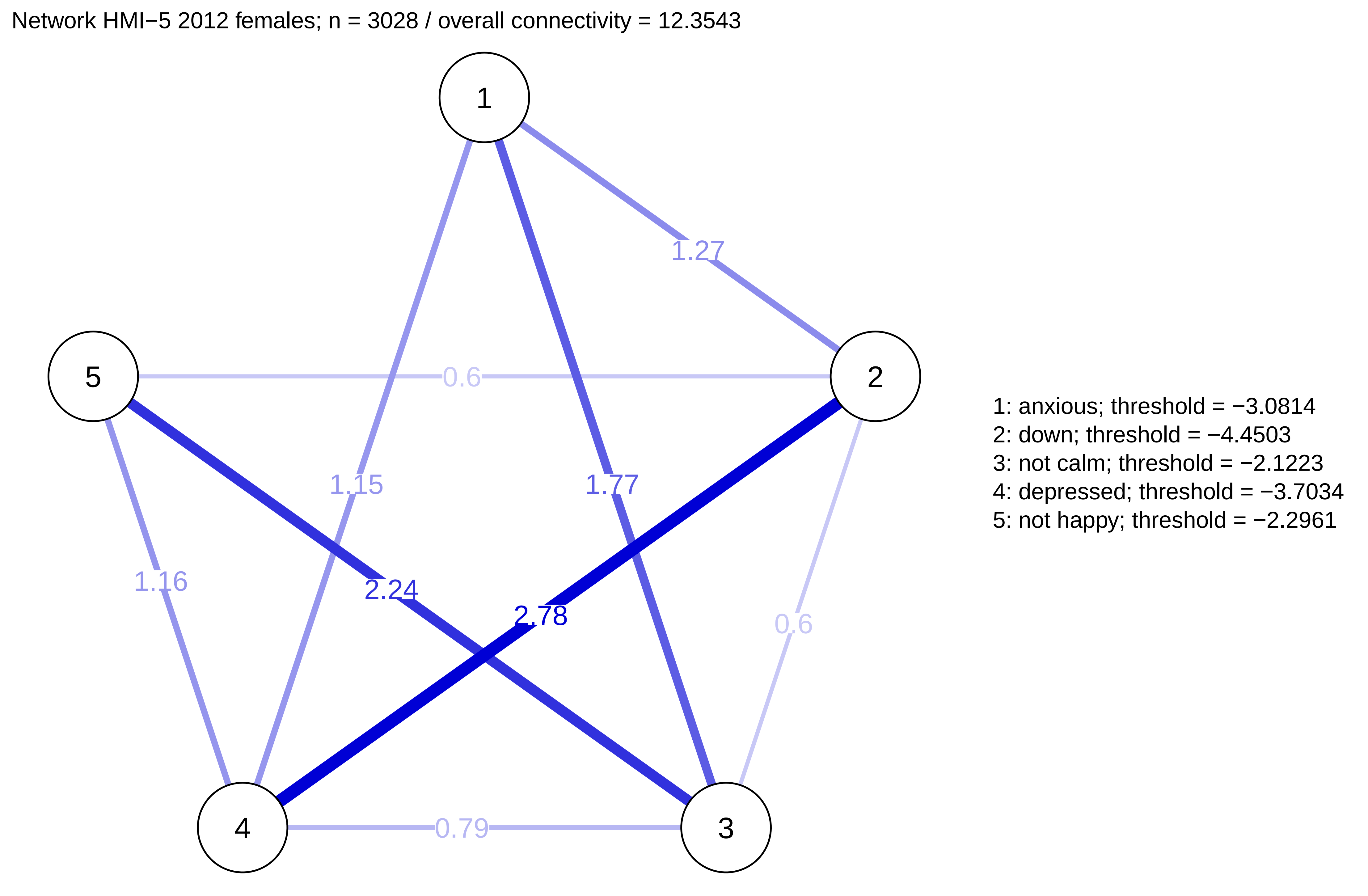
Network HMI−5 2012 females; n = 3028 / overall connectivity = 12.3543
1
2
3
4
5
1.27
0.6
1.15
1.77
1.16
2.24
2.78
0.6
0.79
1: anxious; threshold = −3.0814
2: down; threshold = −4.4503
3: not calm; threshold = −2.1223
4: depressed; threshold = −3.7034
5: not happy; threshold = −2.2961

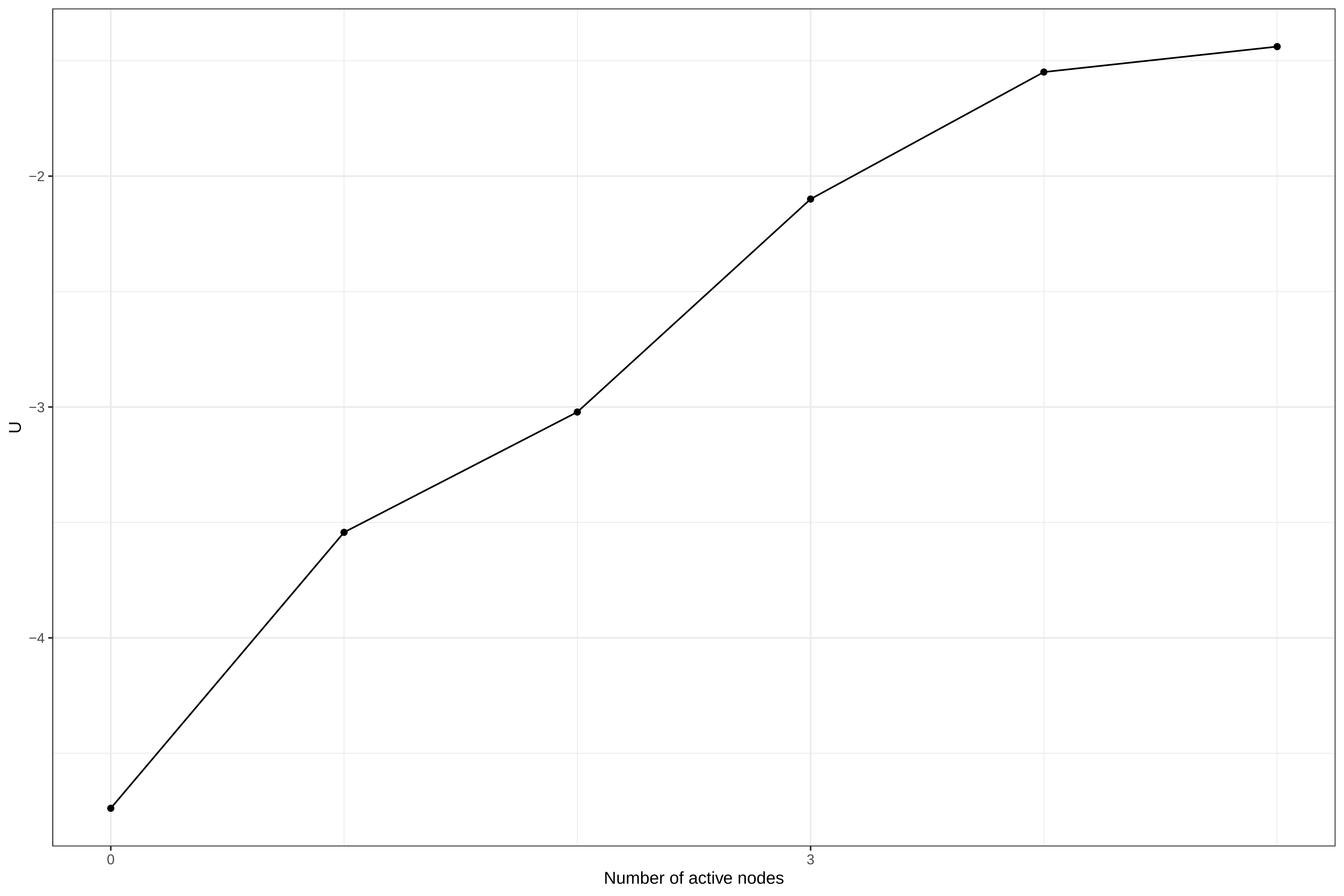

−2
−3
−4
U
0
3
Number of active nodes

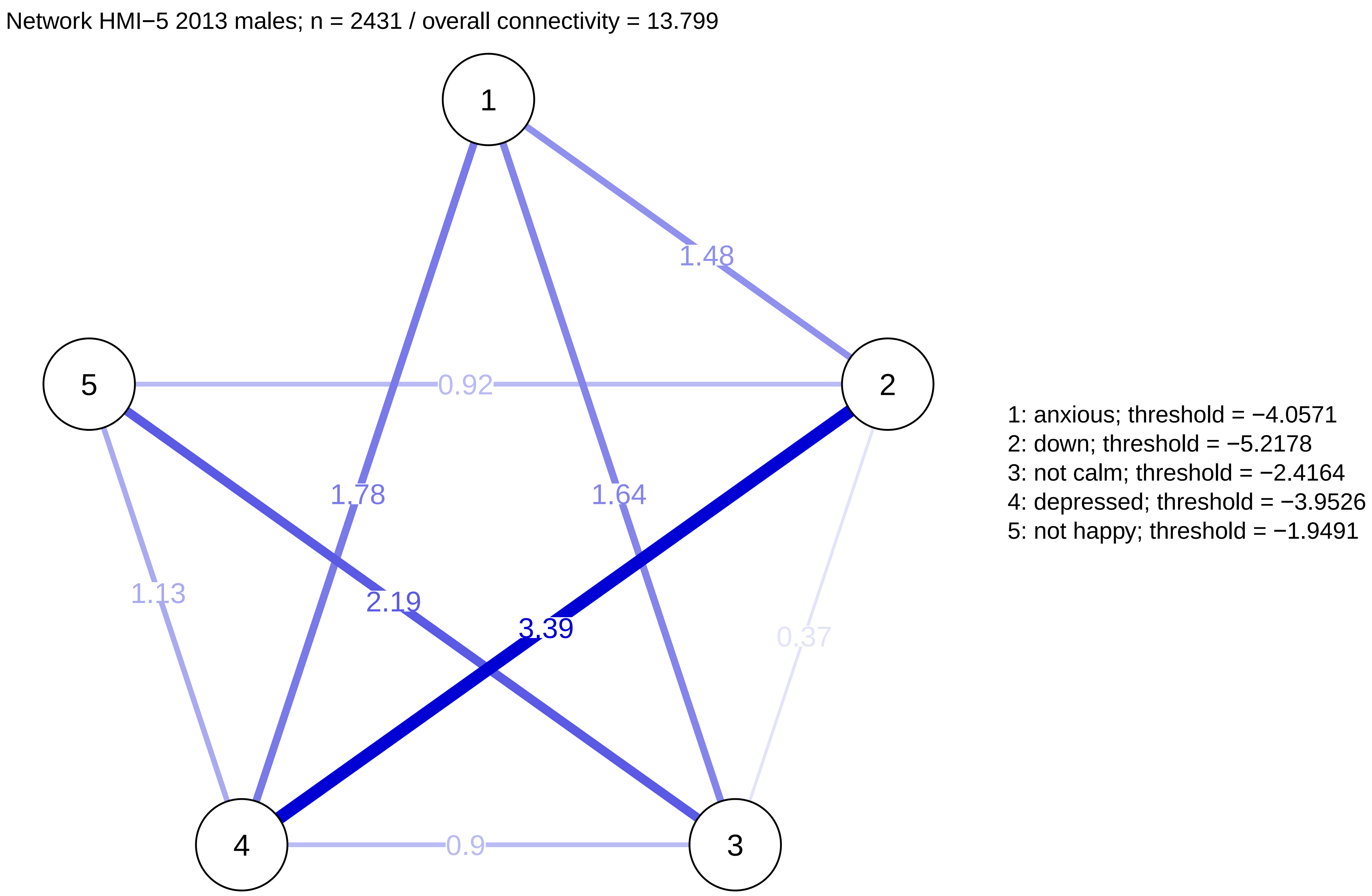
Network HMI−5 2013 males; n = 2431 / overall connectivity = 13.799
1
2
3
4
5
1.48
0.92
1.78
1.64
1.13
2.19
3.39
0.37
0.9
1: anxious; threshold = −4.0571
2: down; threshold = −5.2178
3: not calm; threshold = −2.4164
4: depressed; threshold = −3.9526
5: not happy; threshold = −1.9491

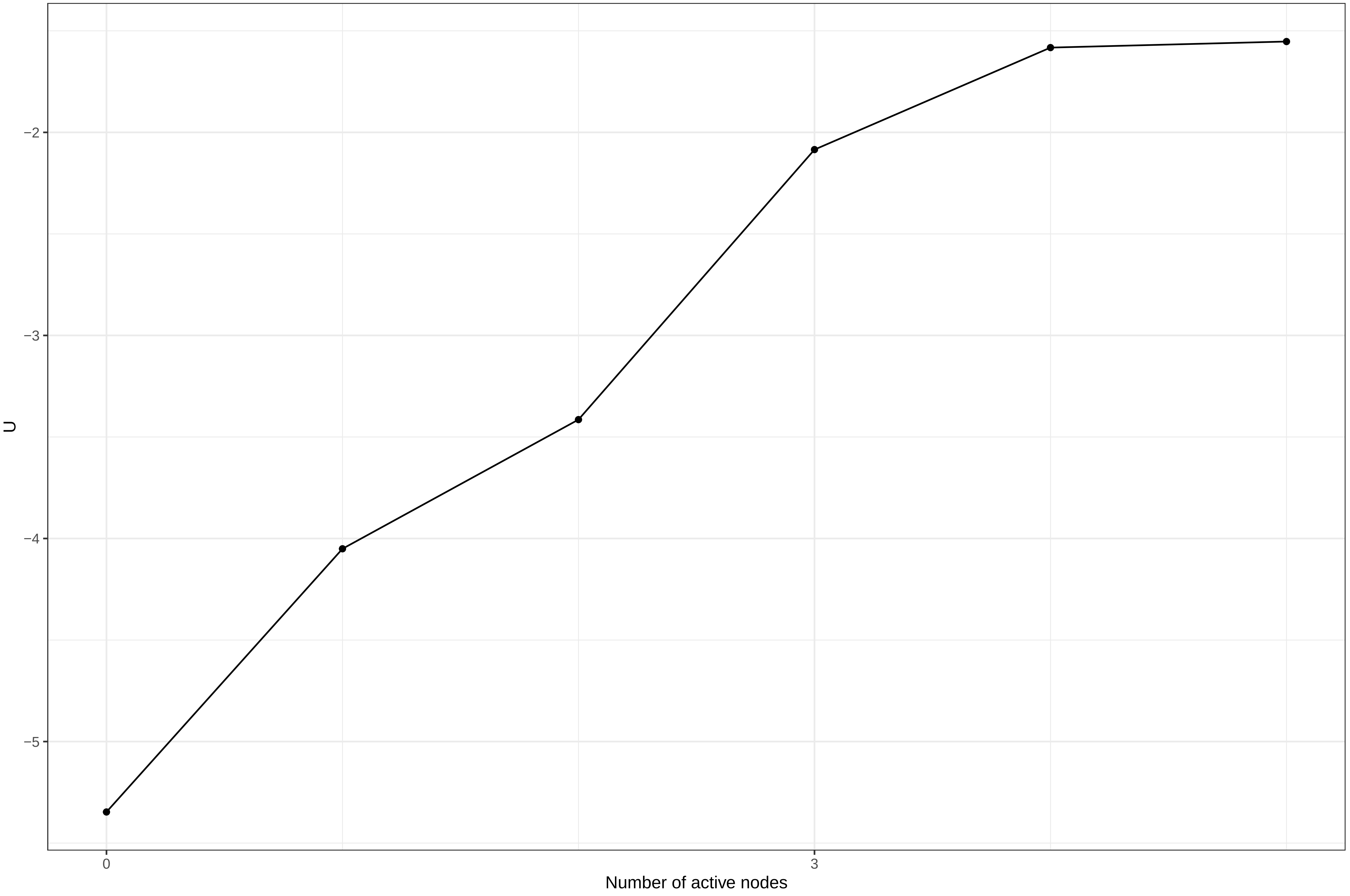

U
−2
−3
−4
−5
0
3
Number of active nodes

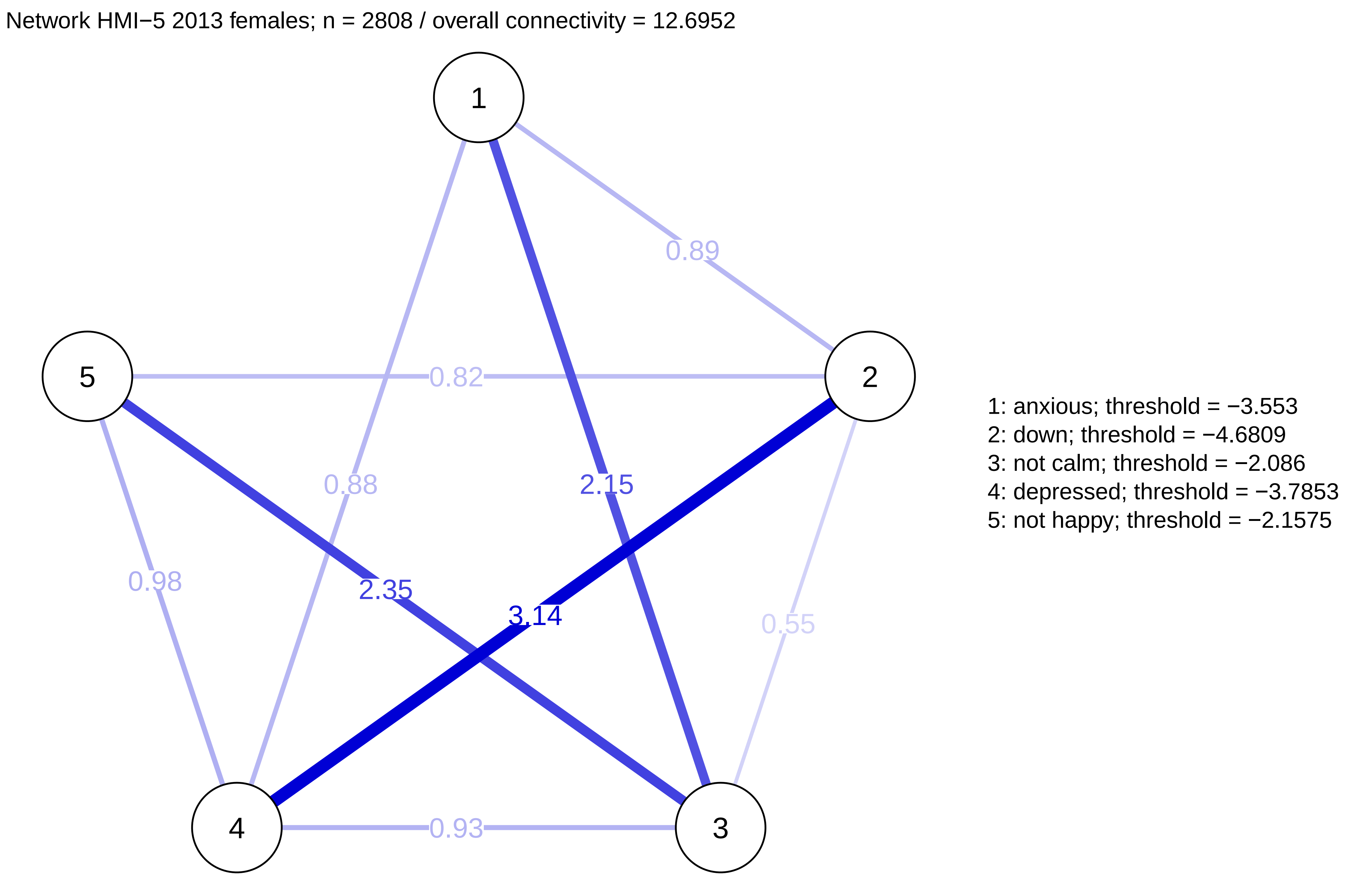
Network HMI−5 2013 females; n = 2808 / overall connectivity = 12.6952
1
2
3
4
5
0.89
0.82
0.88
2.15
0.98
2.35
3.14
0.55
0.93
1: anxious; threshold = −3.553
2: down; threshold = −4.6809
3: not calm; threshold = −2.086
4: depressed; threshold = −3.7853
5: not happy; threshold = −2.1575

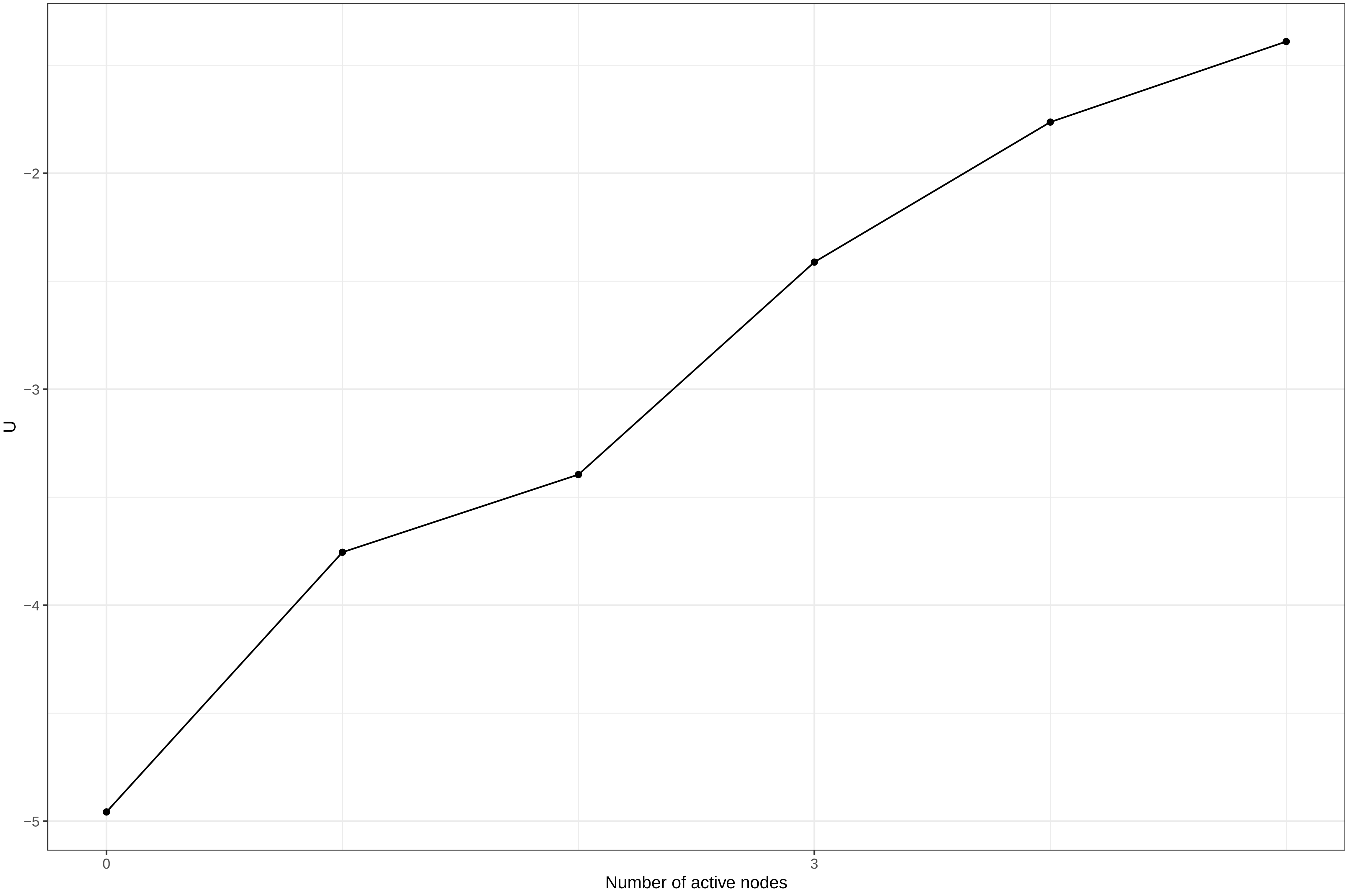

−2
−3
−4
−5
U
0
3
Number of active nodes

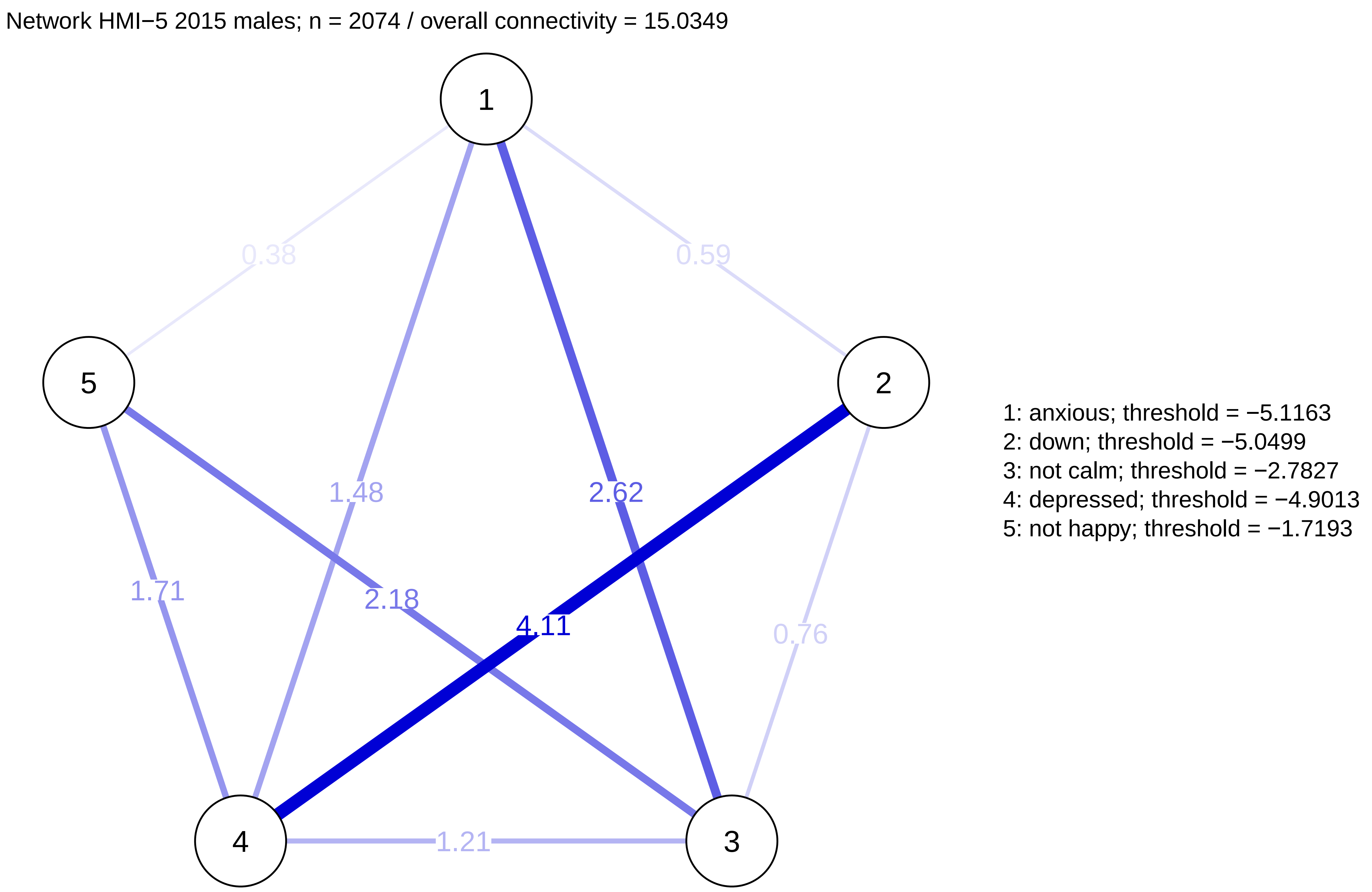
Network HMI−5 2015 males; n = 2074 / overall connectivity = 15.0349
1
2
3
4
5
0.38
0.59
1.48
2.62
1.71
2.18
4.11
0.76
1.21
1: anxious; threshold = −5.1163
2: down; threshold = −5.0499
3: not calm; threshold = −2.7827
4: depressed; threshold = −4.9013
5: not happy; threshold = −1.7193

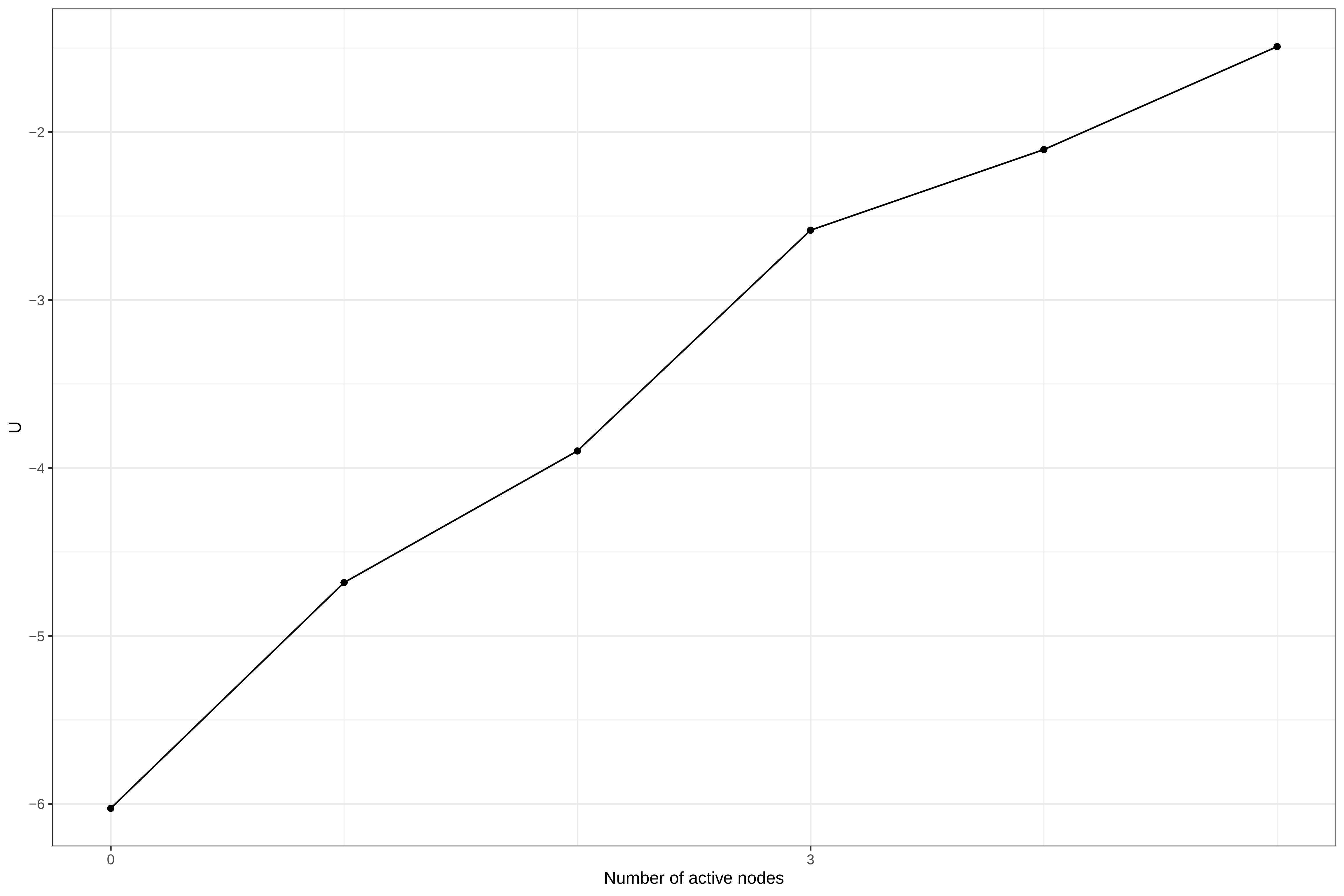

U
−2
−3
−4
−5
−6
0
3
Number of active nodes

Network HMI−5 2015 females; n = 2364 / overall connectivity = 16.4047

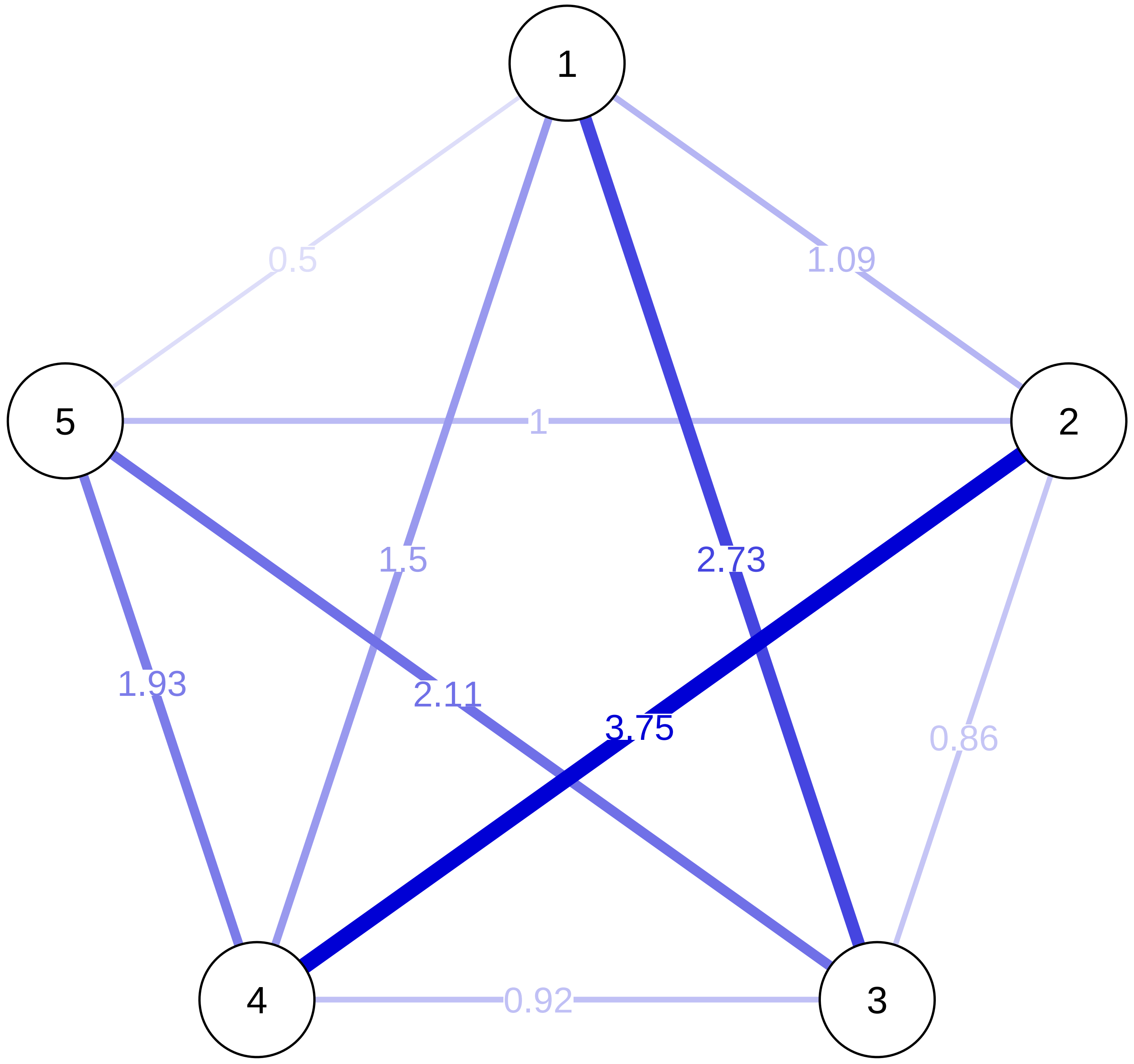

1: anxious; threshold = −4.724
2: down; threshold = −5.9995
3: not calm; threshold = −2.2036
4: depressed; threshold = −4.9839
5: not happy; threshold = −2.0709

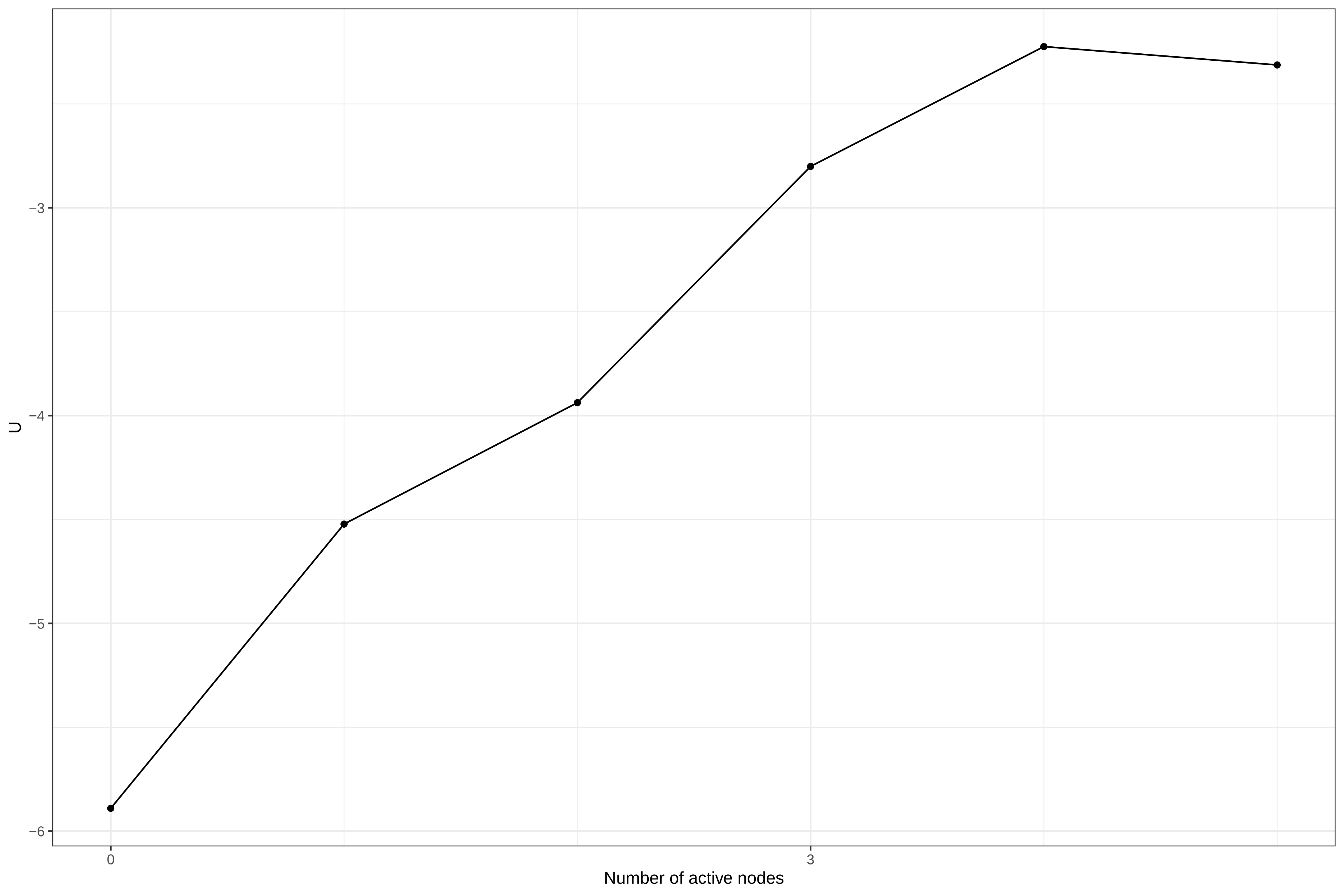
U
−3
−4
−5
−6
0
3
Number of active nodes

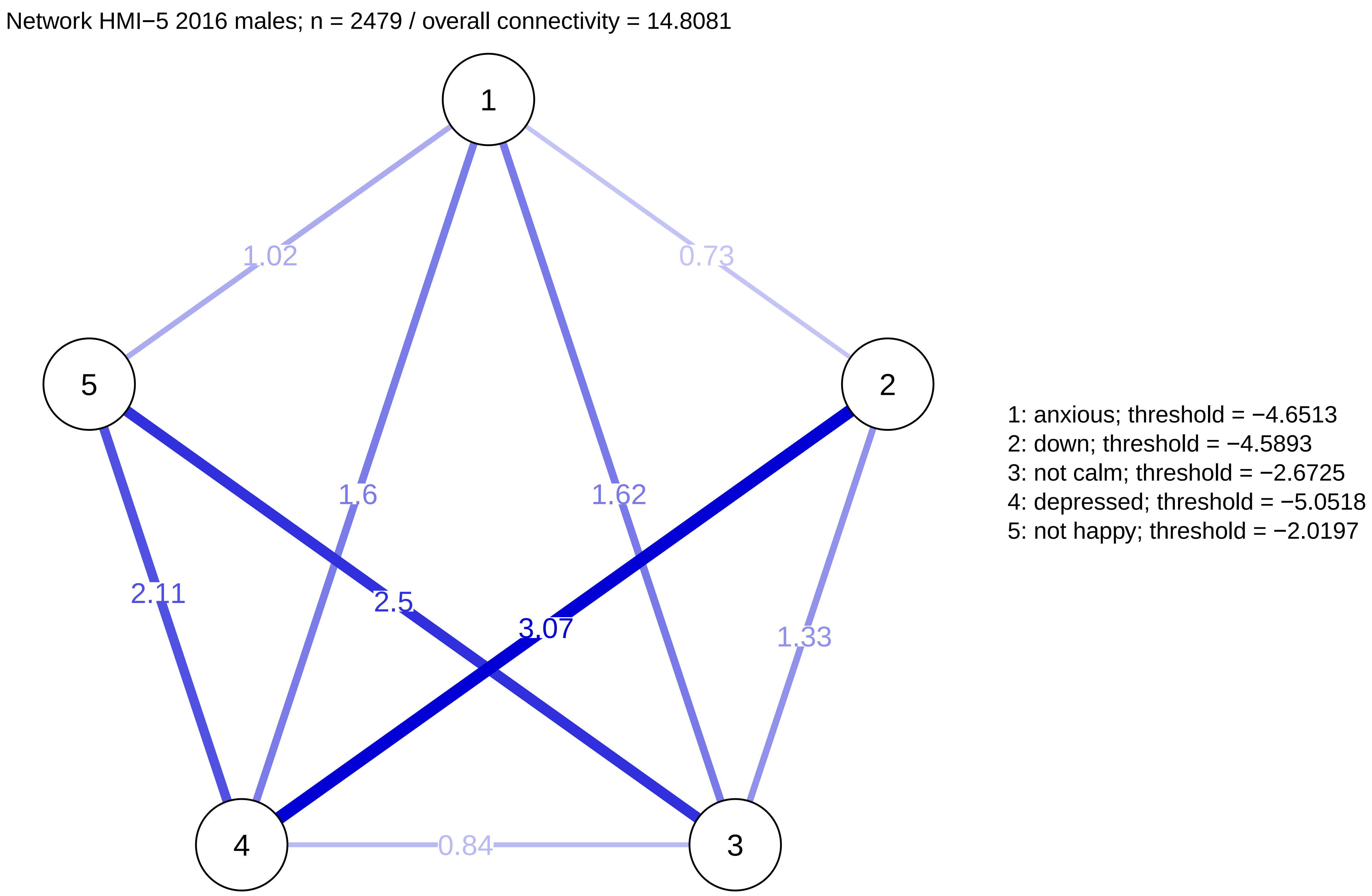
Network HMI−5 2016 males; n = 2479 / overall connectivity = 14.8081
1
2
3
4
5
1.02
0.73
1.6
1.62
2.11
2.5
3.07
1.33
0.84
1: anxious; threshold = −4.6513
2: down; threshold = −4.5893
3: not calm; threshold = −2.6725
4: depressed; threshold = −5.0518
5: not happy; threshold = −2.0197

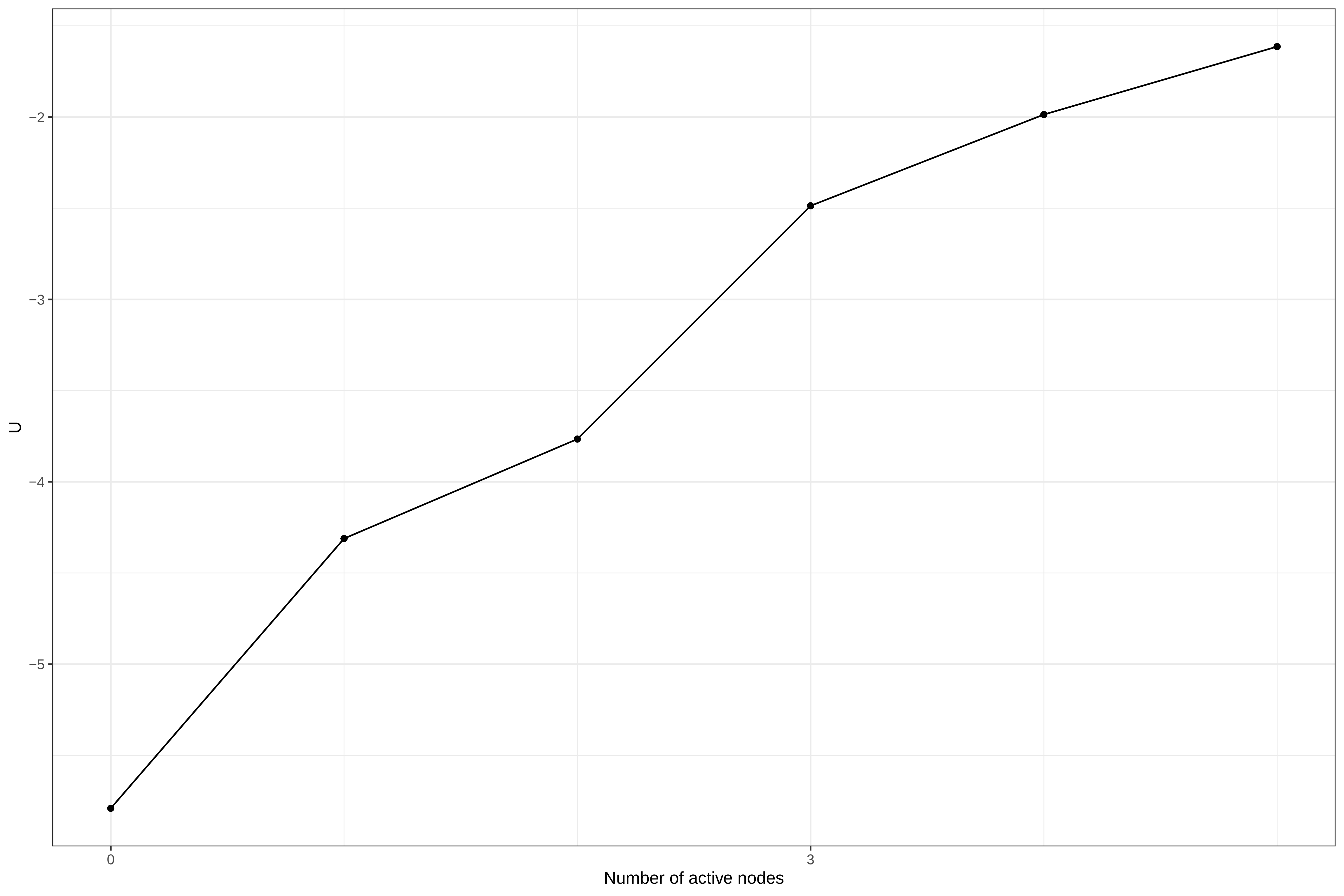

−2
−3
−4
−5
U
0
3
Number of active nodes

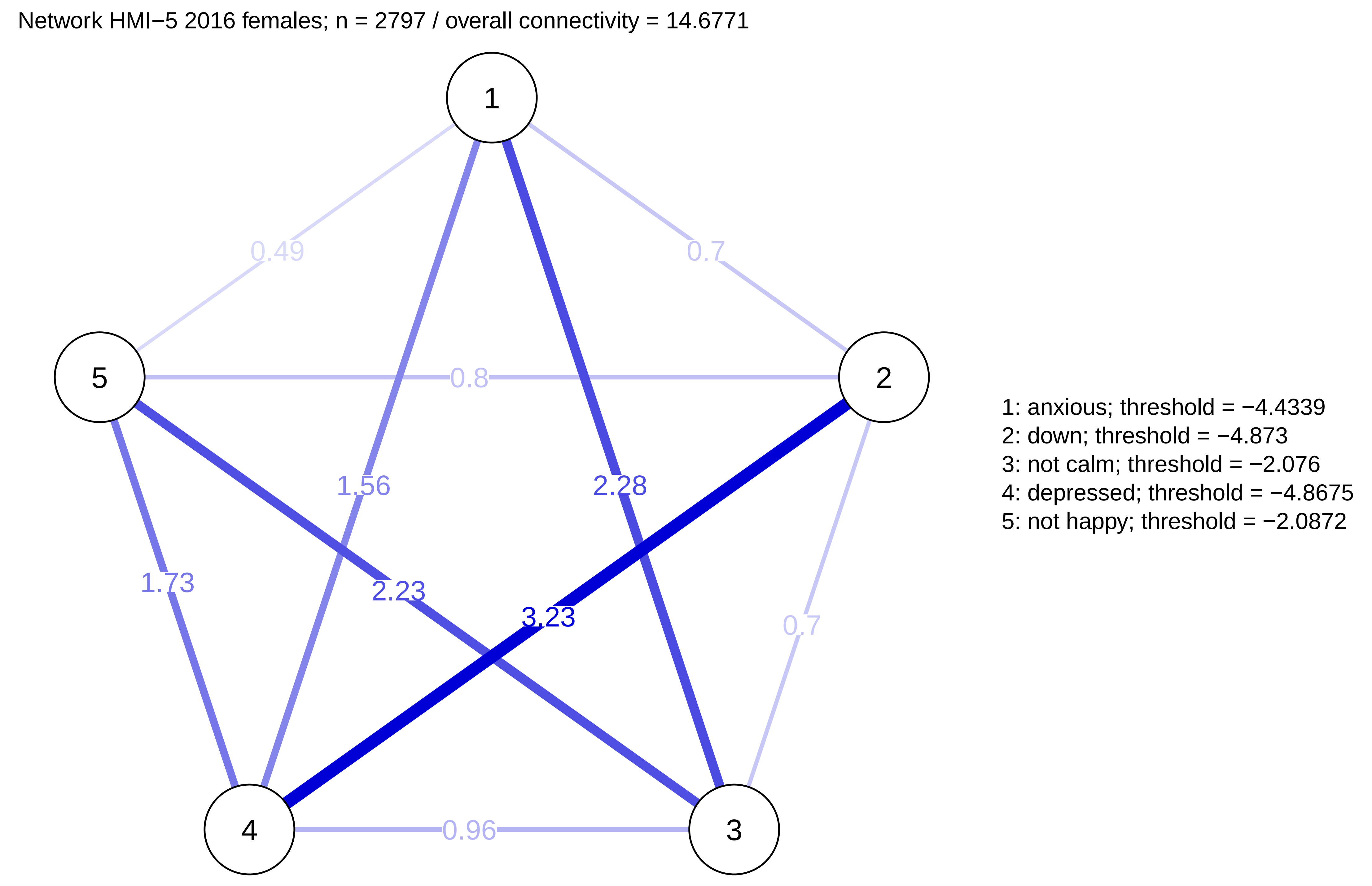

Network HMI−5 2016 females; n = 2797 / overall connectivity = 14.6771
1
2
3
4
5
0.49
0.7
0.8
1.56
2.28
1.73
2.23
3.23
0.7
0.96
1: anxious; threshold = −4.4339
2: down; threshold = −4.873
3: not calm; threshold = −2.076
4: depressed; threshold = −4.8675
5: not happy; threshold = −2.0872

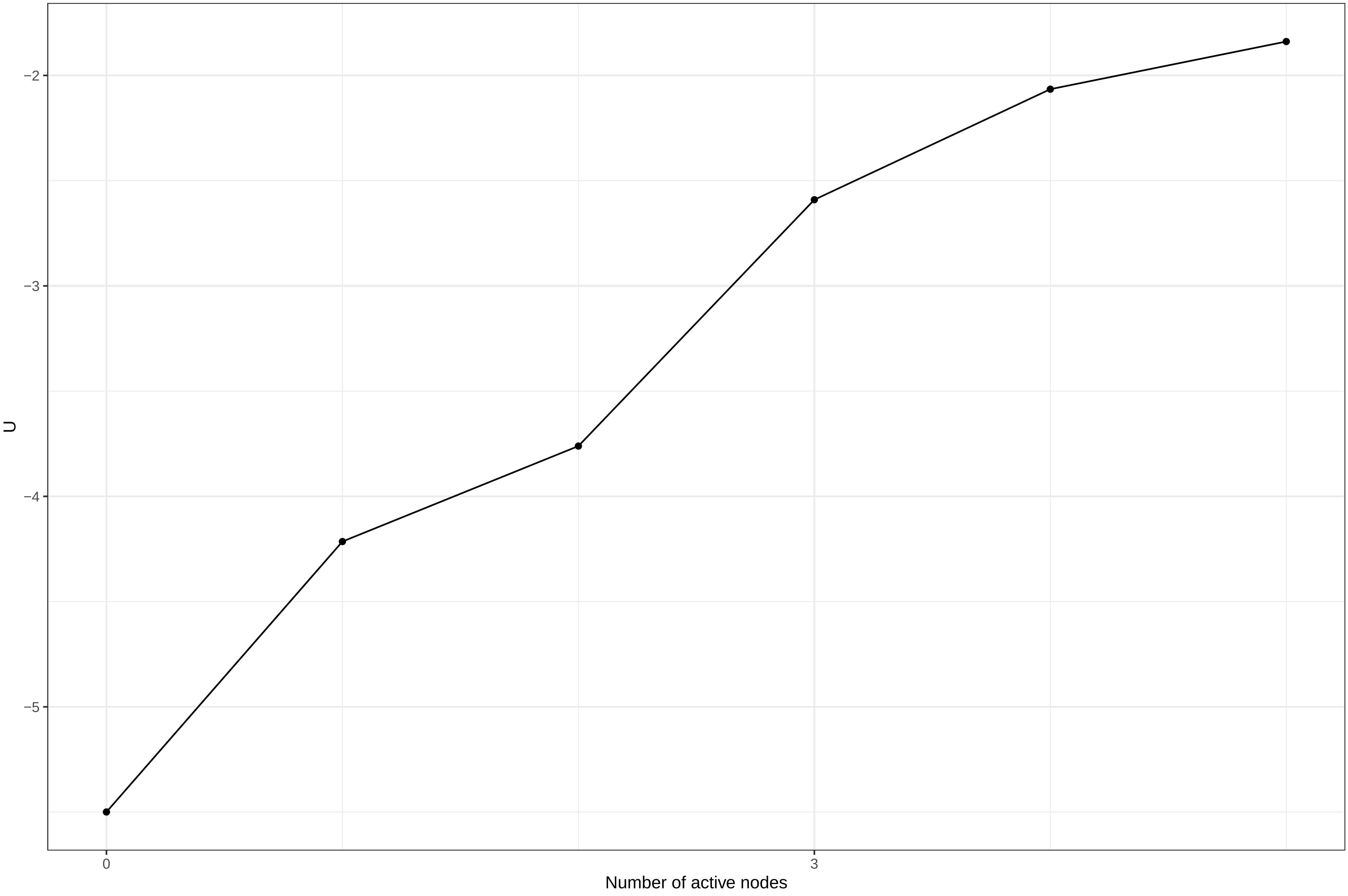
−2
−3
−4
−5
U
0
3
Number of active nodes

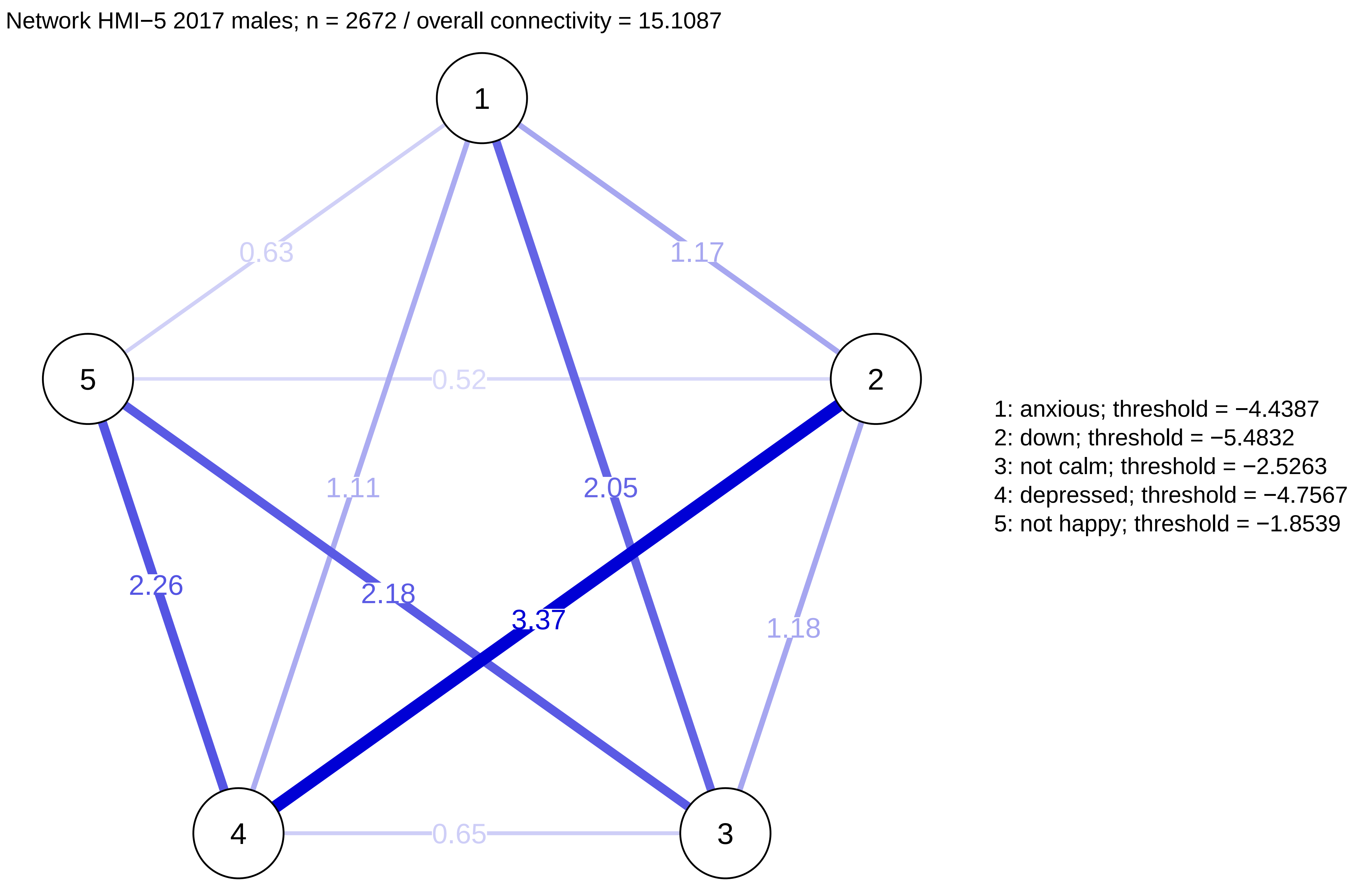
Network HMI−5 2017 males; n = 2672 / overall connectivity = 15.1087
1
2
3
4
5
0.63
1.17
0.52
1.11
2.05
2.26
2.18
3.37
1.18
0.65
1: anxious; threshold = −4.4387
2: down; threshold = −5.4832
3: not calm; threshold = −2.5263
4: depressed; threshold = −4.7567
5: not happy; threshold = −1.8539

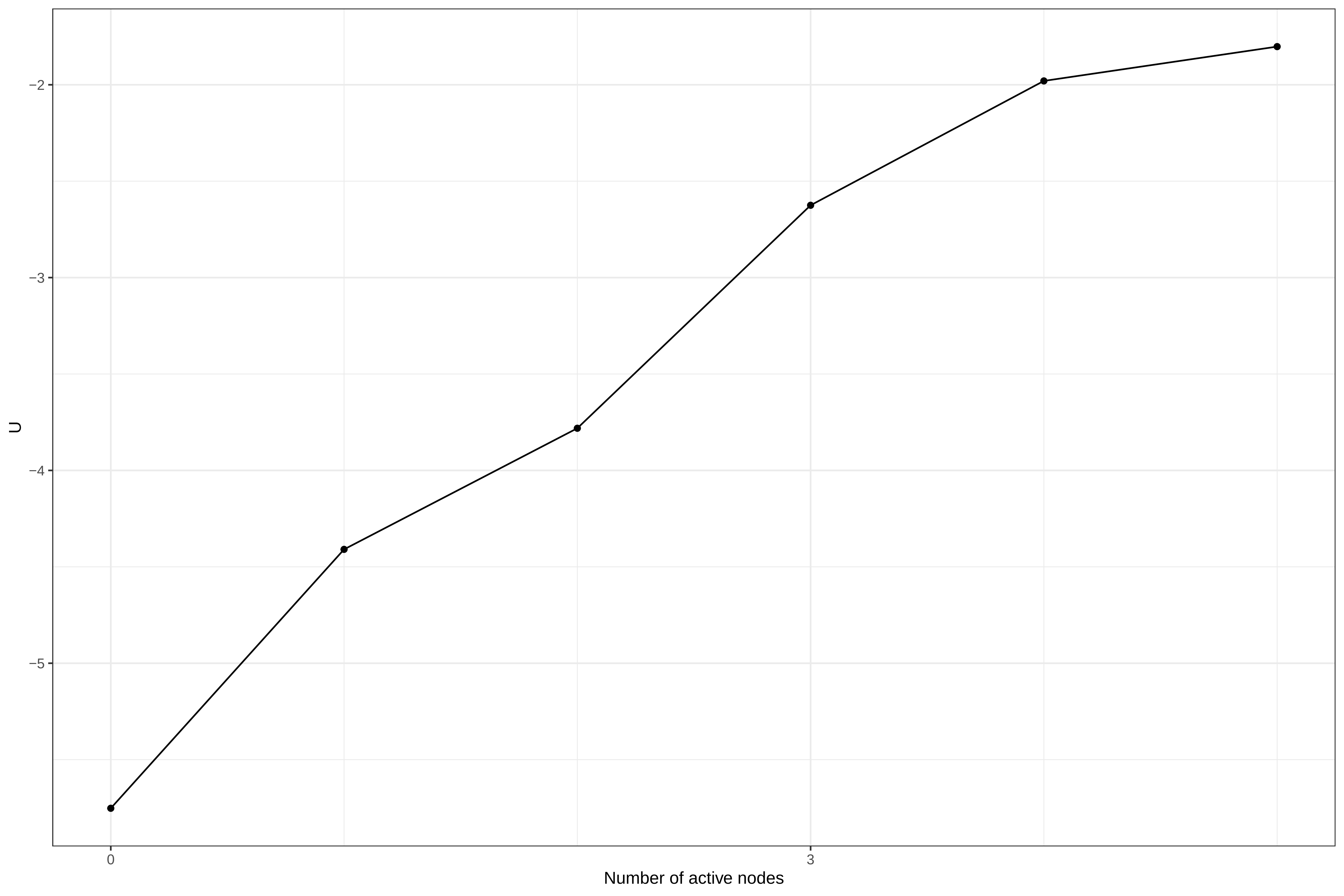

−2
−3
−4
−5
U
0
3
Number of active nodes

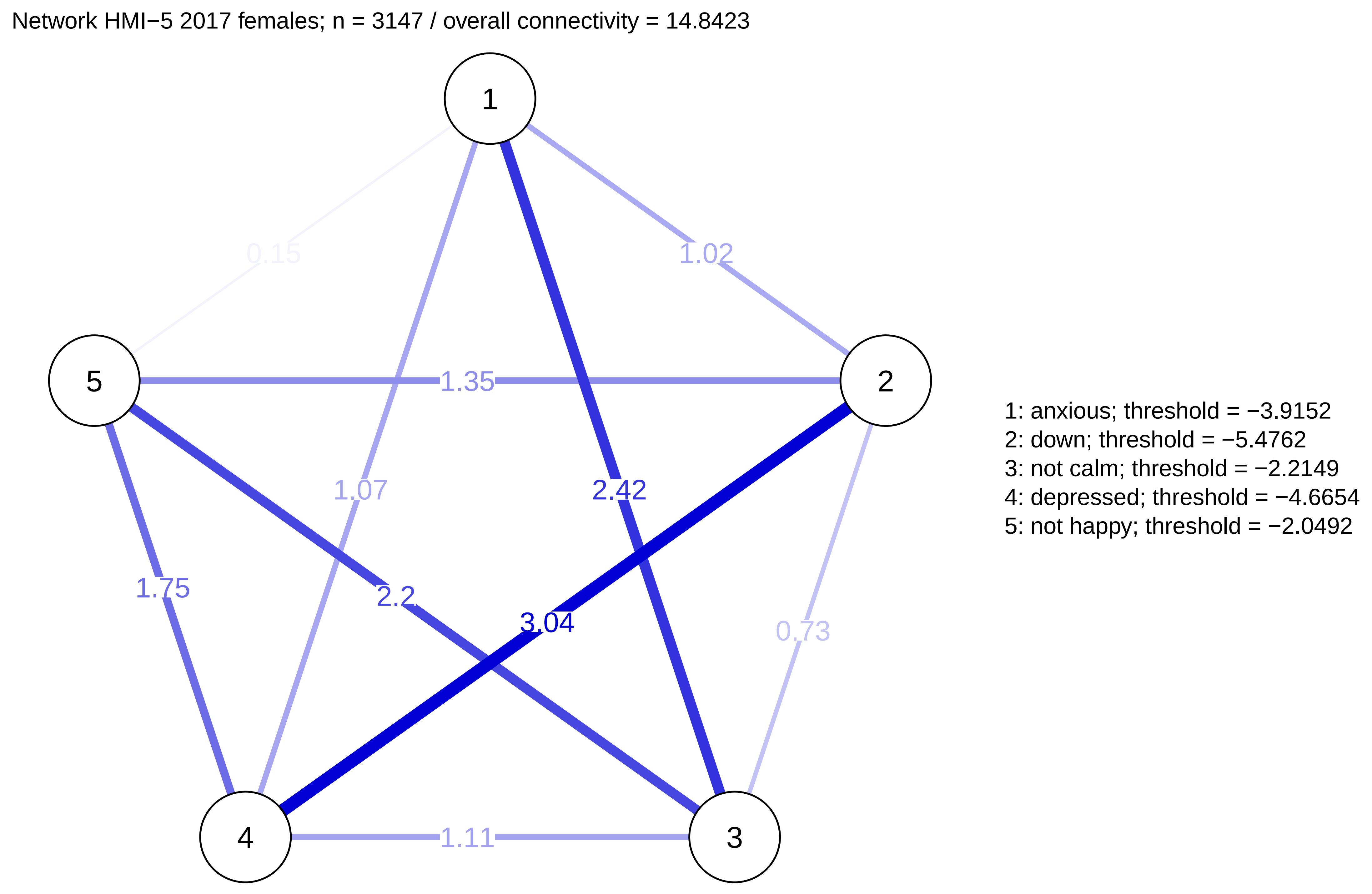
Network HMI−5 2017 females; n = 3147 / overall connectivity = 14.8423
1
2
3
4
5
0.15
1.02
1.35
1.07
2.42
1.75
2.2
3.04
0.73
1.11
1: anxious; threshold = −3.9152
2: down; threshold = −5.4762
3: not calm; threshold = −2.2149
4: depressed; threshold = −4.6654
5: not happy; threshold = −2.0492

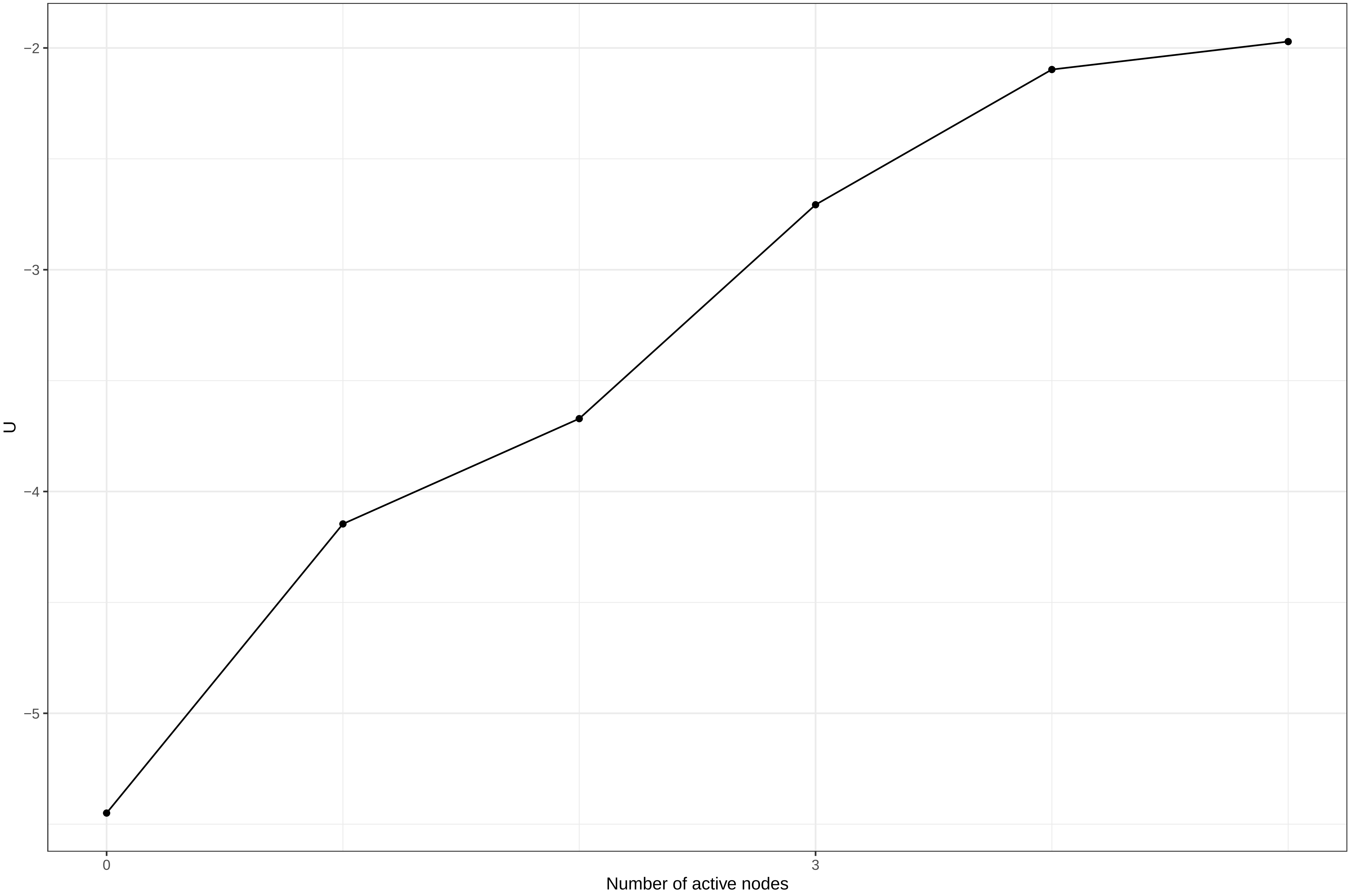

−2
−3
−4
−5
U
0
3
Number of active nodes

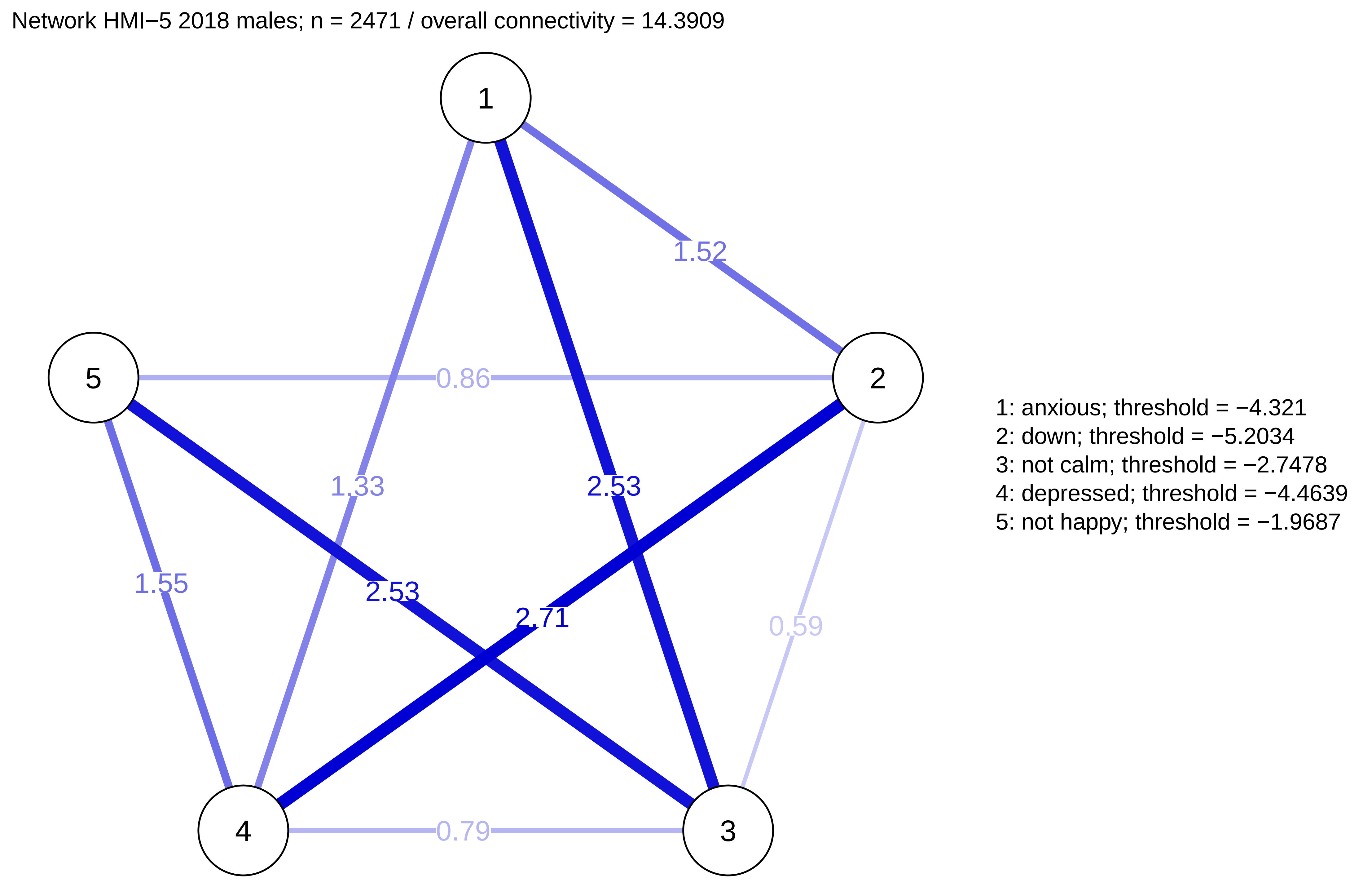
Network HMI−5 2018 males; n = 2471 / overall connectivity = 14.3909
1
2
3
4
5
1.52
0.86
1.33
2.53
1.55
2.53
2.71
0.59
0.79
1: anxious; threshold = −4.321
2: down; threshold = −5.2034
3: not calm; threshold = −2.7478
4: depressed; threshold = −4.4639
5: not happy; threshold = −1.9687

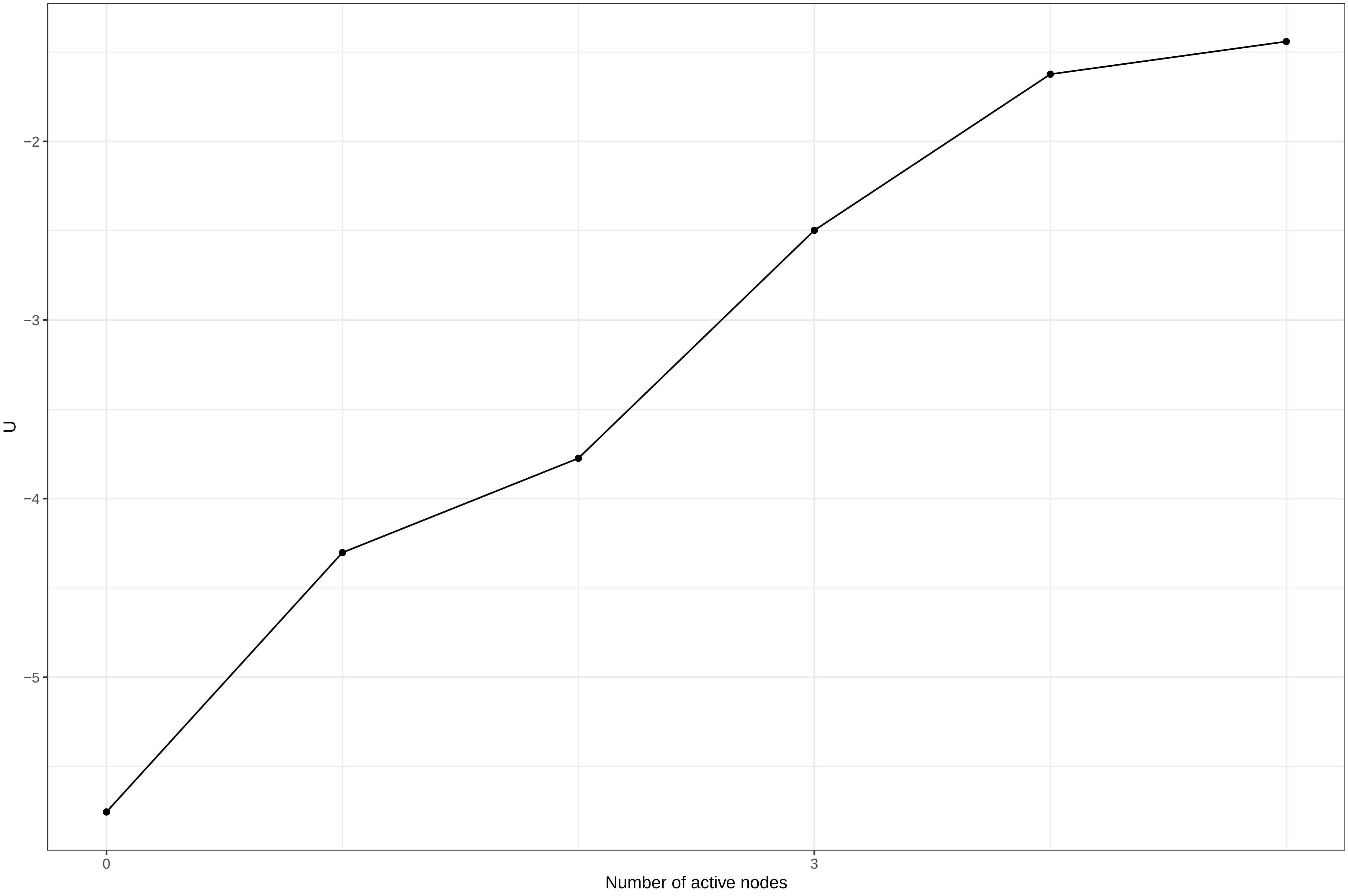
−2
−3
−4
−5
U
0
3
Number of active nodes

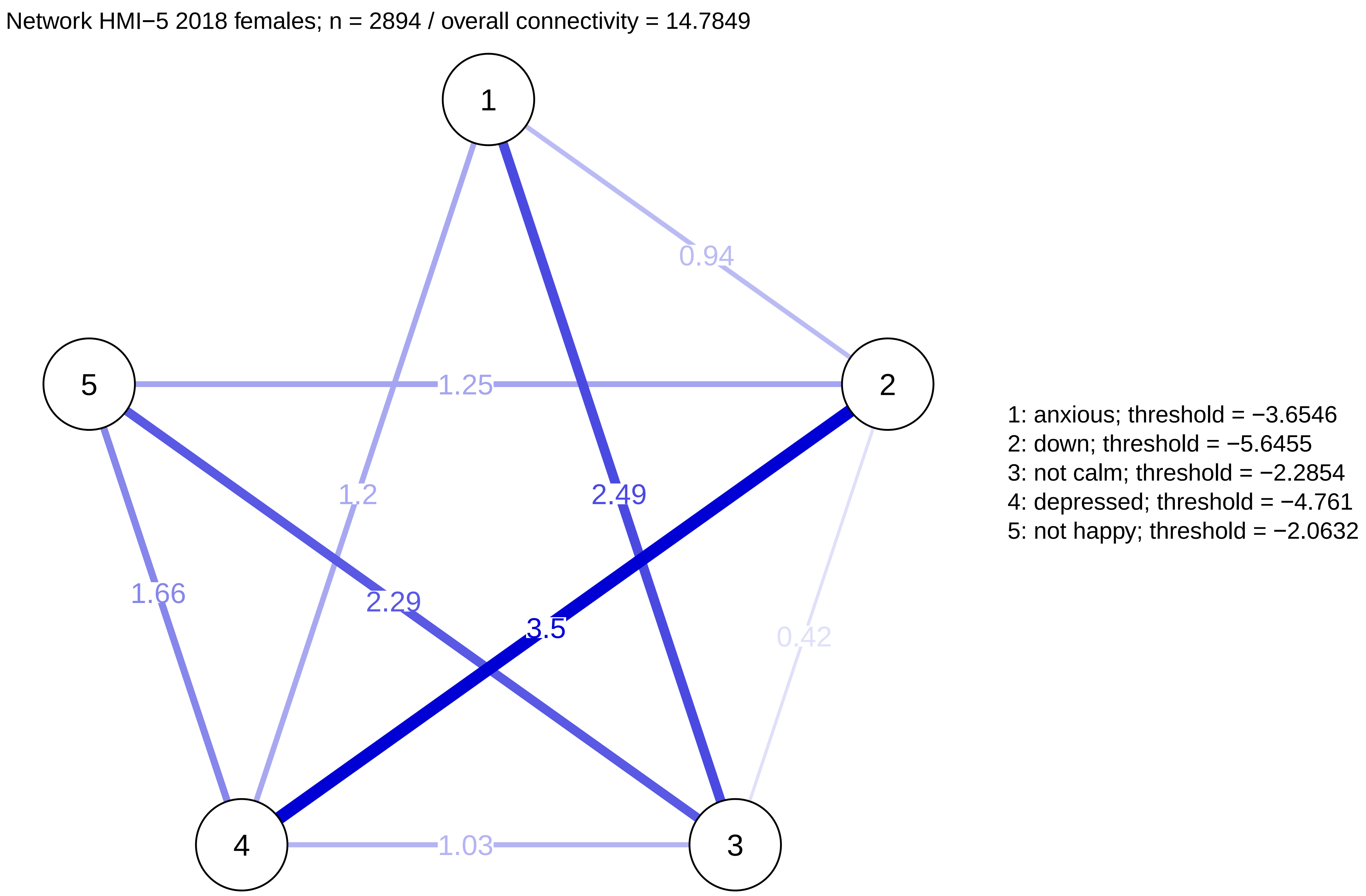
Network HMI−5 2018 females; n = 2894 / overall connectivity = 14.7849
1
2
3
4
5
0.94
1.25
1.2
2.49
1.66
2.29
3.5
0.42
1.03
1: anxious; threshold = −3.6546
2: down; threshold = −5.6455
3: not calm; threshold = −2.2854
4: depressed; threshold = −4.761
5: not happy; threshold = −2.0632

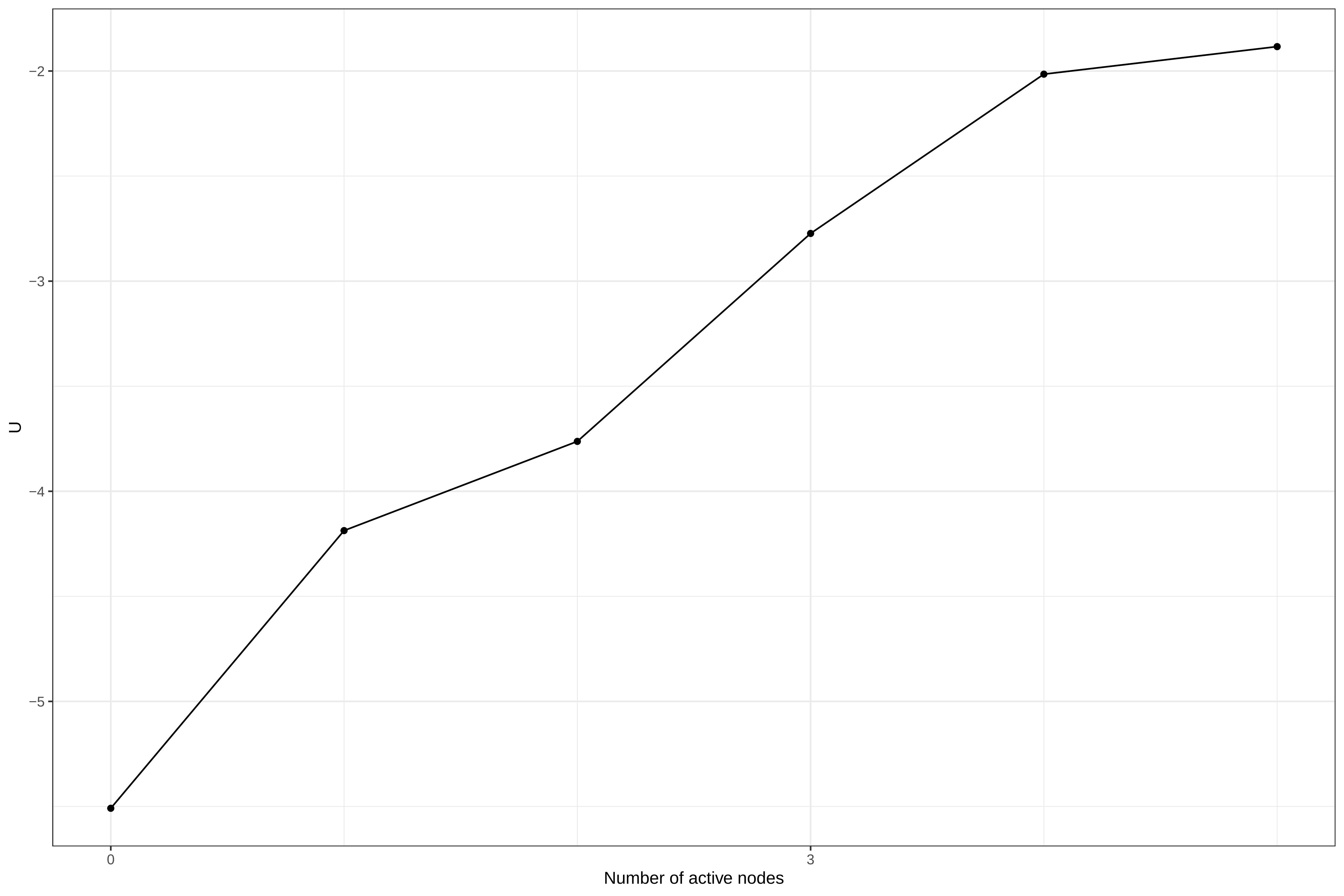

U
−2
−3
−4
−5
0
3
Number of active nodes

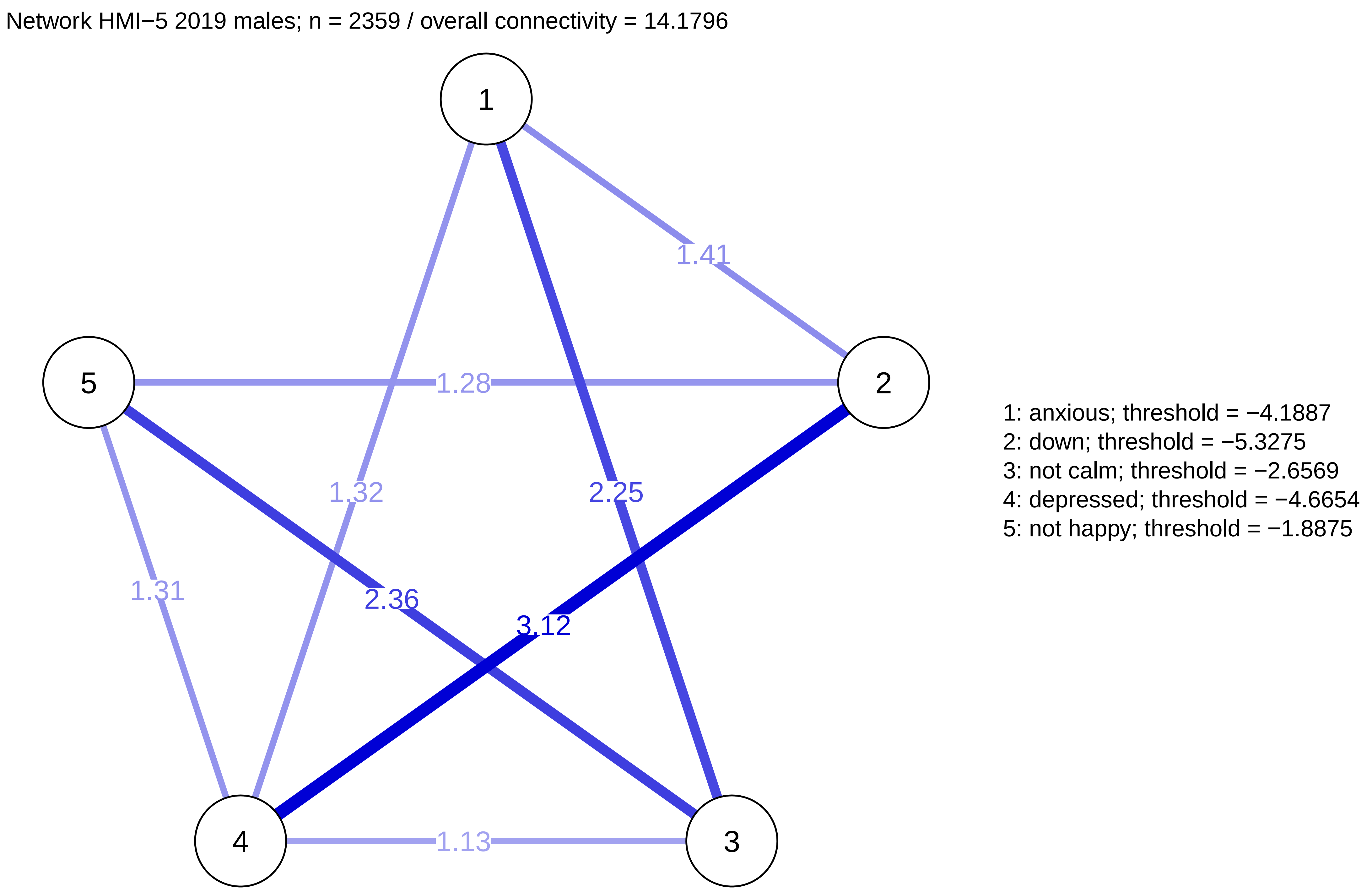
Network HMI−5 2019 males; n = 2359 / overall connectivity = 14.1796
1
2
3
4
5
1.41
1.28
1.32
2.25
1.31
2.36
3.12
1.13
1: anxious; threshold = −4.1887
2: down; threshold = −5.3275
3: not calm; threshold = −2.6569
4: depressed; threshold = −4.6654
5: not happy; threshold = −1.8875

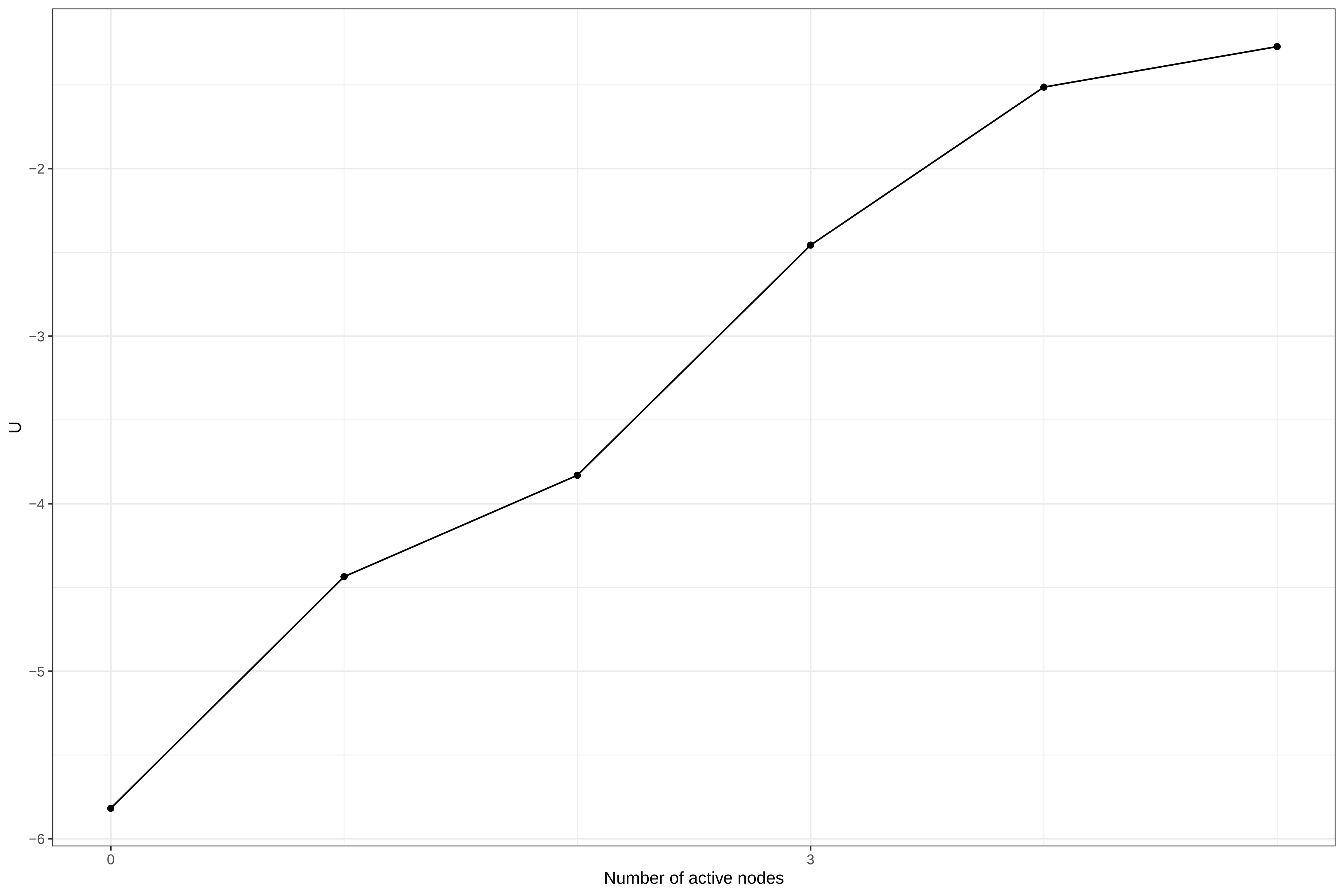

U
−2
−3
−4
−5
−6
0
3
Number of active nodes

Network HMI−5 2019 females; n = 2698 / overall connectivity = 15.408

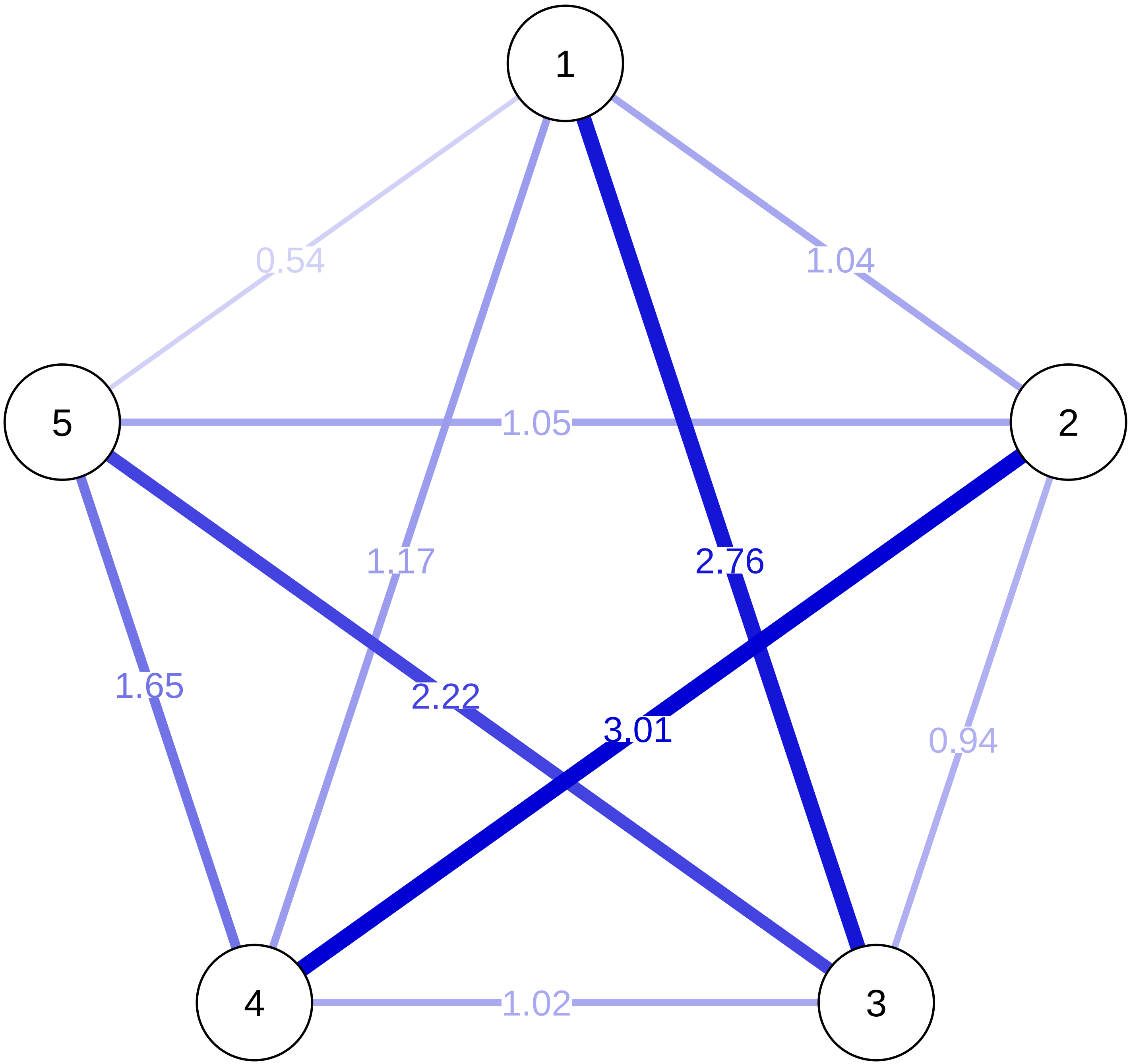

1: anxious; threshold = −4.4077
2: down; threshold = −5.5793
3: not calm; threshold = −2.2378
4: depressed; threshold = −4.6851
5: not happy; threshold = −2.024

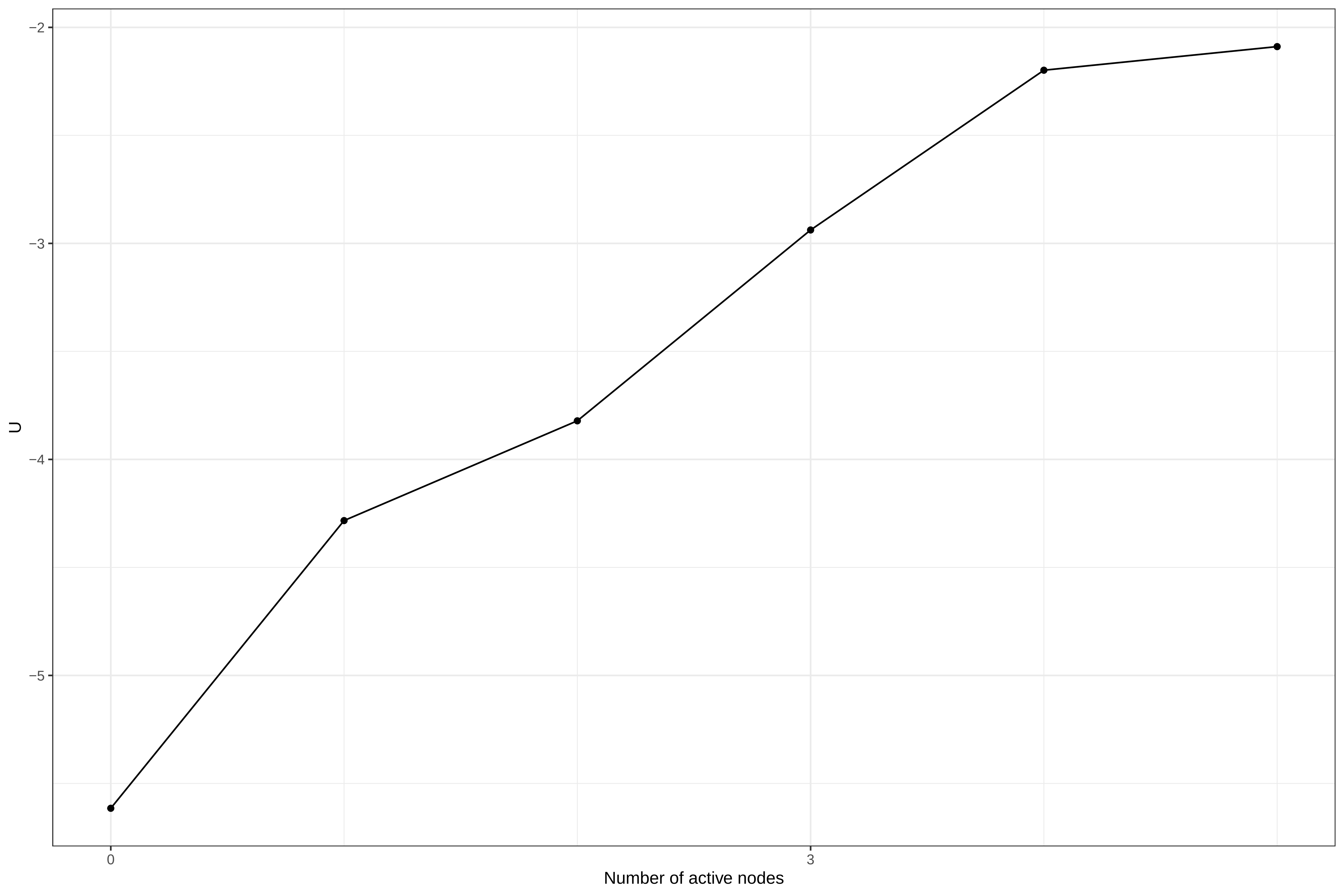

−2
−3
−4
−5
U
0
3
Number of active nodes

Network HMI−5 2020 males; n = 2592 / overall connectivity = 15.7829

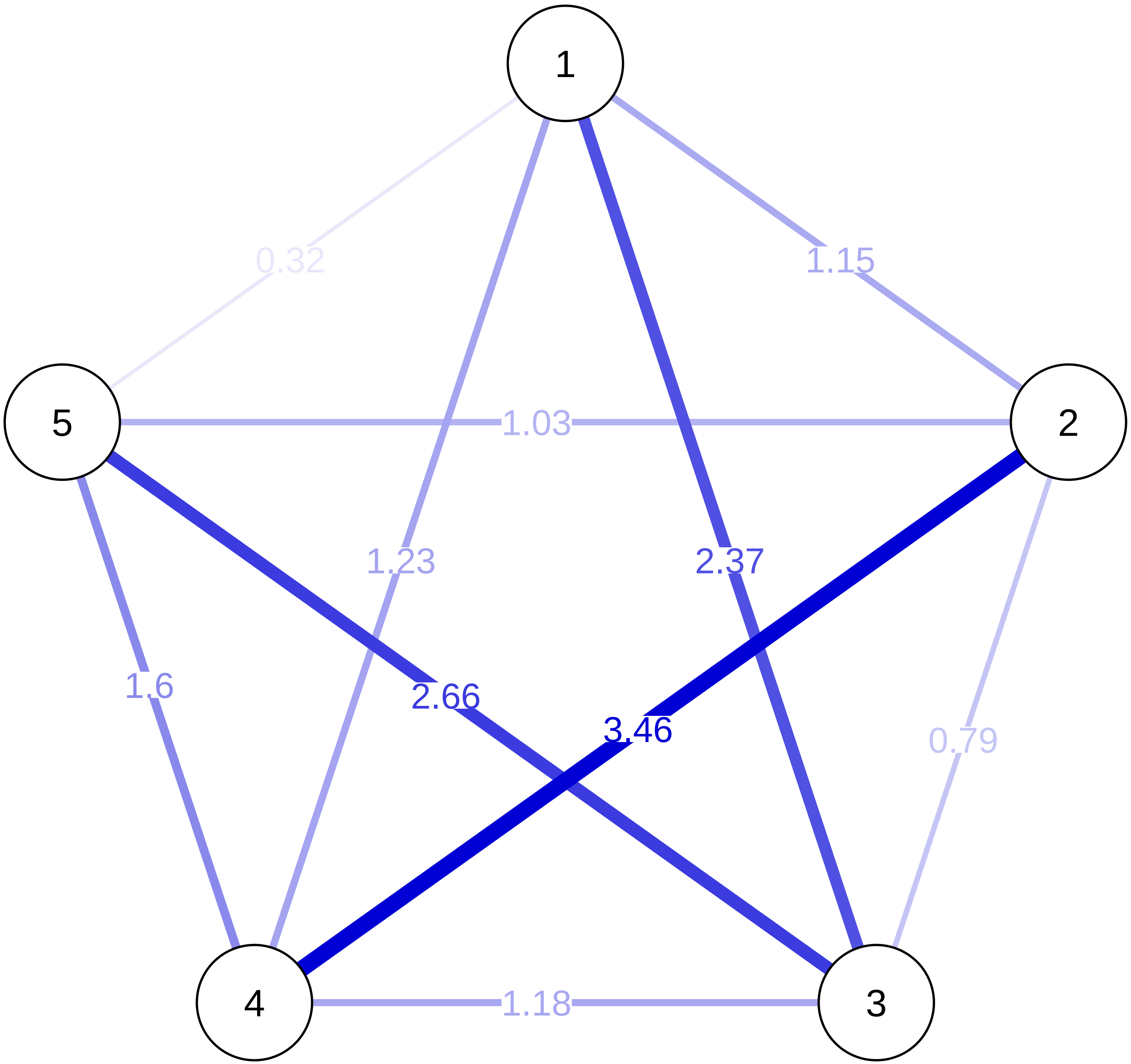

1: anxious; threshold = −4.4977
2: down; threshold = −5.5194
3: not calm; threshold = −3.0202
4: depressed; threshold = −4.6646
5: not happy; threshold = −1.8329

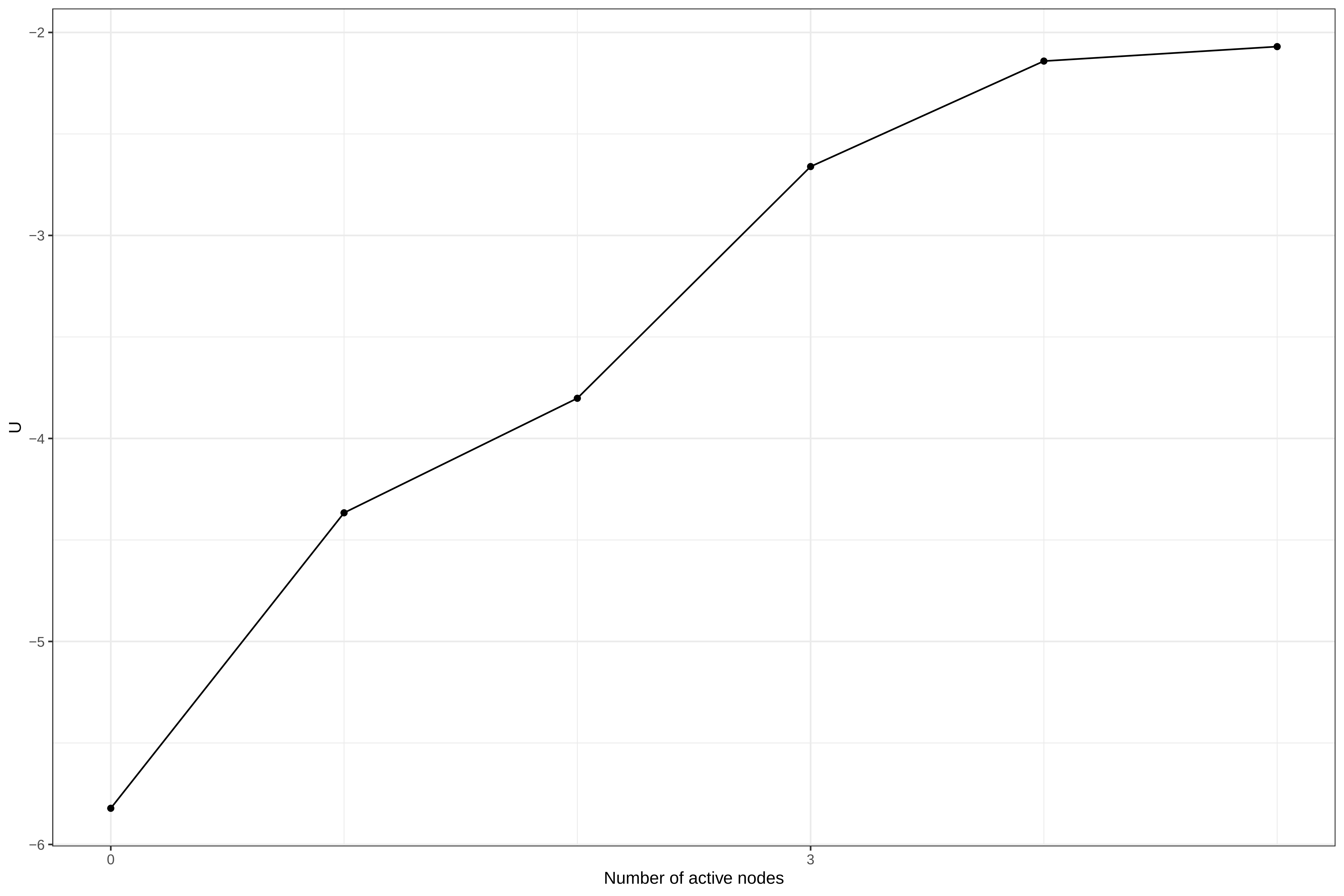

U
−2
−3
−4
−5
−6
0
3
Number of active nodes

Network HMI−5 2020 females; n = 3023 / overall connectivity = 15.1946

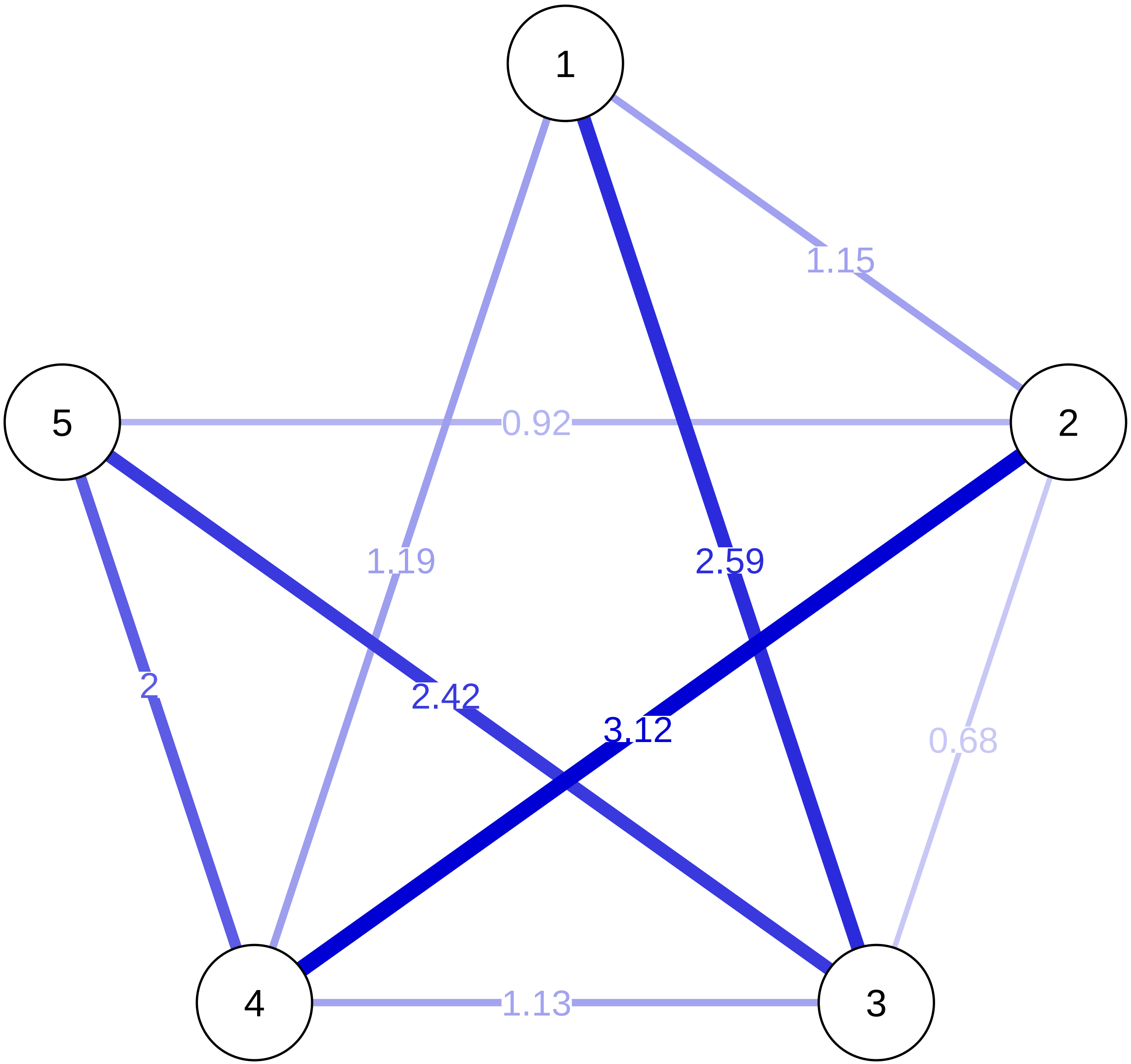

1: anxious; threshold = −3.9132
2: down; threshold = −5.3874
3: not calm; threshold = −2.5739
4: depressed; threshold = −4.8821
5: not happy; threshold = −1.8795

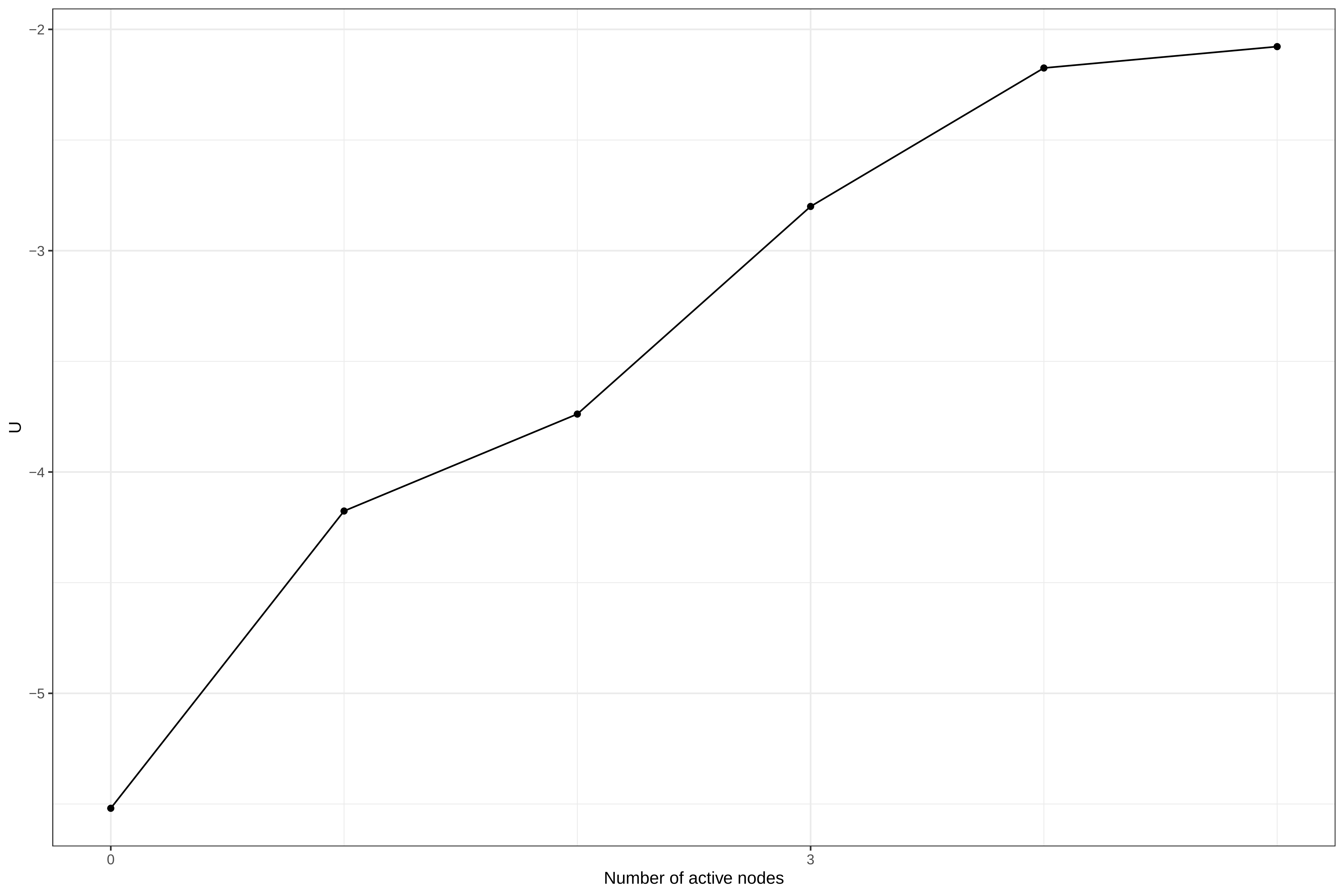

−2
−3
−4
−5
U
0
3
Number of active nodes

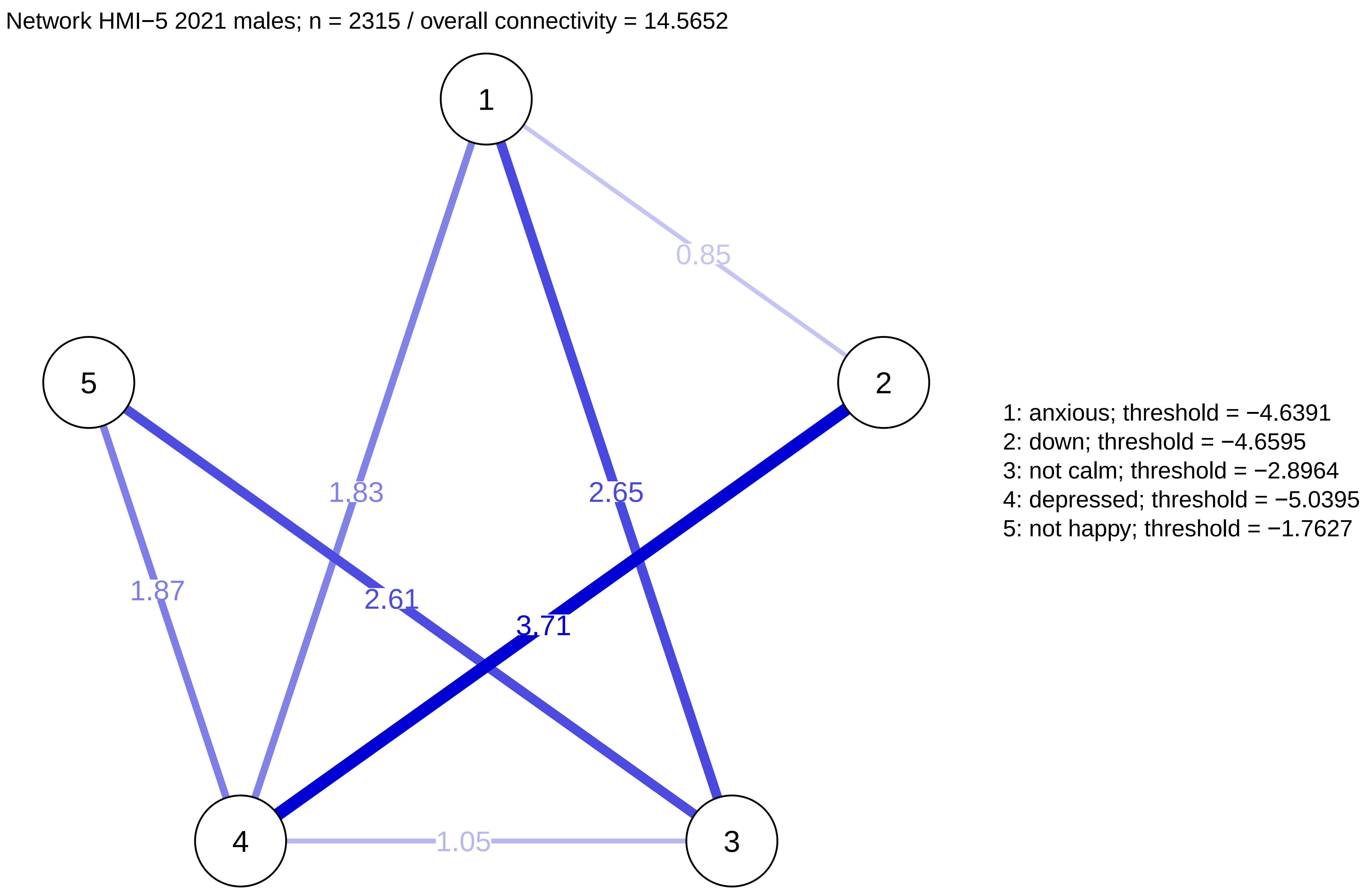
Network HMI−5 2021 males; n = 2315 / overall connectivity = 14.5652
1
2
3
4
5
0.85
1.83
2.65
1.87
2.61
3.71
1.05
1: anxious; threshold = −4.6391
2: down; threshold = −4.6595
3: not calm; threshold = −2.8964
4: depressed; threshold = −5.0395
5: not happy; threshold = −1.7627

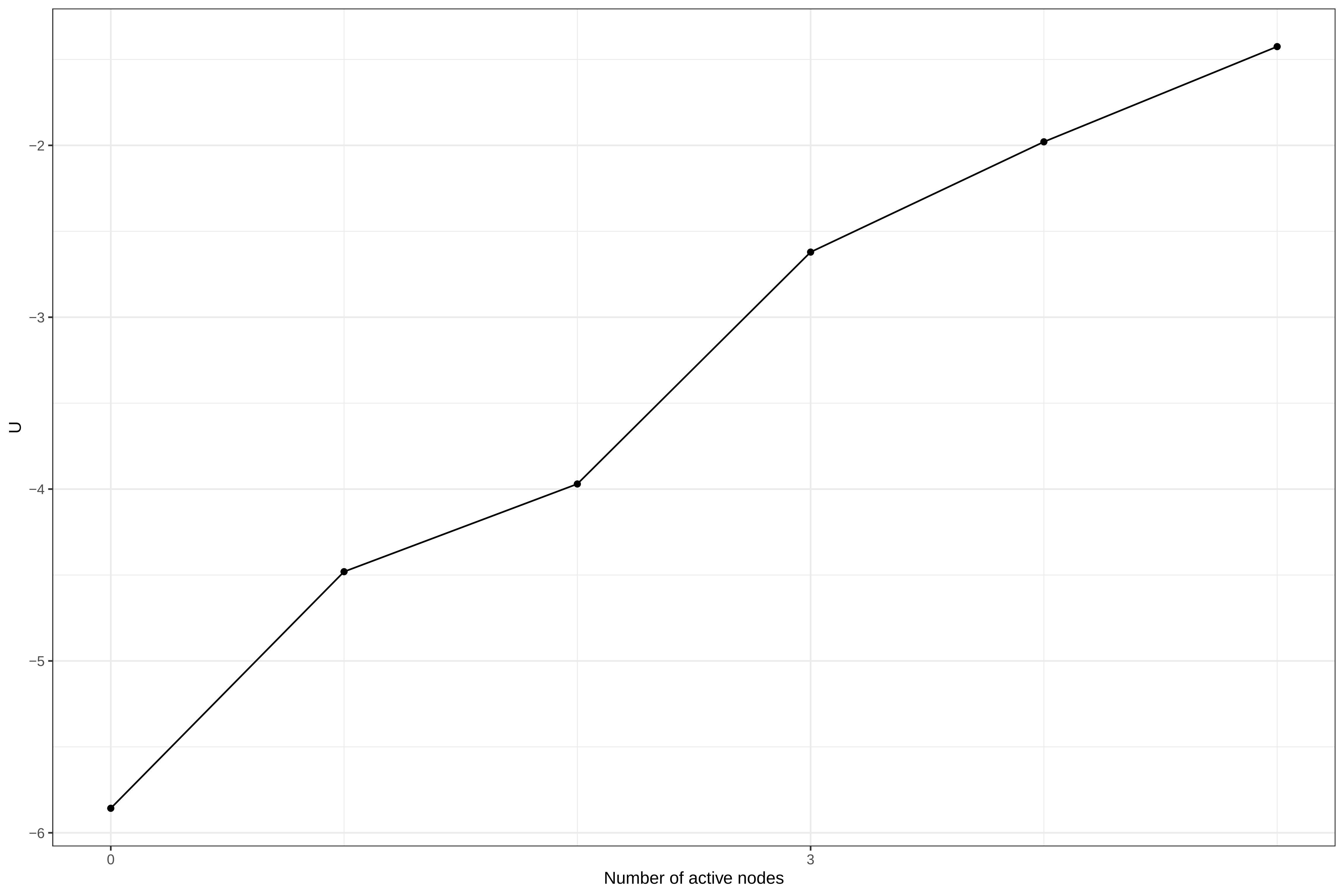

U
−2
−3
−4
−5
−6
0
3
Number of active nodes

Network HMI−5 2021 females; n = 2671 / overall connectivity = 14.6327

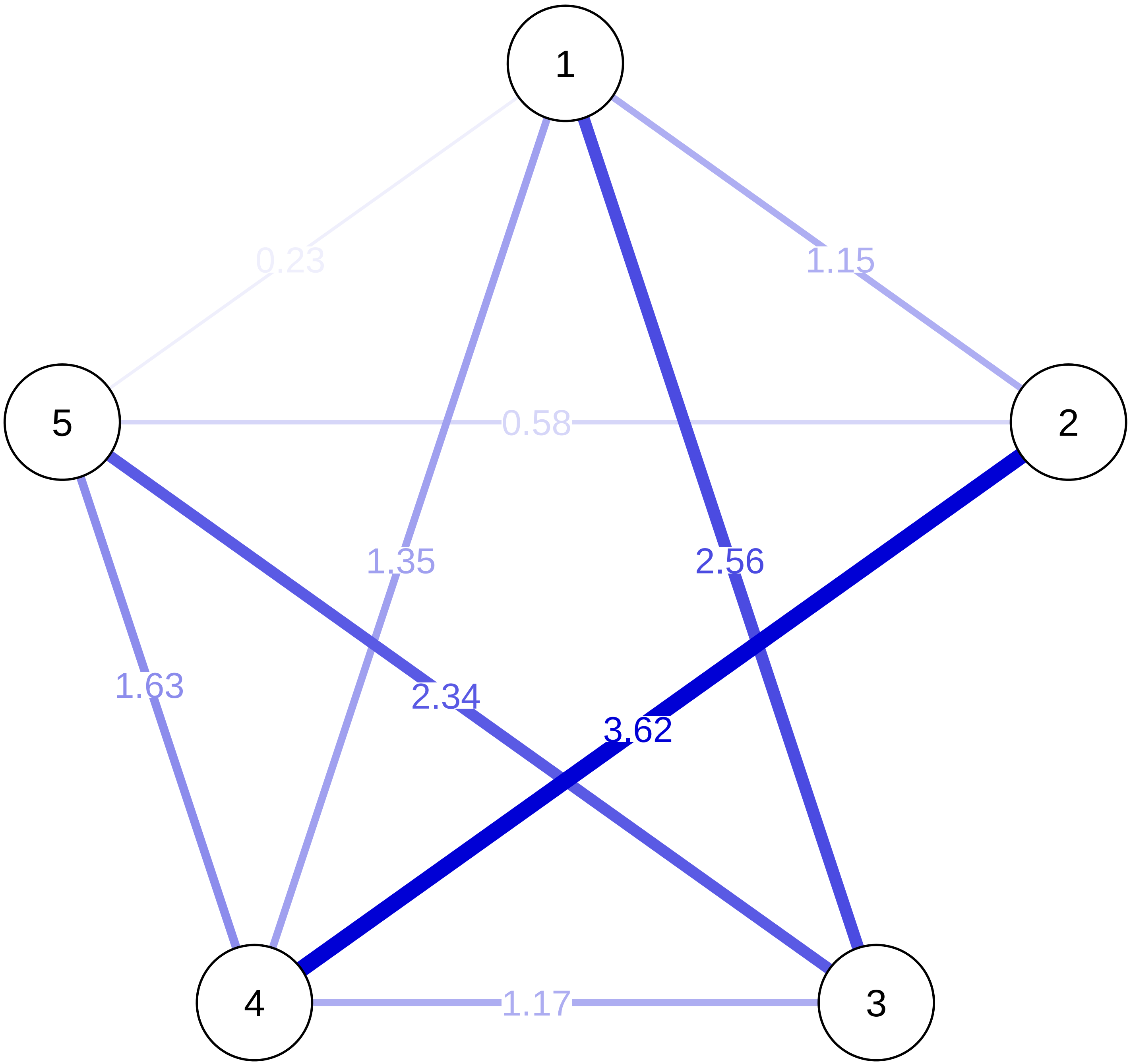

1: anxious; threshold = −4.1772
2: down; threshold = −5.145
3: not calm; threshold = −2.3675
4: depressed; threshold = −4.7306
5: not happy; threshold = −1.9658

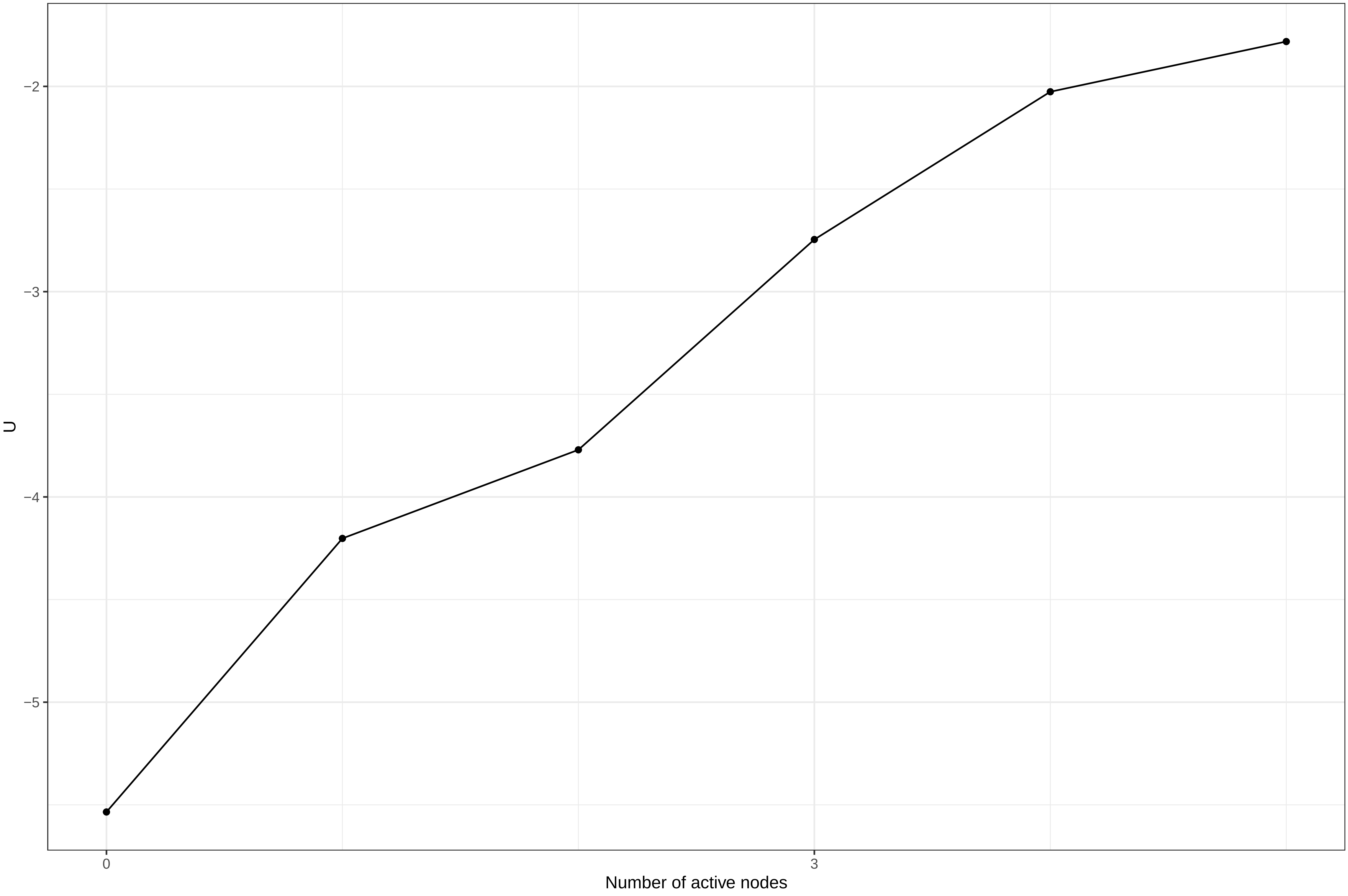

−2
−3
−4
−5
U
0
3
Number of active nodes

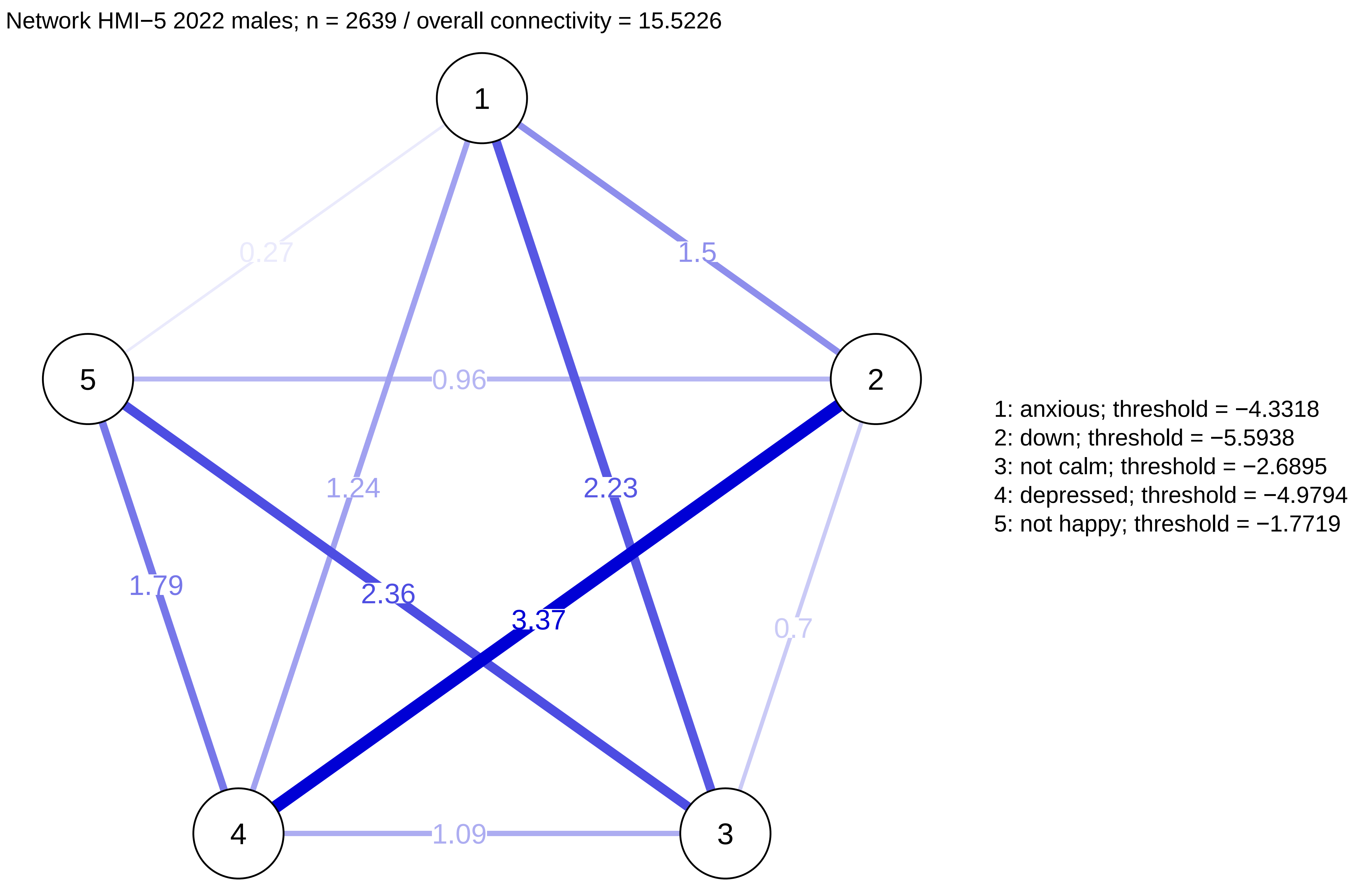

Network HMI−5 2022 males; n = 2639 / overall connectivity = 15.5226
1
2
3
4
5
0.27
1.5
0.96
1.24
2.23
1.79
2.36
3.37
0.7
1.09
1: anxious; threshold = −4.3318
2: down; threshold = −5.5938
3: not calm; threshold = −2.6895
4: depressed; threshold = −4.9794
5: not happy; threshold = −1.7719

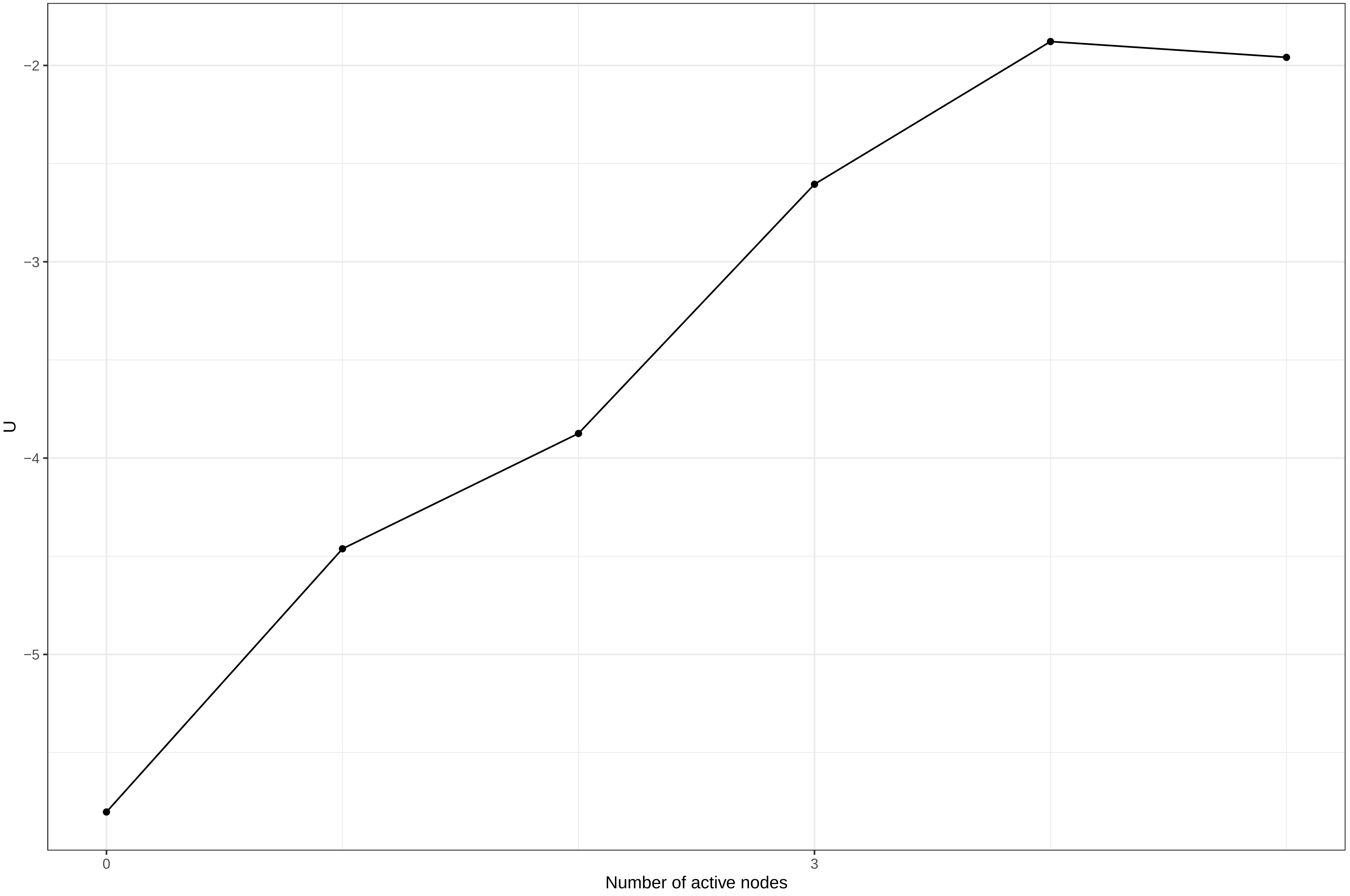

−2
−3
−4
−5
U
0
3
Number of active nodes

Network HMI−5 2022 females; n = 3072 / overall connectivity = 15.2774

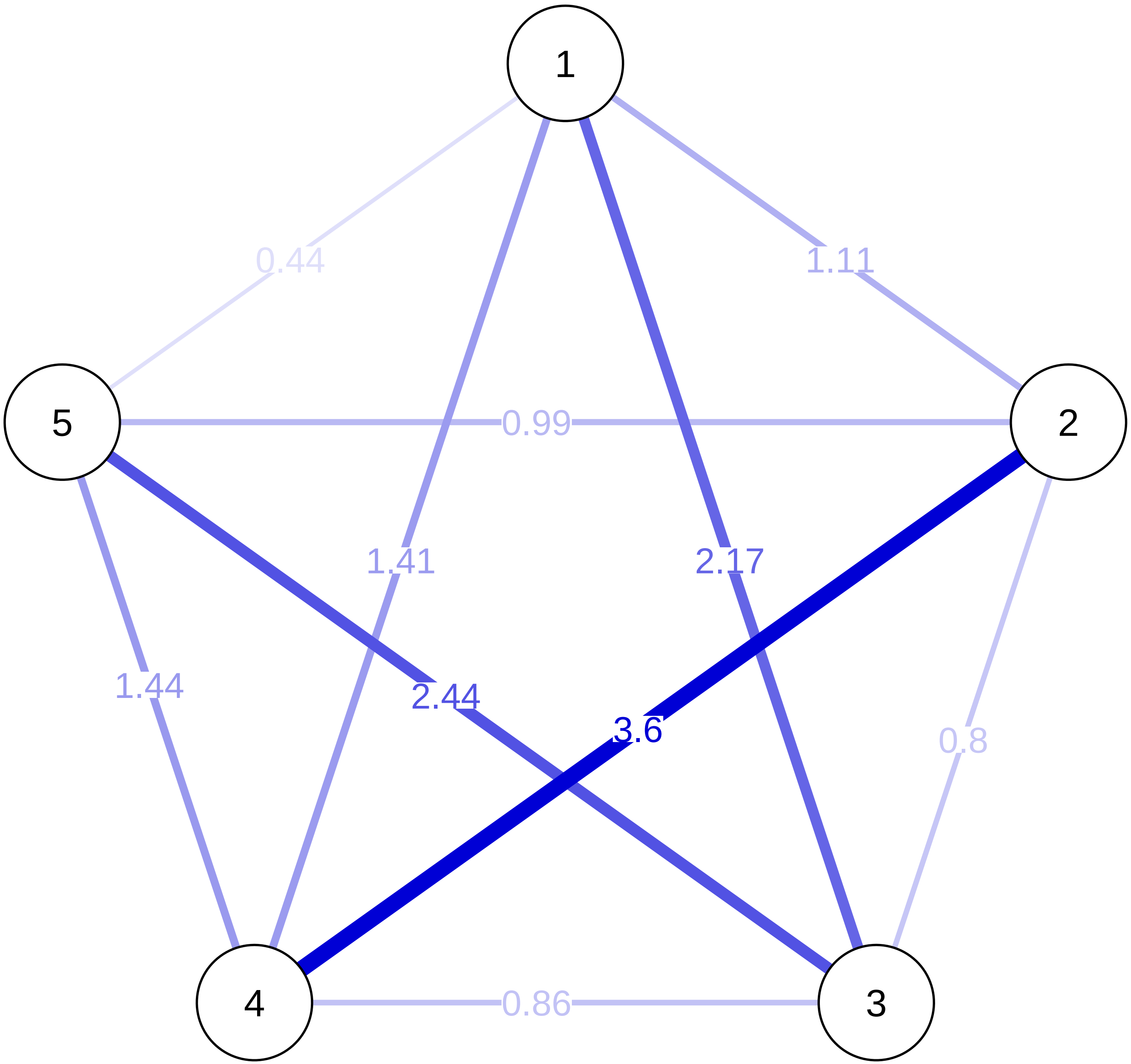

1: anxious; threshold = −3.9333
2: down; threshold = −5.7865
3: not calm; threshold = −2.3558
4: depressed; threshold = −4.699
5: not happy; threshold = −1.9051

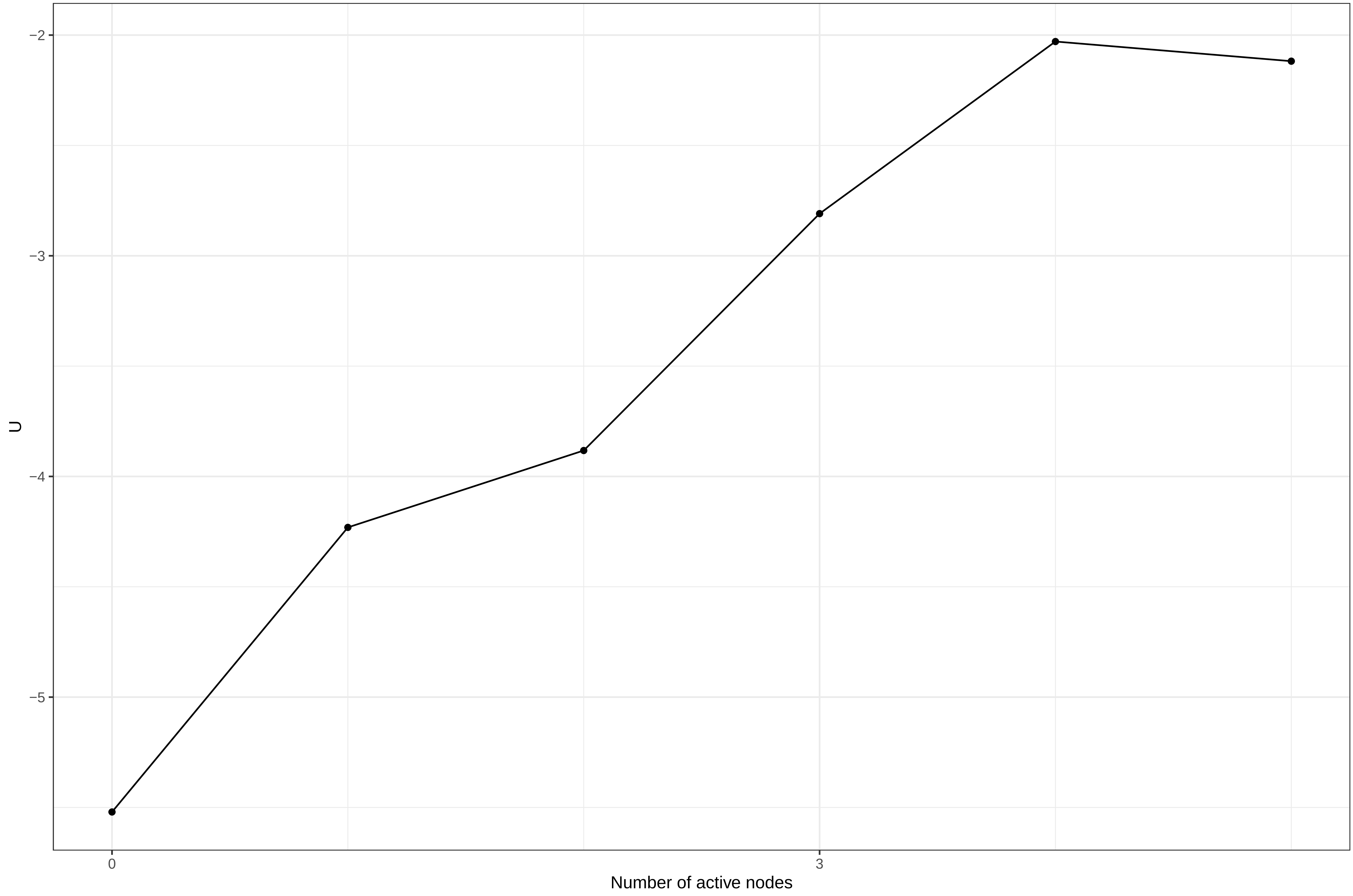

−2
−3
−4
−5
U
0
3
Number of active nodes

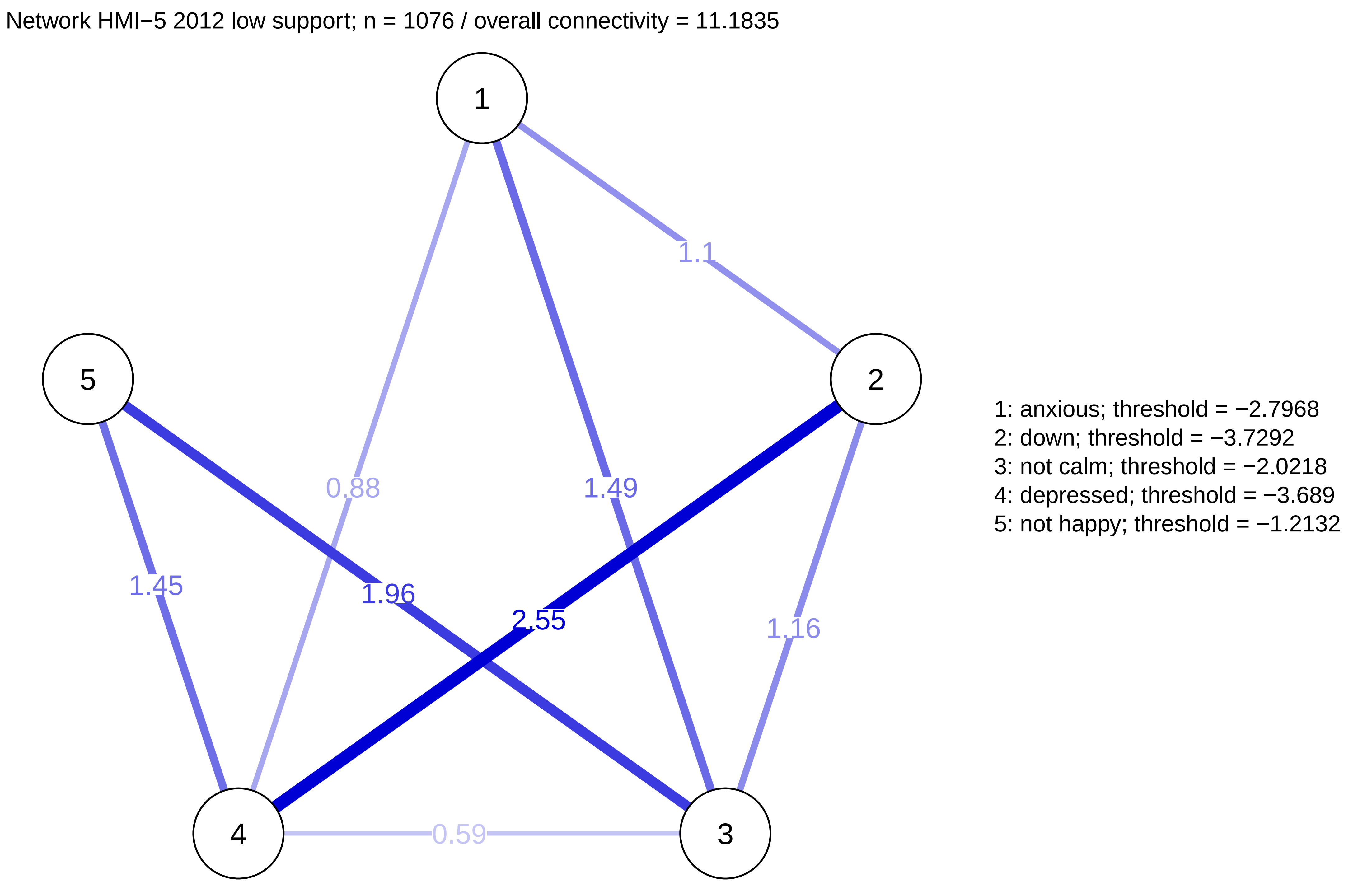
Network HMI−5 2012 low support; n = 1076 / overall connectivity = 11.1835
1
2
3
4
5
1.1
0.88
1.49
1.45
1.96
2.55
1.16
0.59
1: anxious; threshold = −2.7968
2: down; threshold = −3.7292
3: not calm; threshold = −2.0218
4: depressed; threshold = −3.689
5: not happy; threshold = −1.2132

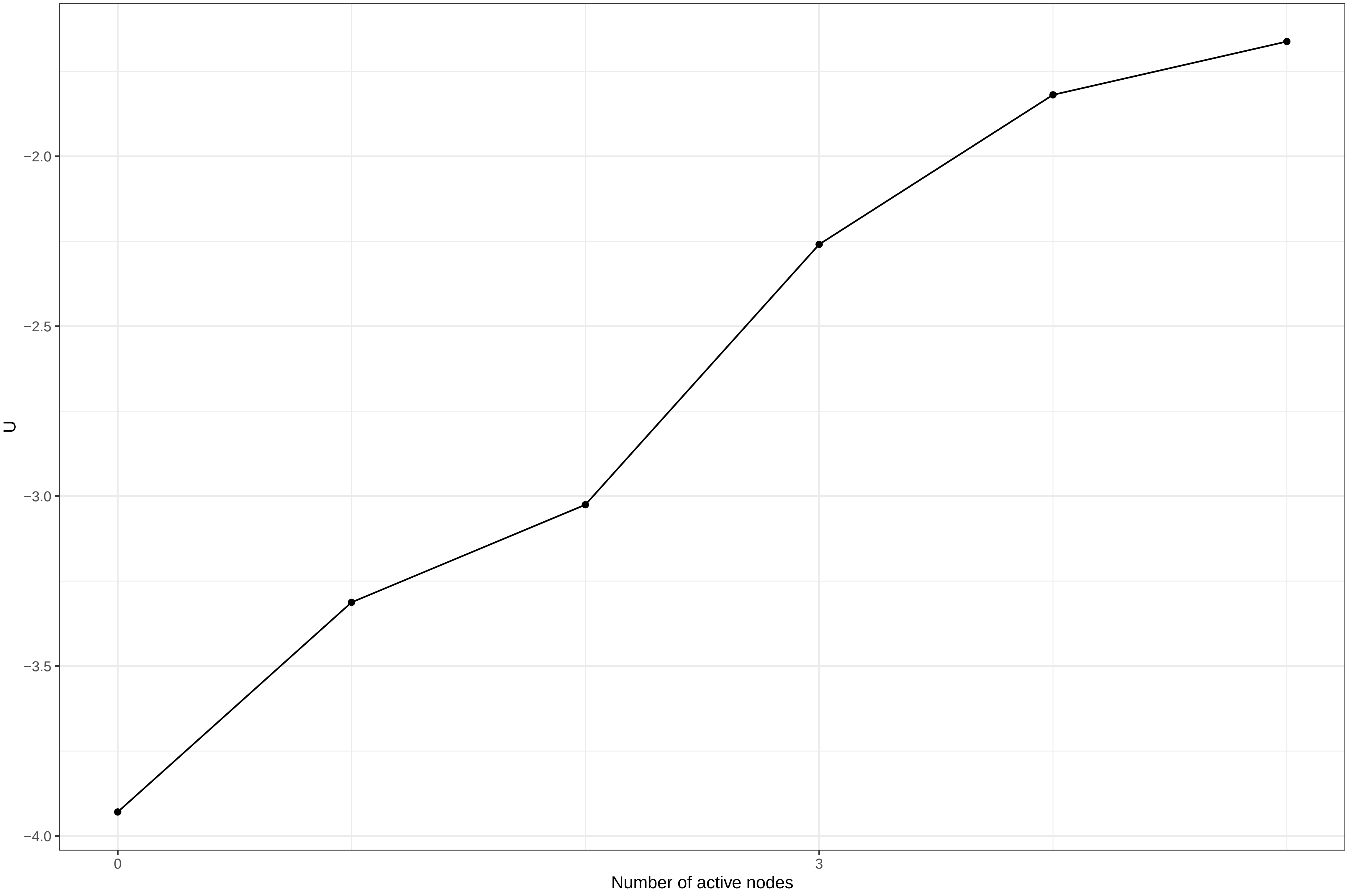

−2.0
−2.5
−3.0
−3.5
−4.0
U
0
3
Number of active nodes

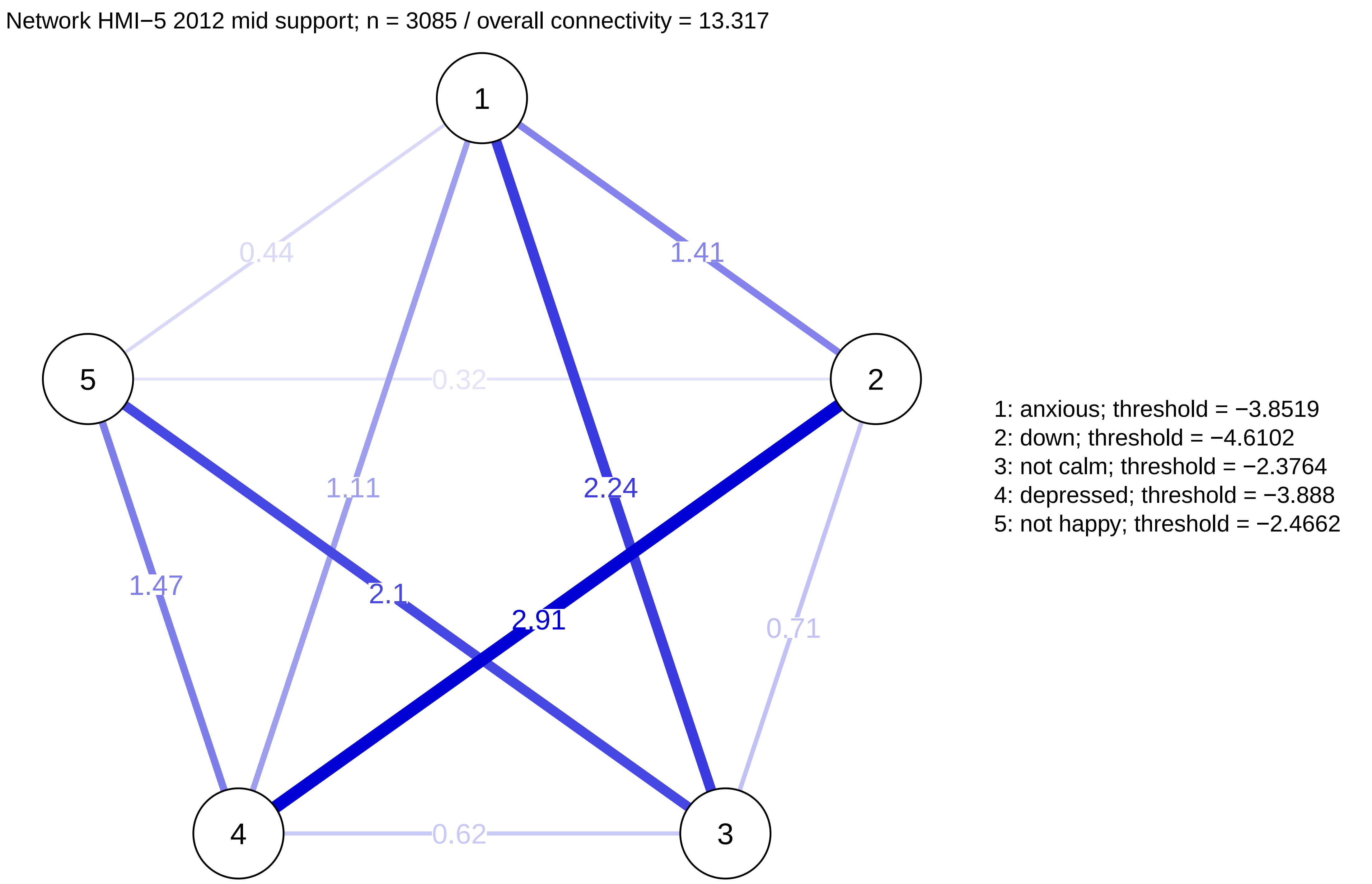
Network HMI−5 2012 mid support; n = 3085 / overall connectivity = 13.317
1
2
3
4
5
0.44
1.41
0.32
1.11
2.24
1.47
2.1
2.91
0.71
0.62
1: anxious; threshold = −3.8519
2: down; threshold = −4.6102
3: not calm; threshold = −2.3764
4: depressed; threshold = −3.888
5: not happy; threshold = −2.4662

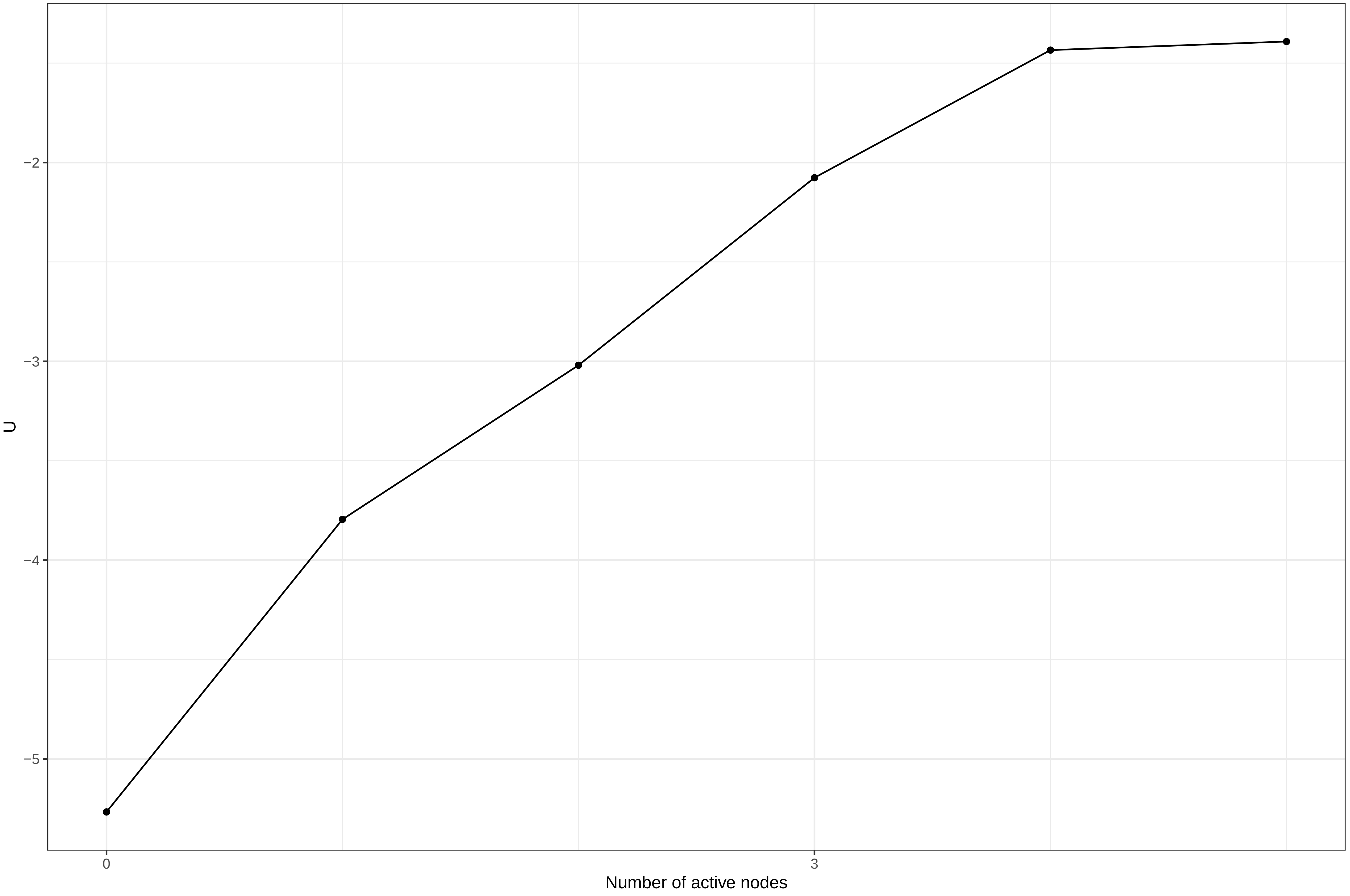

−2
−3
−4
−5
U
0
3
Number of active nodes

Network HMI−5 2012 high support; n = 867 / overall connectivity = 13.5605

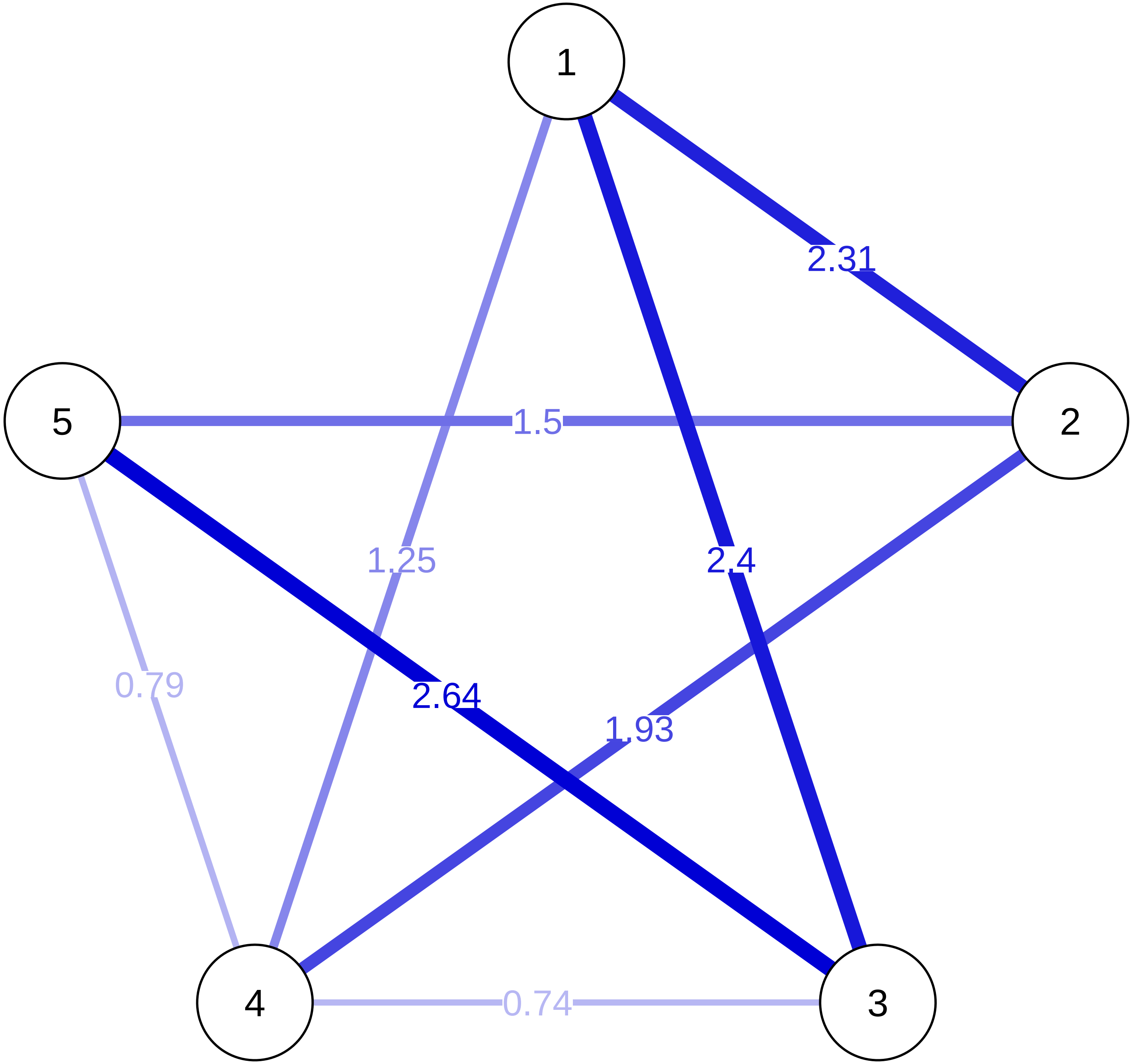

1: anxious; threshold = −3.9419
2: down; threshold = −4.6285
3: not calm; threshold = −2.4236
4: depressed; threshold = −3.7228
5: not happy; threshold = −3.3222

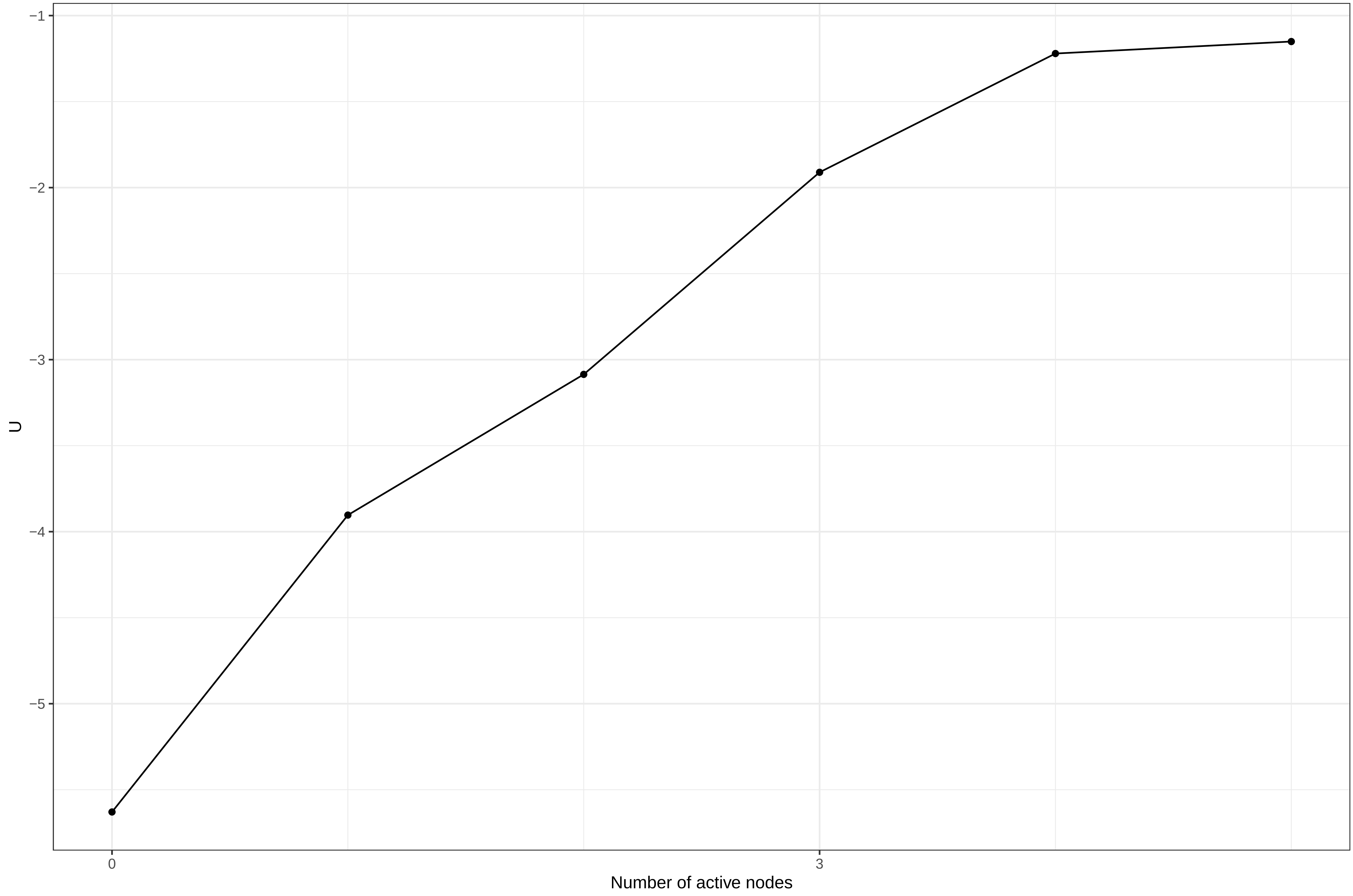

−1
−2
−3
−4
−5
U
0
3
Number of active nodes

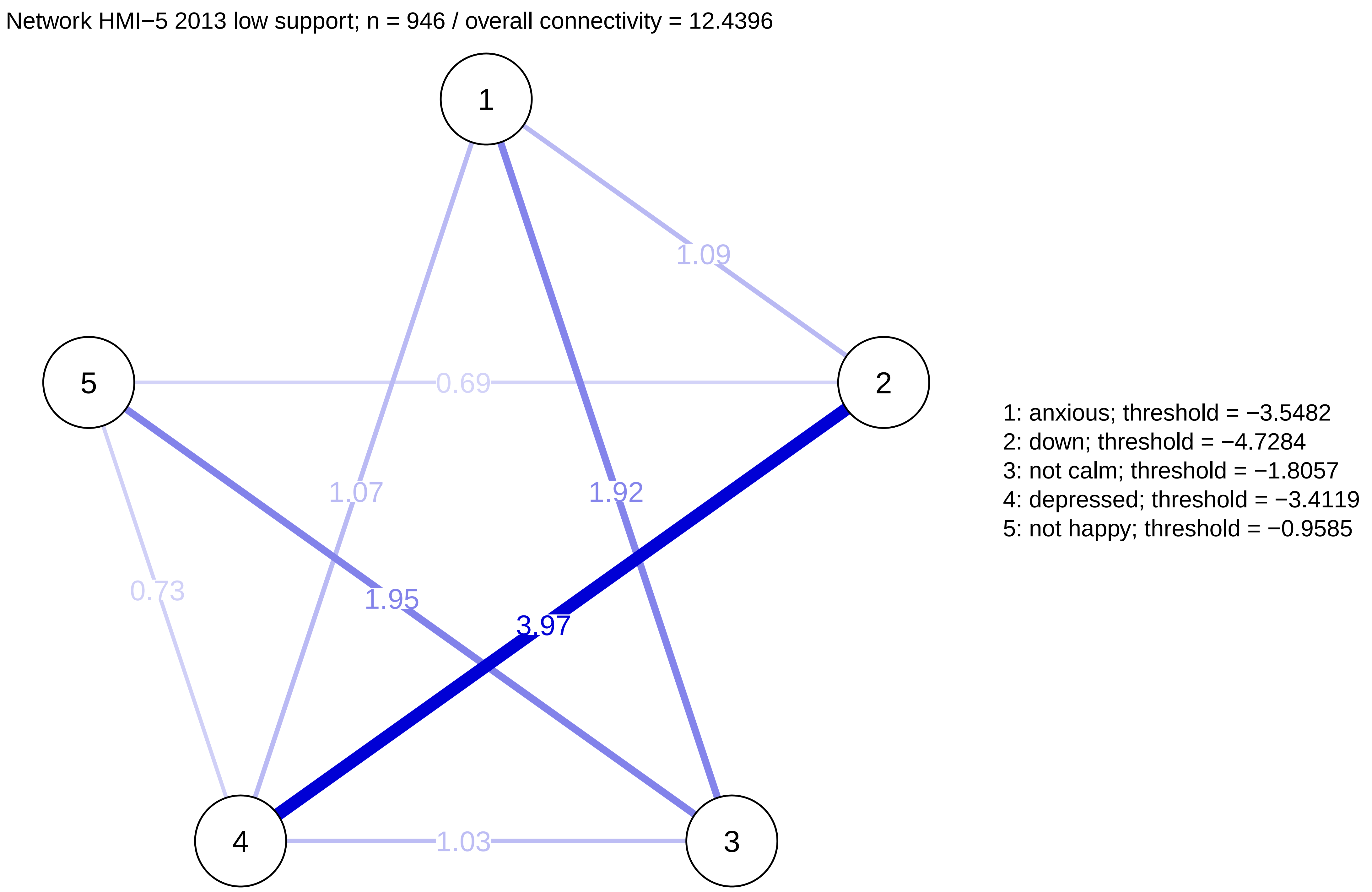
Network HMI−5 2013 low support; n = 946 / overall connectivity = 12.4396
1
2
3
4
5
1.09
0.69
1.07
1.92
0.73
1.95
3.97
1.03
1: anxious; threshold = −3.5482
2: down; threshold = −4.7284
3: not calm; threshold = −1.8057
4: depressed; threshold = −3.4119
5: not happy; threshold = −0.9585

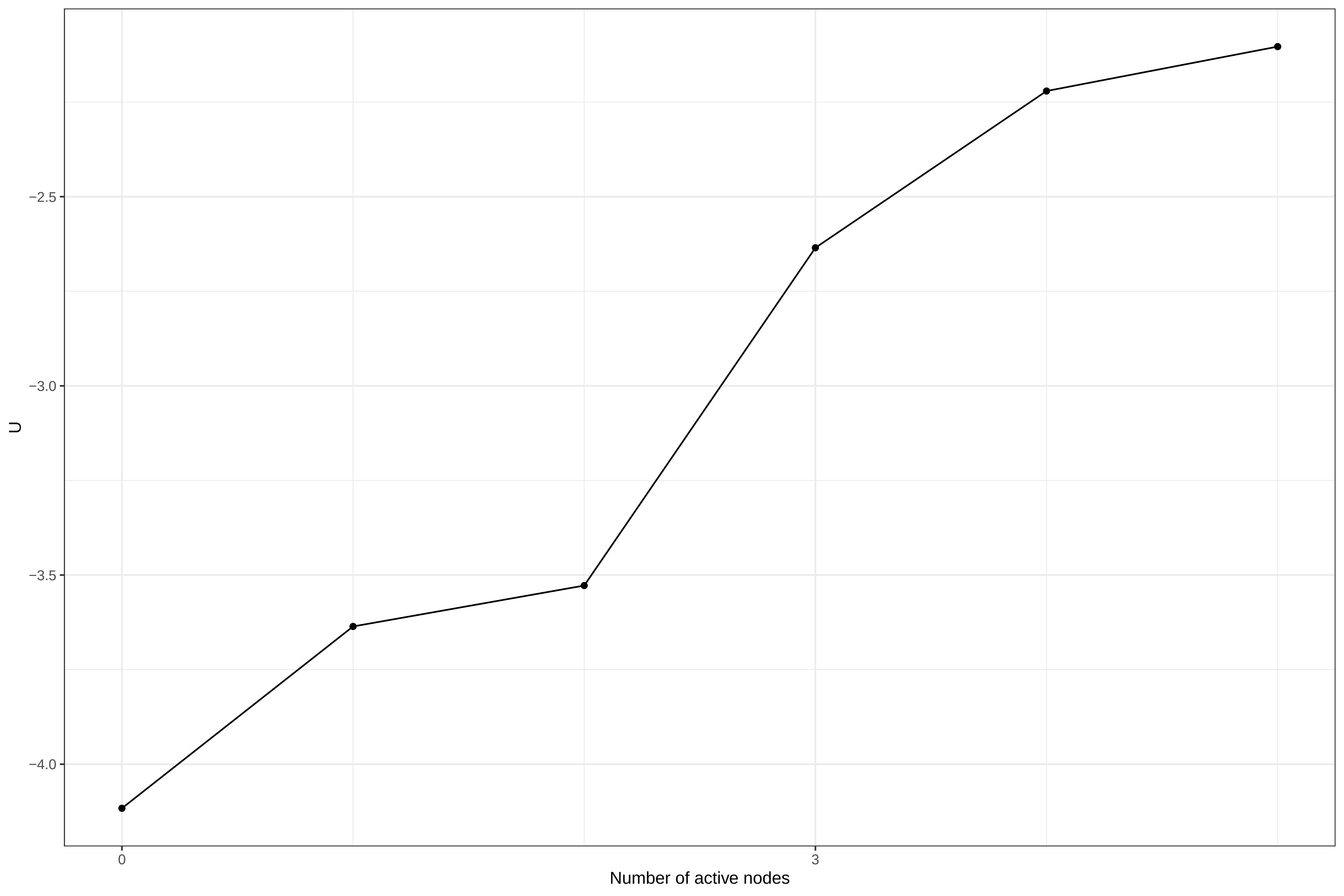

−2.5
−3.0
−3.5
−4.0
U
0
3
Number of active nodes

Network HMI−5 2013 mid support; n = 2972 / overall connectivity = 13.4851

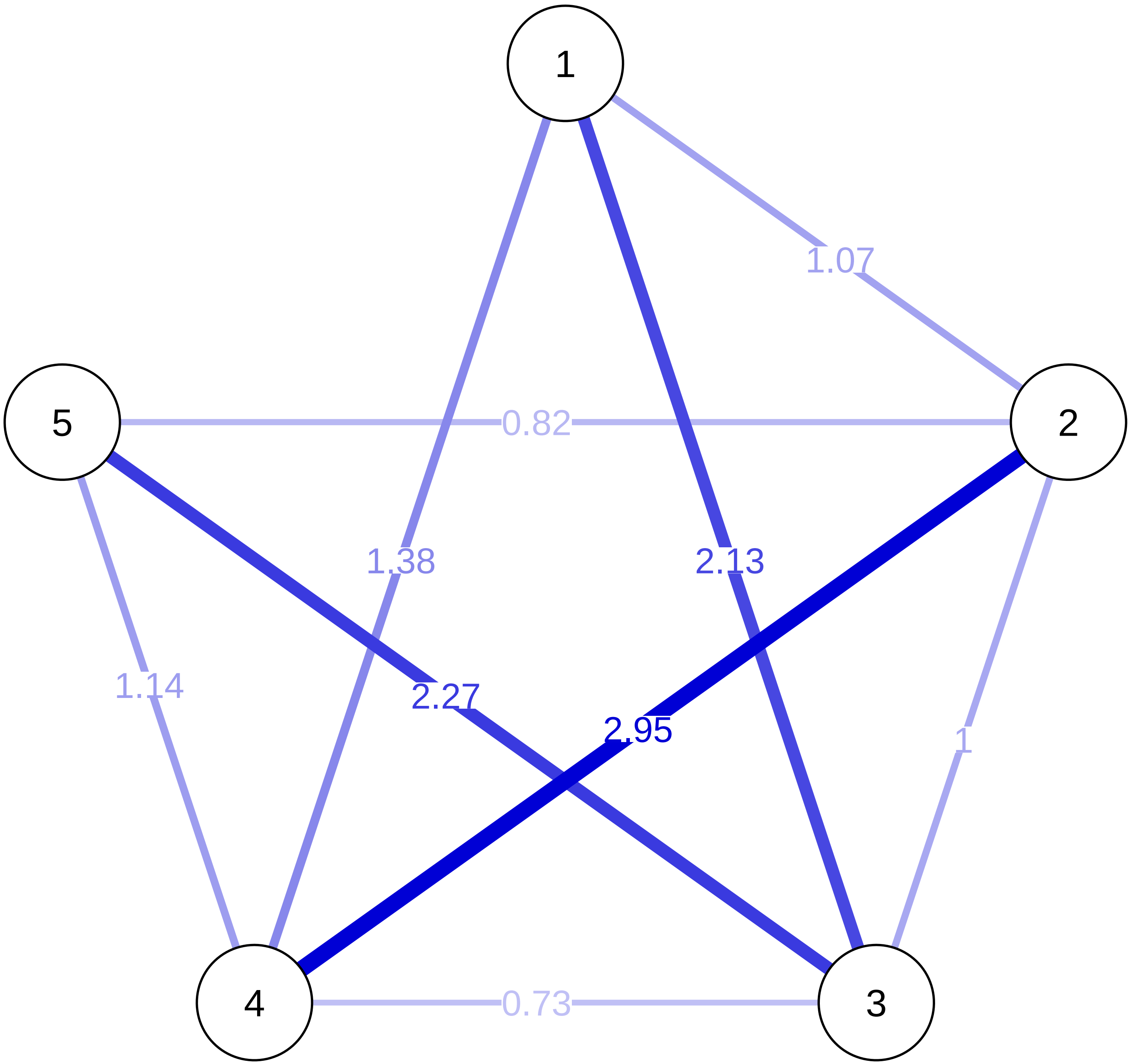

1: anxious; threshold = −3.9818
2: down; threshold = −5.1788
3: not calm; threshold = −2.303
4: depressed; threshold = −3.9503
5: not happy; threshold = −2.3053

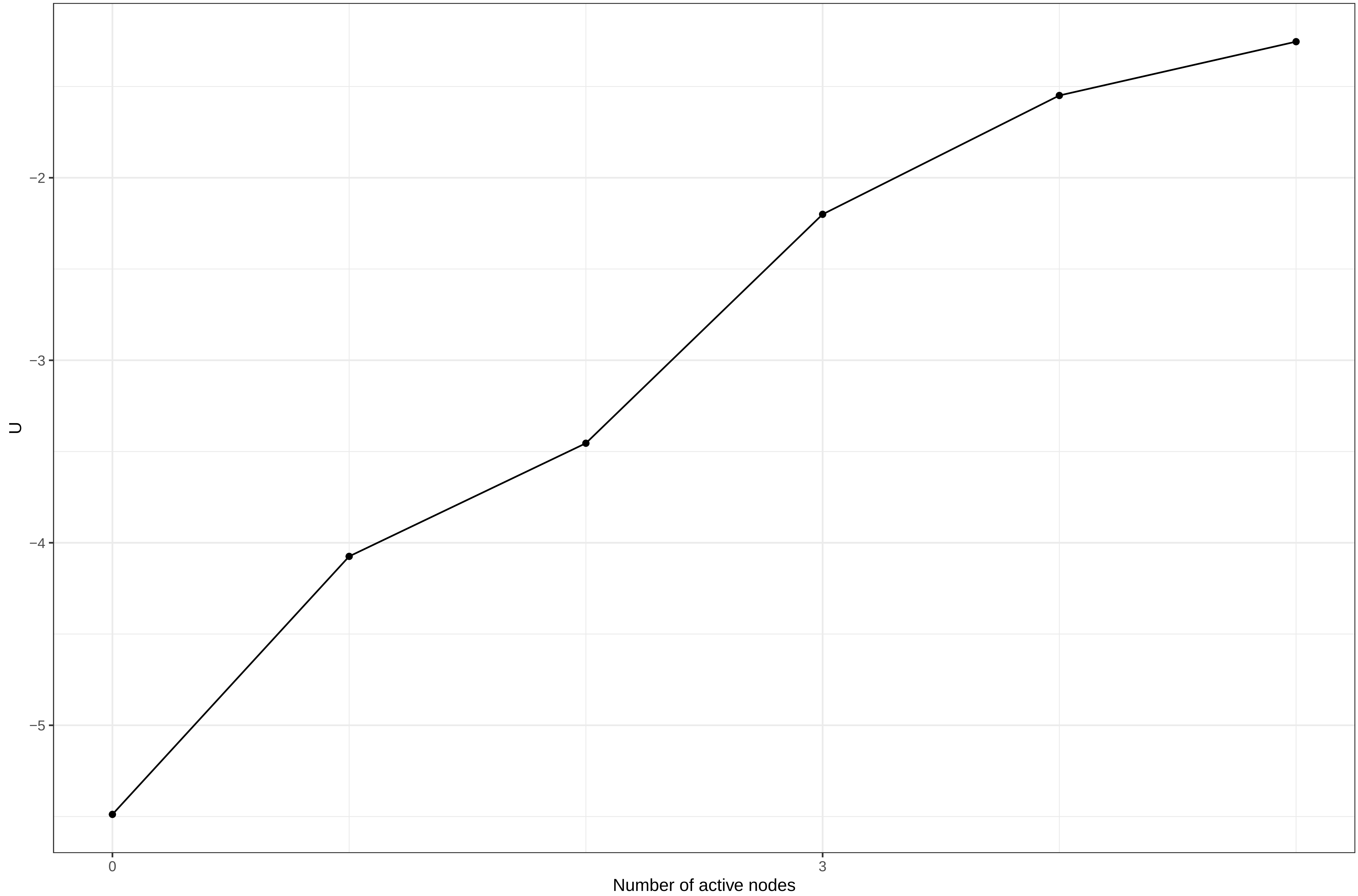
−2
−3
−4
−5
U
0
3
Number of active nodes

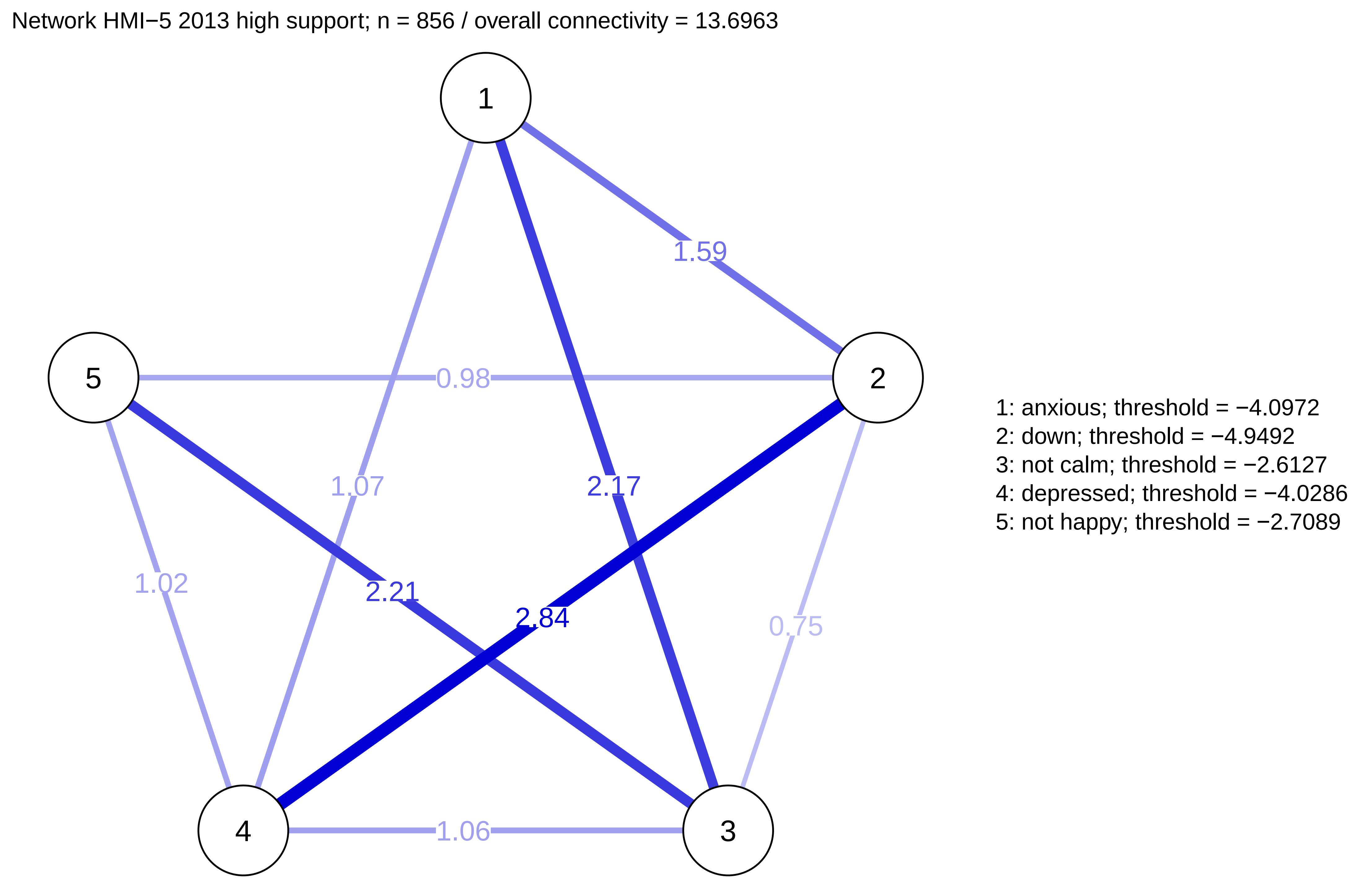

Network HMI−5 2013 high support; n = 856 / overall connectivity = 13.6963
1
2
3
4
5
1.59
0.98
1.07
2.17
1.02
2.21
2.84
0.75
1.06
1: anxious; threshold = −4.0972
2: down; threshold = −4.9492
3: not calm; threshold = −2.6127
4: depressed; threshold = −4.0286
5: not happy; threshold = −2.7089

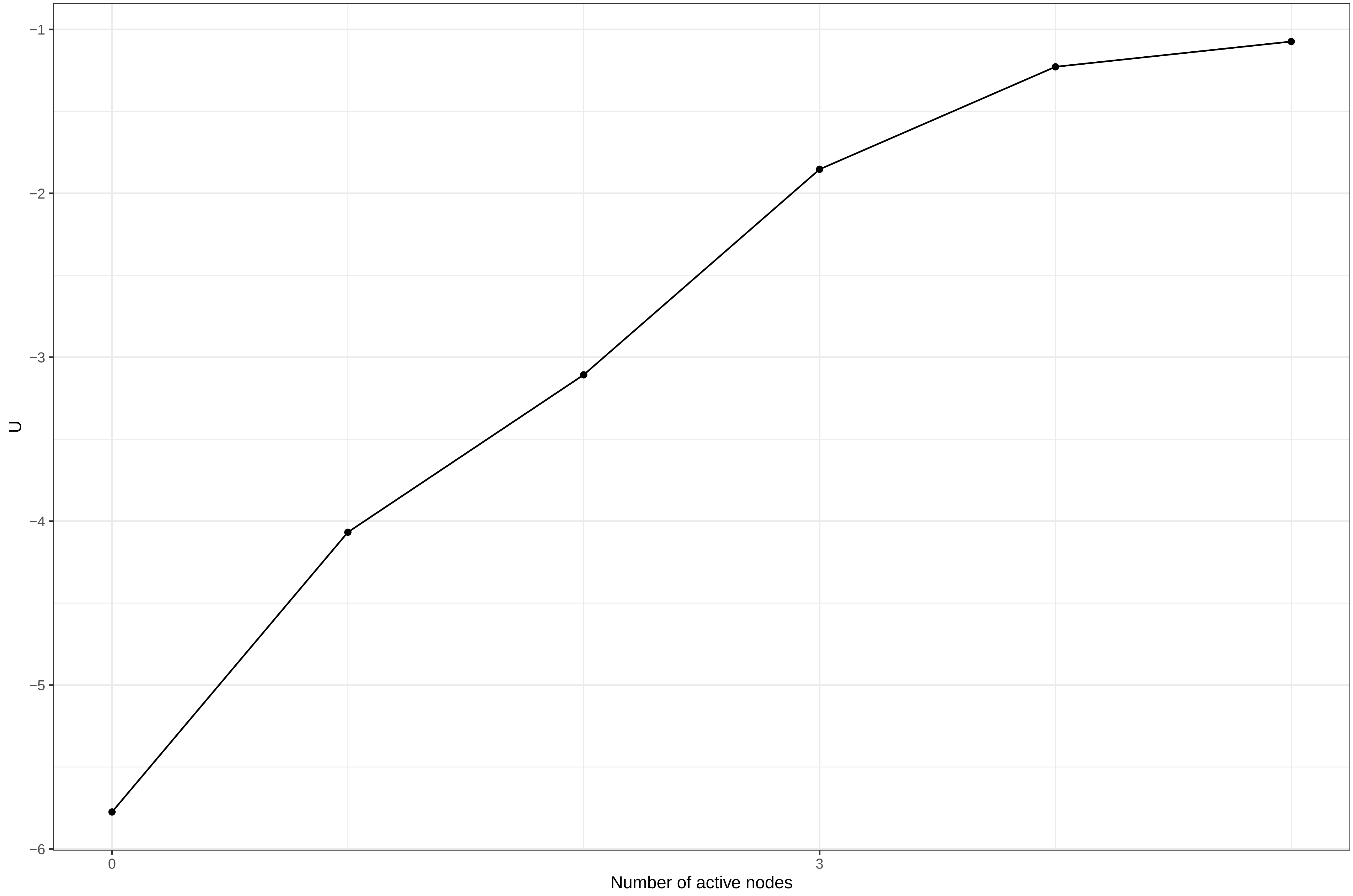

−1
−2
−3
−4
−5
−6
U
0
3
Number of active nodes

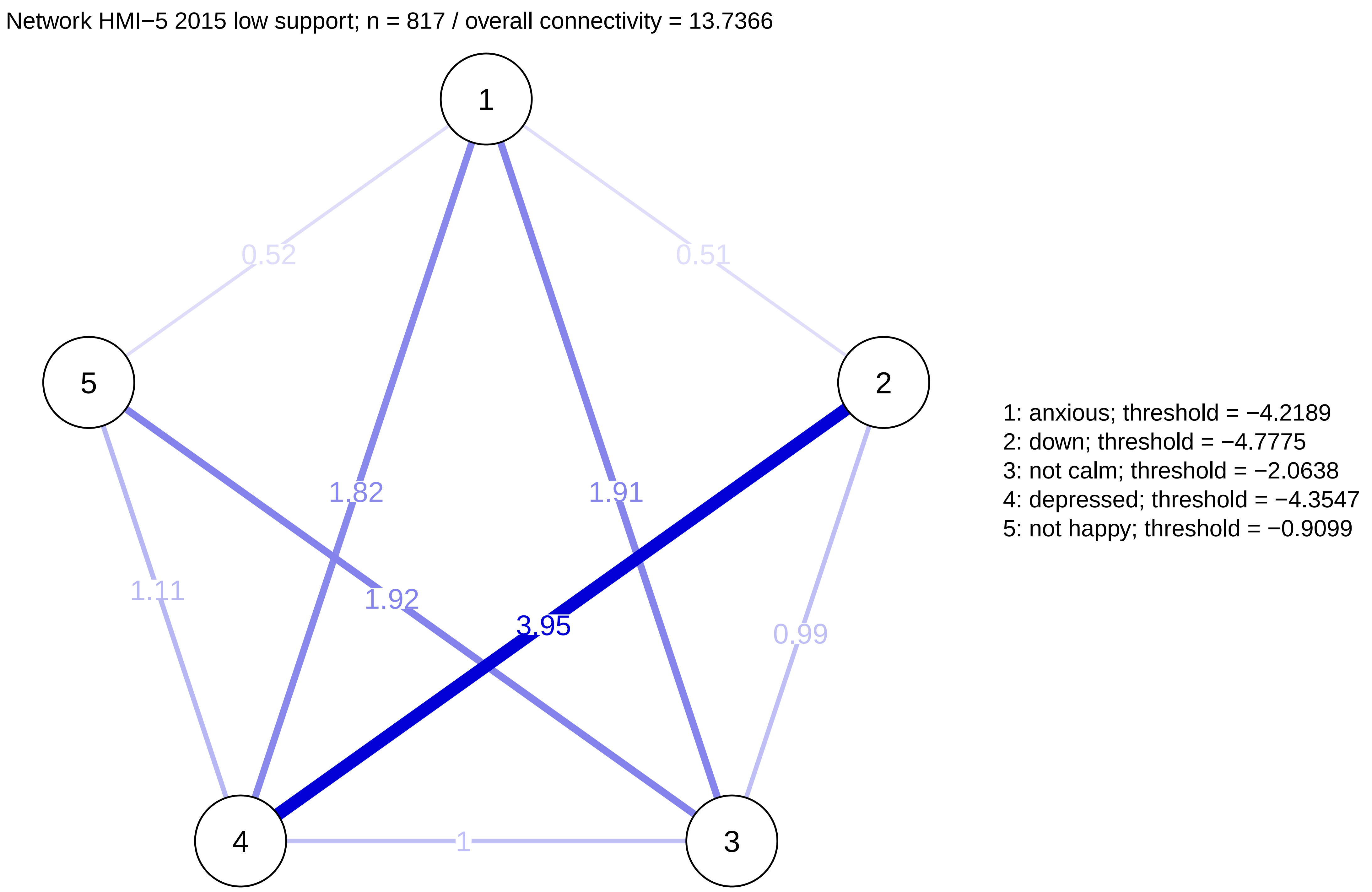
Network HMI−5 2015 low support; n = 817 / overall connectivity = 13.7366
1
2
3
4
5
0.52
0.51
1.82
1.91
1.11
1.92
3.95
0.99
1
1: anxious; threshold = −4.2189
2: down; threshold = −4.7775
3: not calm; threshold = −2.0638
4: depressed; threshold = −4.3547
5: not happy; threshold = −0.9099

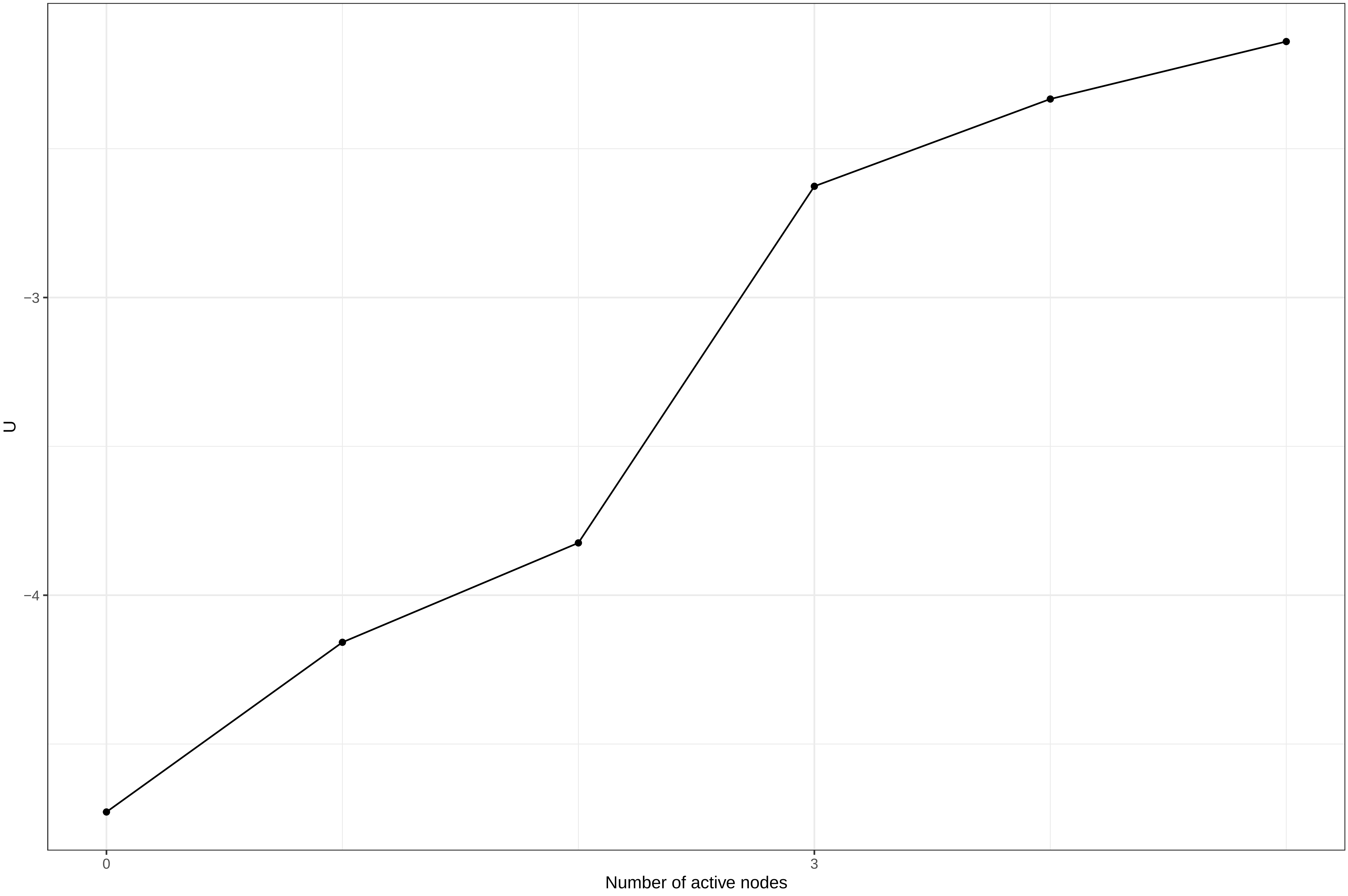

U
−3
−4
0
3
Number of active nodes

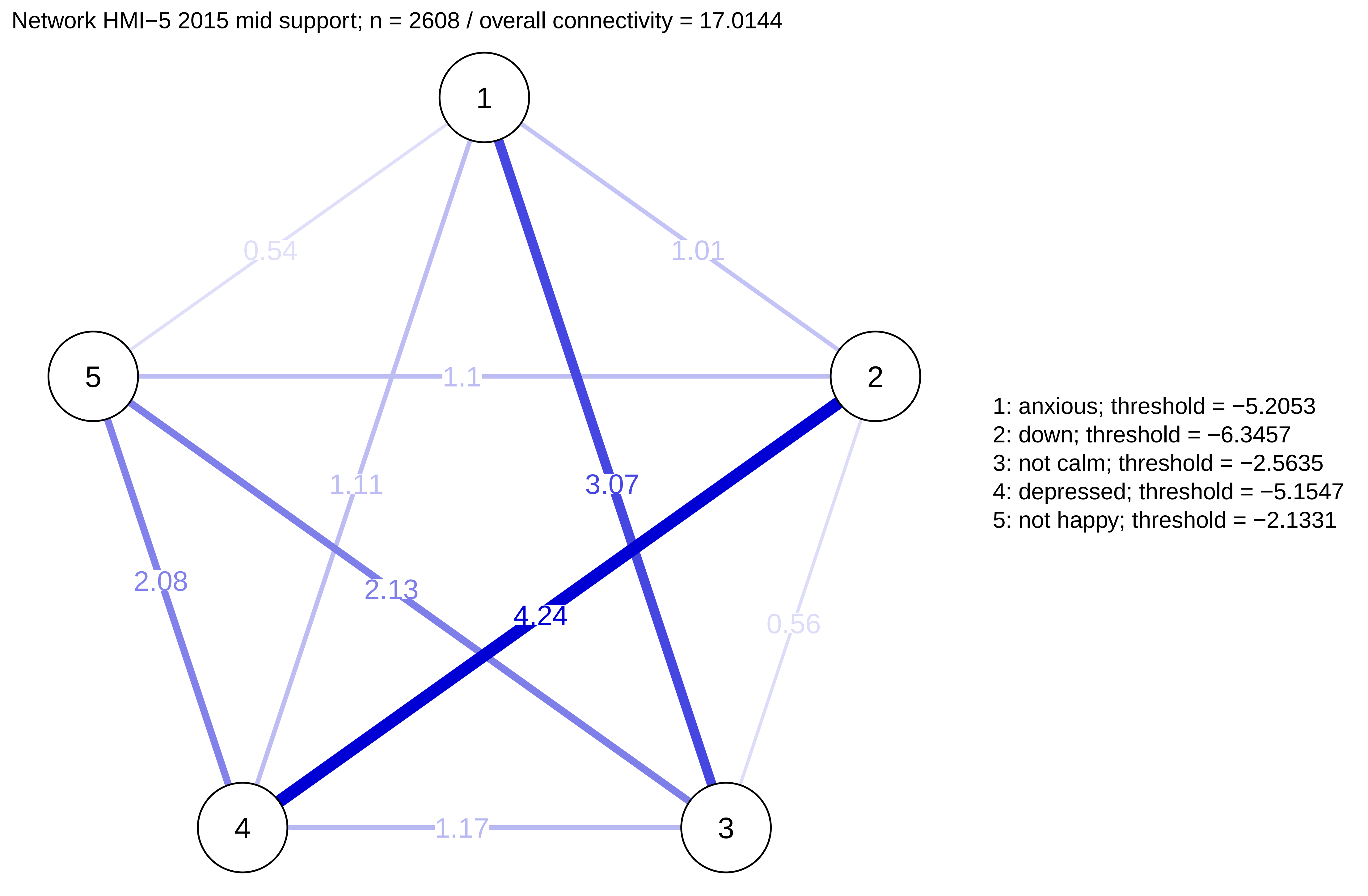
Network HMI−5 2015 mid support; n = 2608 / overall connectivity = 17.0144
1
2
3
4
5
0.54
1.01
1.1
1.11
3.07
2.08
2.13
4.24
0.56
1.17
1: anxious; threshold = −5.2053
2: down; threshold = −6.3457
3: not calm; threshold = −2.5635
4: depressed; threshold = −5.1547
5: not happy; threshold = −2.1331

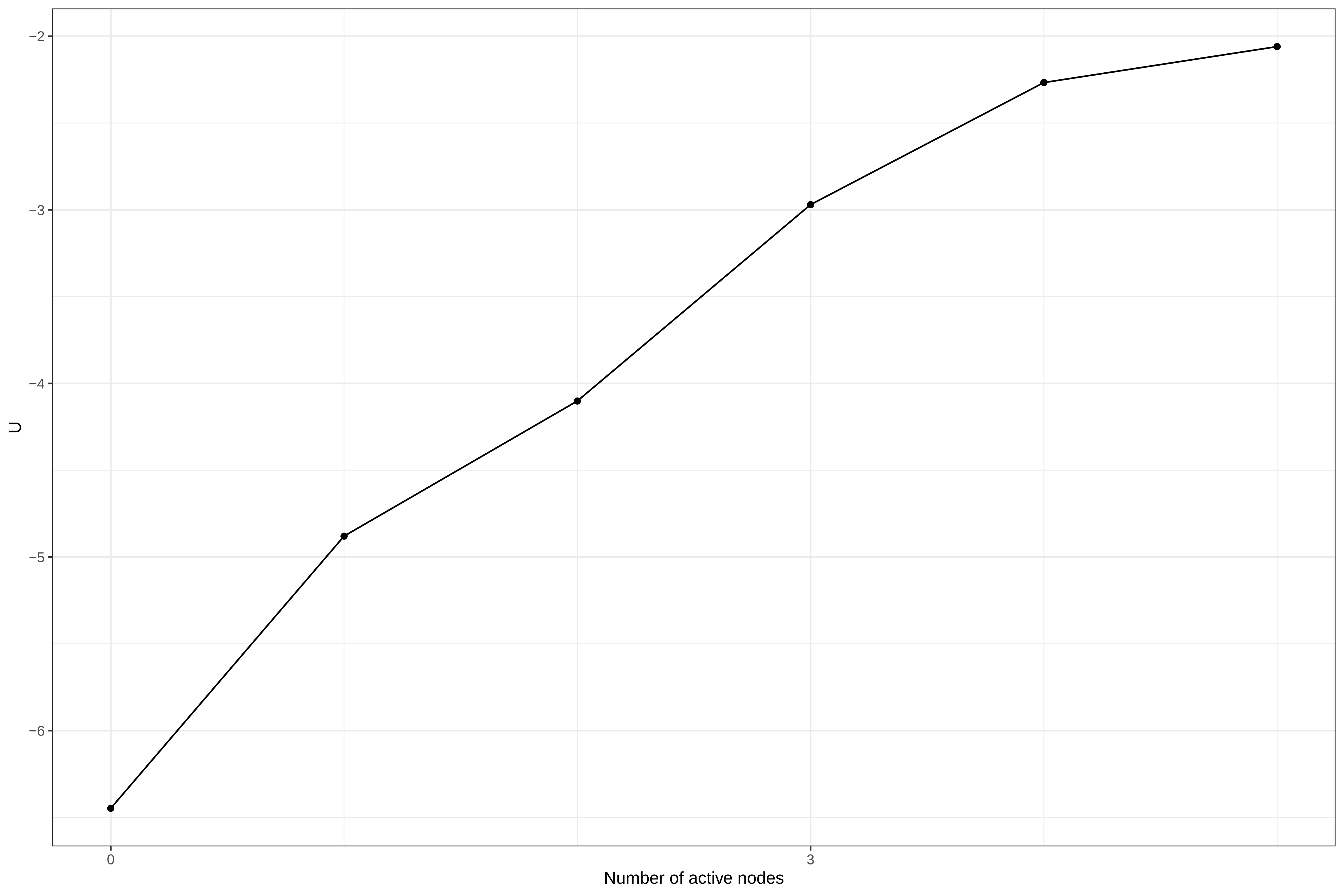

−2
−3
−4
−5
−6
U
0
3
Number of active nodes

Network HMI−5 2015 high support; n = 618 / overall connectivity = 16.0993

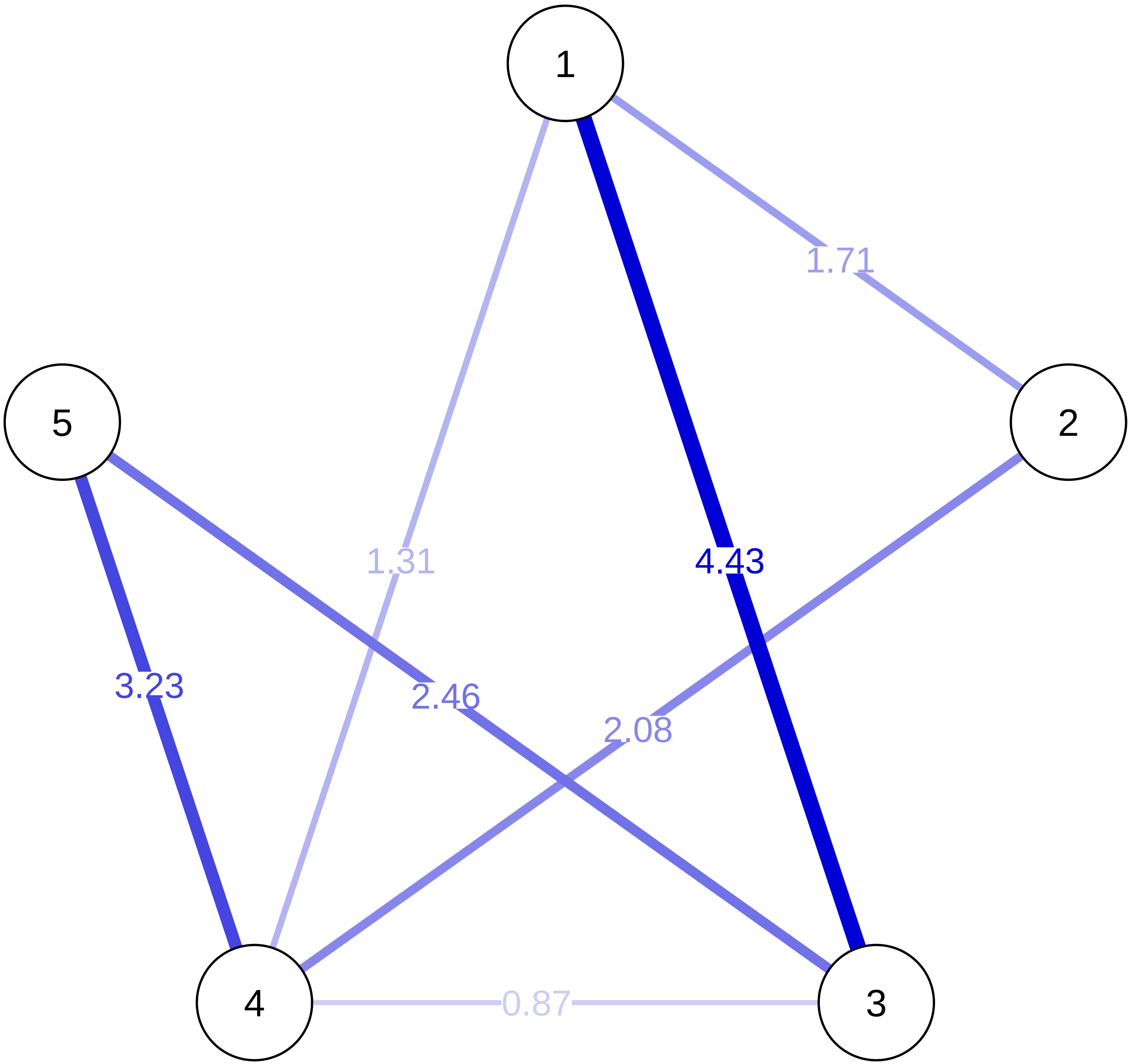

1: anxious; threshold = −6.048
2: down; threshold = −4.5915
3: not calm; threshold = −2.8086
4: depressed; threshold = −5.3111
5: not happy; threshold = −2.9126

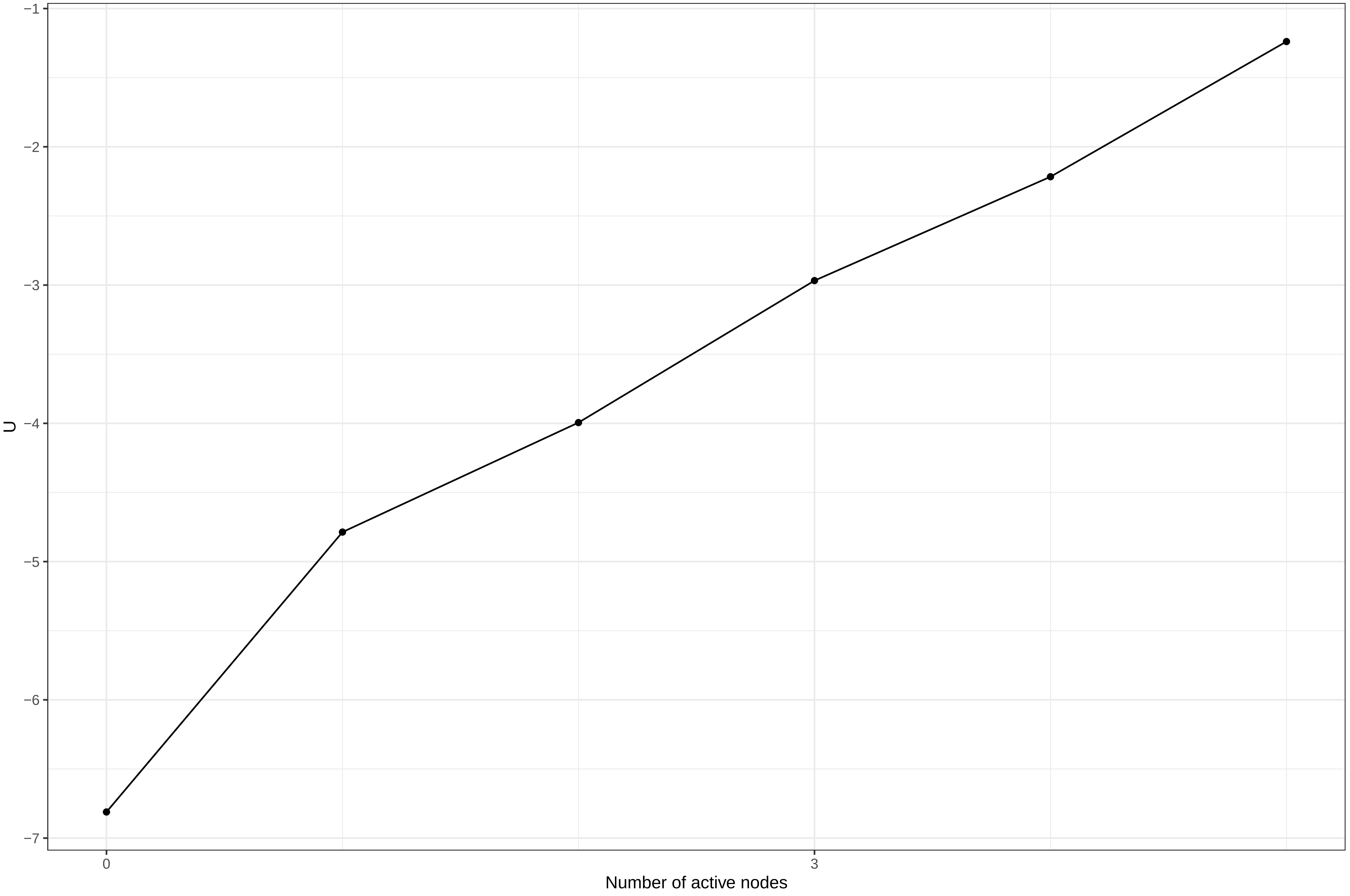

−1
−2
−3
−4
−5
−6
−7
U
0
3
Number of active nodes

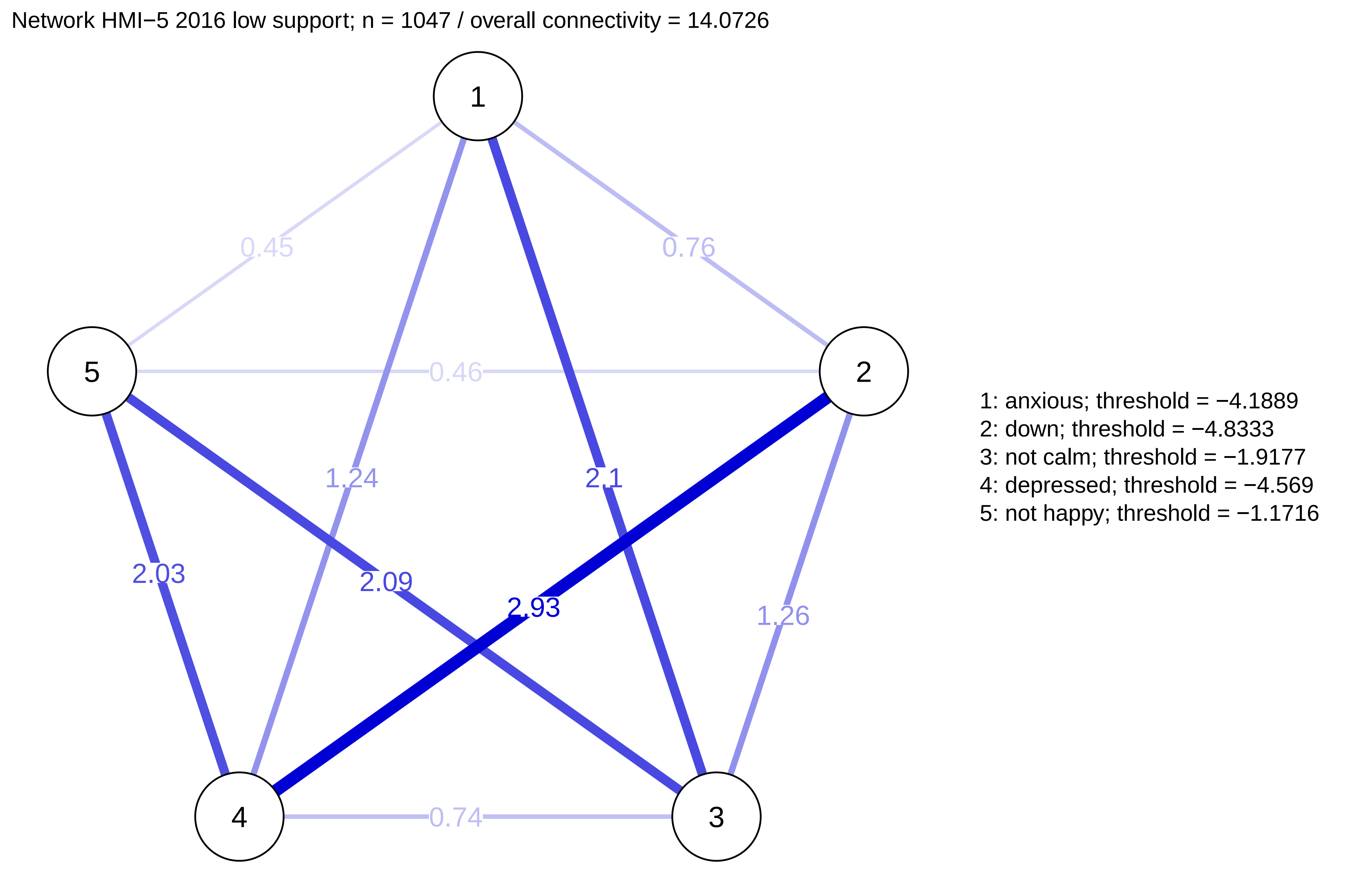
Network HMI−5 2016 low support; n = 1047 / overall connectivity = 14.0726
1
2
3
4
5
0.45
0.76
0.46
1.24
2.1
2.03
2.09
2.93
1.26
0.74
1: anxious; threshold = −4.1889
2: down; threshold = −4.8333
3: not calm; threshold = −1.9177
4: depressed; threshold = −4.569
5: not happy; threshold = −1.1716

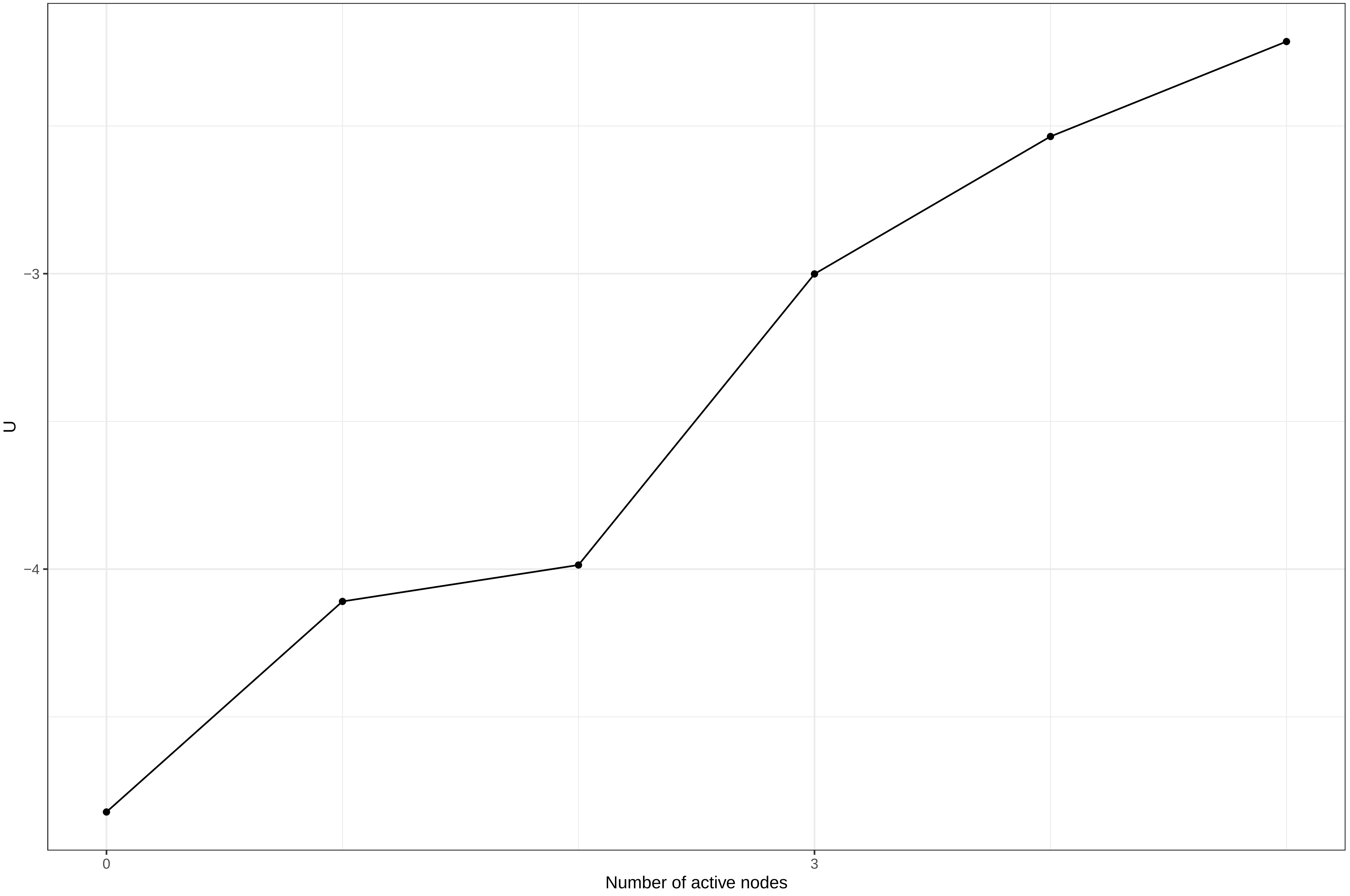

−3
−4
U
0
3
Number of active nodes

Network HMI−5 2016 mid support; n = 3052 / overall connectivity = 14.5603

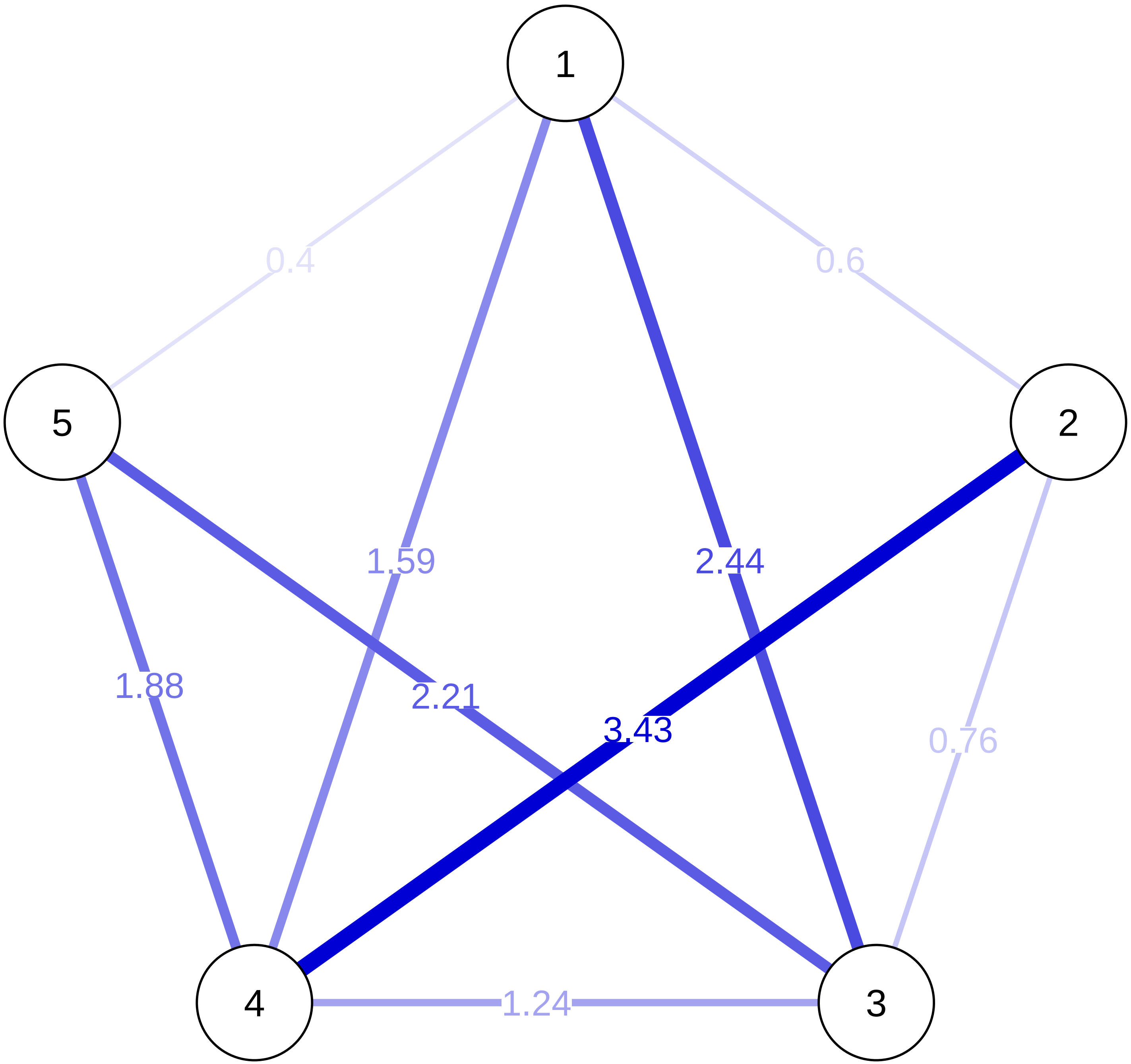

1: anxious; threshold = −4.7263
2: down; threshold = −4.5027
3: not calm; threshold = −2.4762
4: depressed; threshold = −5.2278
5: not happy; threshold = −2.174

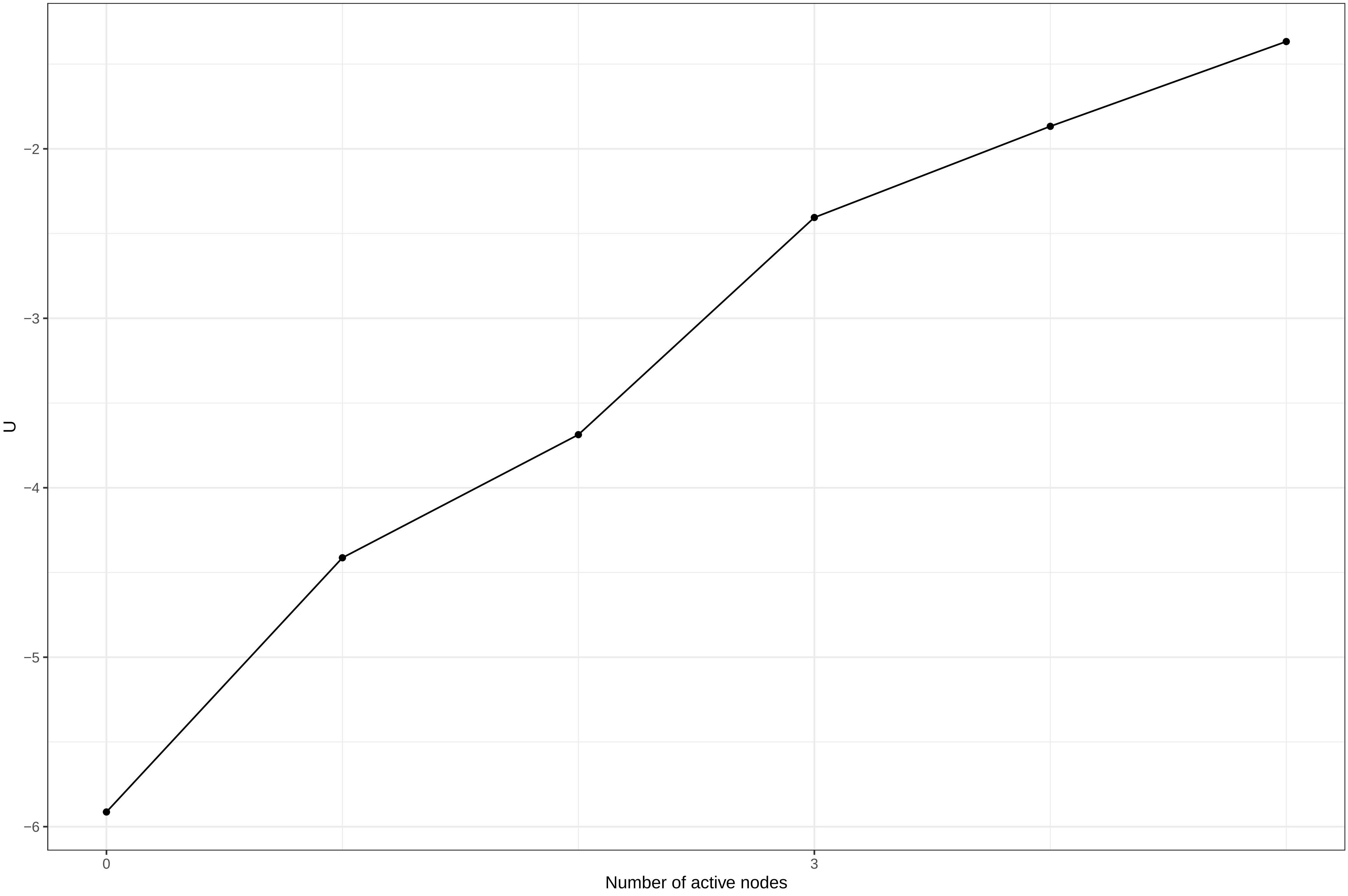

U
−2
−3
−4
−5
−6
0
3
Number of active nodes

Network HMI−5 2016 high support; n = 766 / overall connectivity = 15.6214

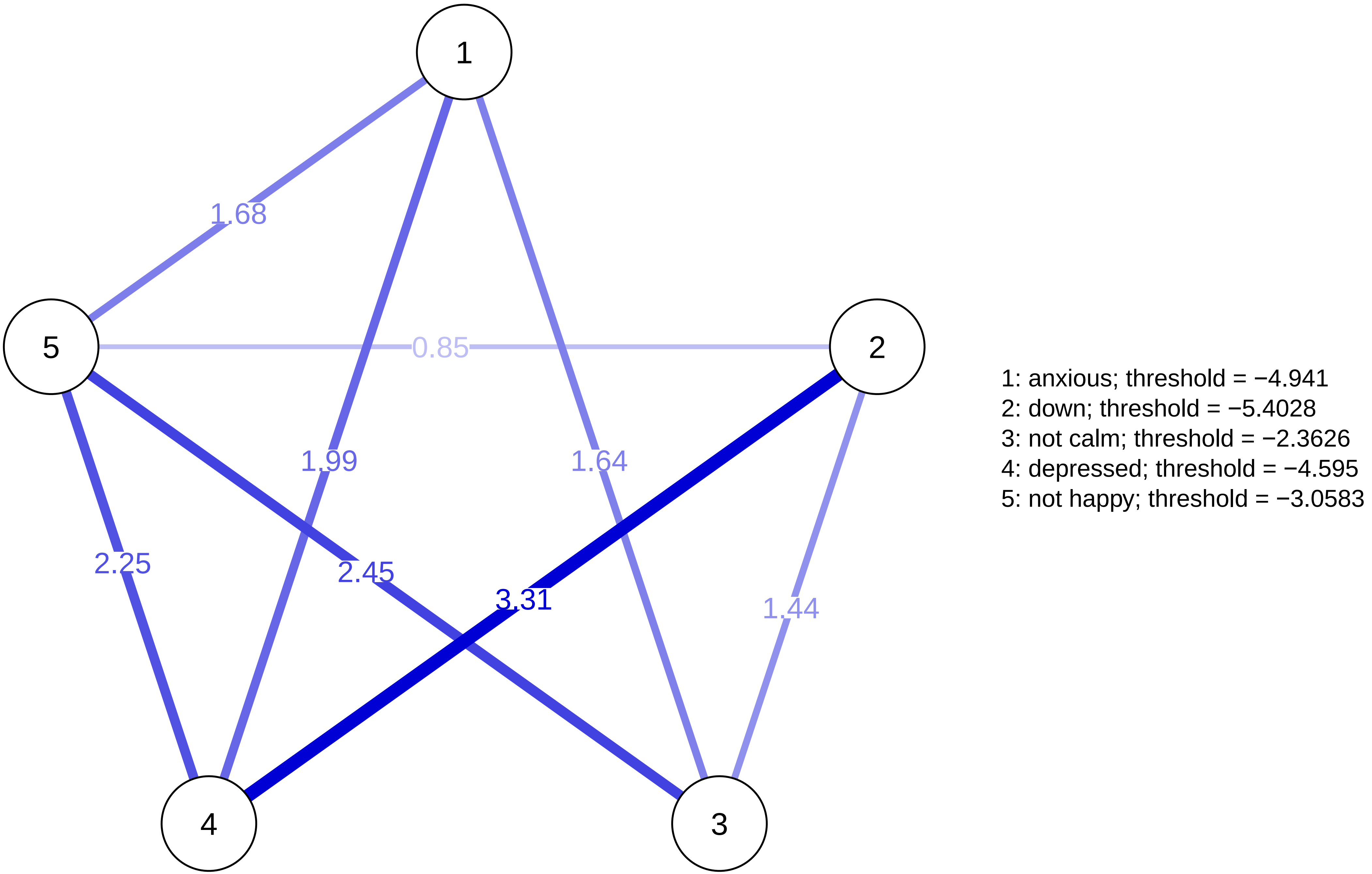

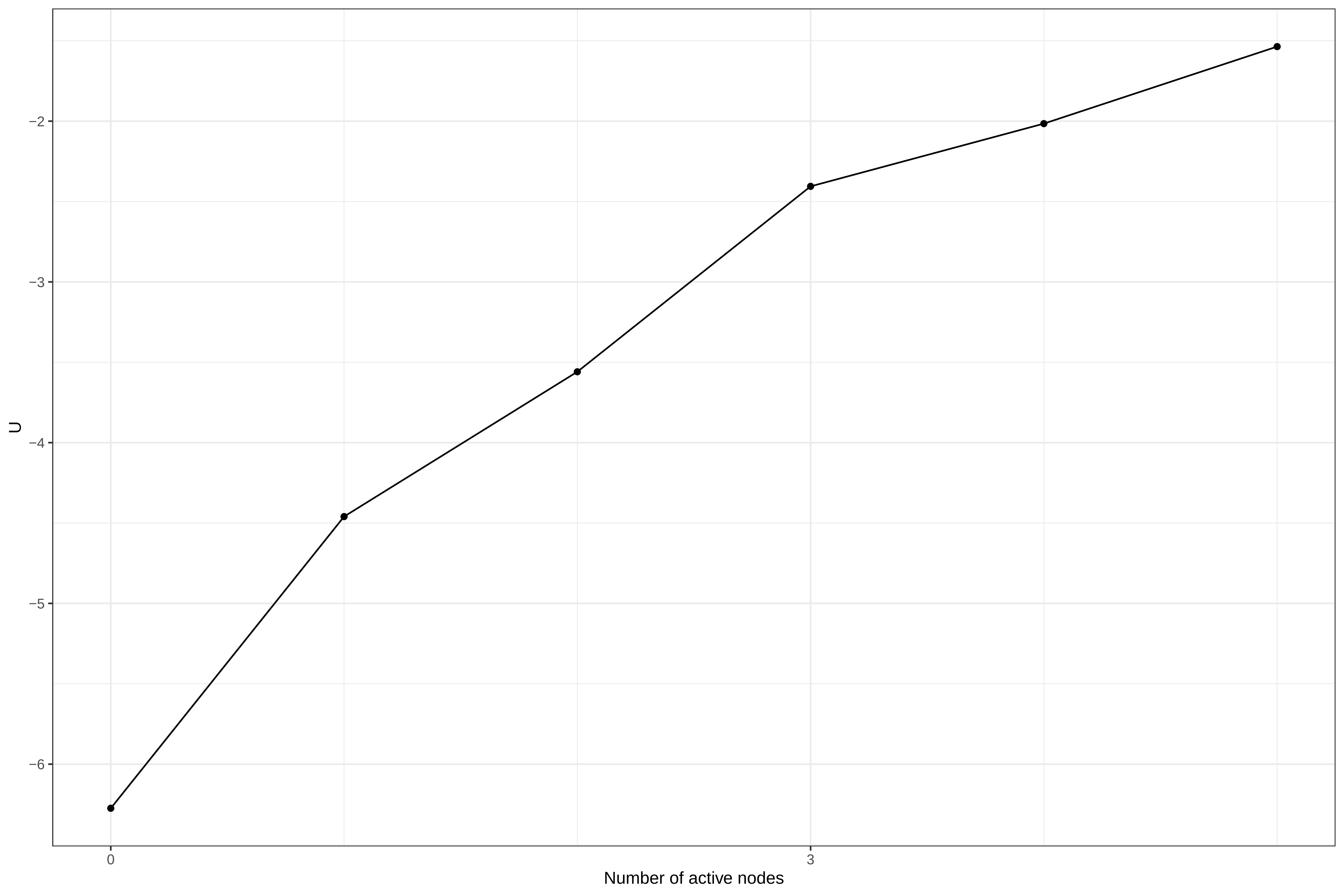

U
−2
−3
−4
−5
−6
0
3
Number of active nodes

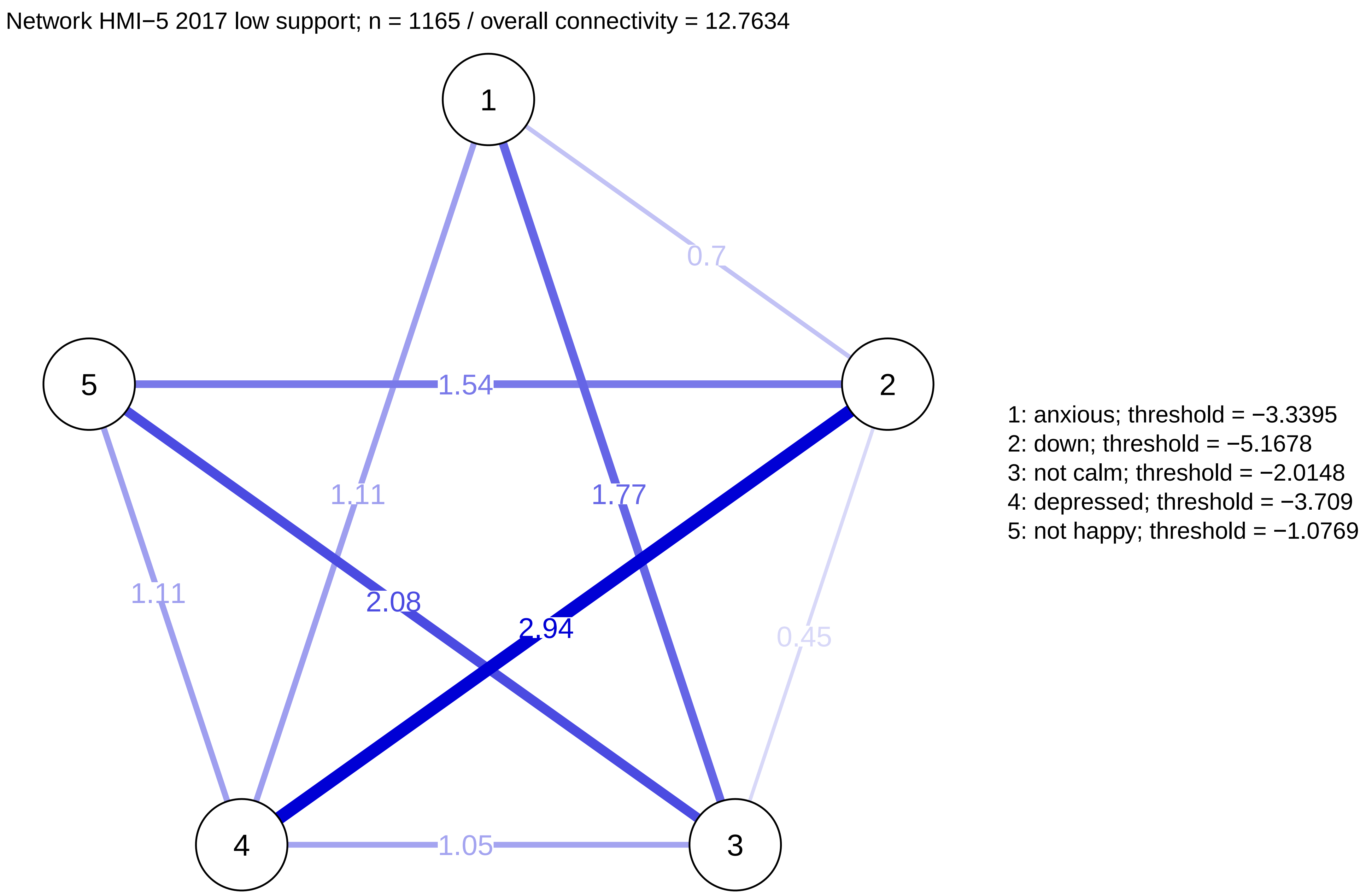
Network HMI−5 2017 low support; n = 1165 / overall connectivity = 12.7634
1
2
3
4
5
0.7
1.54
1.11
1.77
1.11
2.08
2.94
0.45
1.05
1: anxious; threshold = −3.3395
2: down; threshold = −5.1678
3: not calm; threshold = −2.0148
4: depressed; threshold = −3.709
5: not happy; threshold = −1.0769

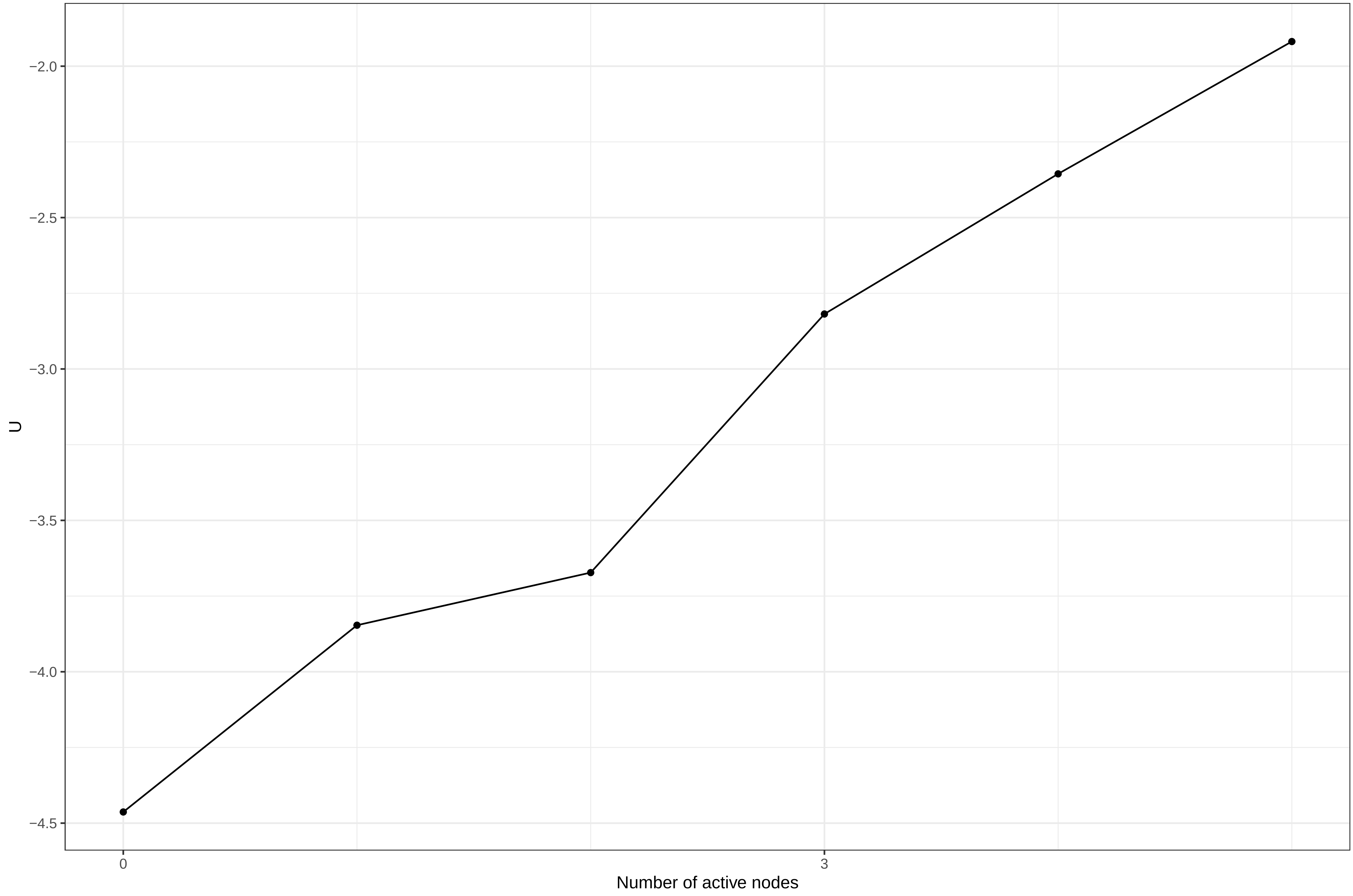

−2.0
−2.5
−3.0
−3.5
−4.0
−4.5
U
0
3
Number of active nodes

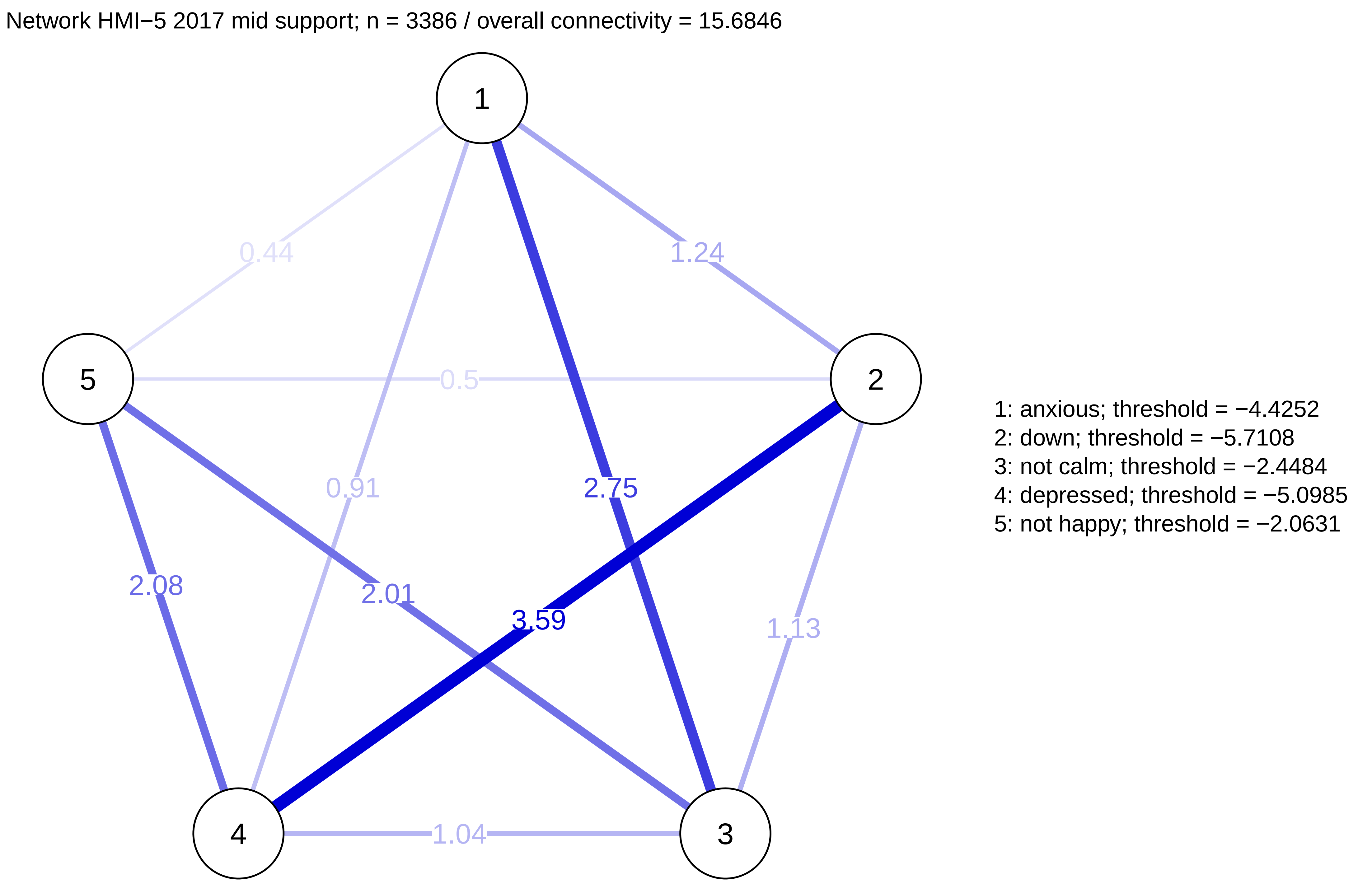
Network HMI−5 2017 mid support; n = 3386 / overall connectivity = 15.6846
1
2
3
4
5
0.44
1.24
0.5
0.91
2.75
2.08
2.01
3.59
1.13
1.04
1: anxious; threshold = −4.4252
2: down; threshold = −5.7108
3: not calm; threshold = −2.4484
4: depressed; threshold = −5.0985
5: not happy; threshold = −2.0631

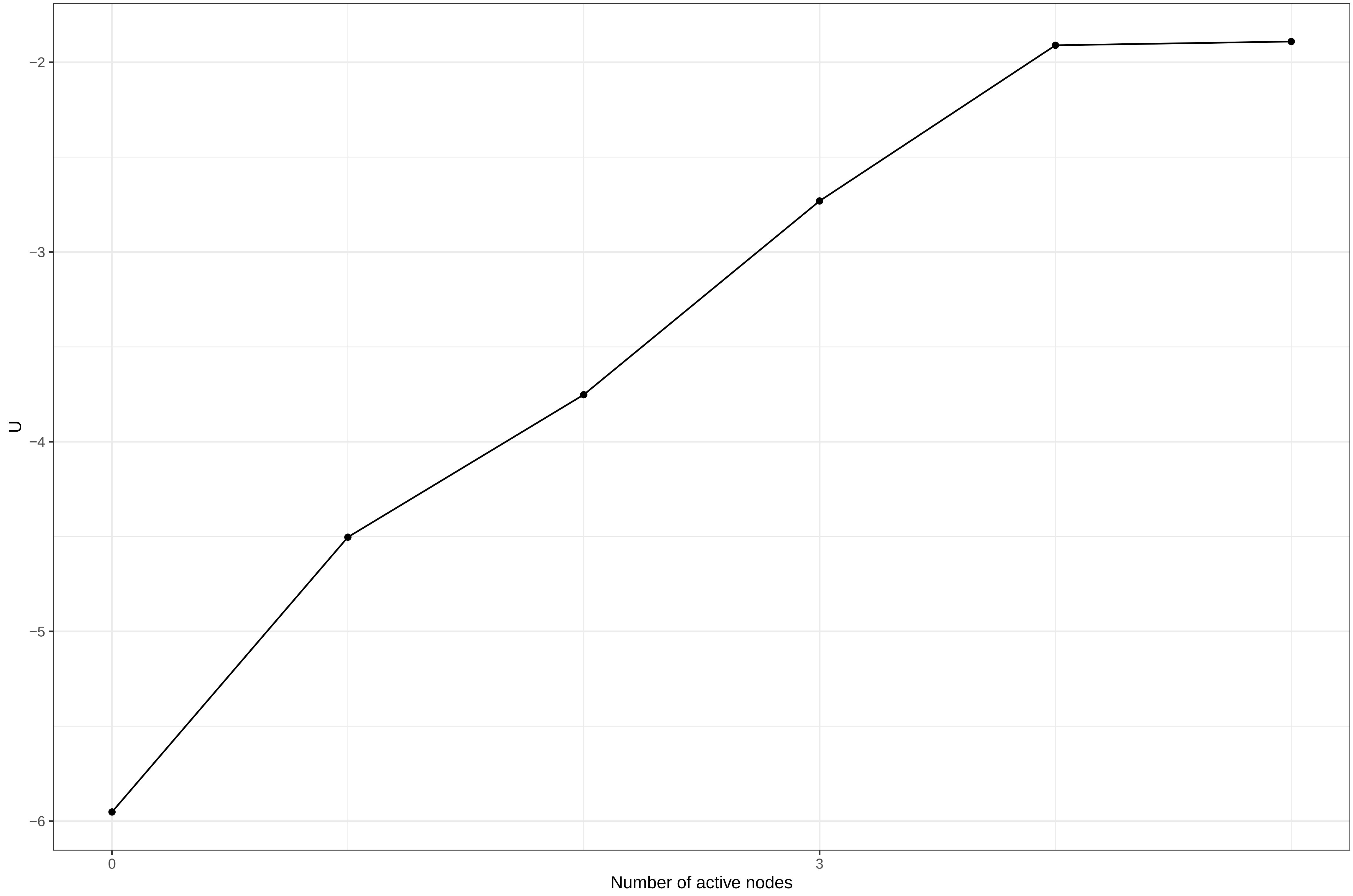

−2
−3
−4
−5
−6
U
0
3
Number of active nodes

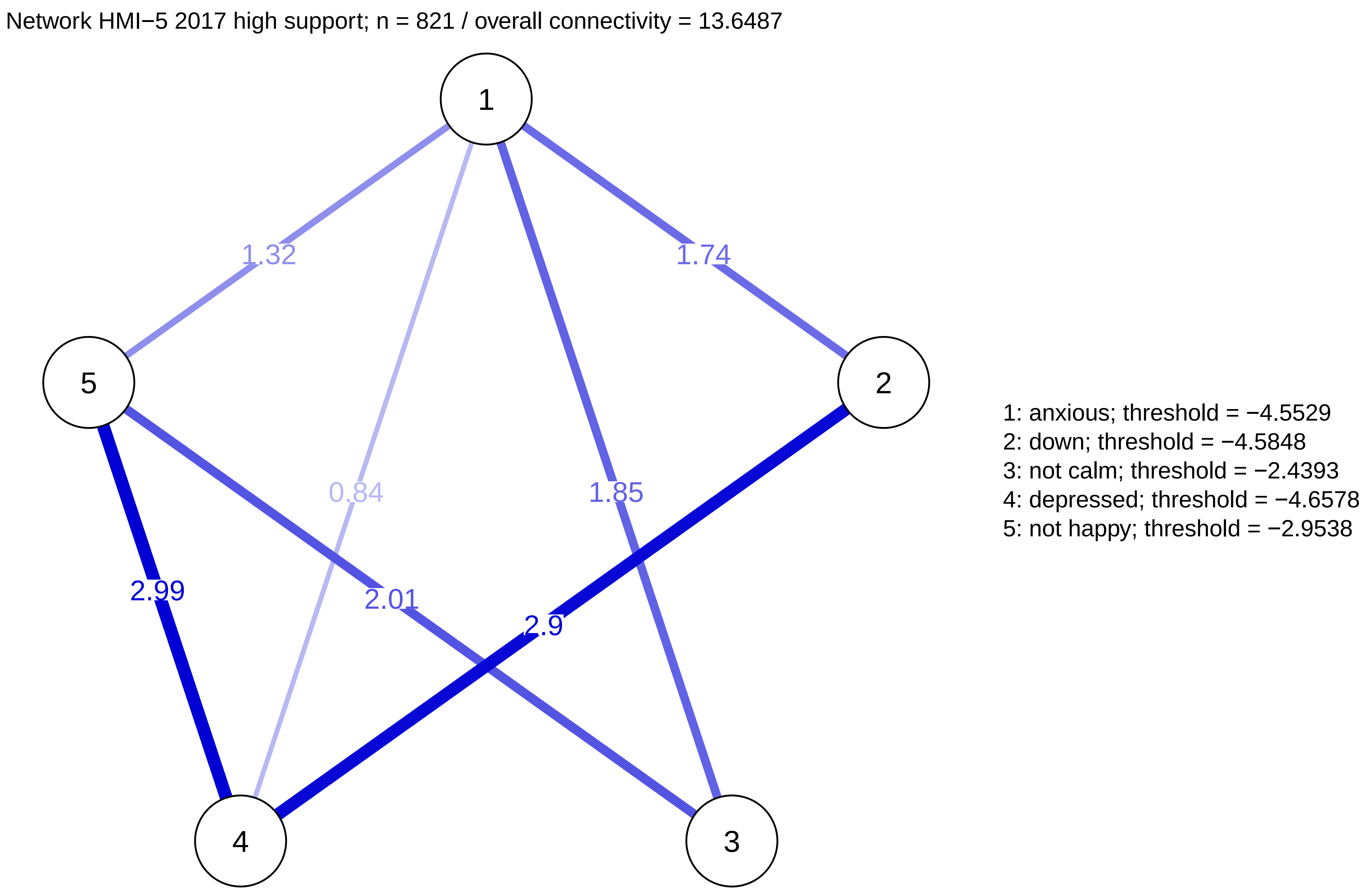
Network HMI−5 2017 high support; n = 821 / overall connectivity = 13.6487
1
2
3
4
5
1.32
1.74
0.84
1.85
2.99
2.01
2.9
1: anxious; threshold = −4.5529
2: down; threshold = −4.5848
3: not calm; threshold = −2.4393
4: depressed; threshold = −4.6578
5: not happy; threshold = −2.9538

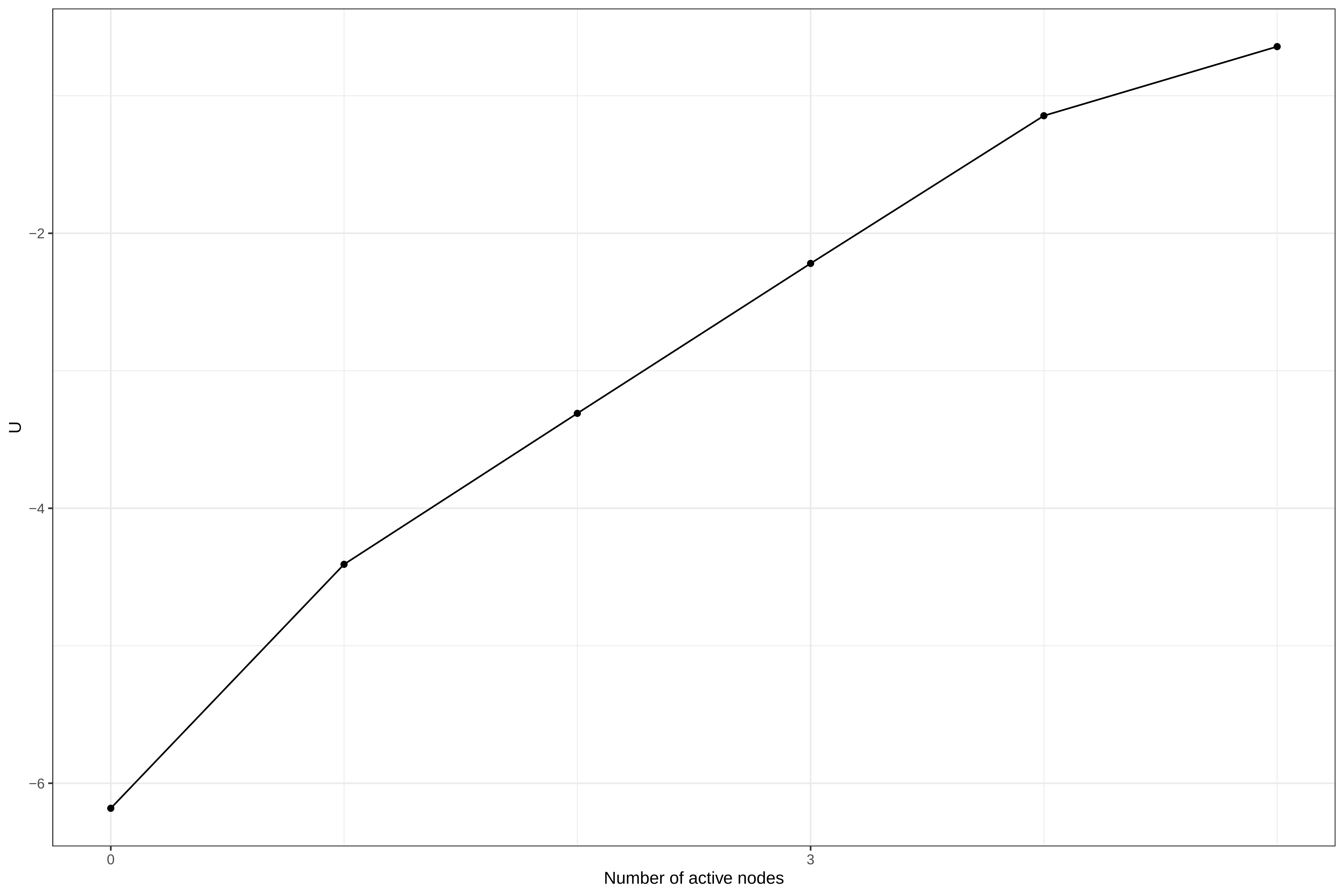

−2
−4
−6
U
0
3
Number of active nodes

Network HMI−5 2018 low support; n = 1097 / overall connectivity = 13.1219

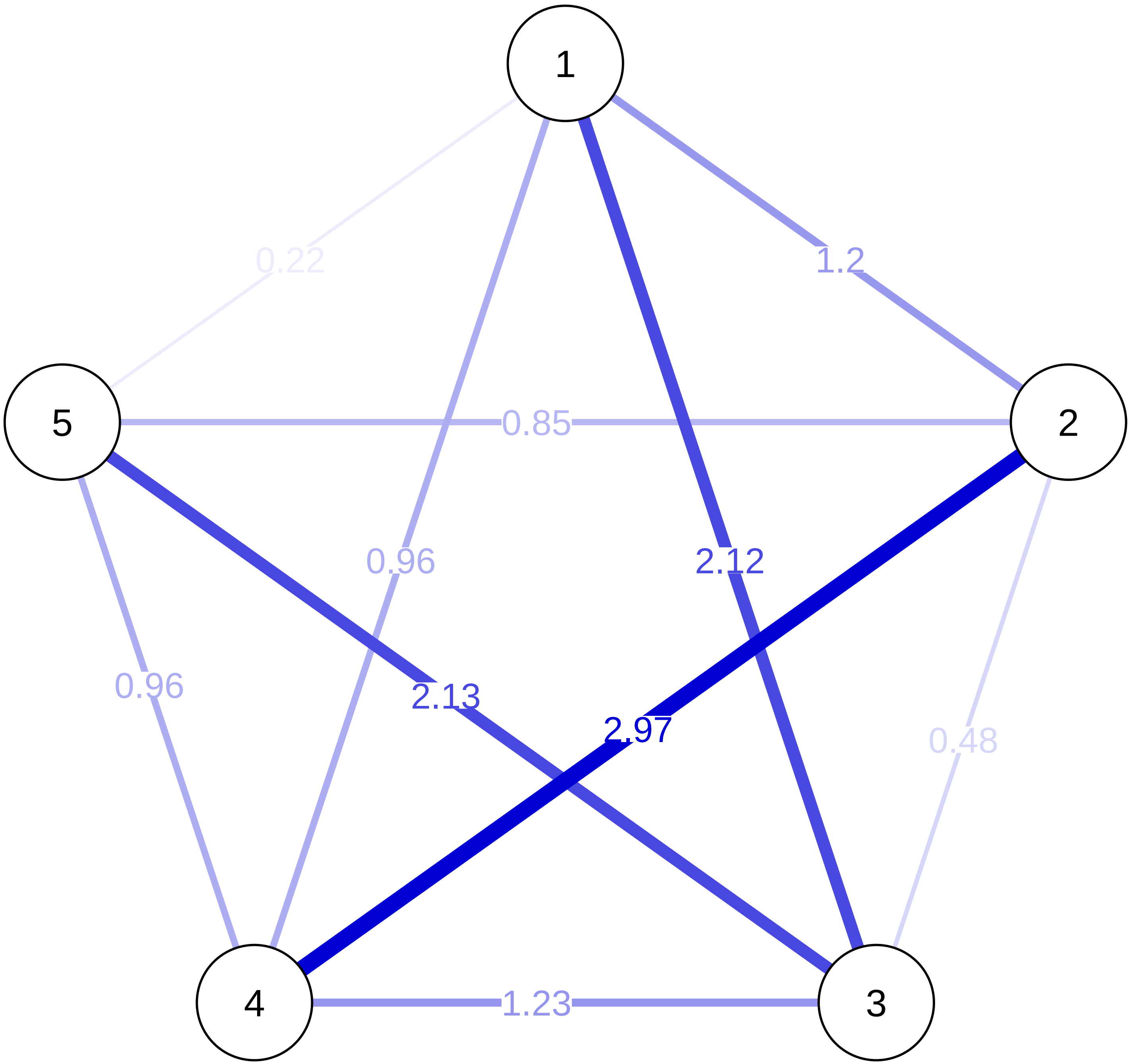

1: anxious; threshold = −3.6425
2: down; threshold = −4.7581
3: not calm; threshold = −2.0392
4: depressed; threshold = −3.7659
5: not happy; threshold = −1.1501

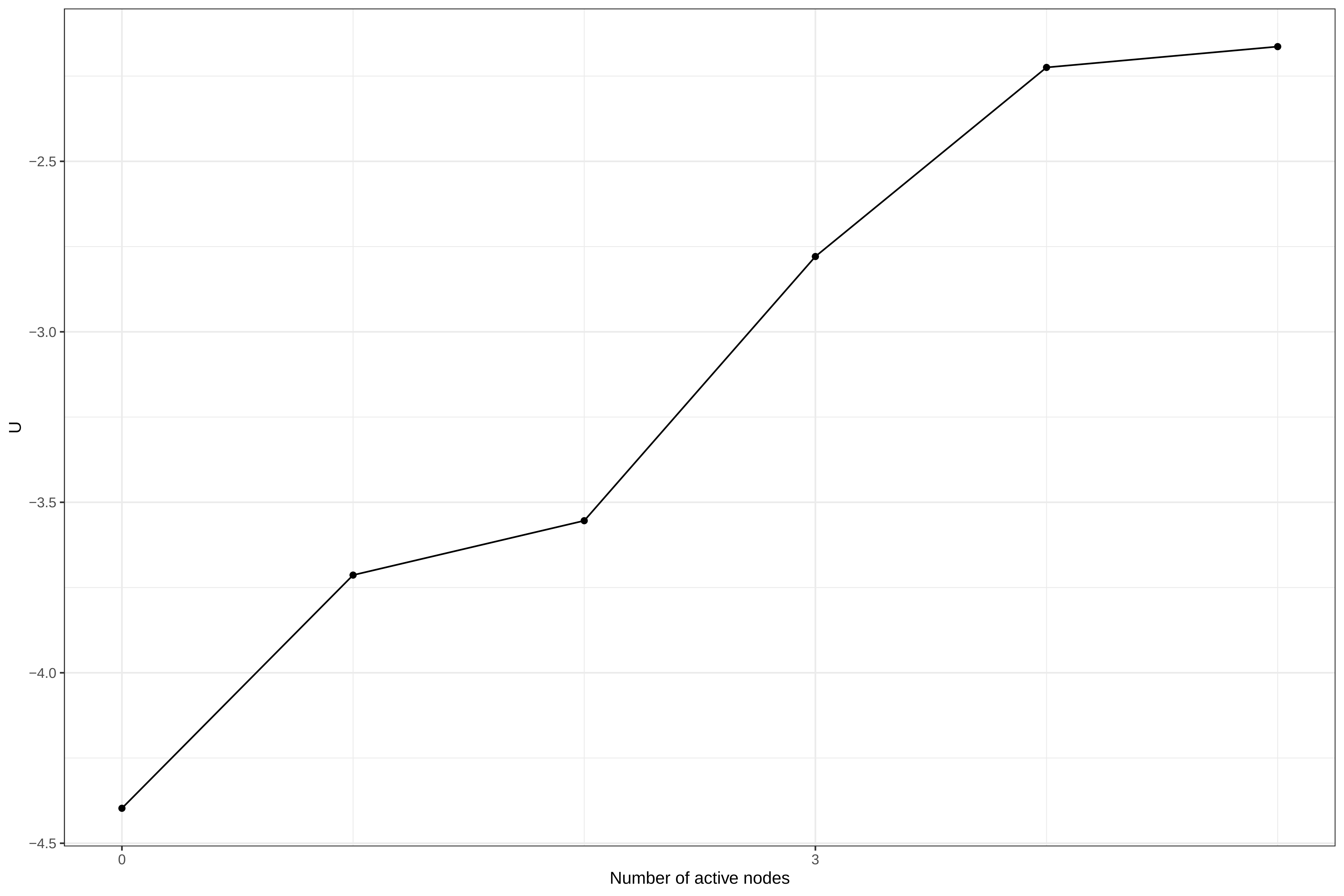

−2.5
−3.0
−3.5
−4.0
−4.5
U
0
3
Number of active nodes

Network HMI−5 2018 mid support; n = 3115 / overall connectivity = 15.6773

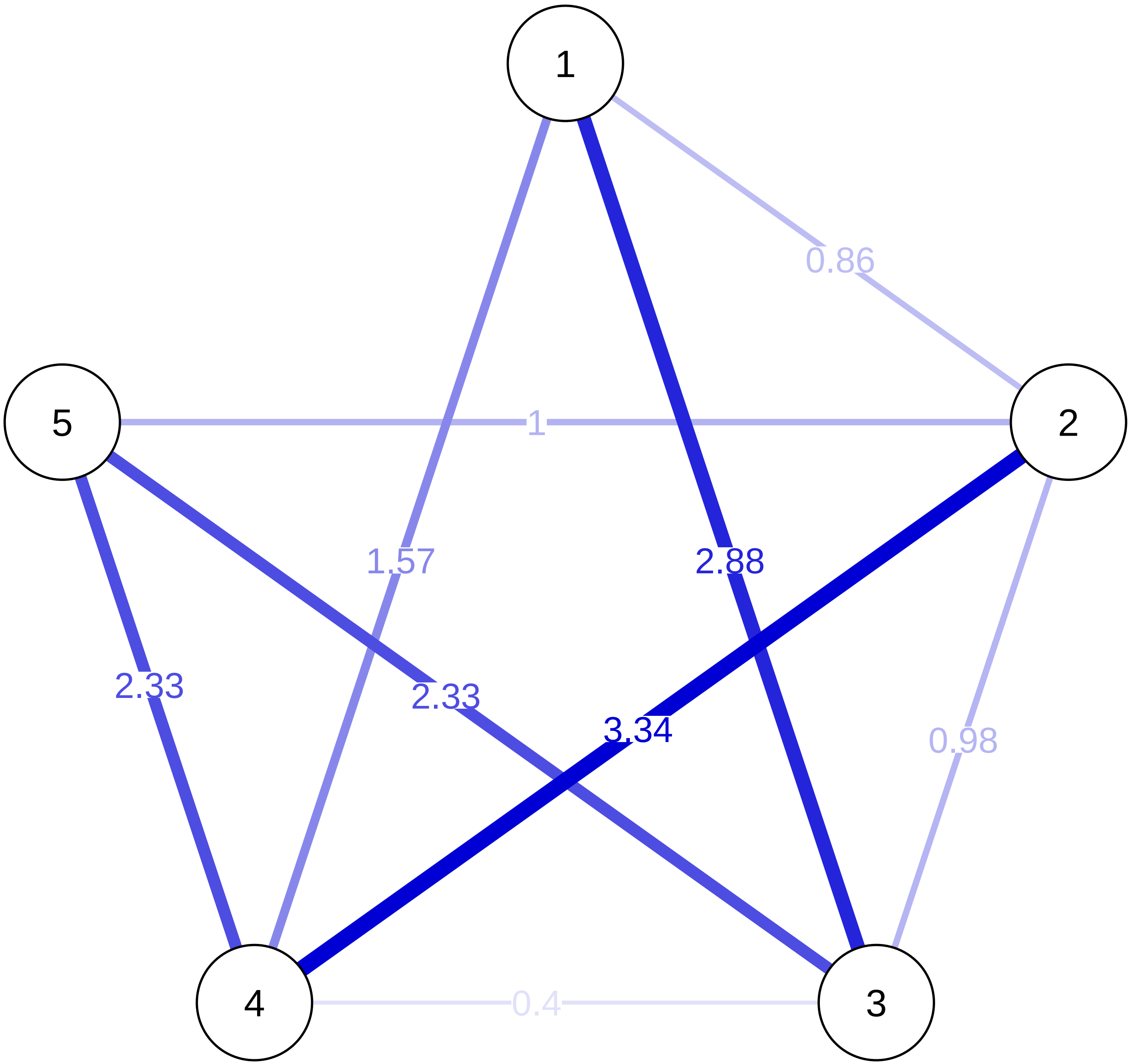

1: anxious; threshold = −4.4478
2: down; threshold = −5.8116
3: not calm; threshold = −2.6206
4: depressed; threshold = −5.0826
5: not happy; threshold = −2.2702

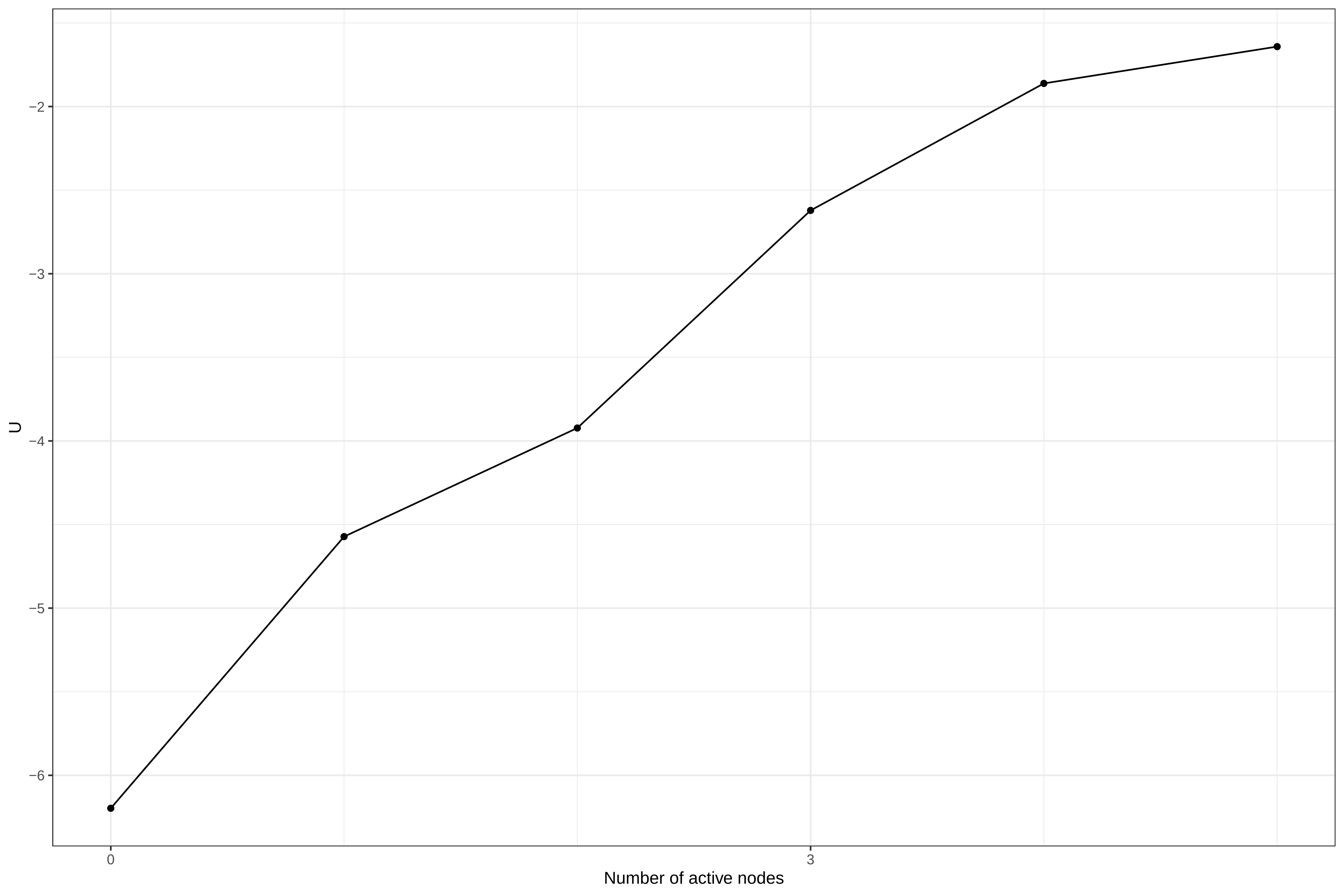

U
−2
−3
−4
−5
−6
0
3
Number of active nodes

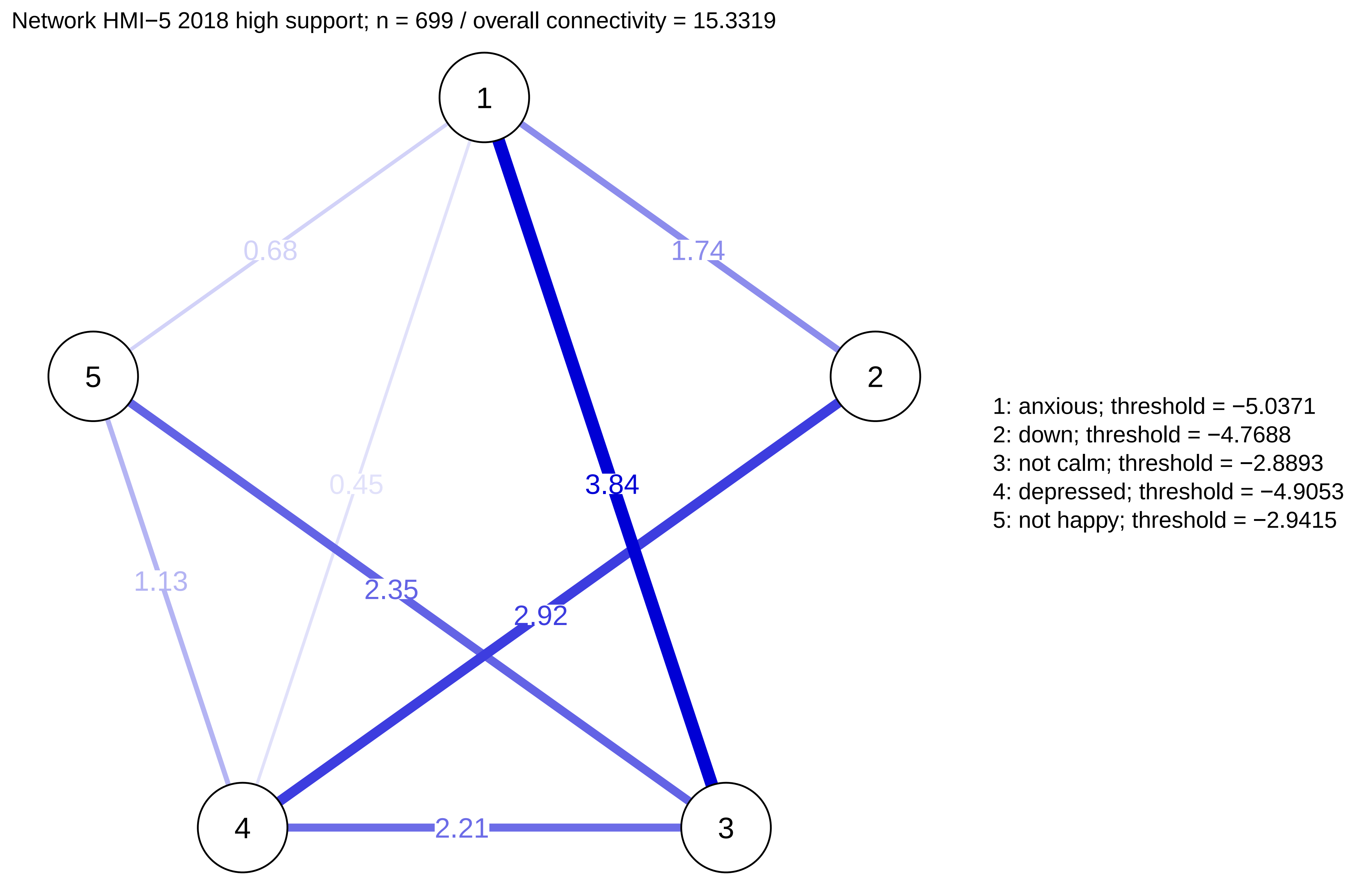
Network HMI−5 2018 high support; n = 699 / overall connectivity = 15.3319
1
2
3
4
5
0.68
1.74
0.45
3.84
1.13
2.35
2.92
2.21
1: anxious; threshold = −5.0371
2: down; threshold = −4.7688
3: not calm; threshold = −2.8893
4: depressed; threshold = −4.9053
5: not happy; threshold = −2.9415

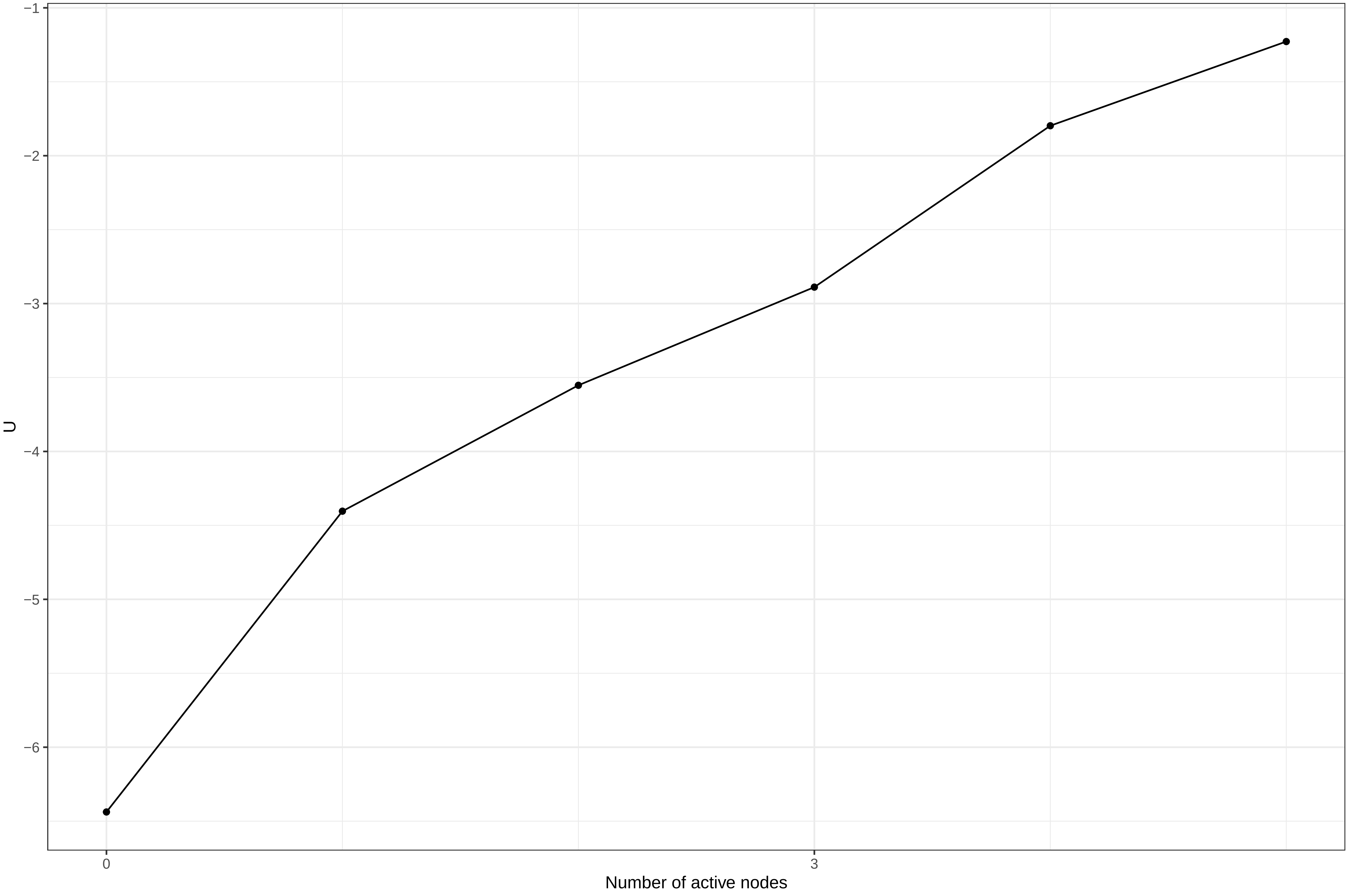

−1
−2
−3
−4
−5
−6
U
0
3
Number of active nodes

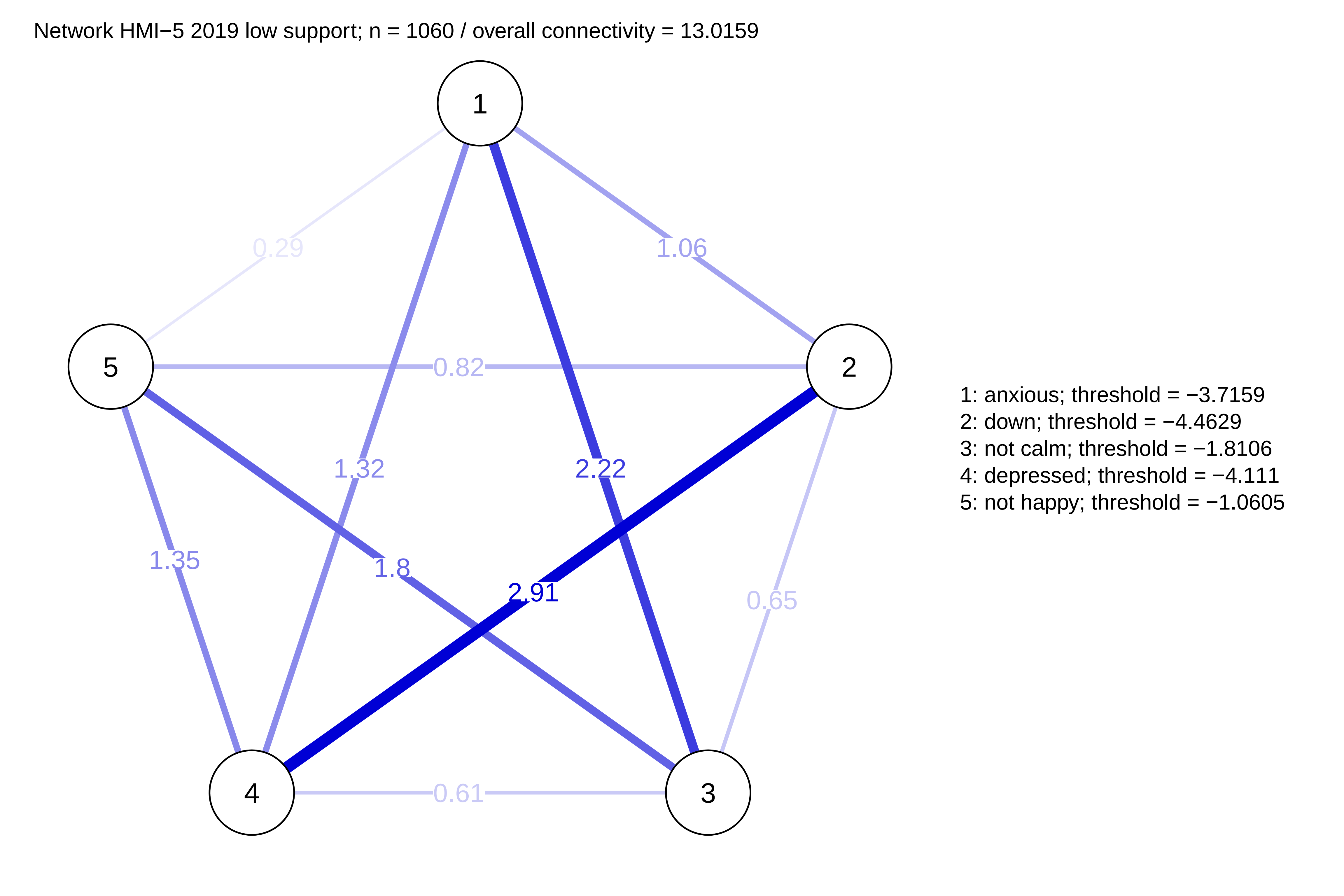
Network HMI−5 2019 low support; n = 1060 / overall connectivity = 13.0159
1
2
3
4
5
0.29
1.06
0.82
1.32
2.22
1.35
1.8
2.91
0.65
0.61
1: anxious; threshold = −3.7159
2: down; threshold = −4.4629
3: not calm; threshold = −1.8106
4: depressed; threshold = −4.111
5: not happy; threshold = −1.0605

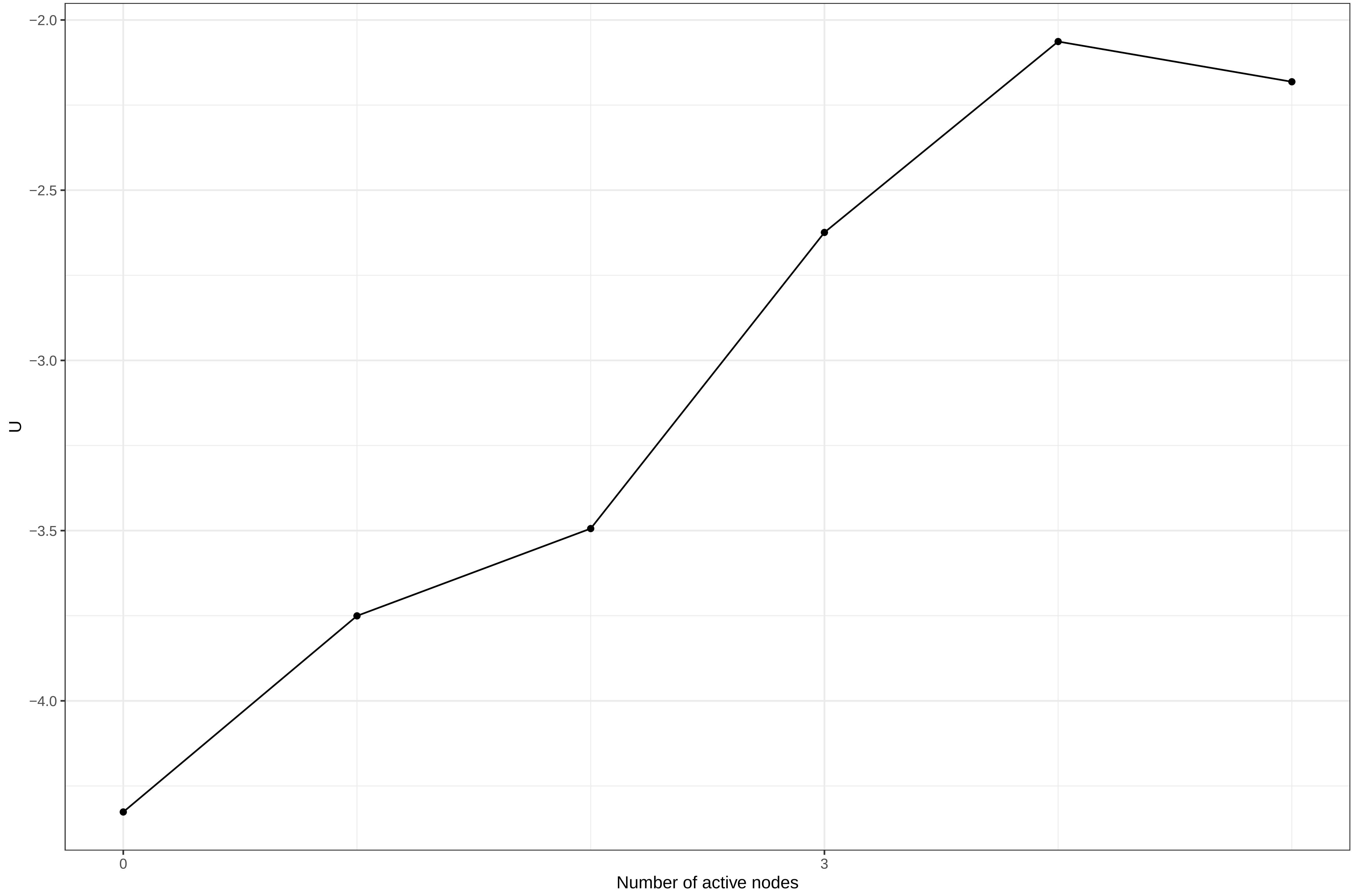

−2.0
−2.5
−3.0
−3.5
−4.0
U
0
3
Number of active nodes

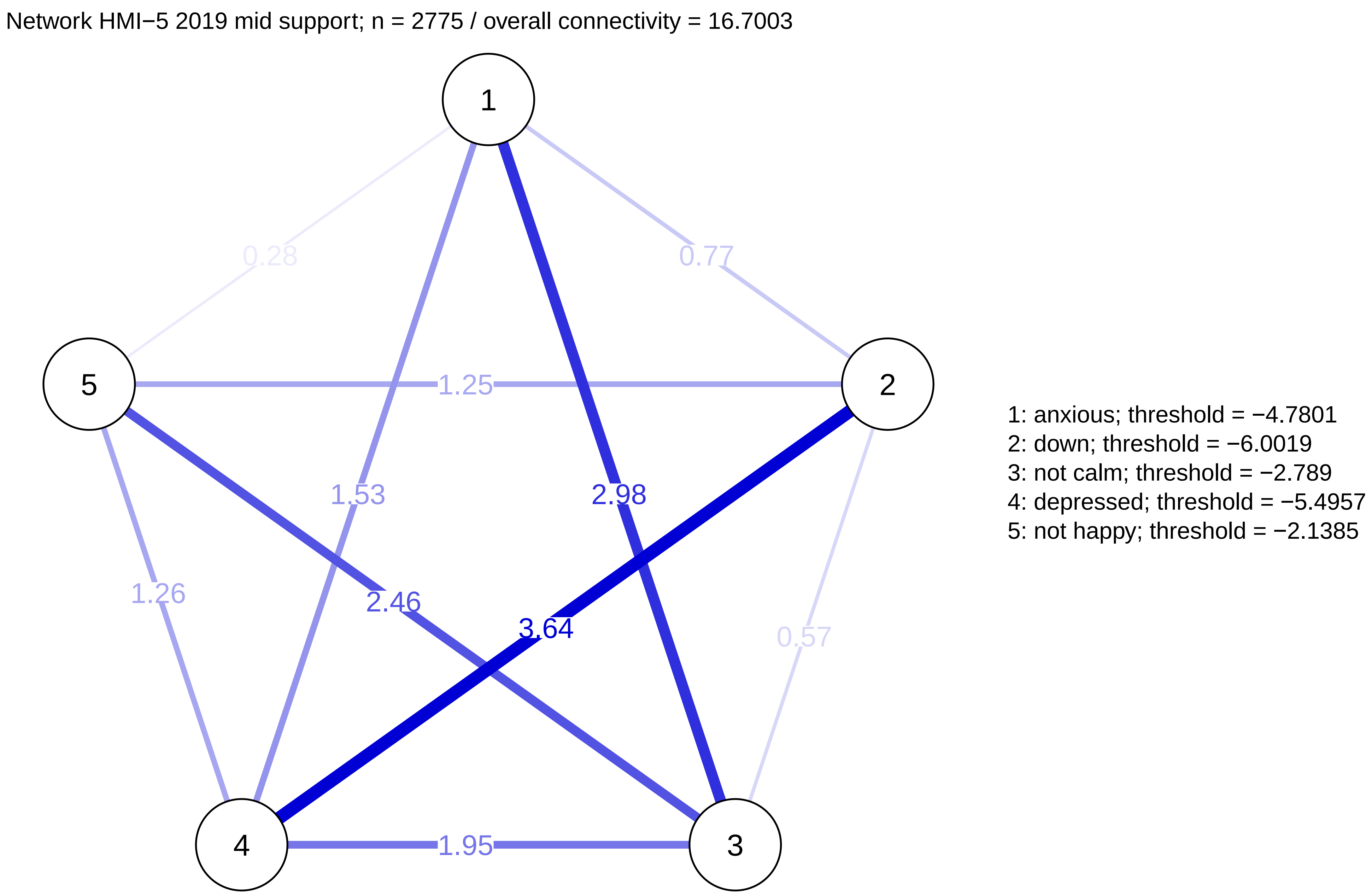
Network HMI−5 2019 mid support; n = 2775 / overall connectivity = 16.7003
1
2
3
4
5
0.28
0.77
1.25
1.53
2.98
1.26
2.46
3.64
0.57
1.95
1: anxious; threshold = −4.7801
2: down; threshold = −6.0019
3: not calm; threshold = −2.789
4: depressed; threshold = −5.4957
5: not happy; threshold = −2.1385

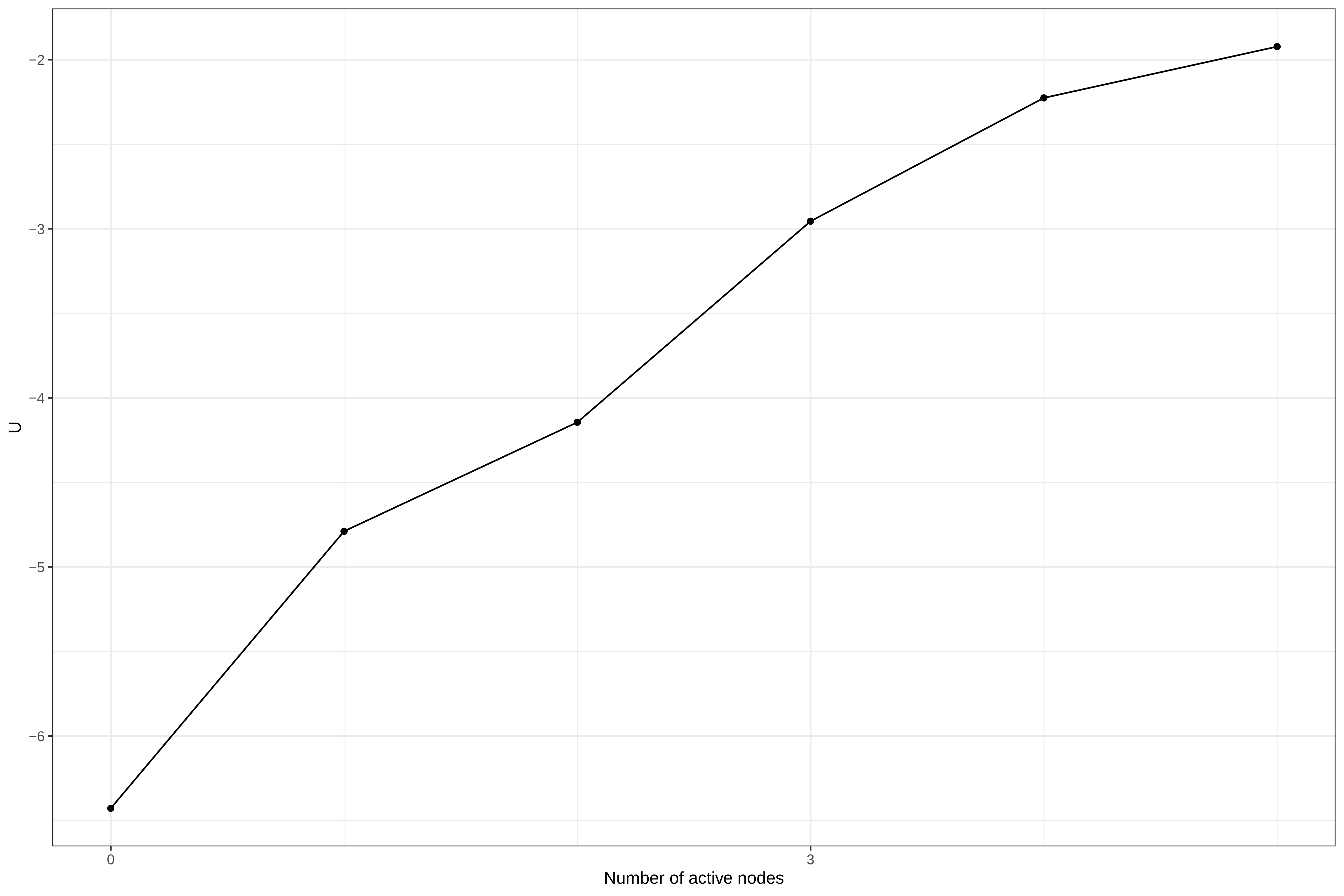

−2
−3
−4
−5
−6
U
0
3
Number of active nodes

Network HMI−5 2019 high support; n = 679 / overall connectivity = 17.0375

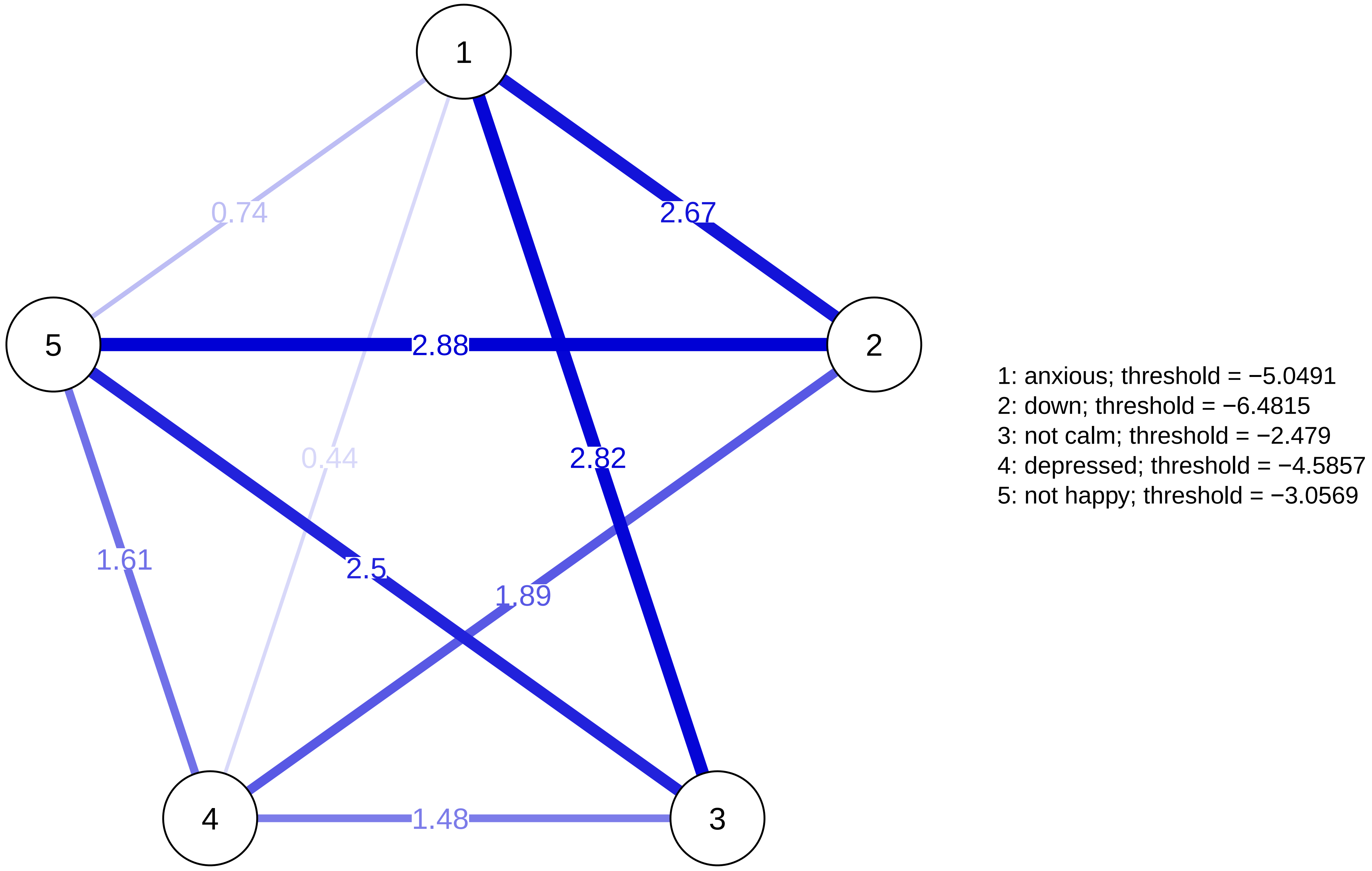

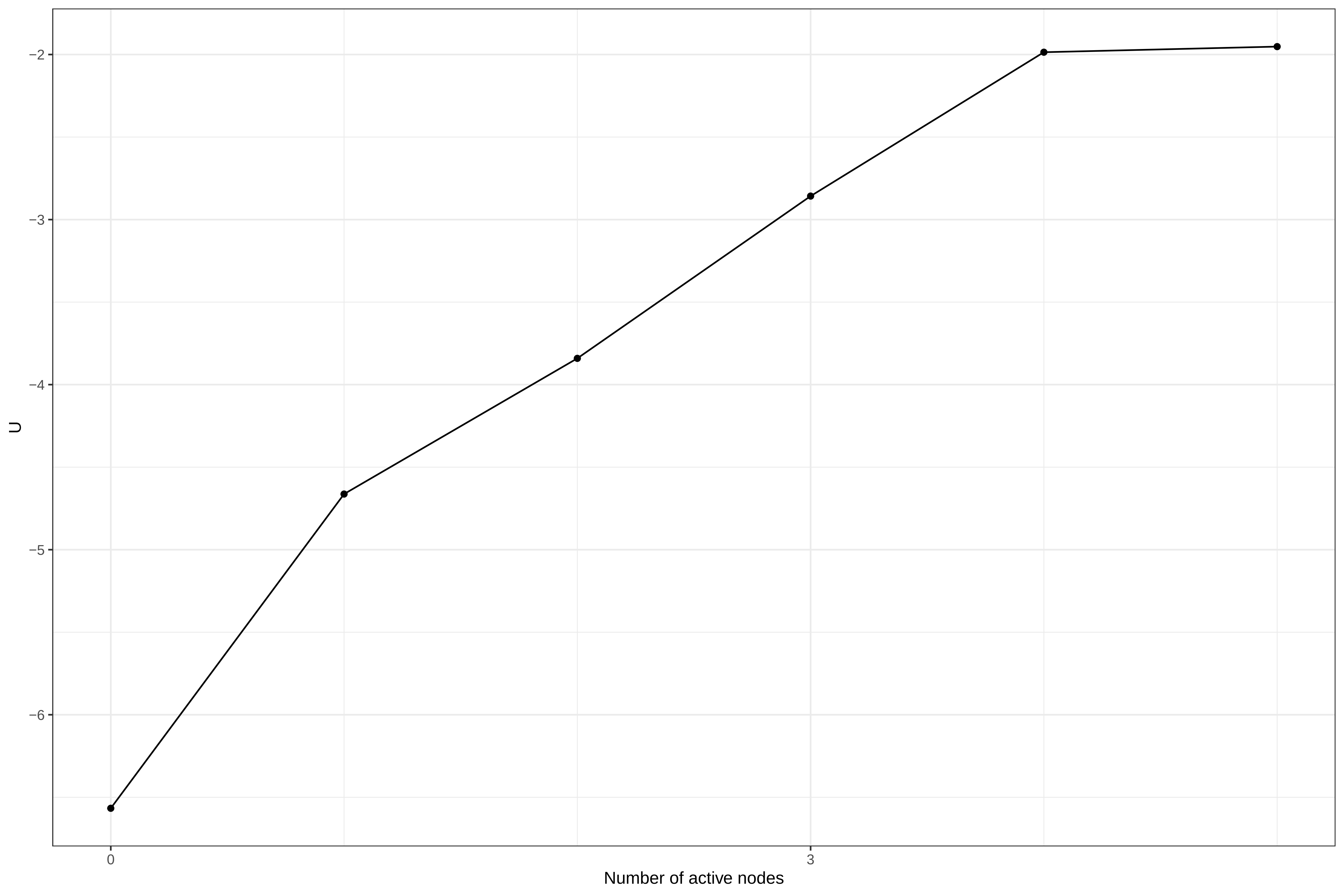

−2
−3
−4
−5
−6
U
0
3
Number of active nodes

Network HMI−5 2020 low support; n = 785 / overall connectivity = 11.8679

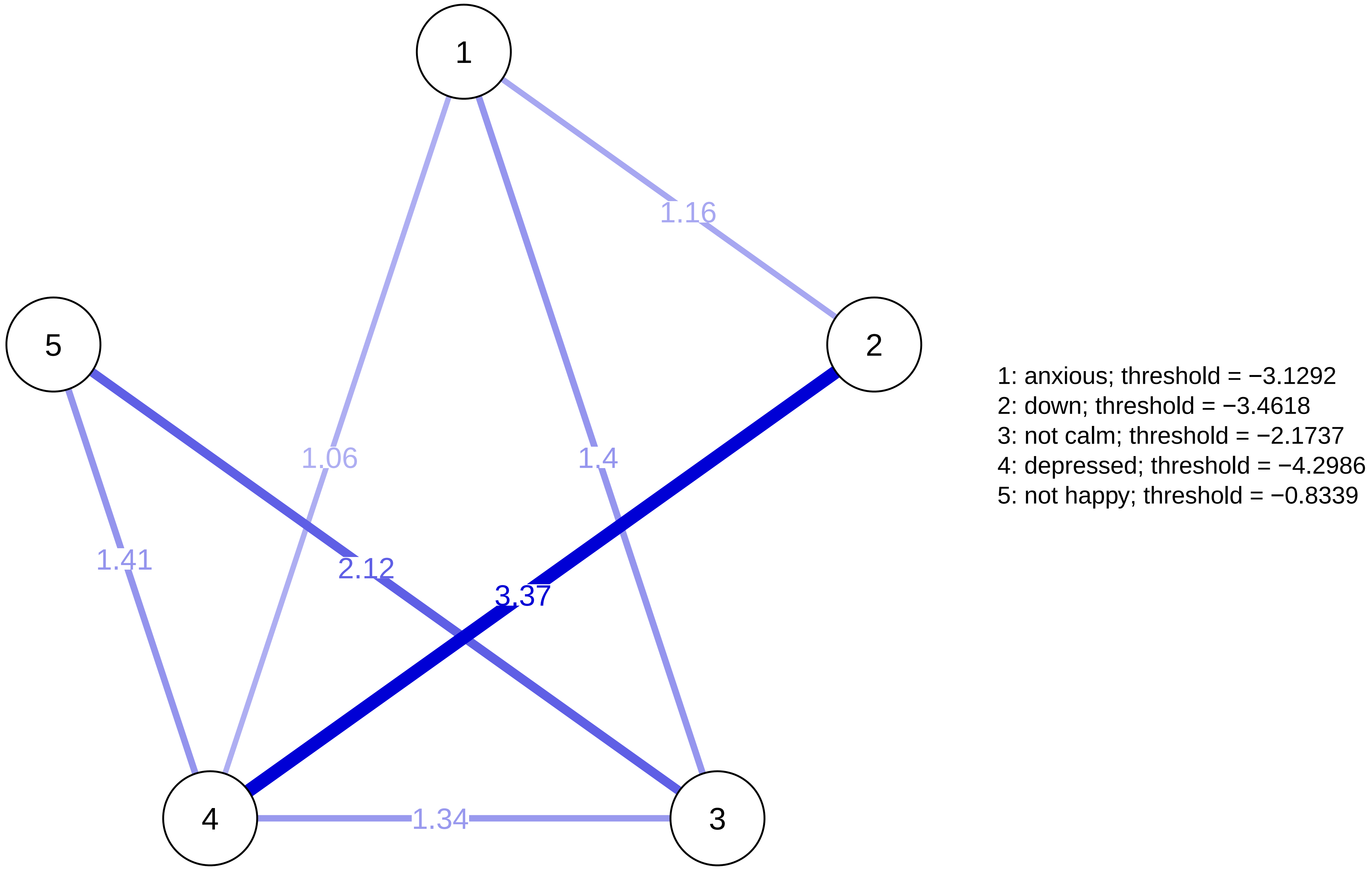

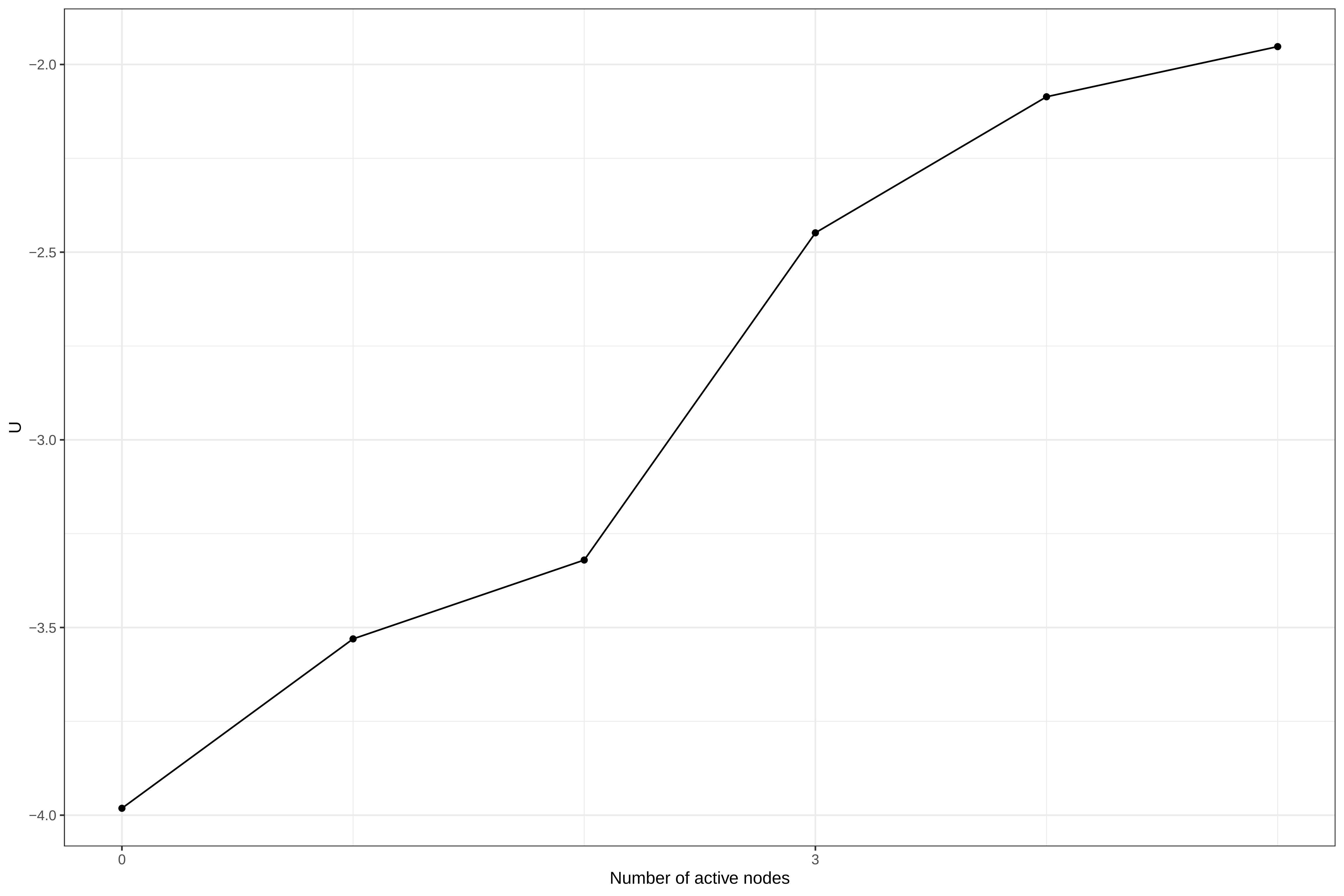

−2.0
−2.5
−3.0
−3.5
−4.0
U
0
3
Number of active nodes

Network HMI−5 2020 mid support; n = 3634 / overall connectivity = 15.477

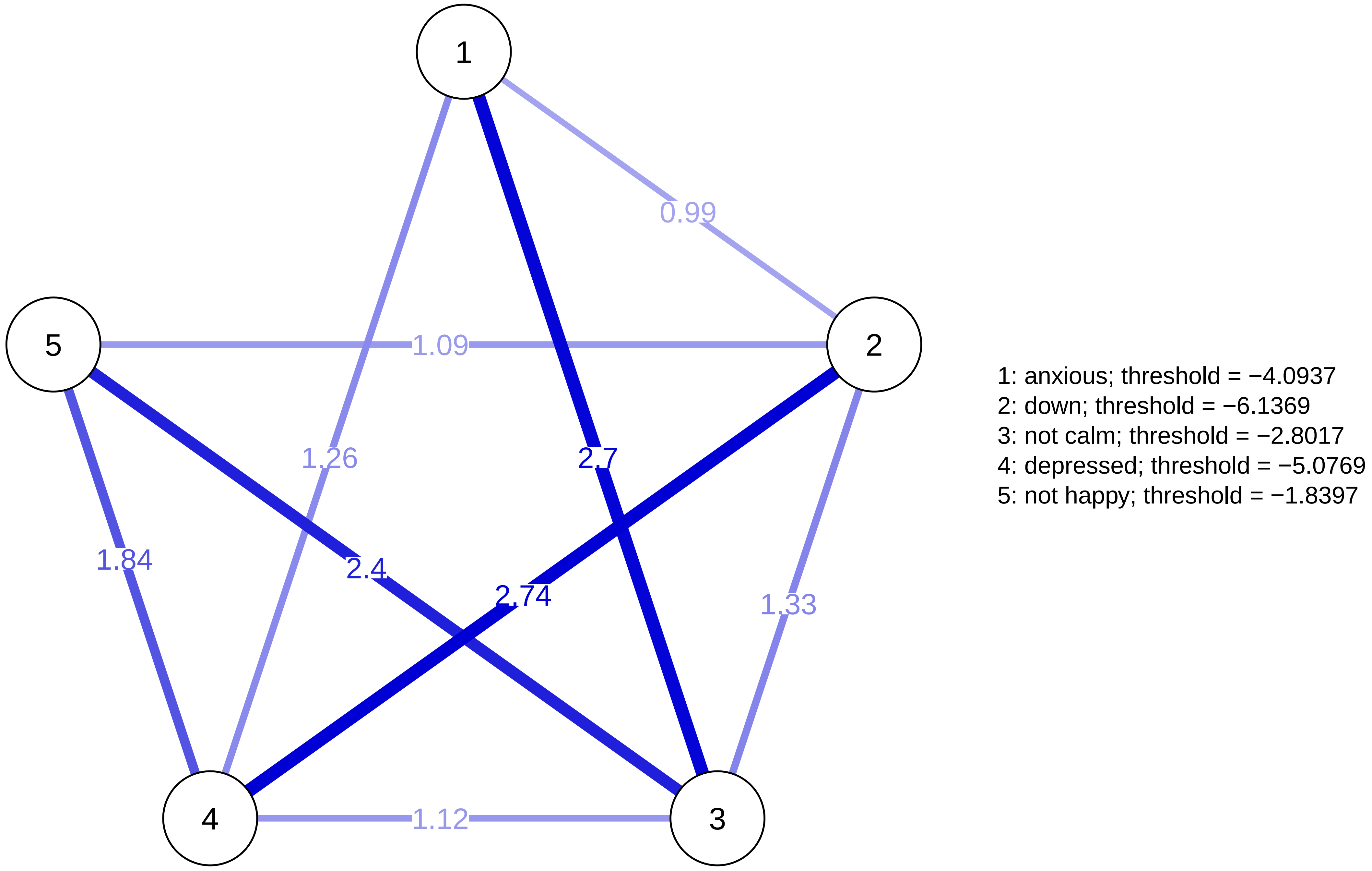

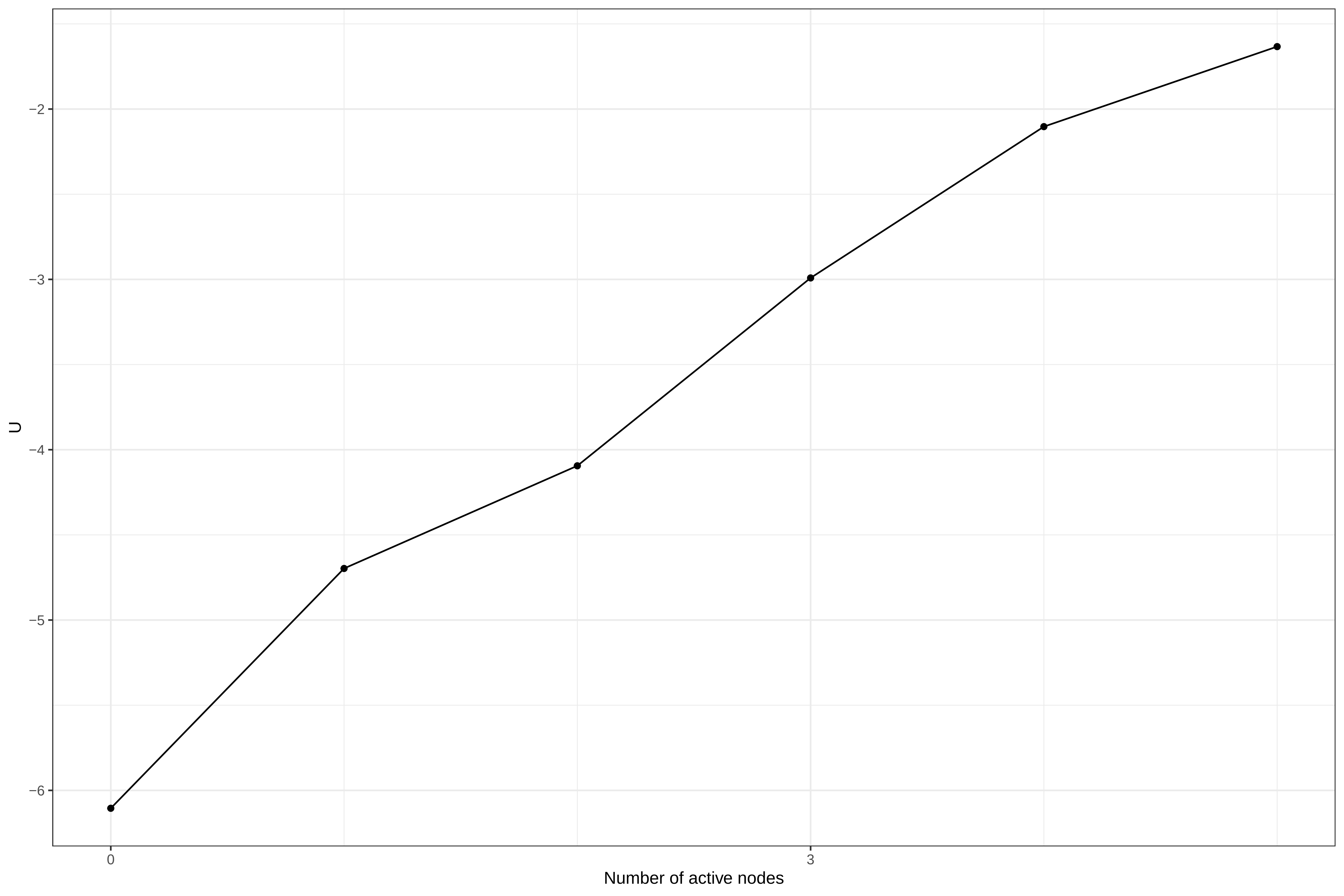

−2
−3
−4
−5
−6
U
0
3
Number of active nodes

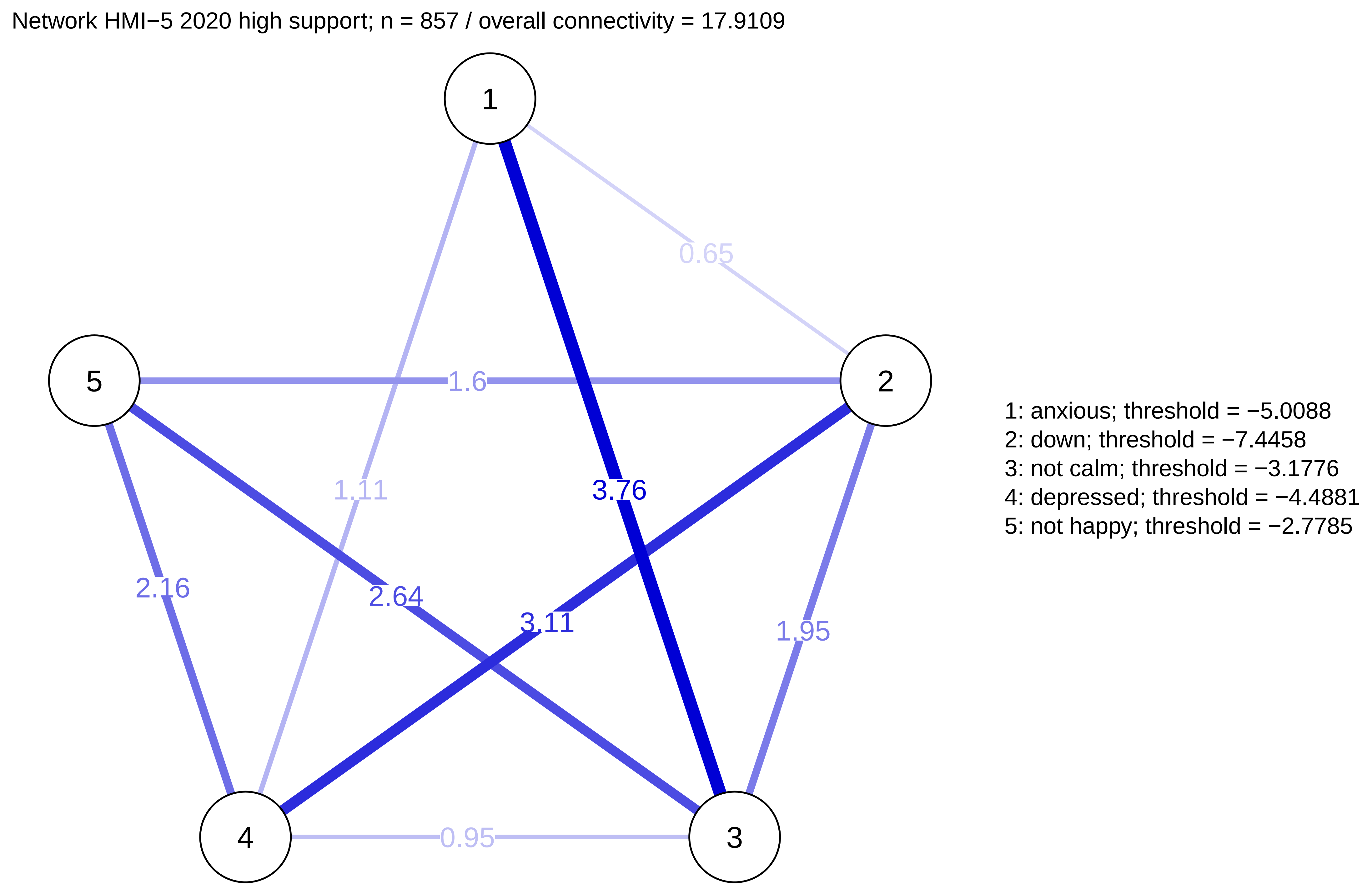
Network HMI−5 2020 high support; n = 857 / overall connectivity = 17.9109
1
2
3
4
5
0.65
1.6
1.11
3.76
2.16
2.64
3.11
1.95
0.95
1: anxious; threshold = −5.0088
2: down; threshold = −7.4458
3: not calm; threshold = −3.1776
4: depressed; threshold = −4.4881
5: not happy; threshold = −2.7785

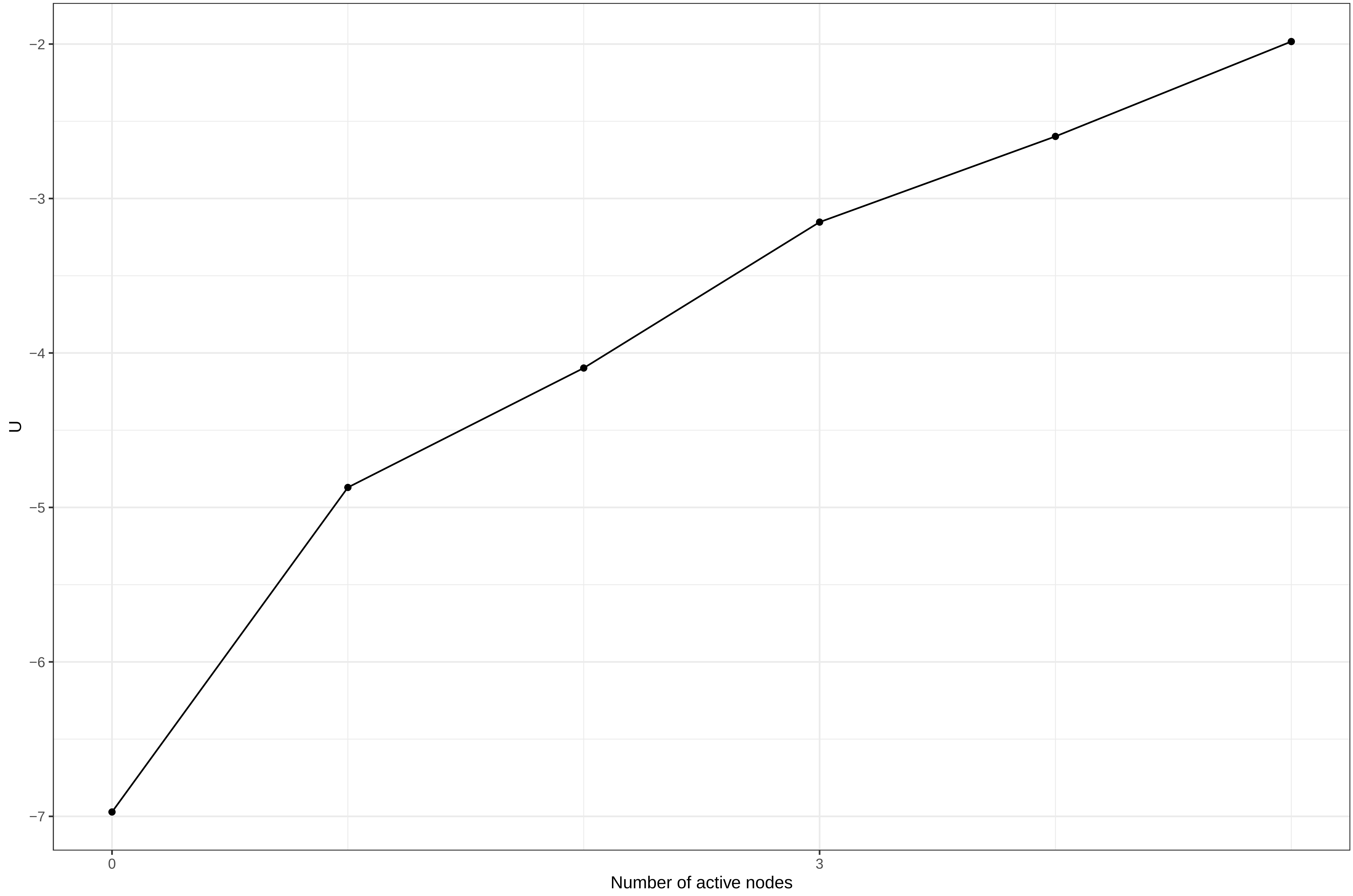

−2
−3
−4
−5
−6
−7
U
0
3
Number of active nodes

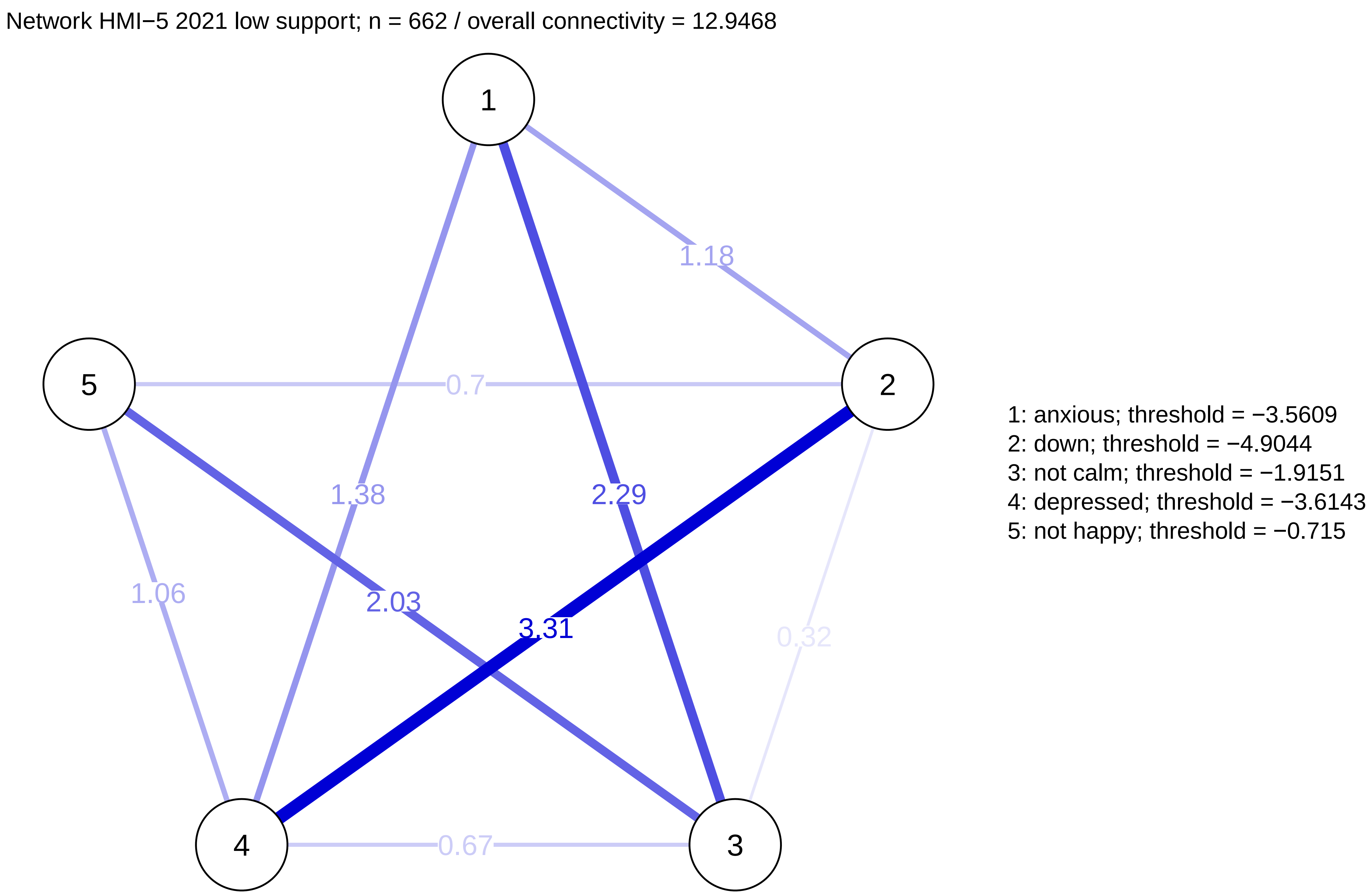
Network HMI−5 2021 low support; n = 662 / overall connectivity = 12.9468
1
2
3
4
5
1.18
0.7
1.38
2.29
1.06
2.03
3.31
0.32
0.67
1: anxious; threshold = −3.5609
2: down; threshold = −4.9044
3: not calm; threshold = −1.9151
4: depressed; threshold = −3.6143
5: not happy; threshold = −0.715

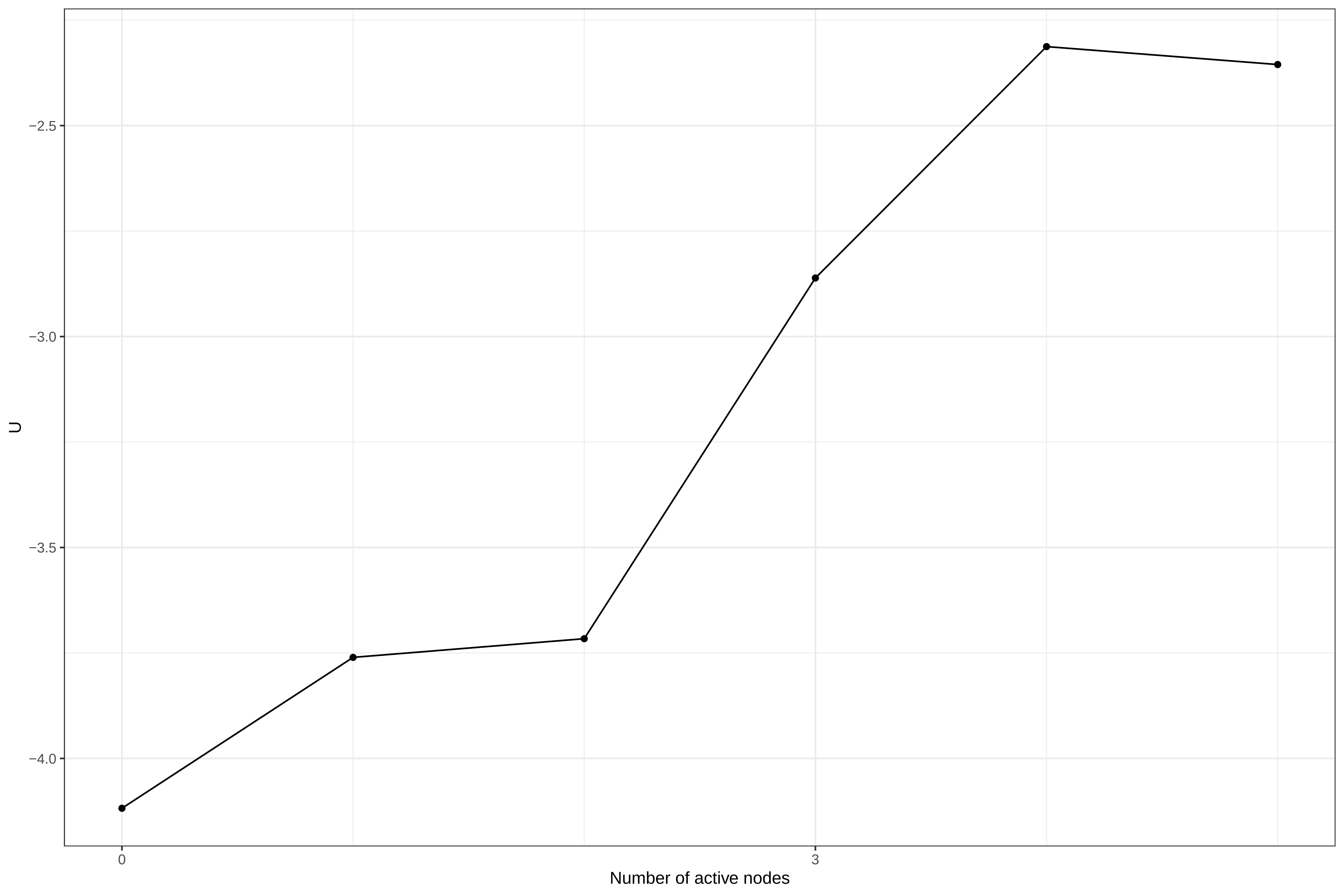

−2.5
−3.0
−3.5
−4.0
U
0
3
Number of active nodes

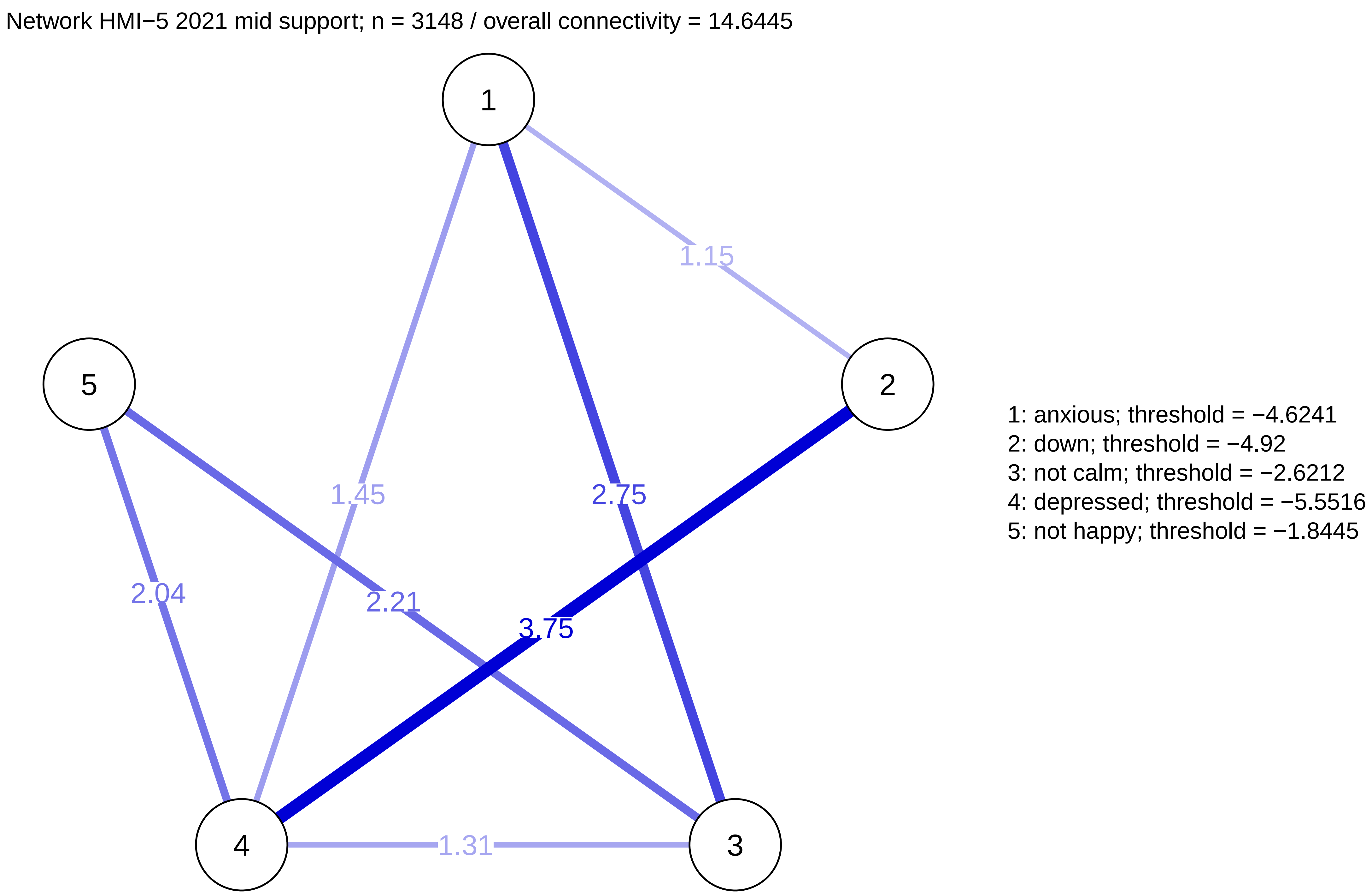
Network HMI−5 2021 mid support; n = 3148 / overall connectivity = 14.6445
1
2
3
4
5
1.15
1.45
2.75
2.04
2.21
3.75
1.31
1: anxious; threshold = −4.6241
2: down; threshold = −4.92
3: not calm; threshold = −2.6212
4: depressed; threshold = −5.5516
5: not happy; threshold = −1.8445

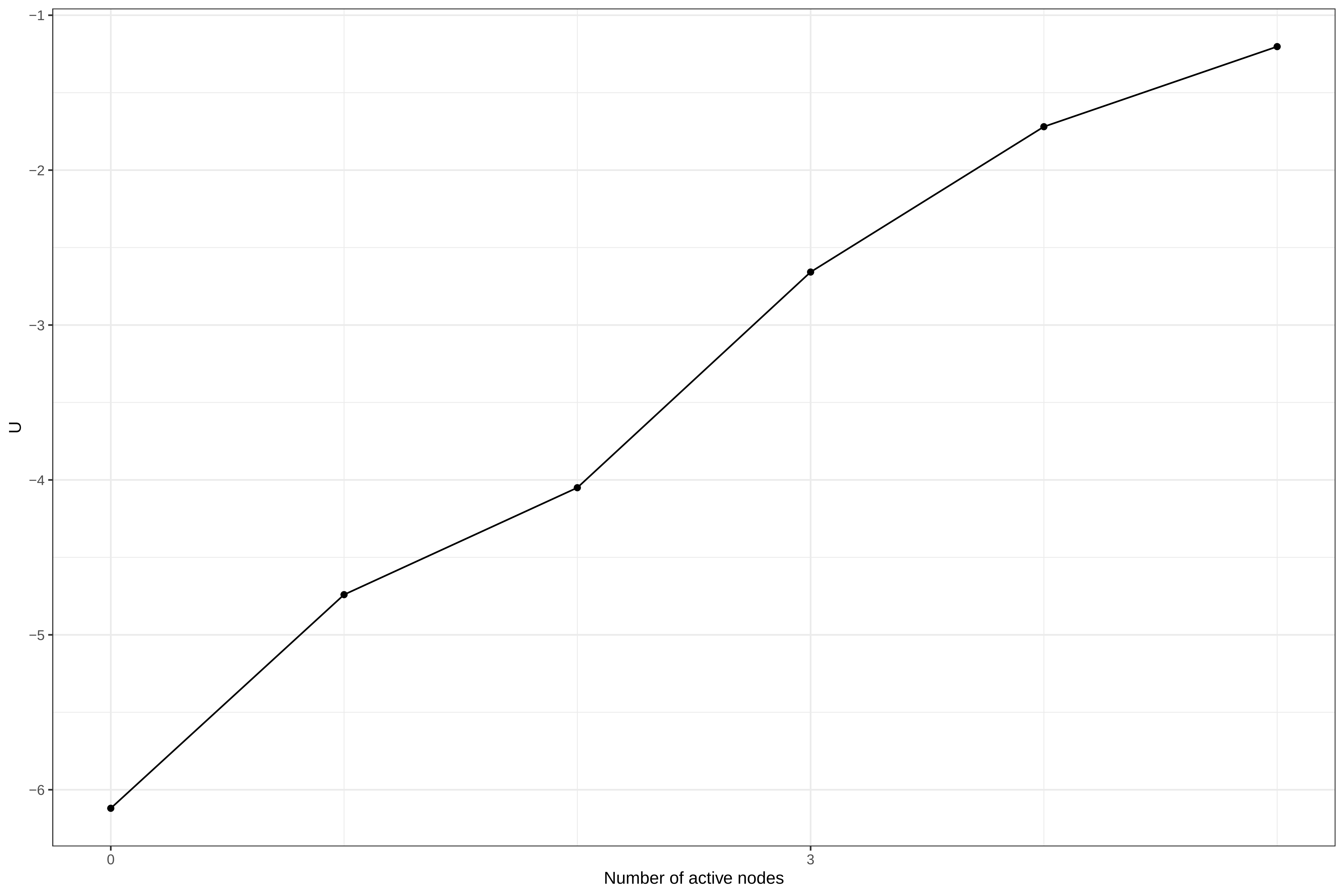

−1
−2
−3
−4
−5
−6
U
0
3
Number of active nodes

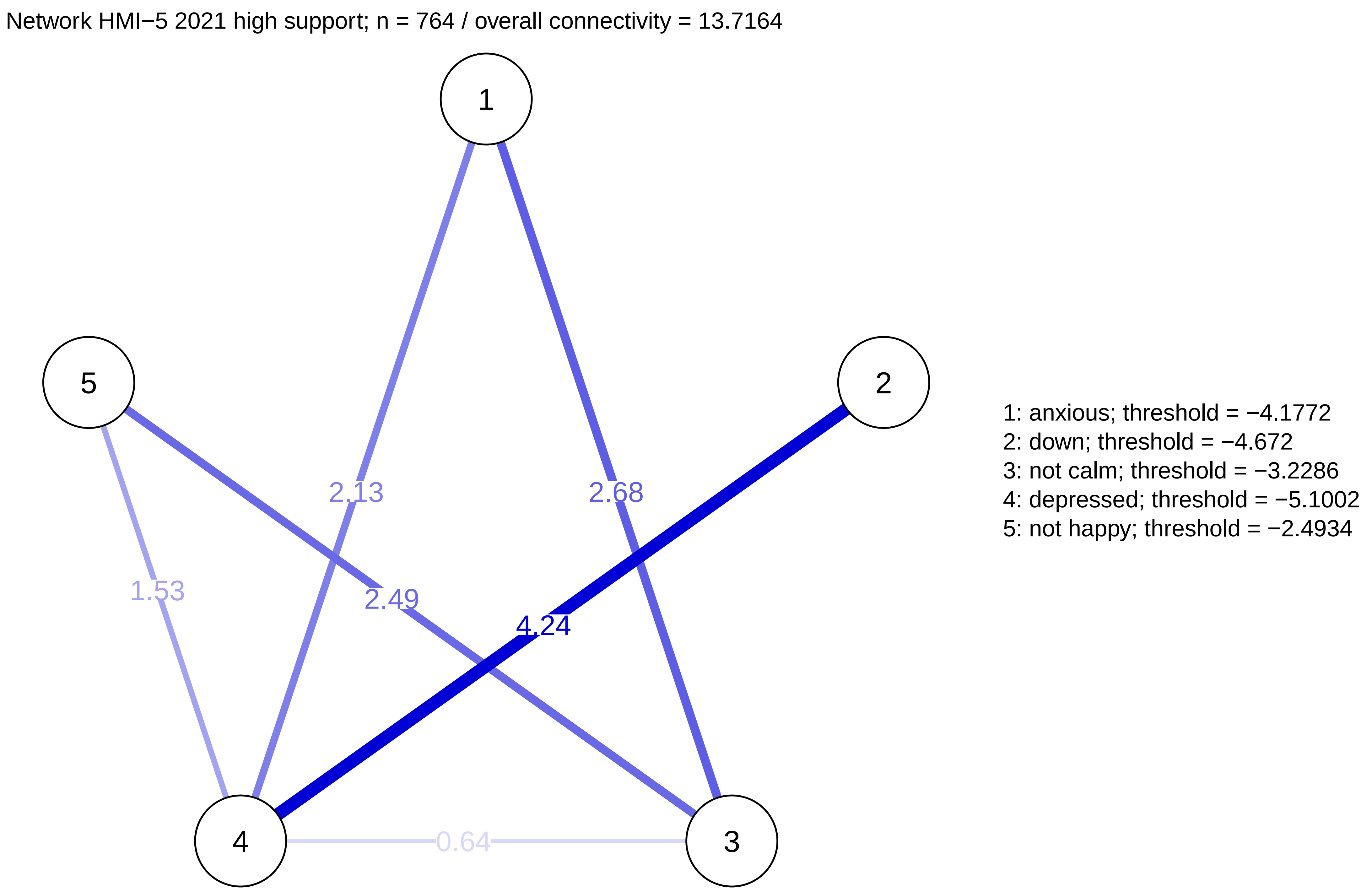

Network HMI−5 2021 high support; n = 764 / overall connectivity = 13.7164
1
2
3
4
5
2.13
2.68
1.53
2.49
4.24
0.64
1: anxious; threshold = −4.1772
2: down; threshold = −4.672
3: not calm; threshold = −3.2286
4: depressed; threshold = −5.1002
5: not happy; threshold = −2.4934

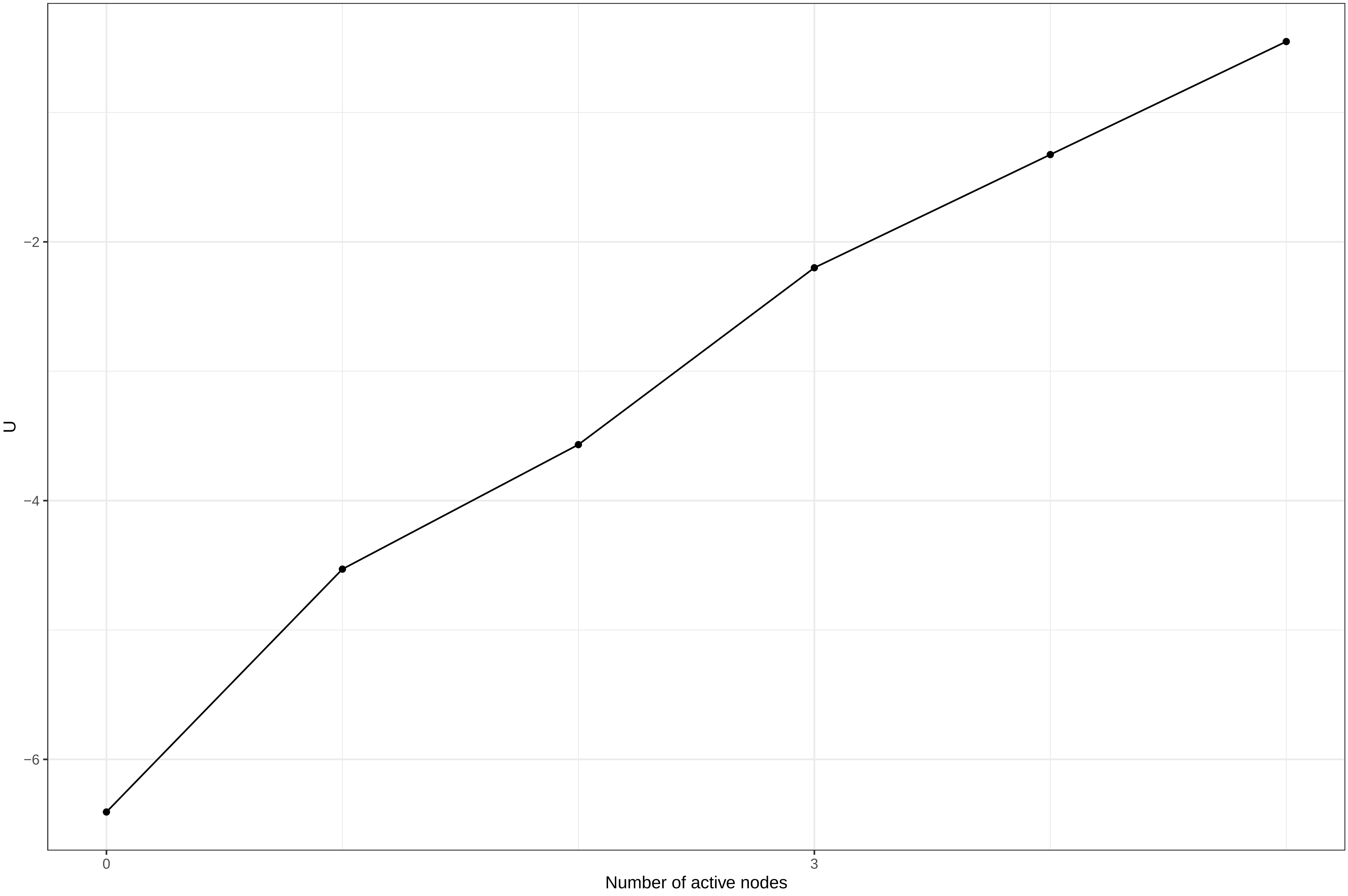
U
−2
−4
−6
0
3
Number of active nodes

Network HMI−5 2022 low support; n = 1251 / overall connectivity = 14.0826

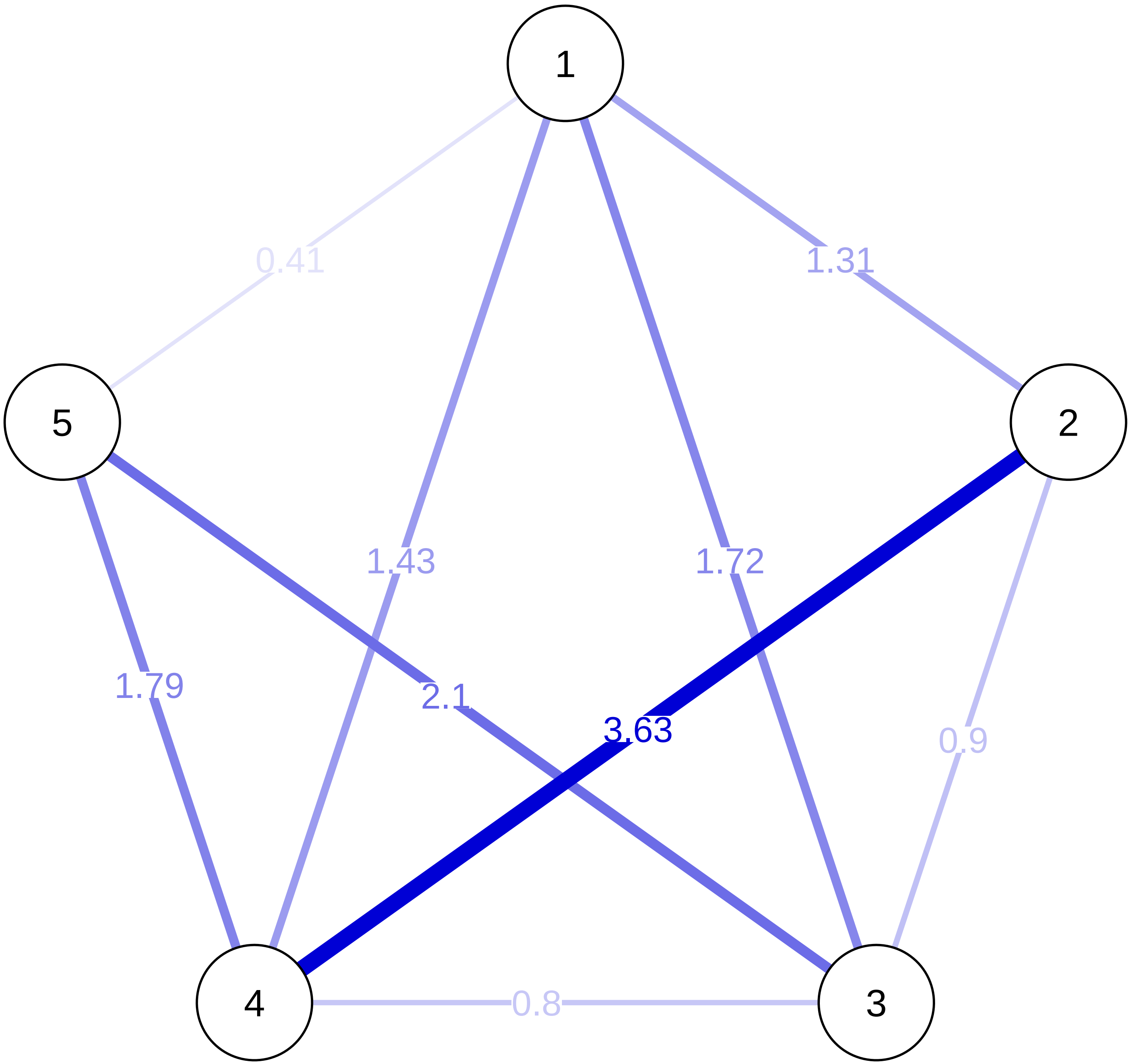

1: anxious; threshold = −3.5867
2: down; threshold = −4.8153
3: not calm; threshold = −2.0386
4: depressed; threshold = −4.7896
5: not happy; threshold = −0.9158

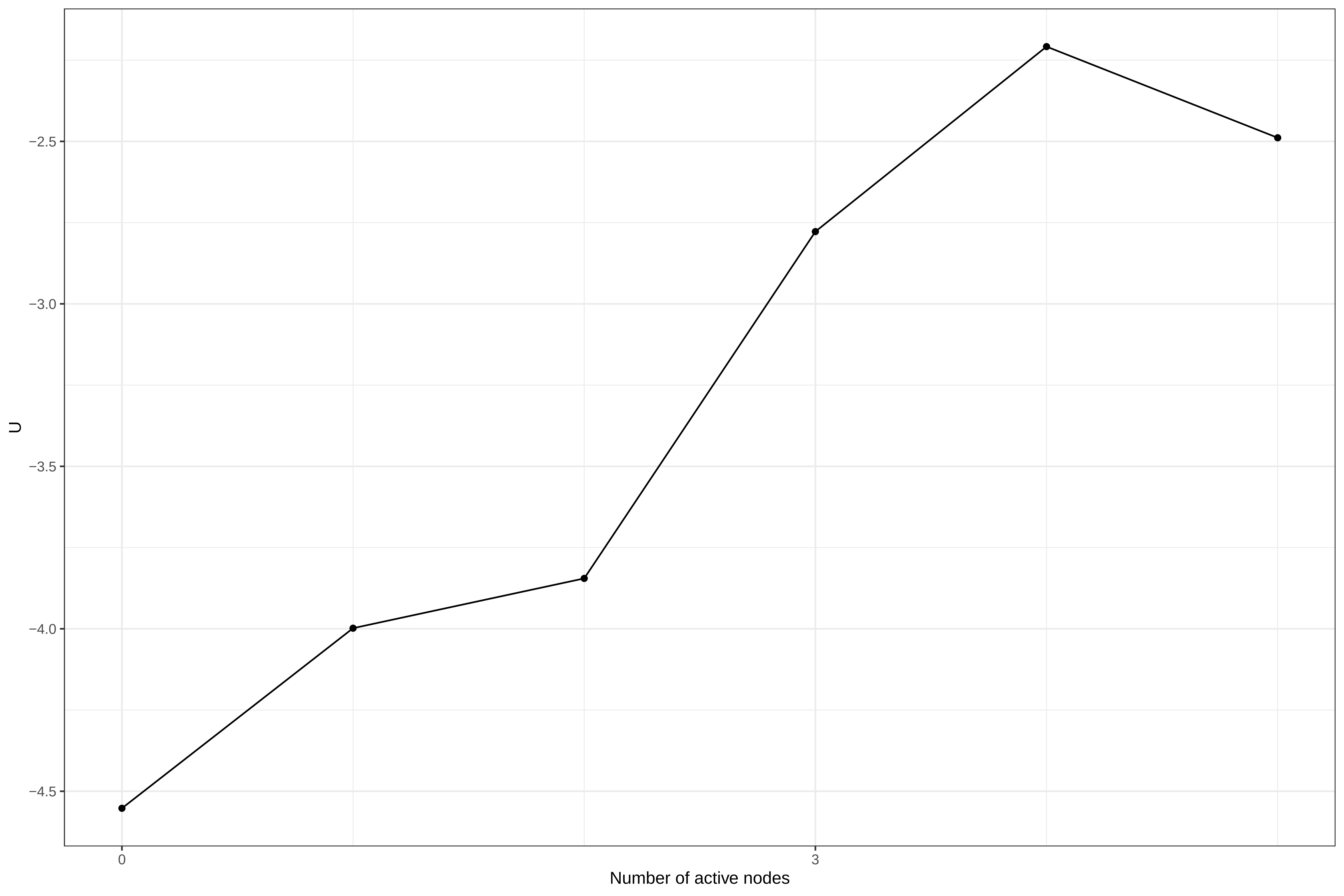

−2.5
−3.0
−3.5
−4.0
−4.5
U
0
3
Number of active nodes

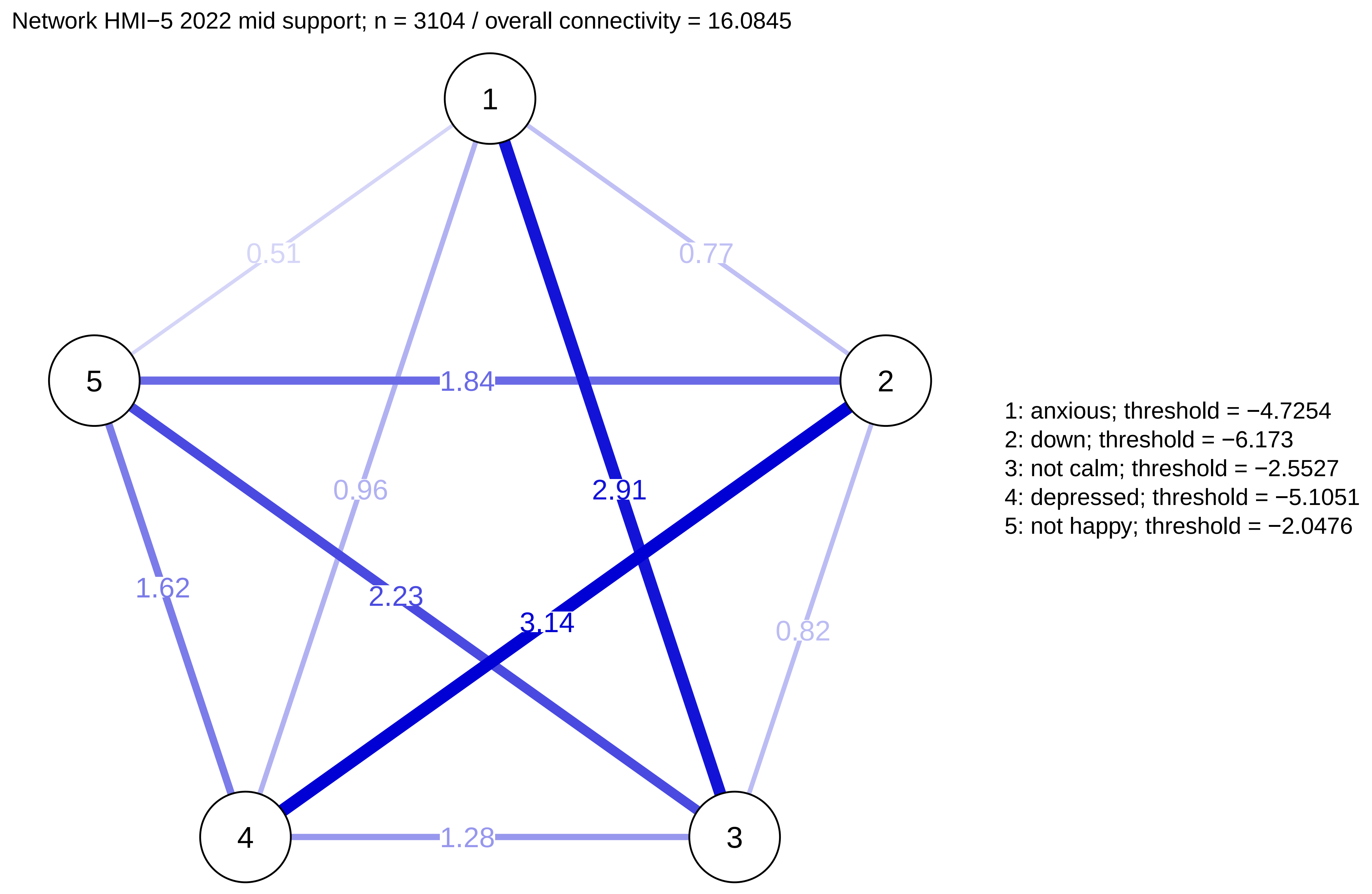
Network HMI−5 2022 mid support; n = 3104 / overall connectivity = 16.0845
1
2
3
4
5
0.51
0.77
1.84
0.96
2.91
1.62
2.23
3.14
0.82
1.28
1: anxious; threshold = −4.7254
2: down; threshold = −6.173
3: not calm; threshold = −2.5527
4: depressed; threshold = −5.1051
5: not happy; threshold = −2.0476

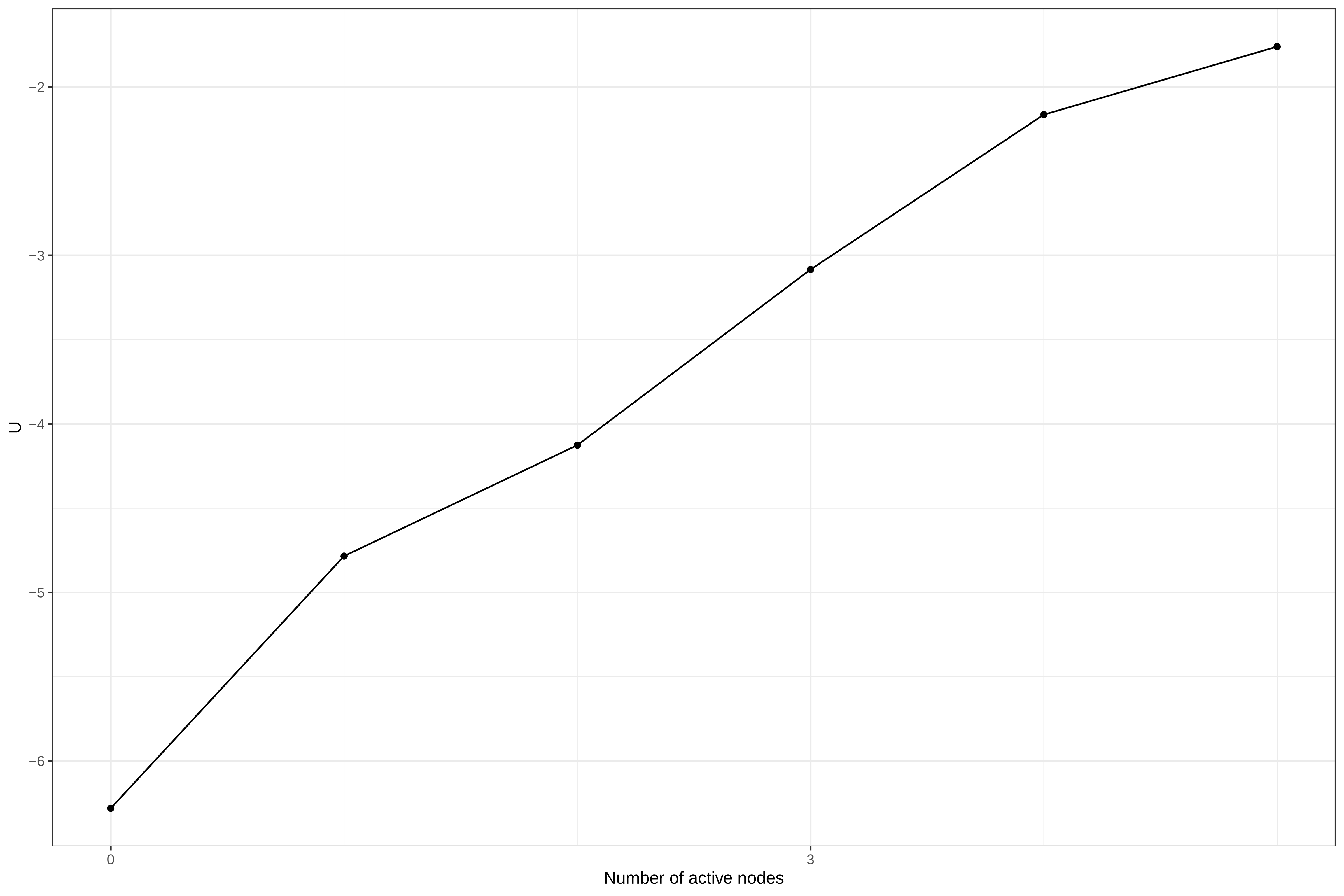

−2
−3
−4
−5
−6
U
0
3
Number of active nodes

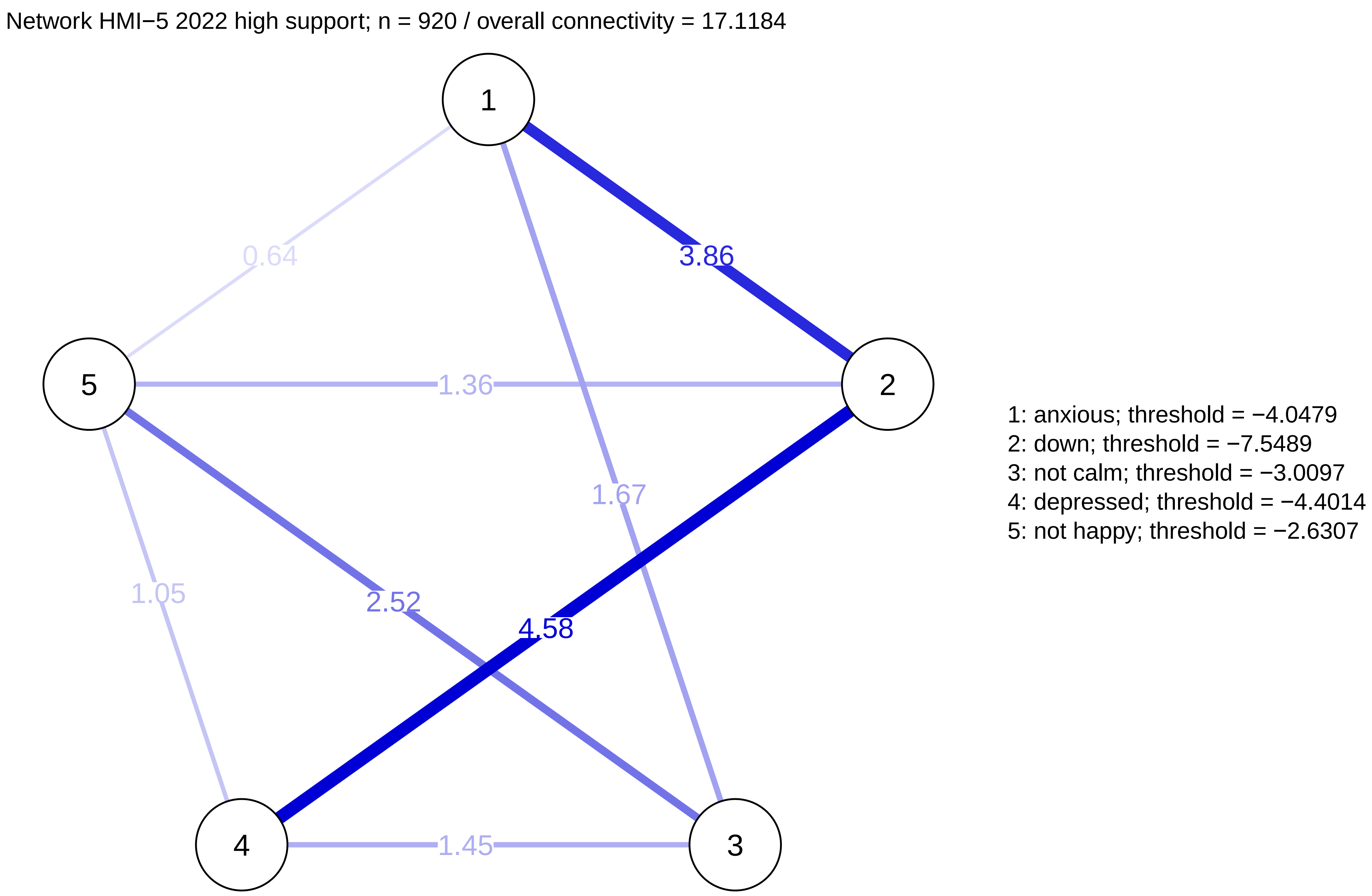

Network HMI−5 2022 high support; n = 920 / overall connectivity = 17.1184
1
2
3
4
5
3.86
0.64
1.36
1.67
1.05
2.52
4.58
1.45
1: anxious; threshold = −4.0479
2: down; threshold = −7.5489
3: not calm; threshold = −3.0097
4: depressed; threshold = −4.4014
5: not happy; threshold = −2.6307

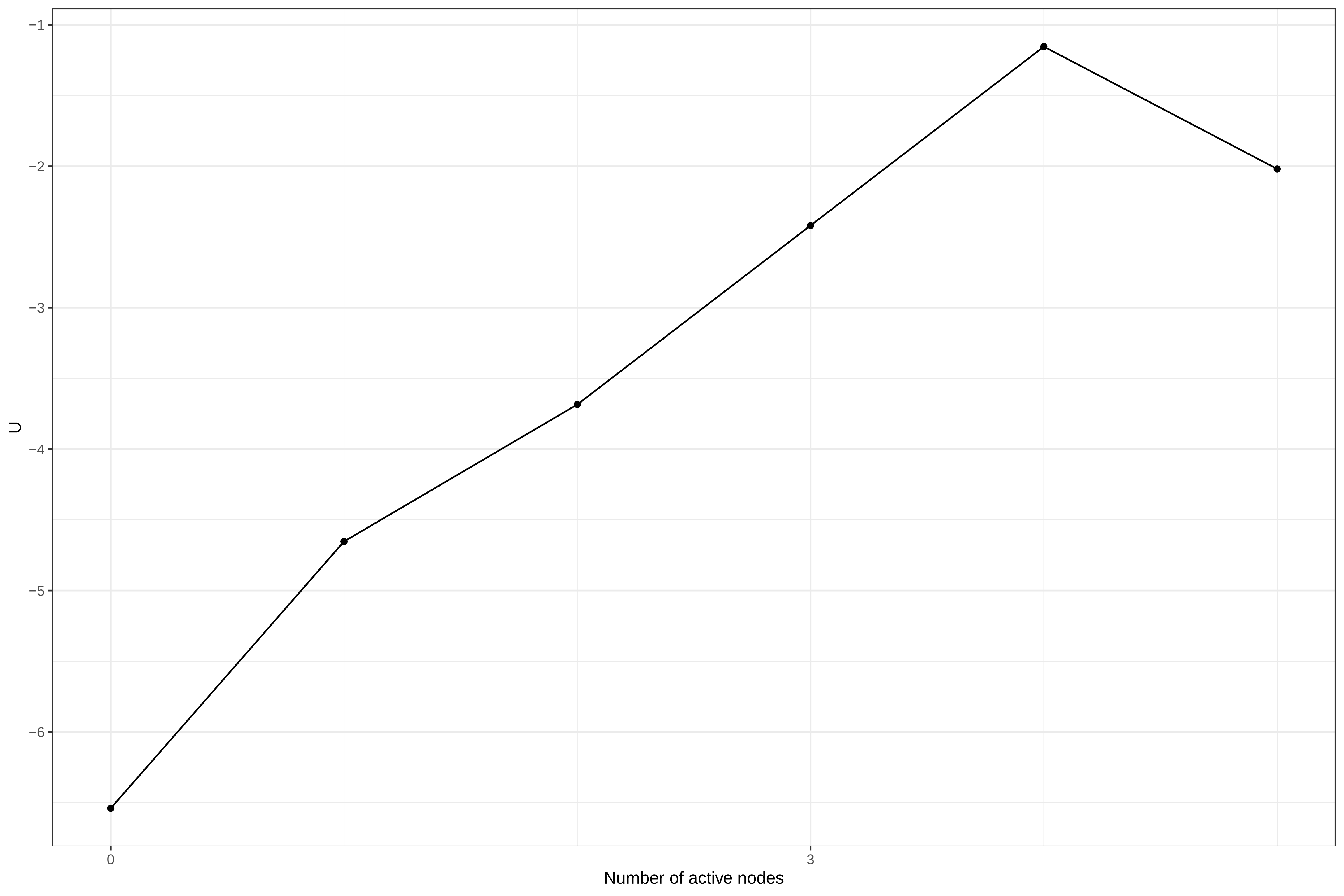

−1
−2
−3
−4
−5
−6
U
0
3
Number of active nodes

Network HMI−5 2012 low urban; n = 2076 / overall connectivity = 12.6054

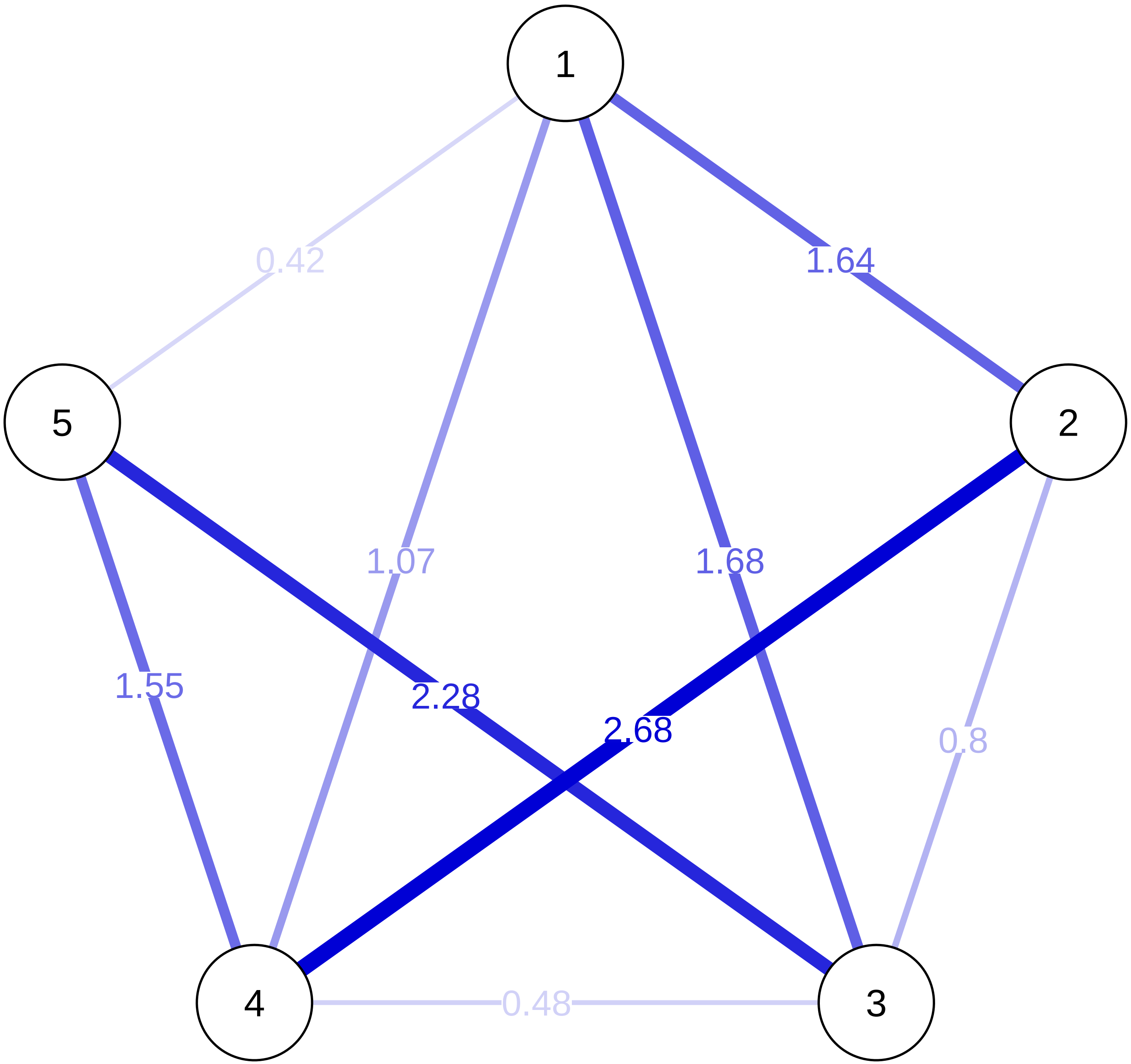

1: anxious; threshold = −3.443
2: down; threshold = −4.1881
3: not calm; threshold = −2.427
4: depressed; threshold = −3.6896
5: not happy; threshold = −2.342

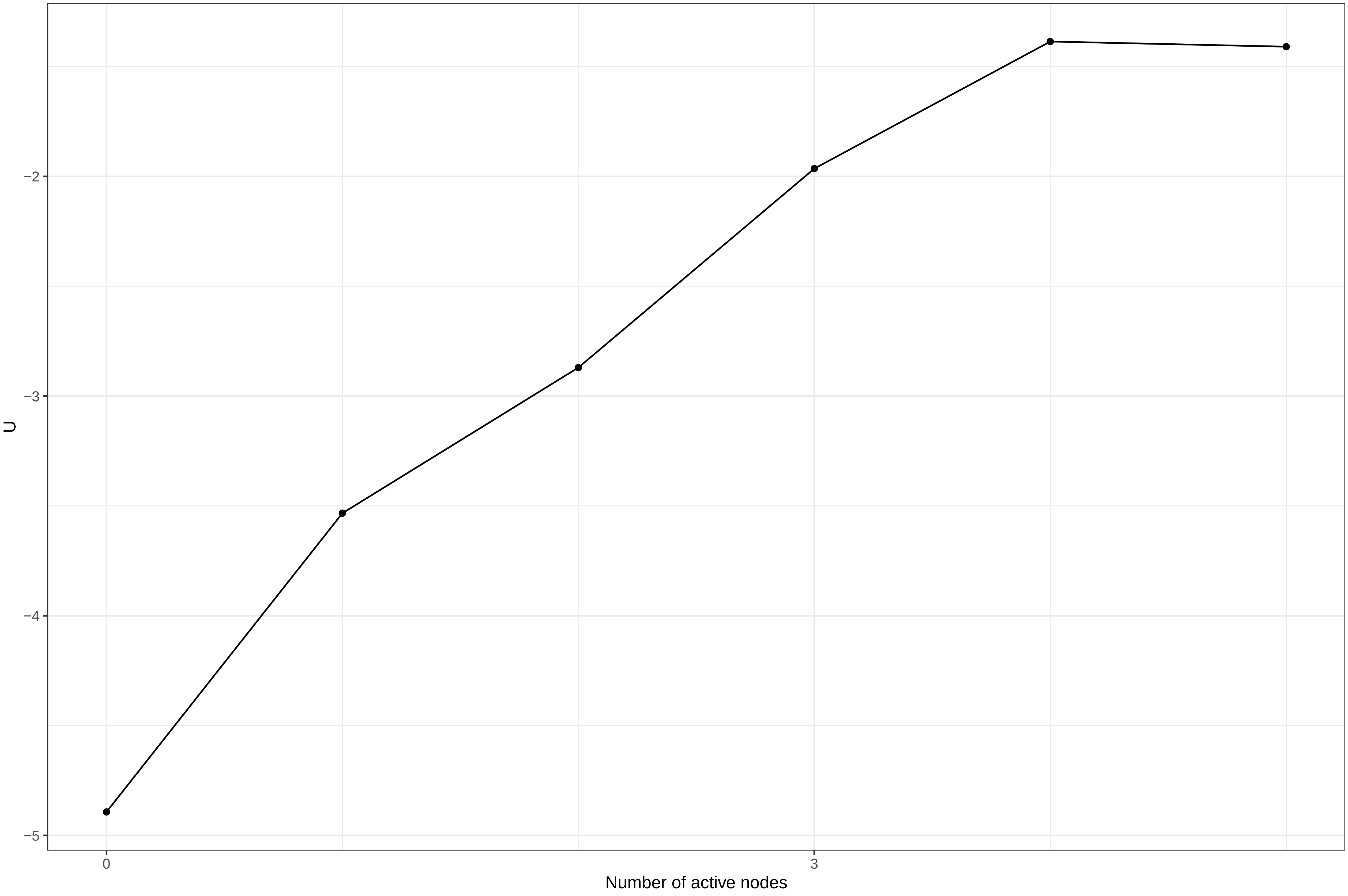

−2
−3
−4
−5
U
0
3
Number of active nodes

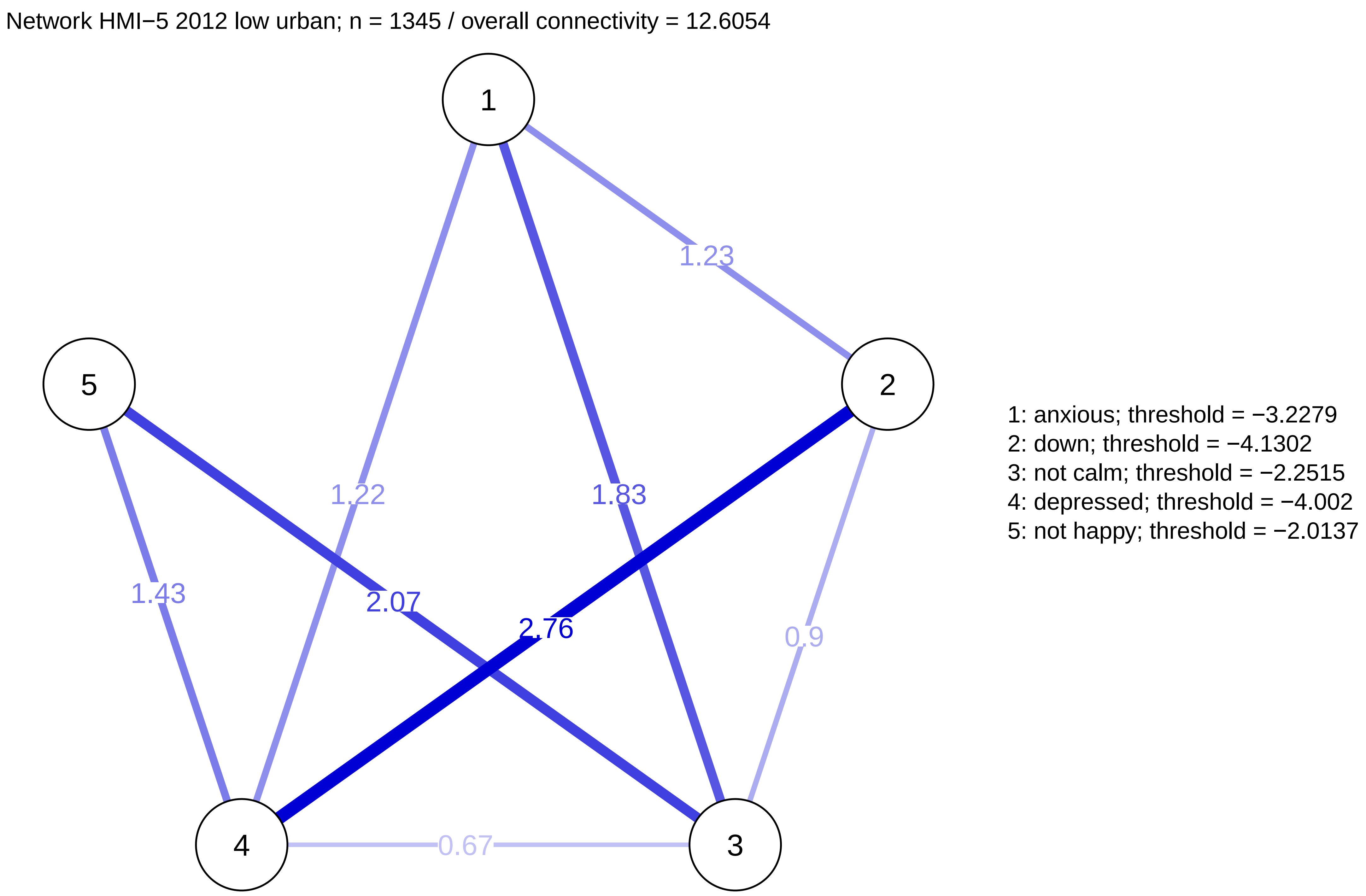
Network HMI−5 2012 low urban; n = 1345 / overall connectivity = 12.6054
1
2
3
4
5
1.23
1.22
1.83
1.43
2.07
2.76
0.9
0.67
1: anxious; threshold = −3.2279
2: down; threshold = −4.1302
3: not calm; threshold = −2.2515
4: depressed; threshold = −4.002
5: not happy; threshold = −2.0137

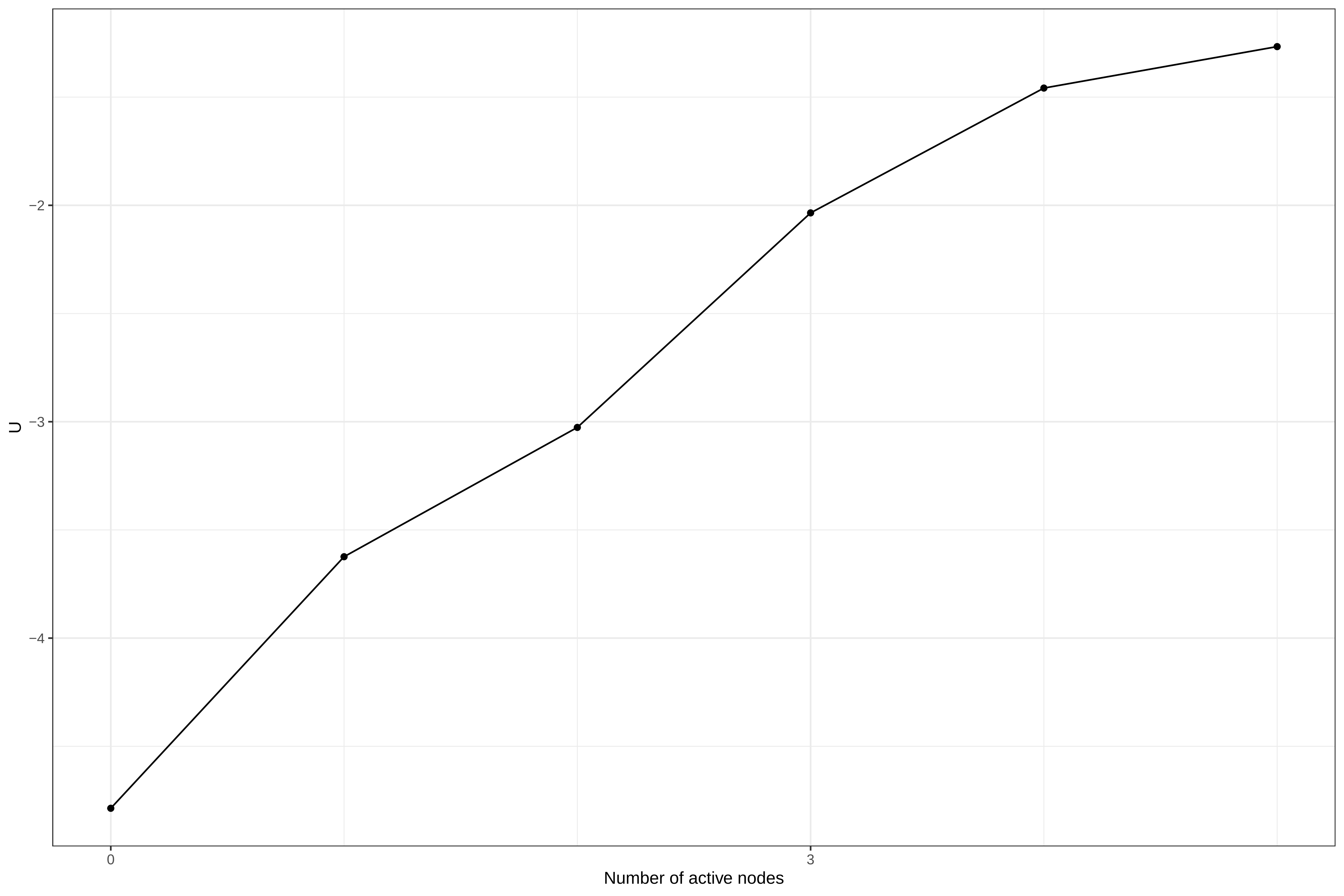

−2
−3
−4
U
0
3
Number of active nodes

Network HMI−5 2012 low urban; n = 2206 / overall connectivity = 12.6054

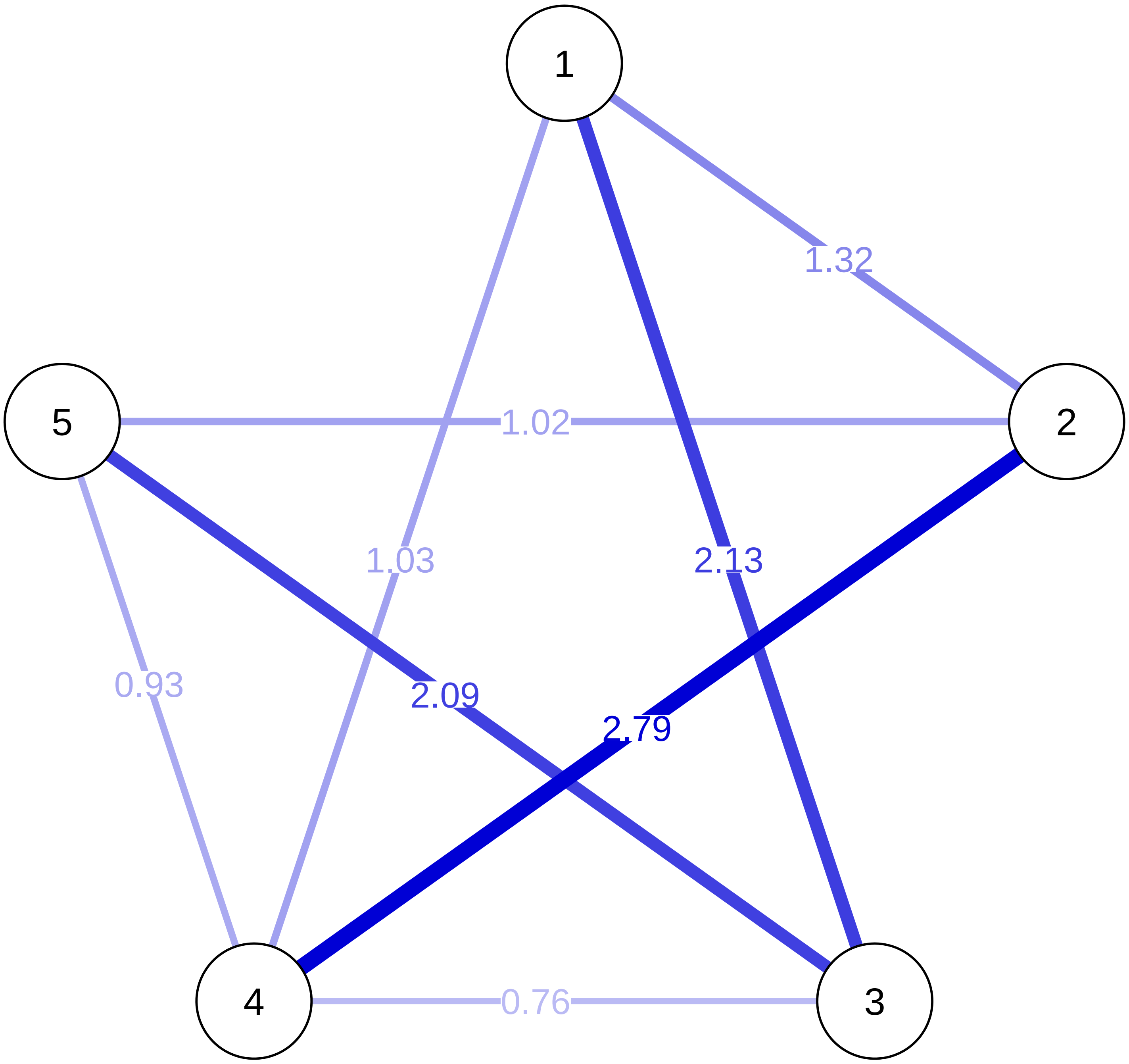

1: anxious; threshold = −3.4814
2: down; threshold = −4.546
3: not calm; threshold = −2.0746
4: depressed; threshold = −3.551
5: not happy; threshold = −2.1913

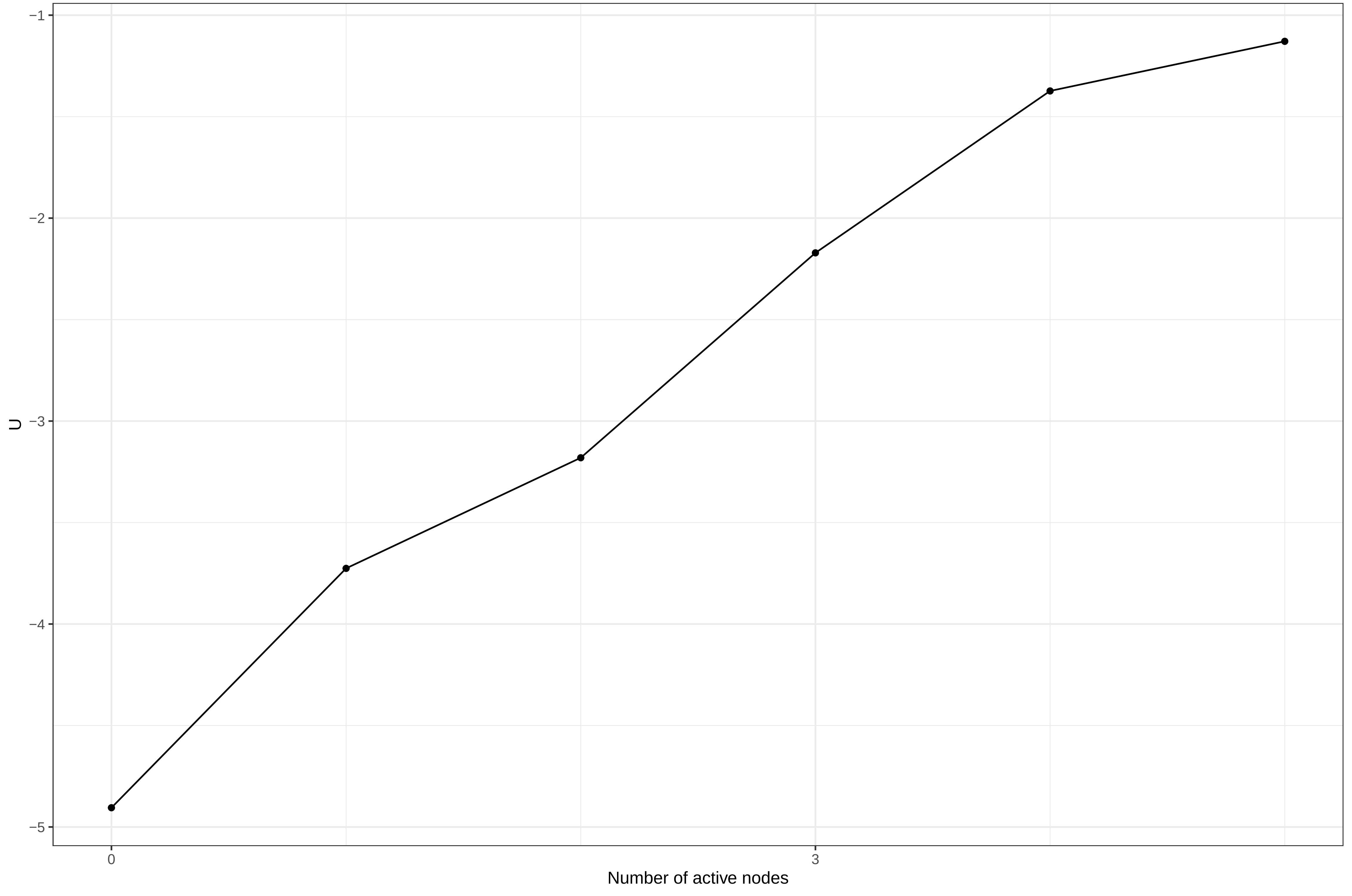
−1
−2
−3
−4
−5
U
0
3
Number of active nodes

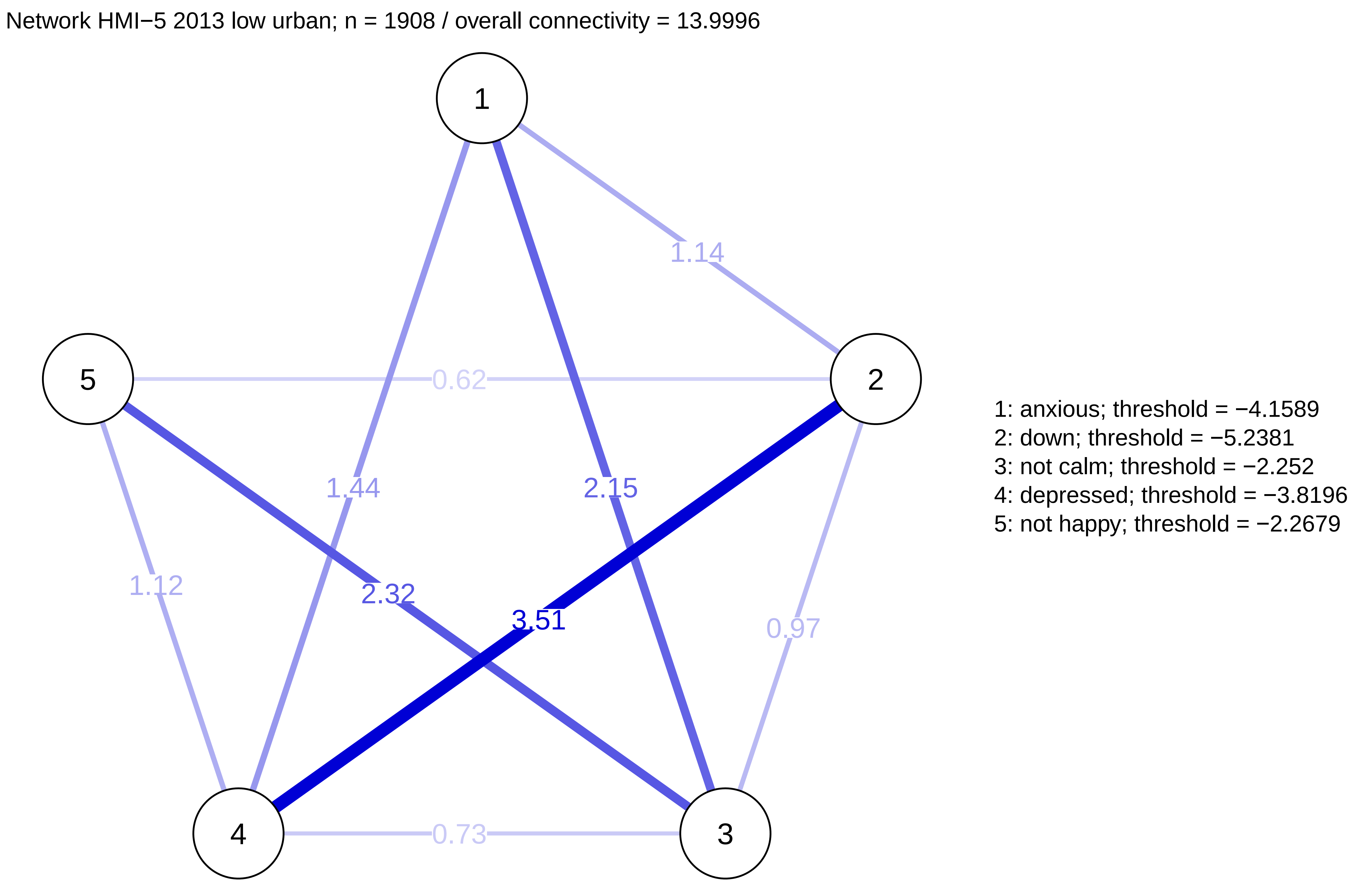
Network HMI−5 2013 low urban; n = 1908 / overall connectivity = 13.9996
1
2
3
4
5
1.14
0.62
1.44
2.15
1.12
2.32
3.51
0.97
0.73
1: anxious; threshold = −4.1589
2: down; threshold = −5.2381
3: not calm; threshold = −2.252
4: depressed; threshold = −3.8196
5: not happy; threshold = −2.2679

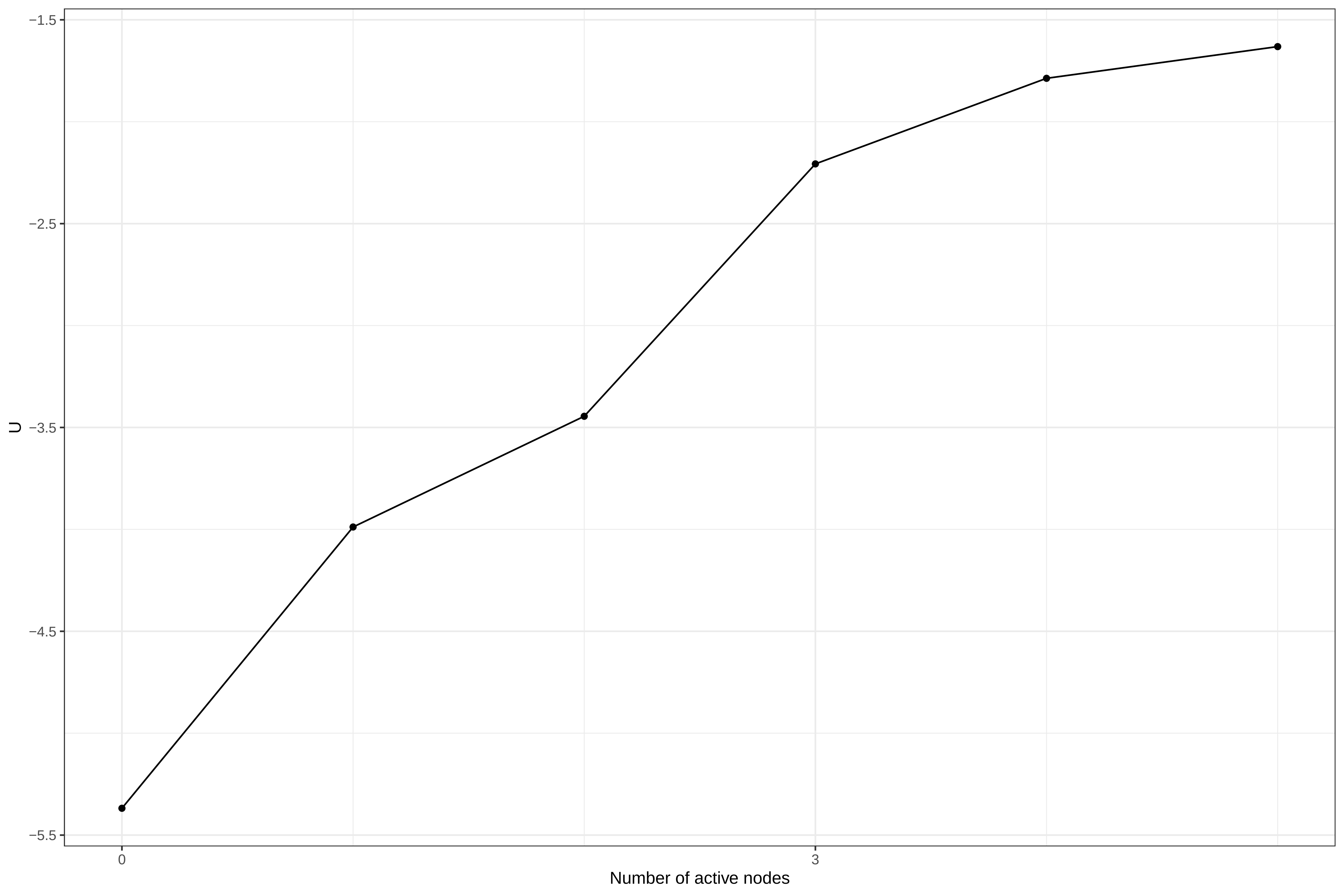
−1.5
−2.5
−3.5
−4.5
−5.5
U
0
3
Number of active nodes

Network HMI−5 2013 mid urban; n = 1256 / overall connectivity = 12.8237

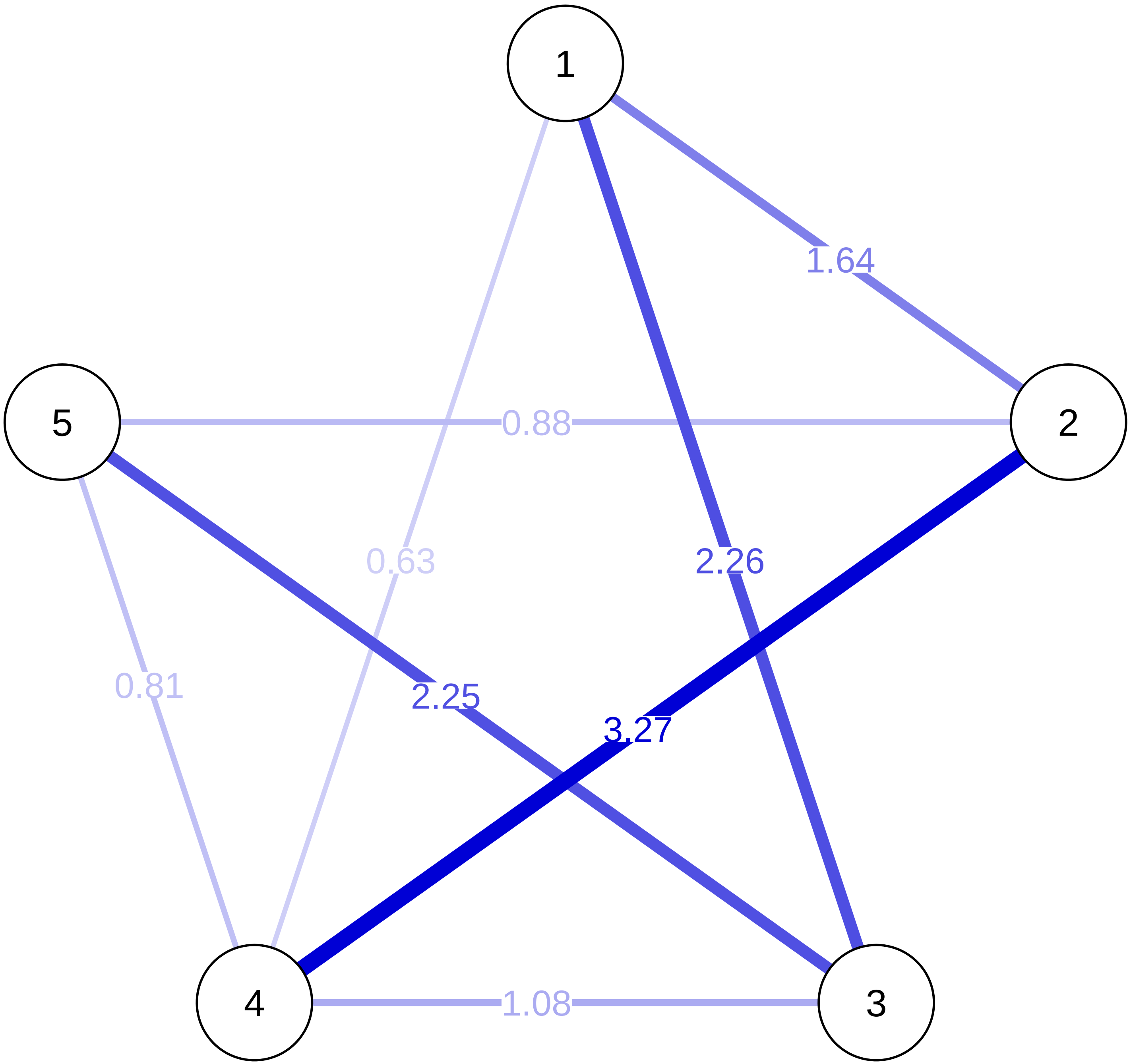

1: anxious; threshold = −3.8802
2: down; threshold = −4.5078
3: not calm; threshold = −2.1952
4: depressed; threshold = −3.8619
5: not happy; threshold = −1.9845

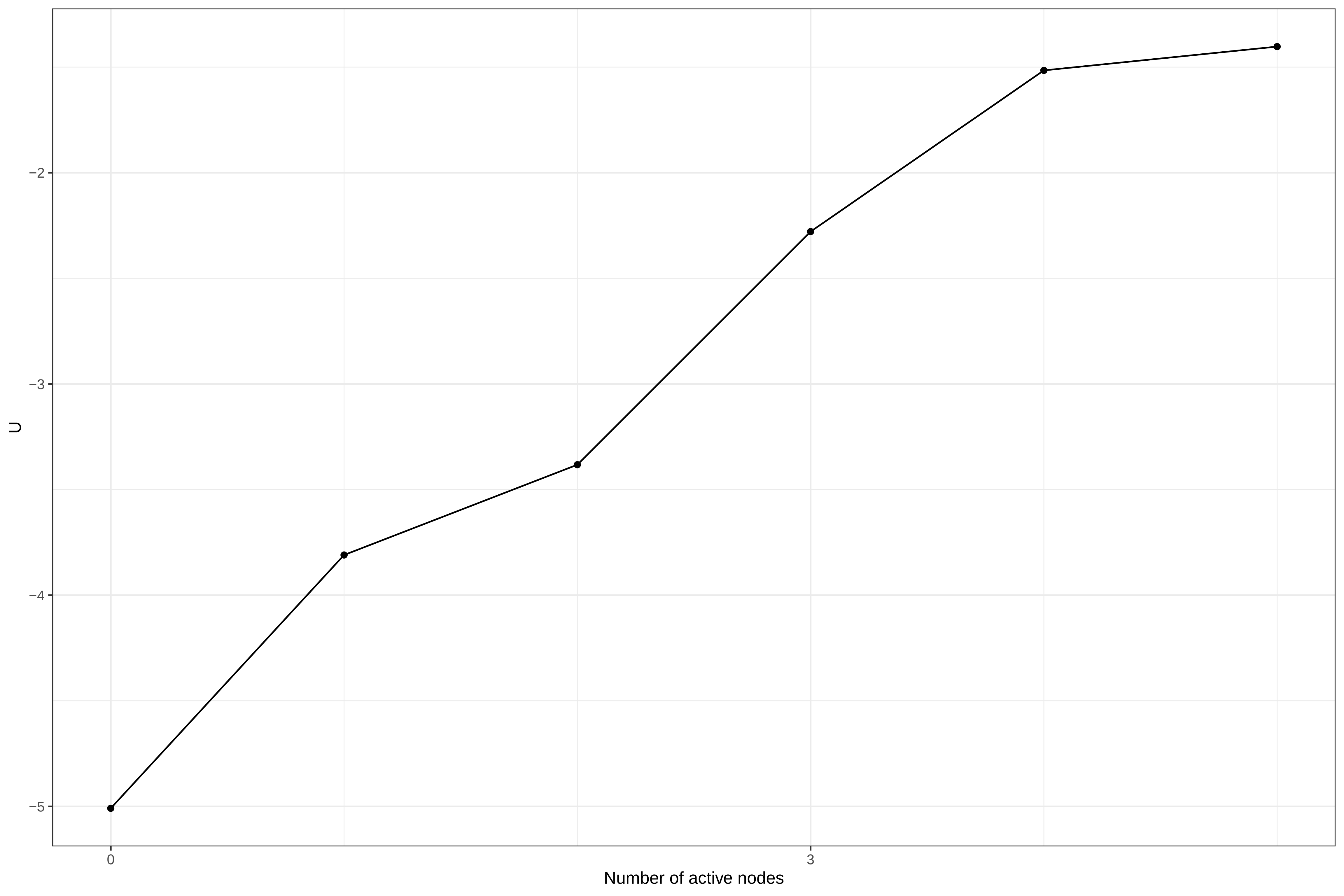

U
−2
−3
−4
−5
0
3
Number of active nodes

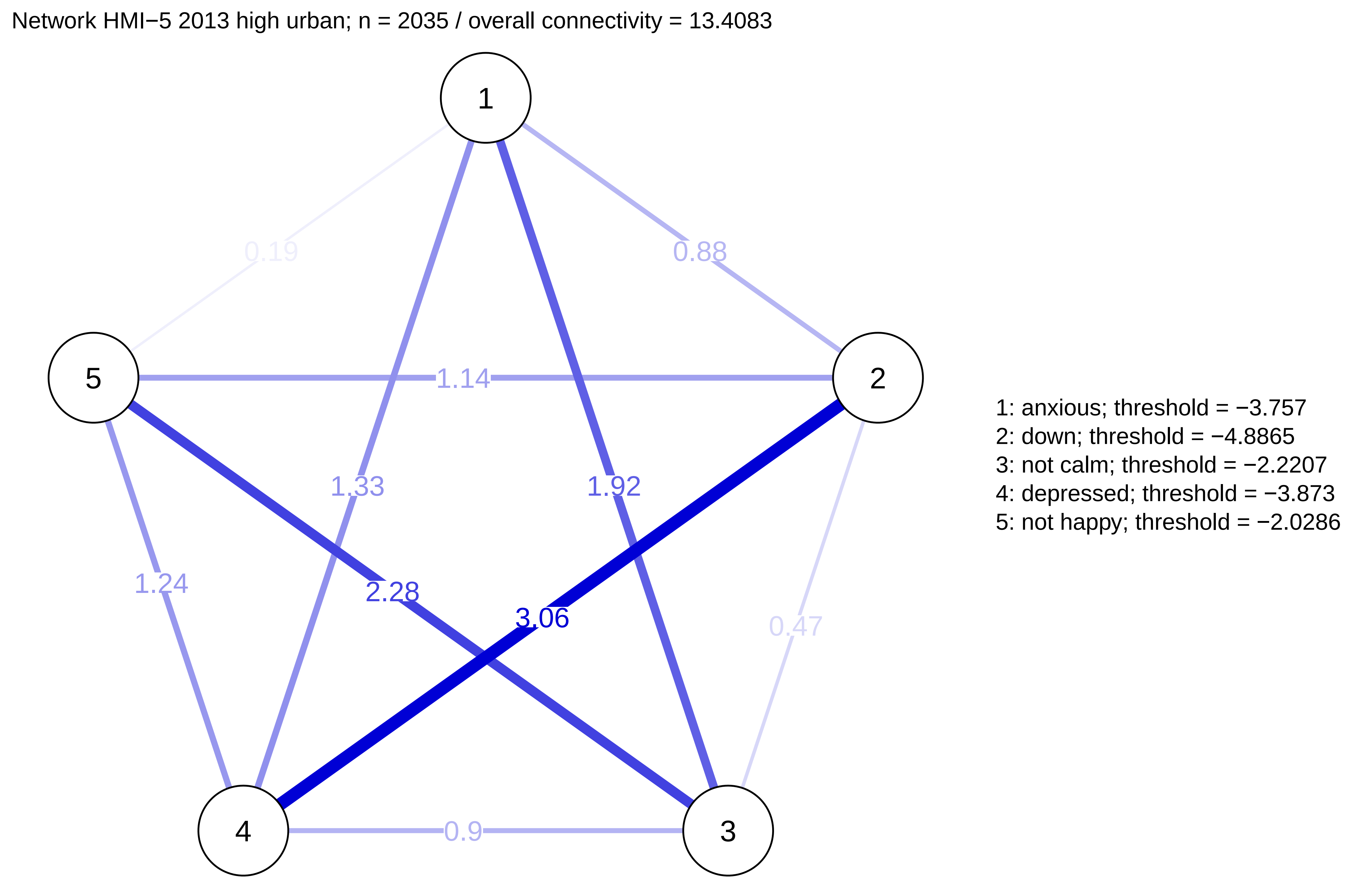
Network HMI−5 2013 high urban; n = 2035 / overall connectivity = 13.4083
1
2
3
4
5
0.19
0.88
1.14
1.33
1.92
1.24
2.28
3.06
0.47
0.9
1: anxious; threshold = −3.757
2: down; threshold = −4.8865
3: not calm; threshold = −2.2207
4: depressed; threshold = −3.873
5: not happy; threshold = −2.0286

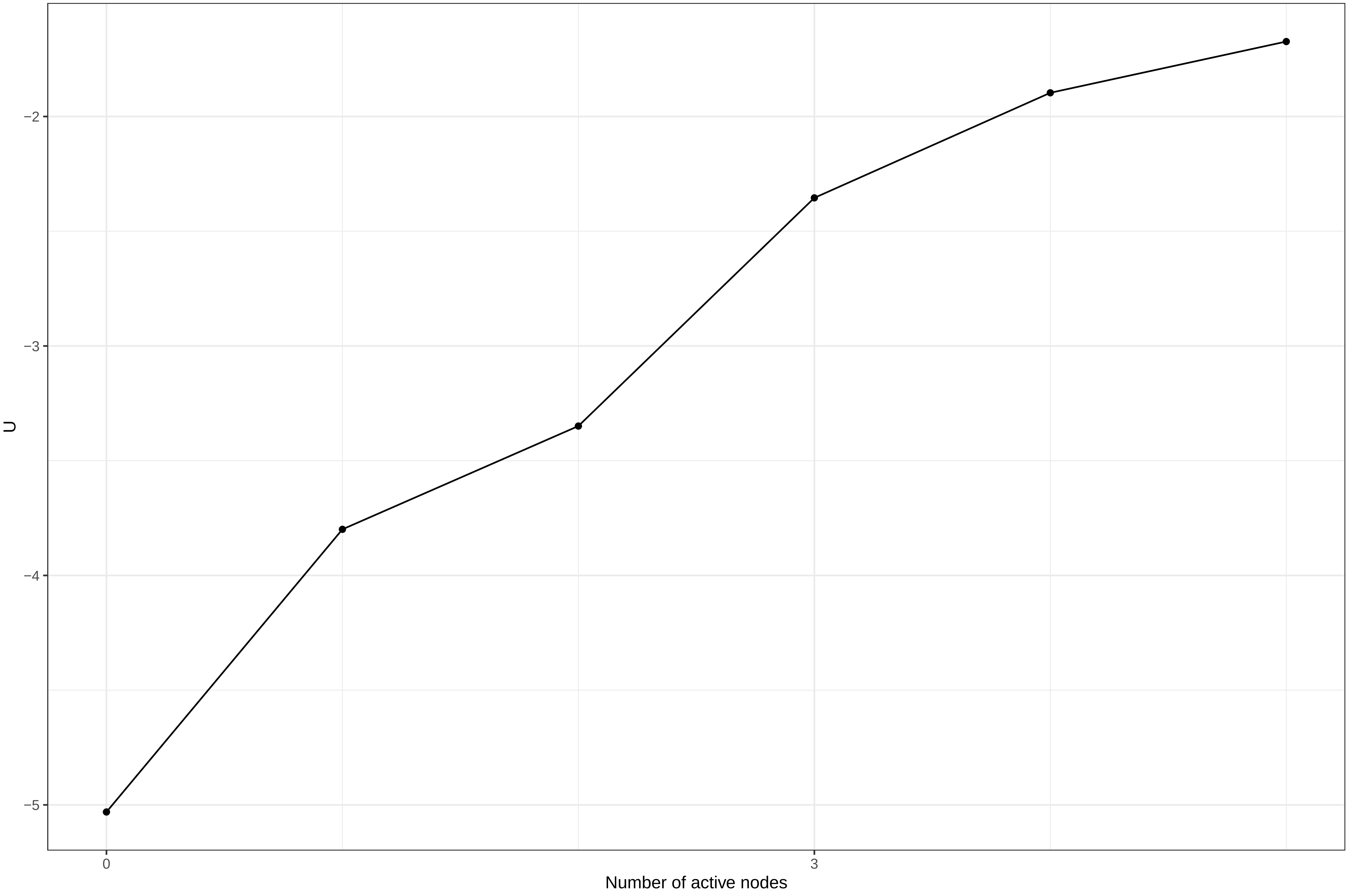

−2
−3
−4
−5
U
0
3
Number of active nodes

Network HMI−5 2015 low urban; n = 1639 / overall connectivity = 17.7104

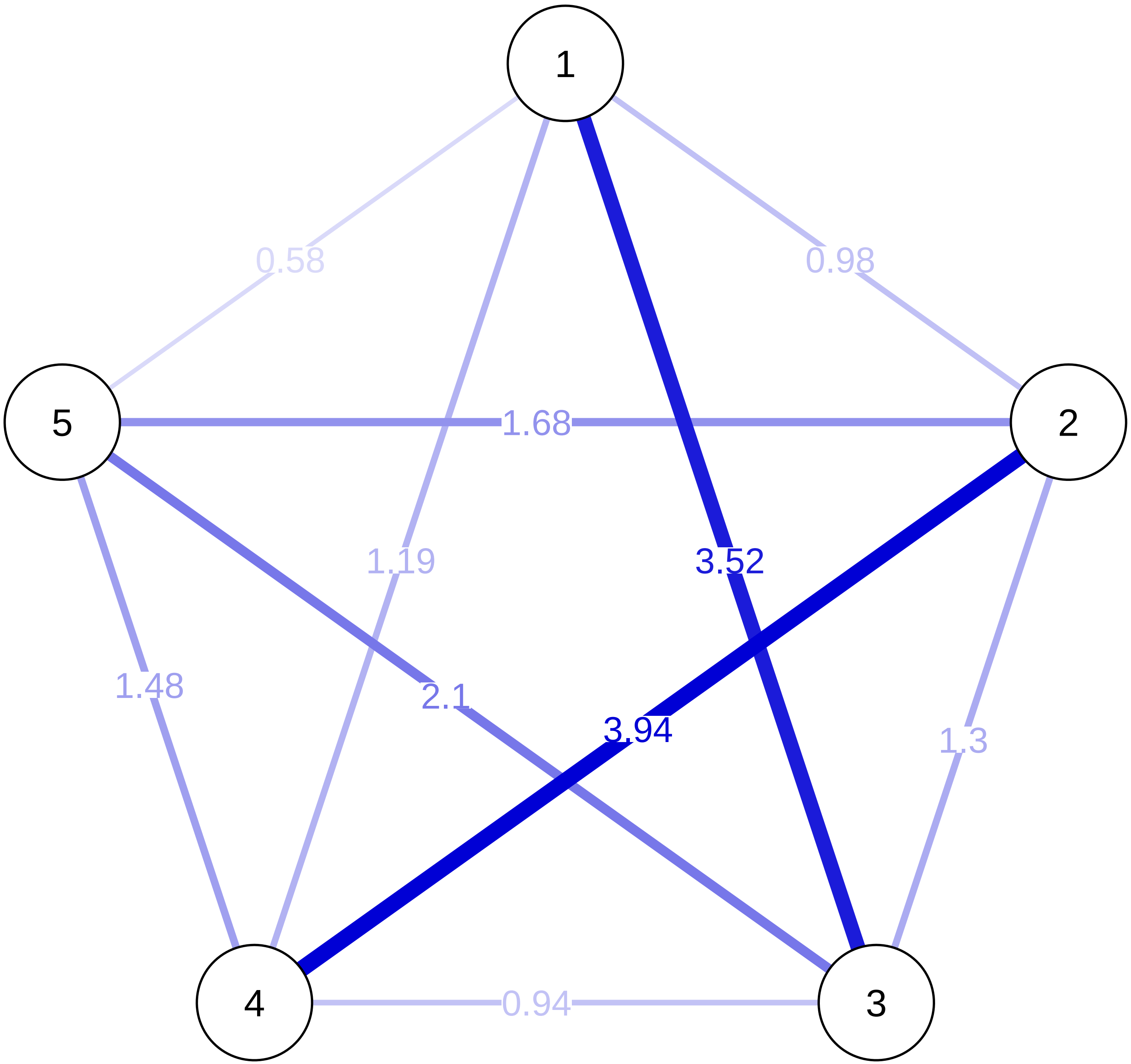

1: anxious; threshold = −5.6626
2: down; threshold = −7.0597
3: not calm; threshold = −2.3916
4: depressed; threshold = −4.7072
5: not happy; threshold = −2.0433

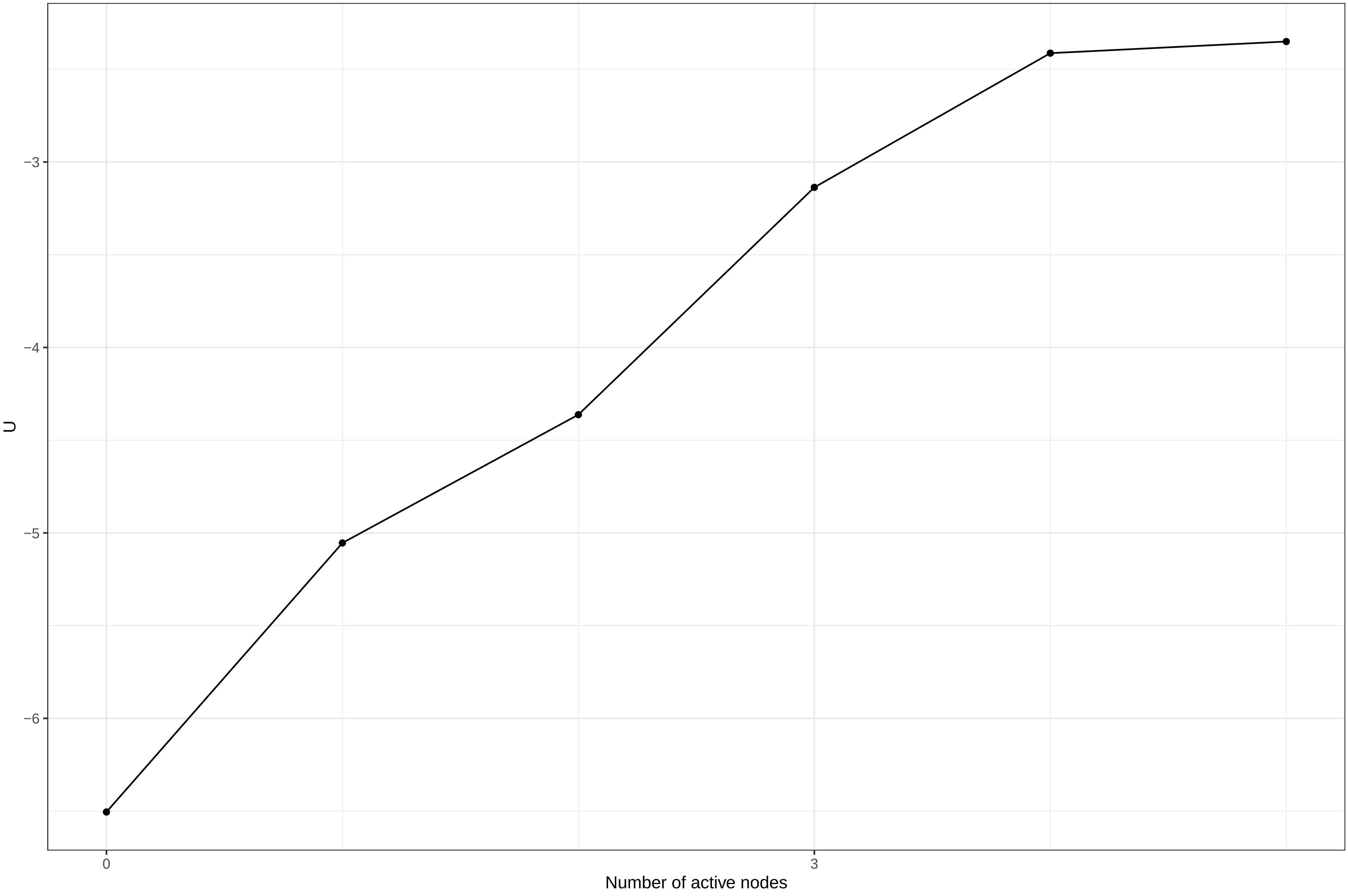
−3
−4
−5
−6
U
0
3
Number of active nodes

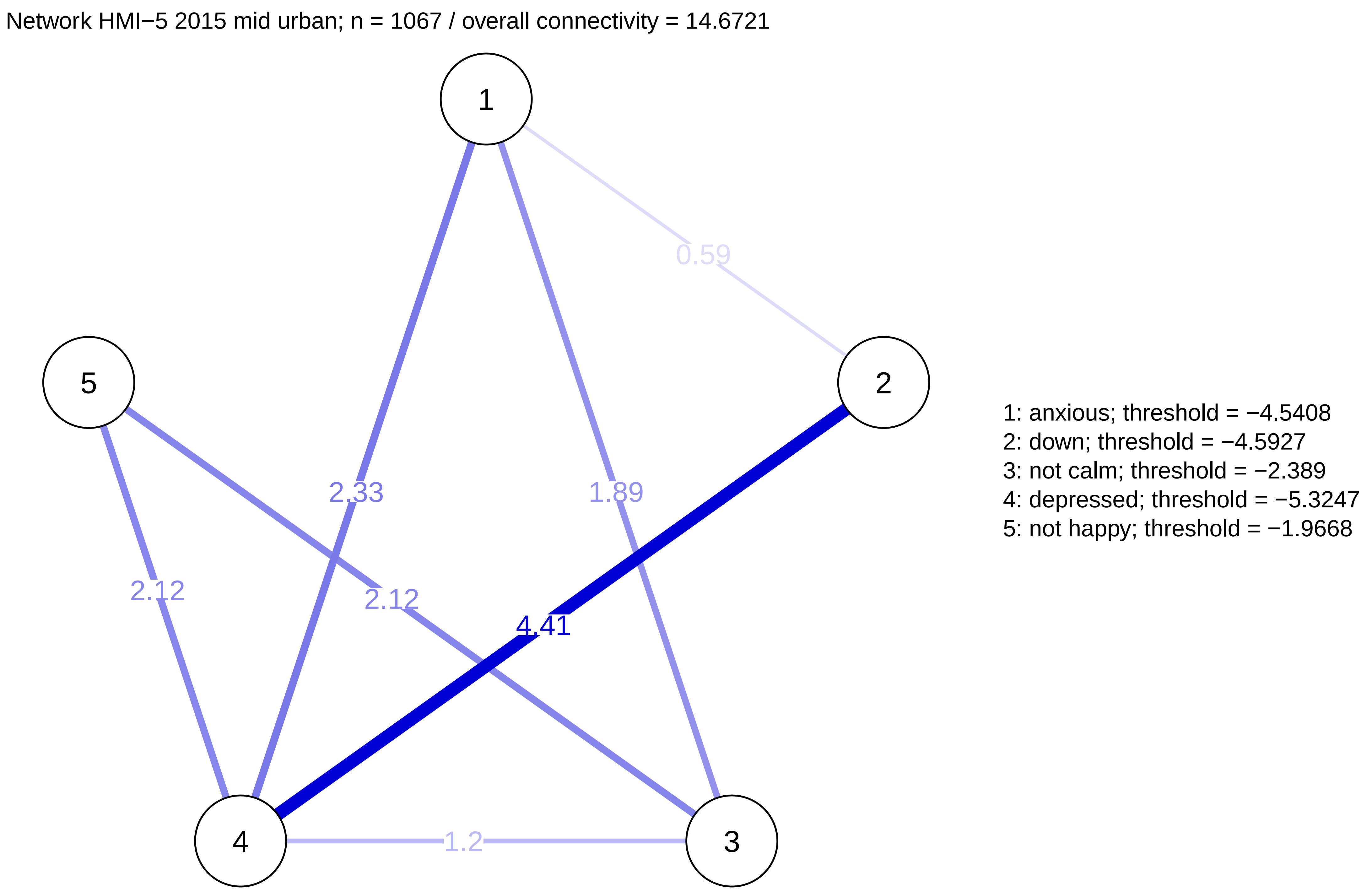
Network HMI−5 2015 mid urban; n = 1067 / overall connectivity = 14.6721
1
2
3
4
5
0.59
2.33
1.89
2.12
2.12
4.41
1.2
1: anxious; threshold = −4.5408
2: down; threshold = −4.5927
3: not calm; threshold = −2.389
4: depressed; threshold = −5.3247
5: not happy; threshold = −1.9668

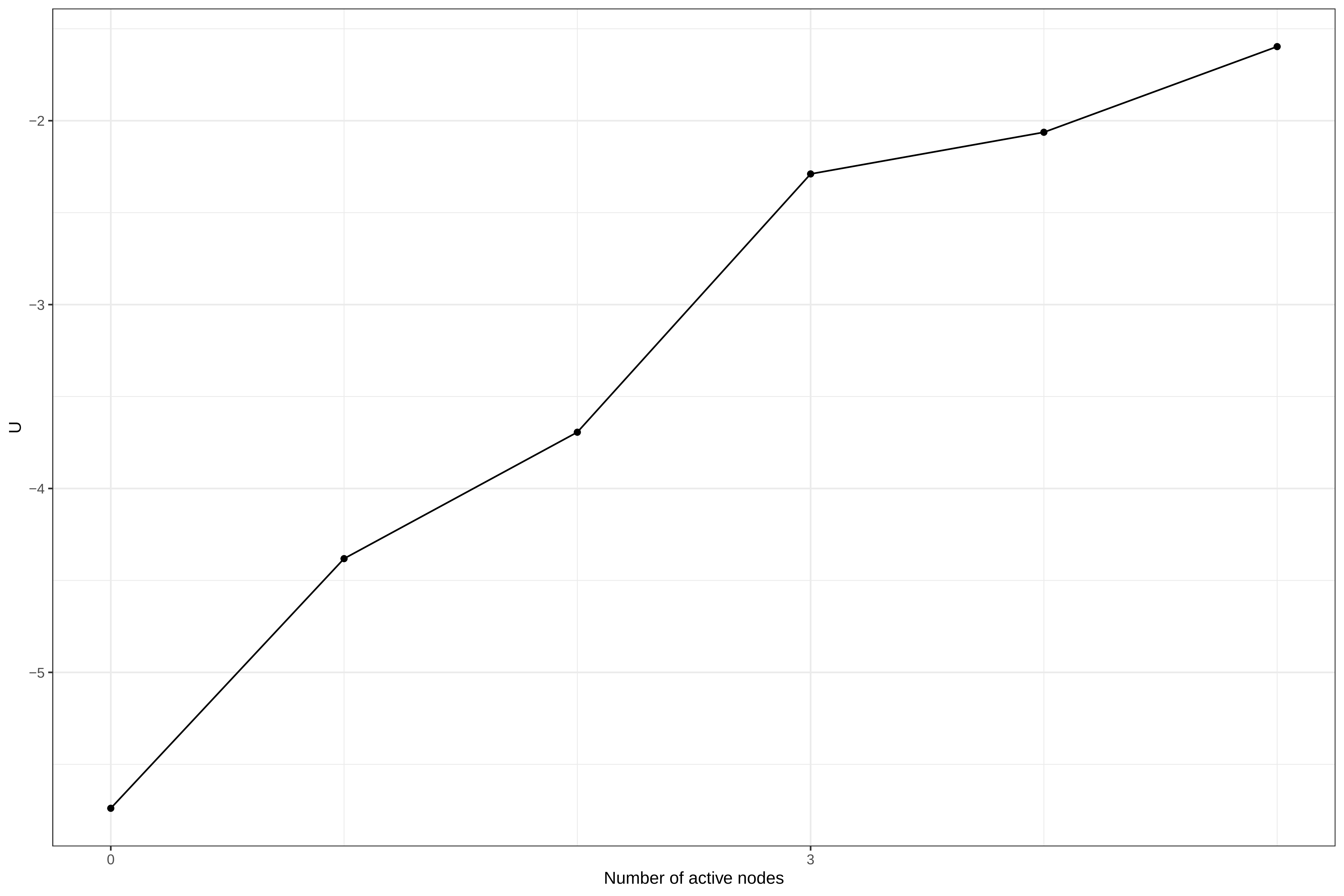

−2
−3
−4
−5
U
0
3
Number of active nodes

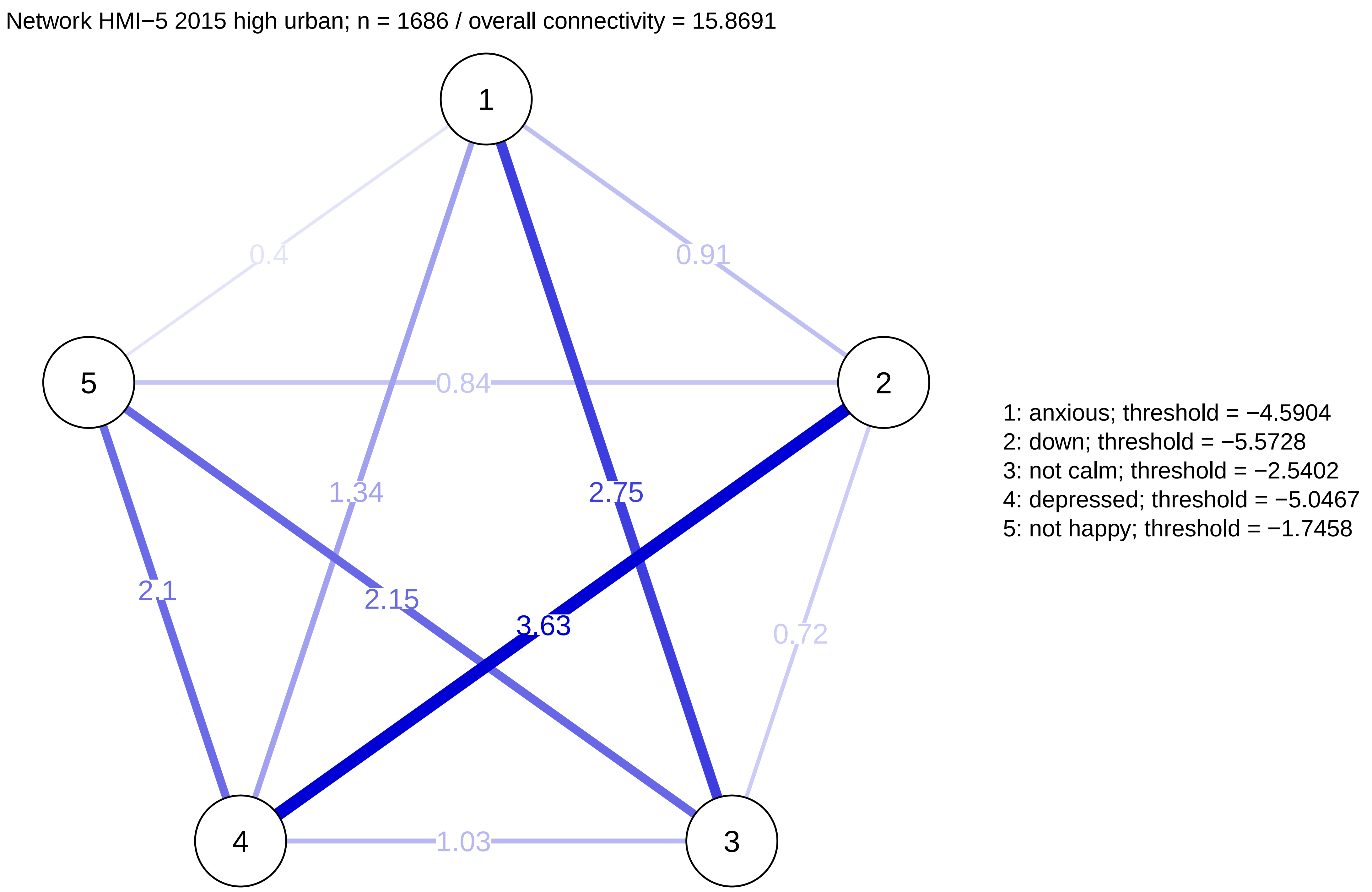
Network HMI−5 2015 high urban; n = 1686 / overall connectivity = 15.8691
1
2
3
4
5
0.4
0.91
0.84
1.34
2.75
2.1
2.15
3.63
0.72
1.03
1: anxious; threshold = −4.5904
2: down; threshold = −5.5728
3: not calm; threshold = −2.5402
4: depressed; threshold = −5.0467
5: not happy; threshold = −1.7458

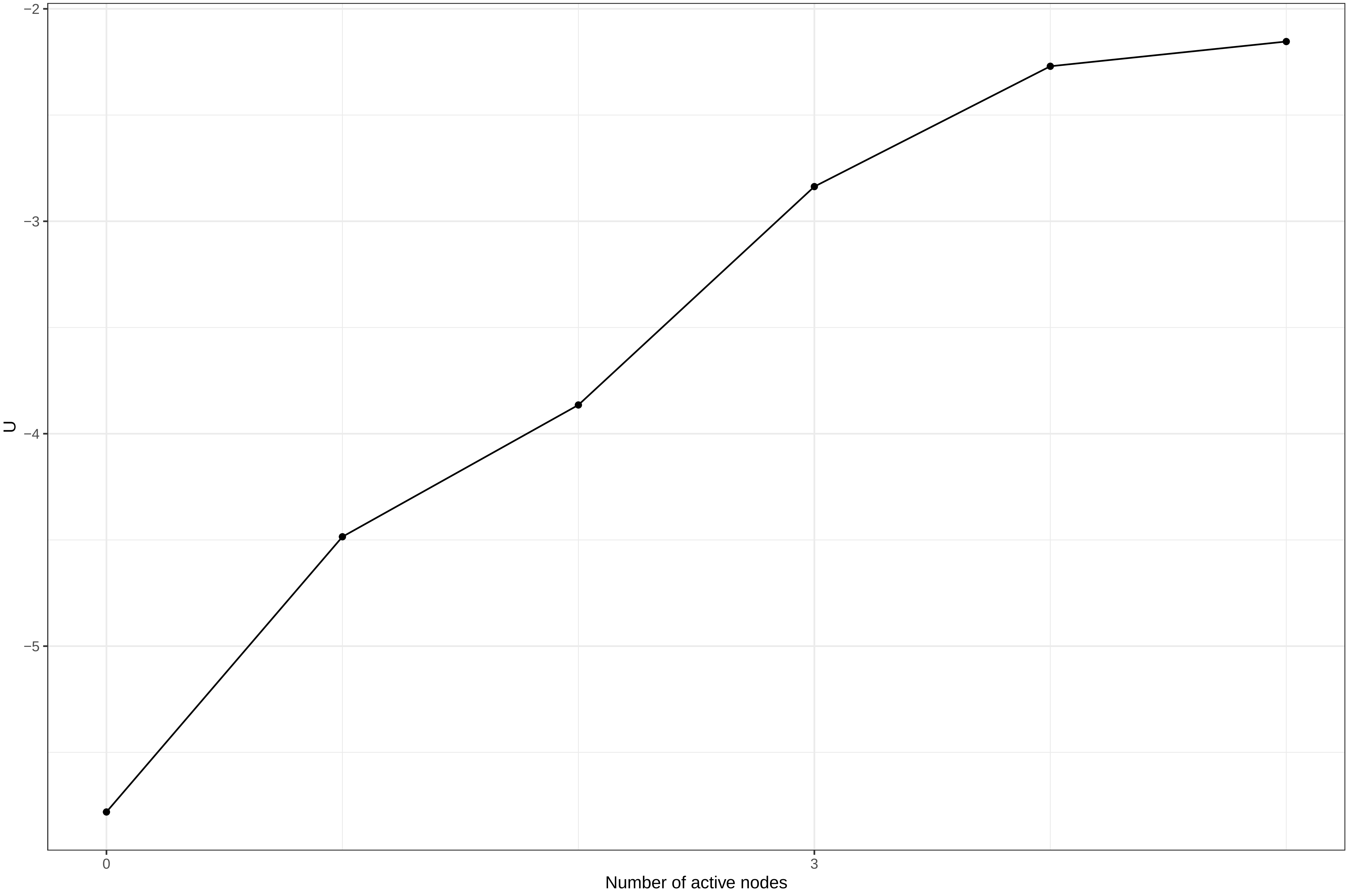

−2
−3
−4
−5
U
0
3
Number of active nodes

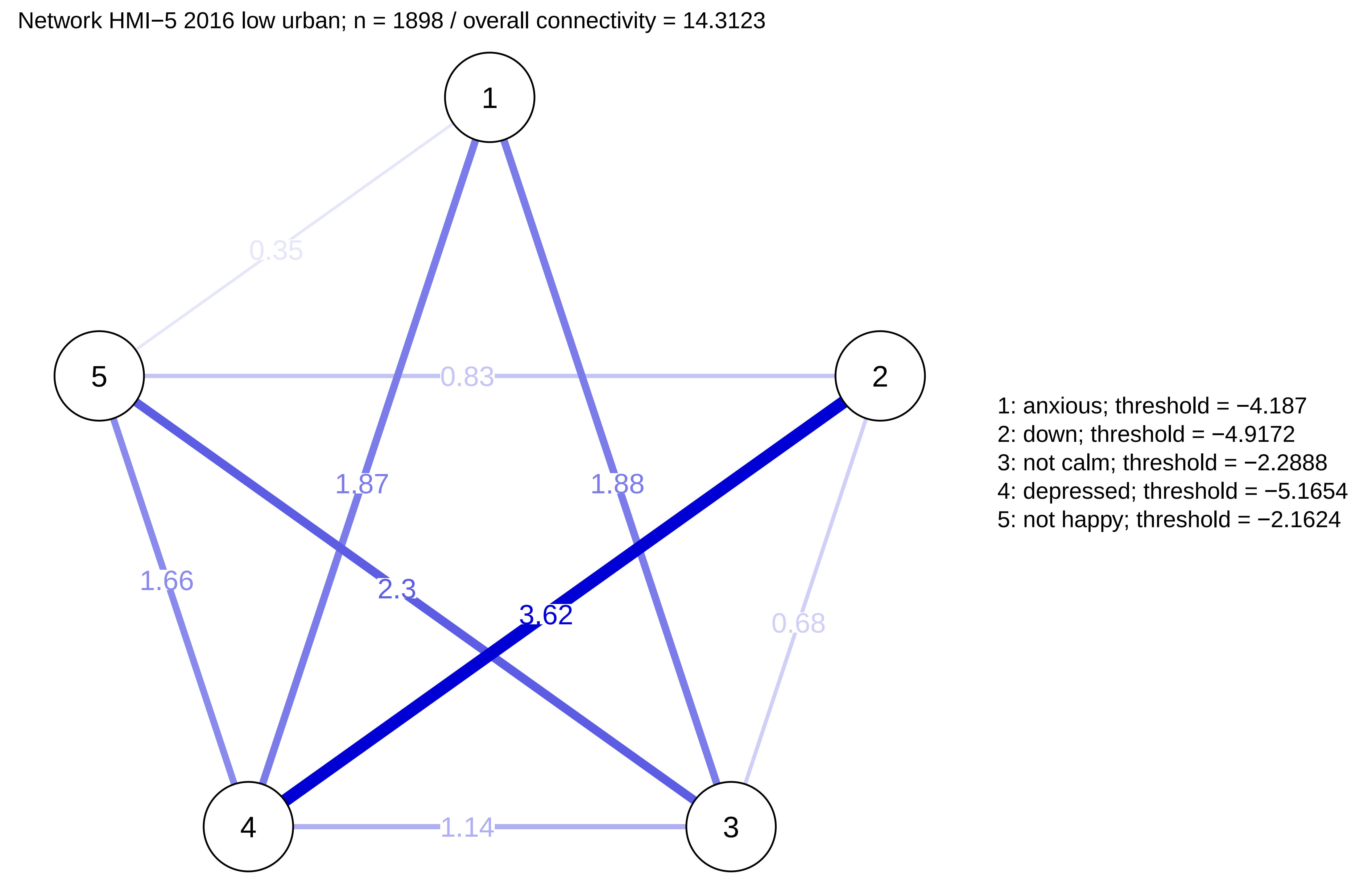
Network HMI−5 2016 low urban; n = 1898 / overall connectivity = 14.3123
1
2
3
4
5
0.35
0.83
1.87
1.88
1.66
2.3
3.62
0.68
1.14
1: anxious; threshold = −4.187
2: down; threshold = −4.9172
3: not calm; threshold = −2.2888
4: depressed; threshold = −5.1654
5: not happy; threshold = −2.1624

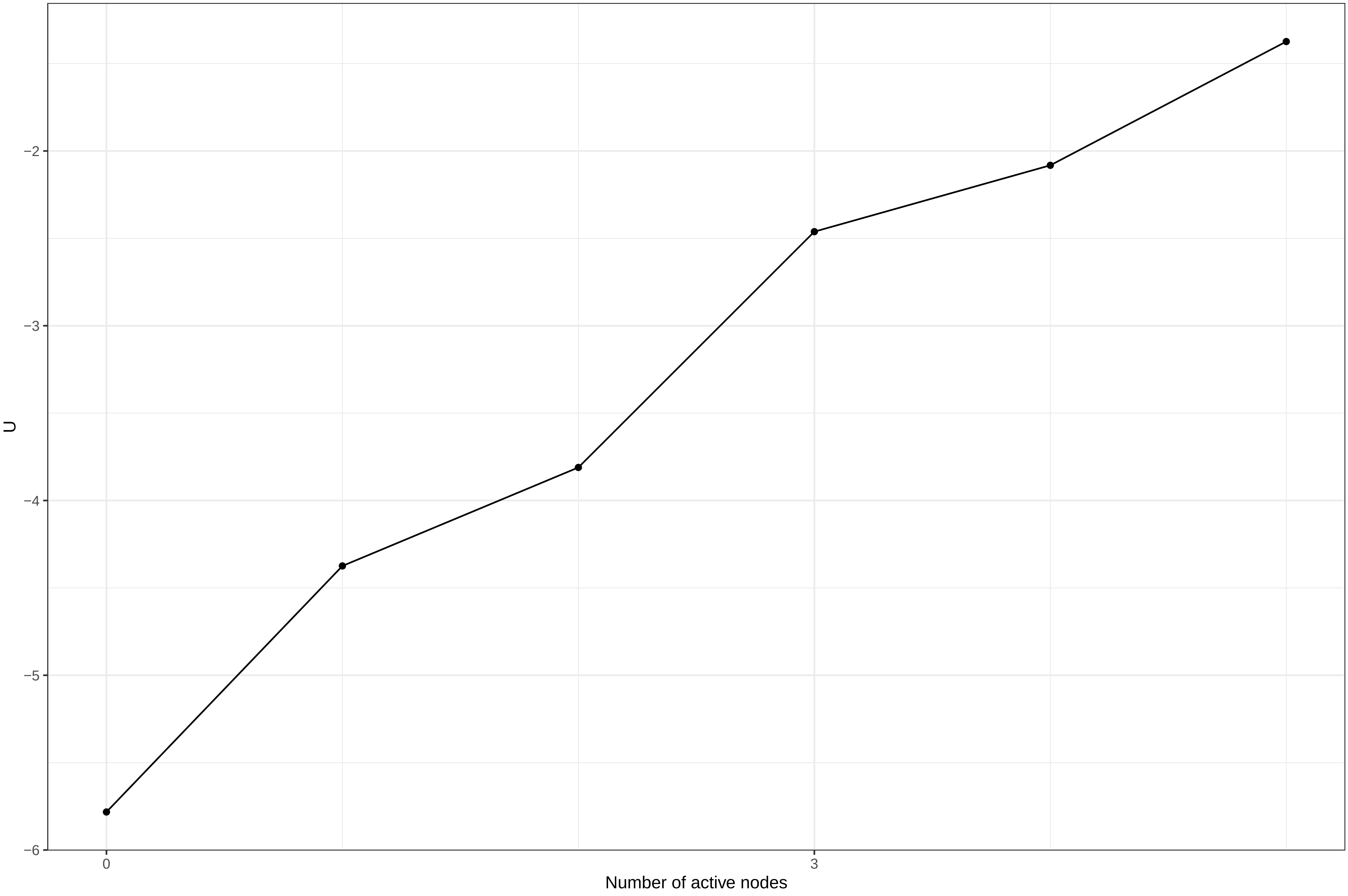

U
−2
−3
−4
−5
−6
0
3
Number of active nodes

Network HMI−5 2016 mid urban; n = 1193 / overall connectivity = 15.6078

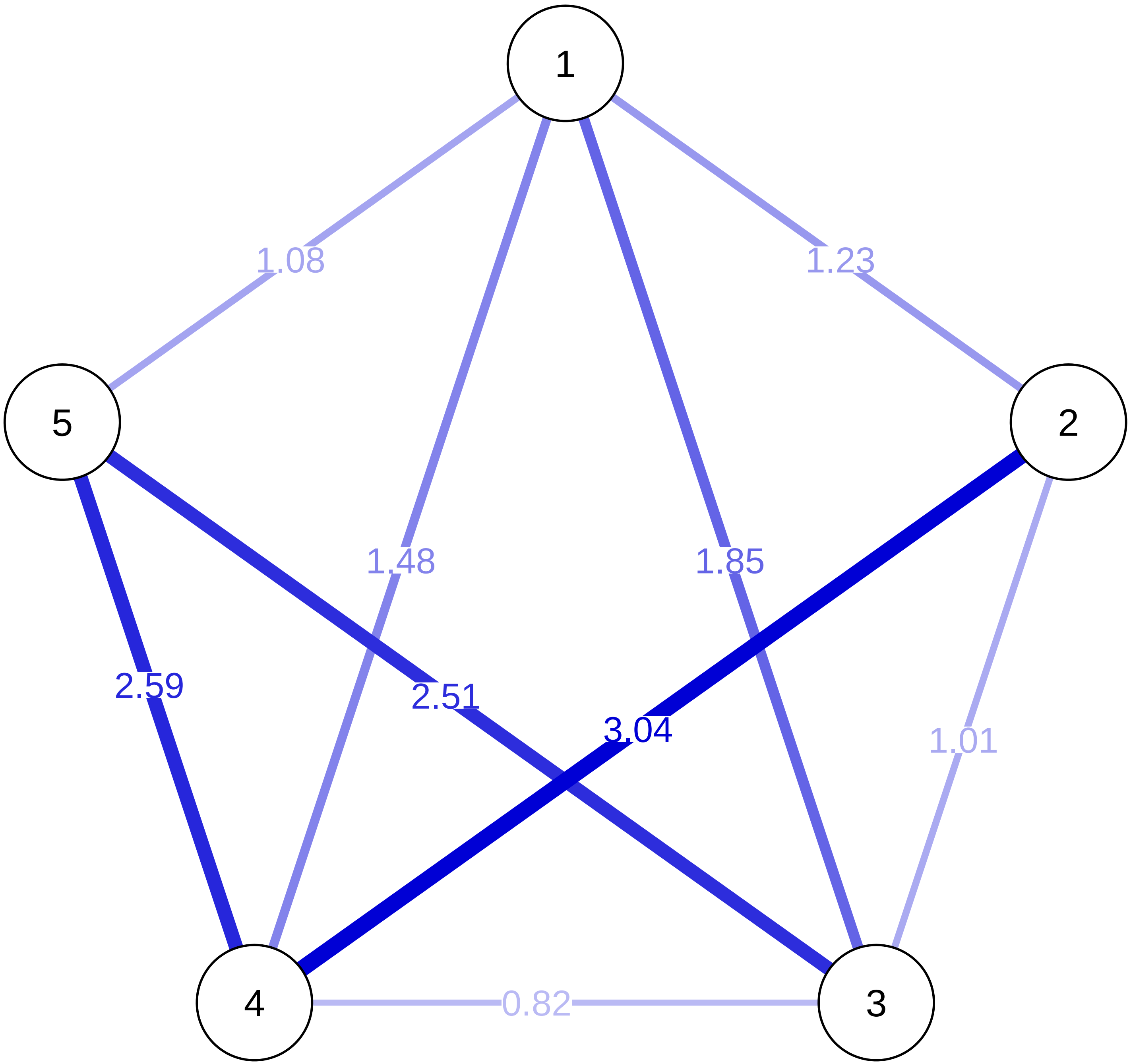

1: anxious; threshold = −4.6446
2: down; threshold = −4.3967
3: not calm; threshold = −2.4968
4: depressed; threshold = −5.554
5: not happy; threshold = −2.137

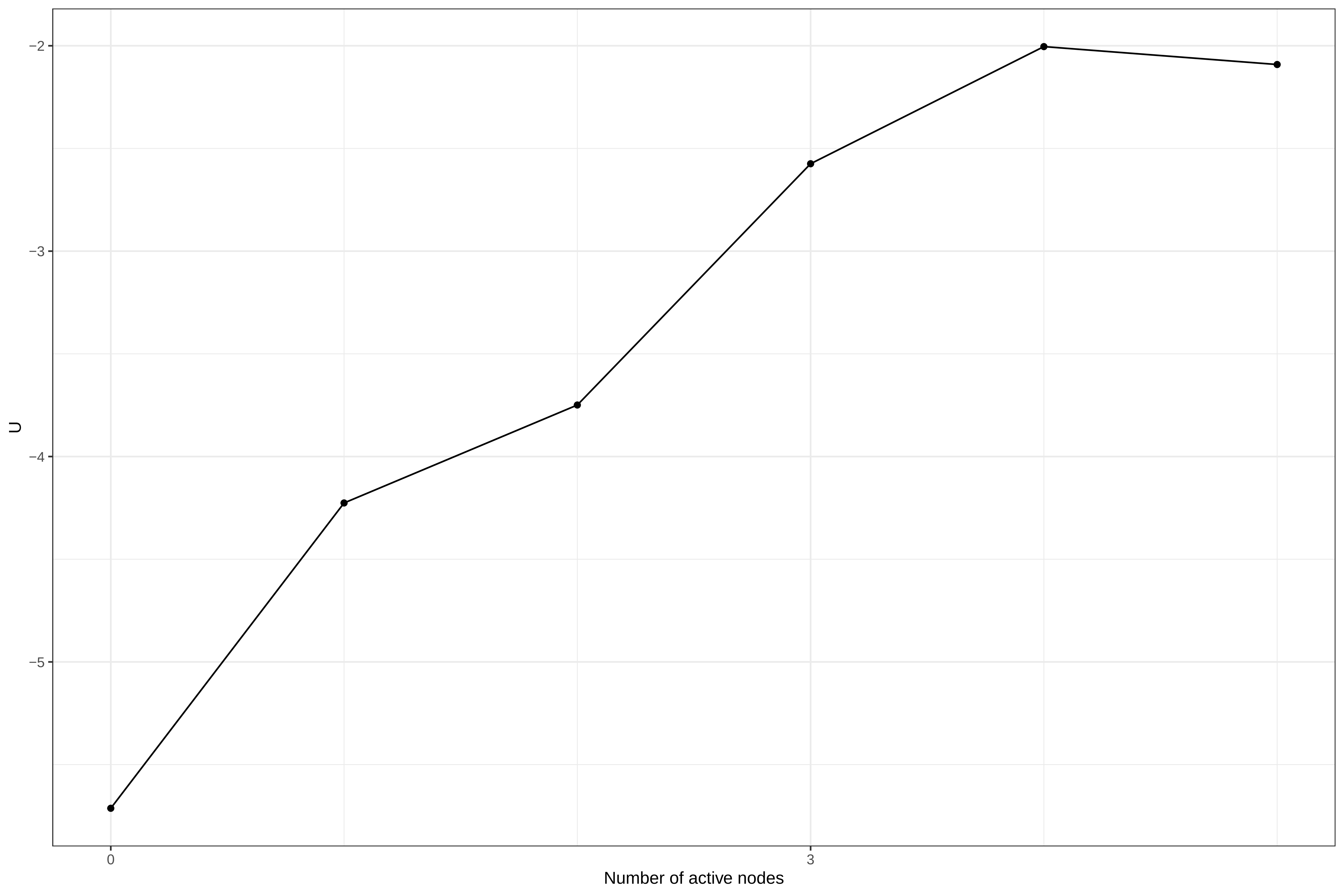
−2
−3
−4
−5
U
0
3
Number of active nodes

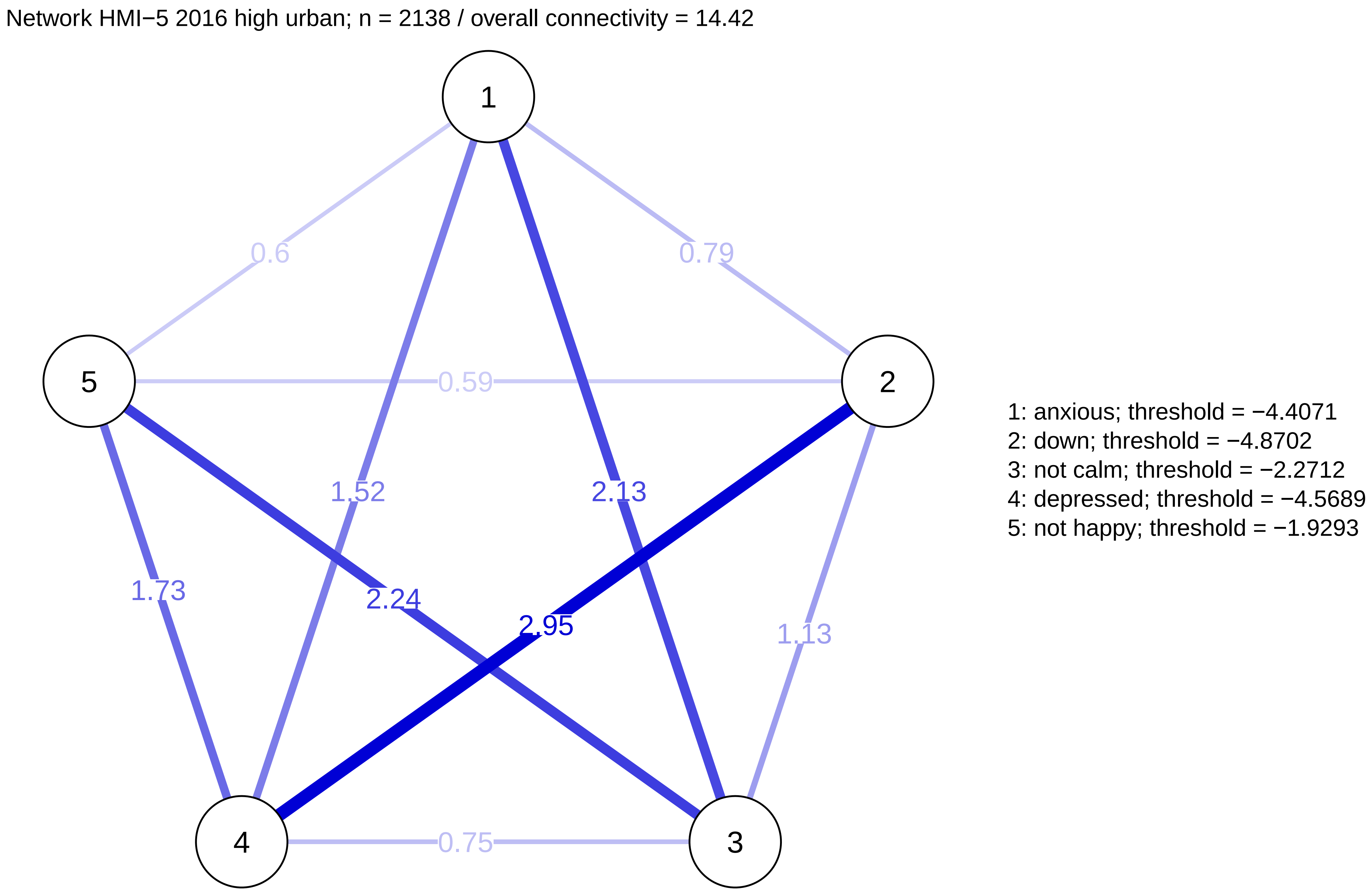

Network HMI−5 2016 high urban; n = 2138 / overall connectivity = 14.42
1
2
3
4
5
0.6
0.79
0.59
1.52
2.13
1.73
2.24
2.95
1.13
0.75
1: anxious; threshold = −4.4071
2: down; threshold = −4.8702
3: not calm; threshold = −2.2712
4: depressed; threshold = −4.5689
5: not happy; threshold = −1.9293

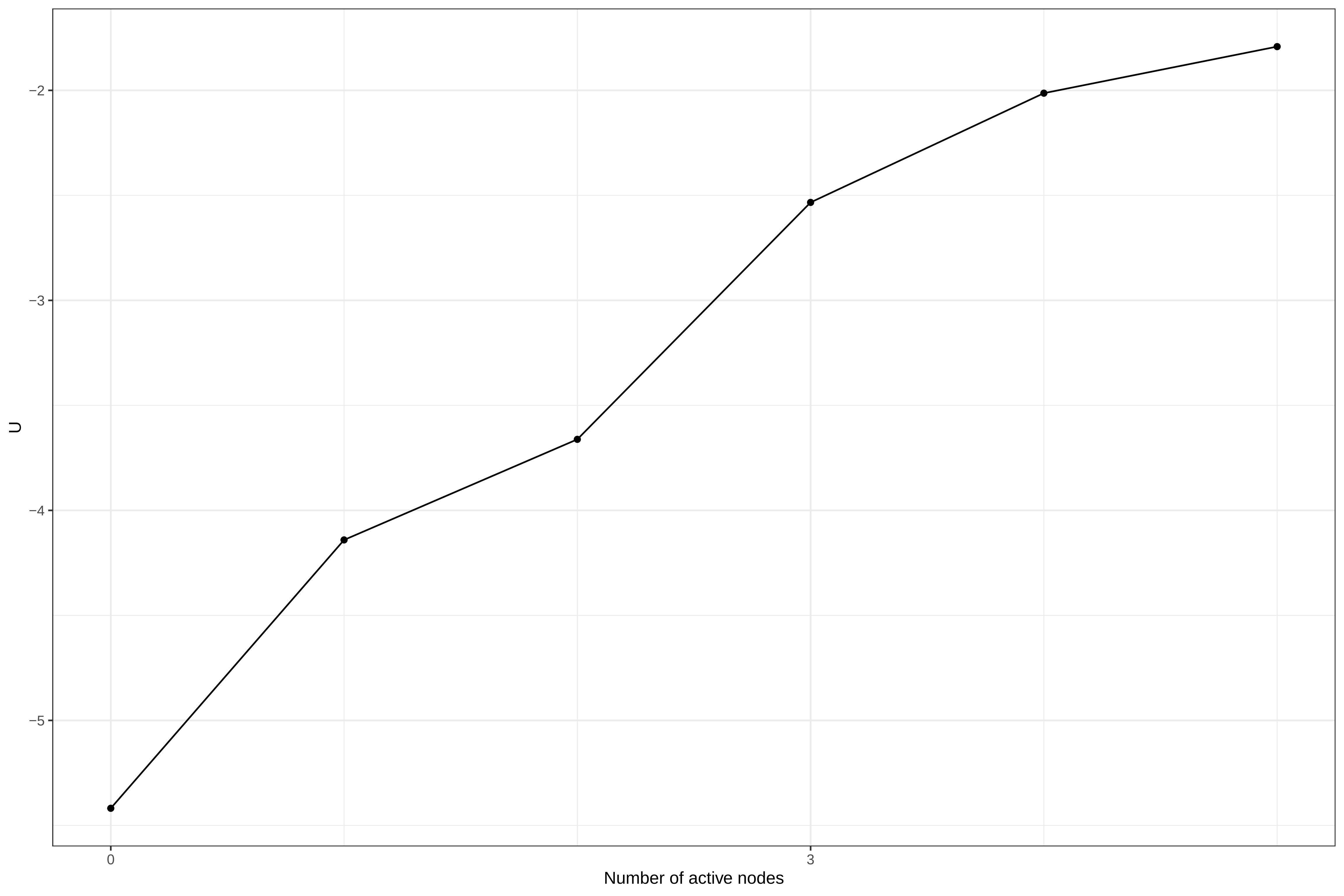

−2
−3
−4
−5
U
0
3
Number of active nodes

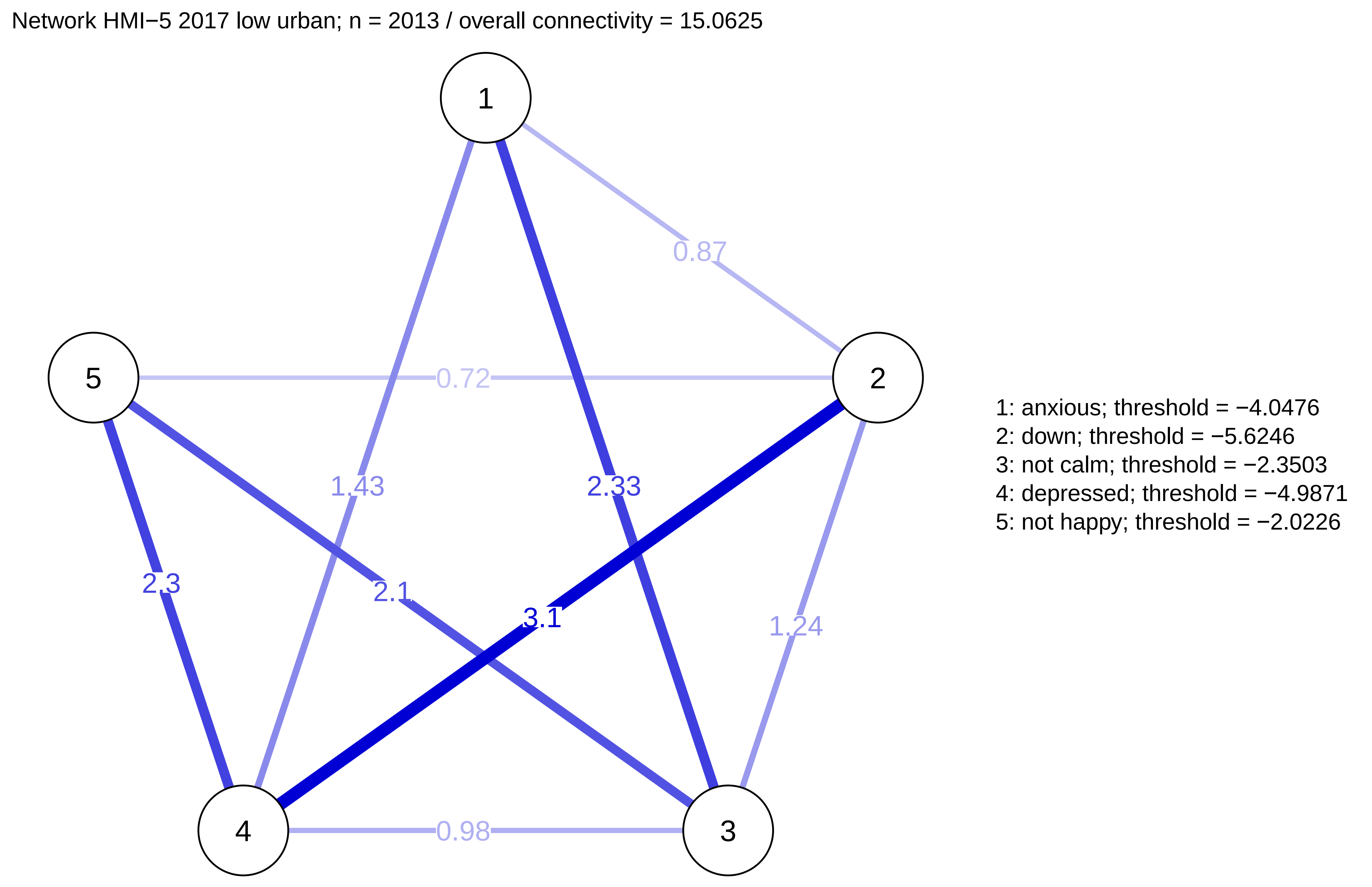
Network HMI−5 2017 low urban; n = 2013 / overall connectivity = 15.0625
1
2
3
4
5
0.87
0.72
1.43
2.33
2.3
2.1
3.1
1.24
0.98
1: anxious; threshold = −4.0476
2: down; threshold = −5.6246
3: not calm; threshold = −2.3503
4: depressed; threshold = −4.9871
5: not happy; threshold = −2.0226

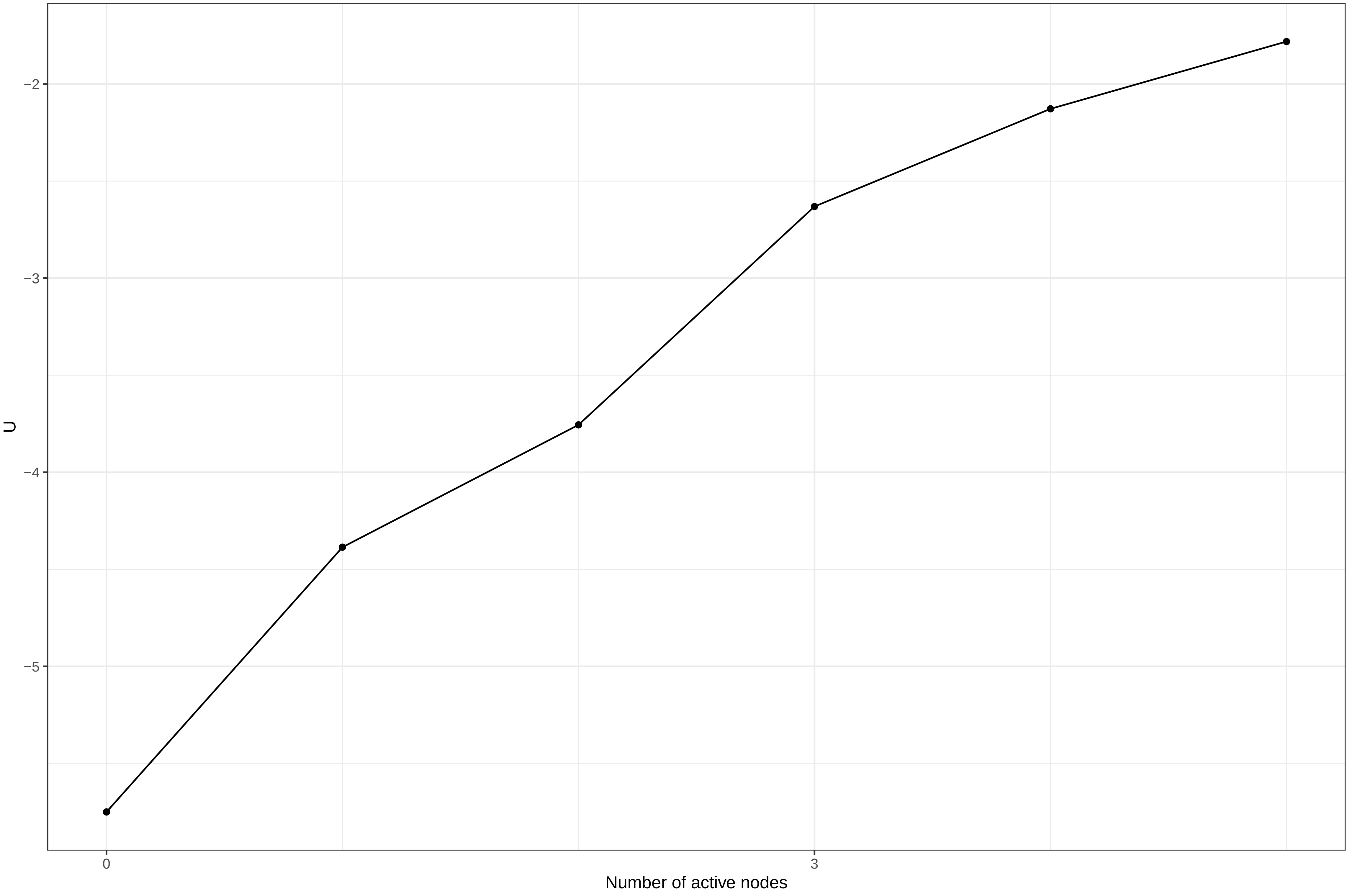

−2
−3
−4
−5
U
0
3
Number of active nodes

Network HMI−5 2017 mid urban; n = 1294 / overall connectivity = 14.8887

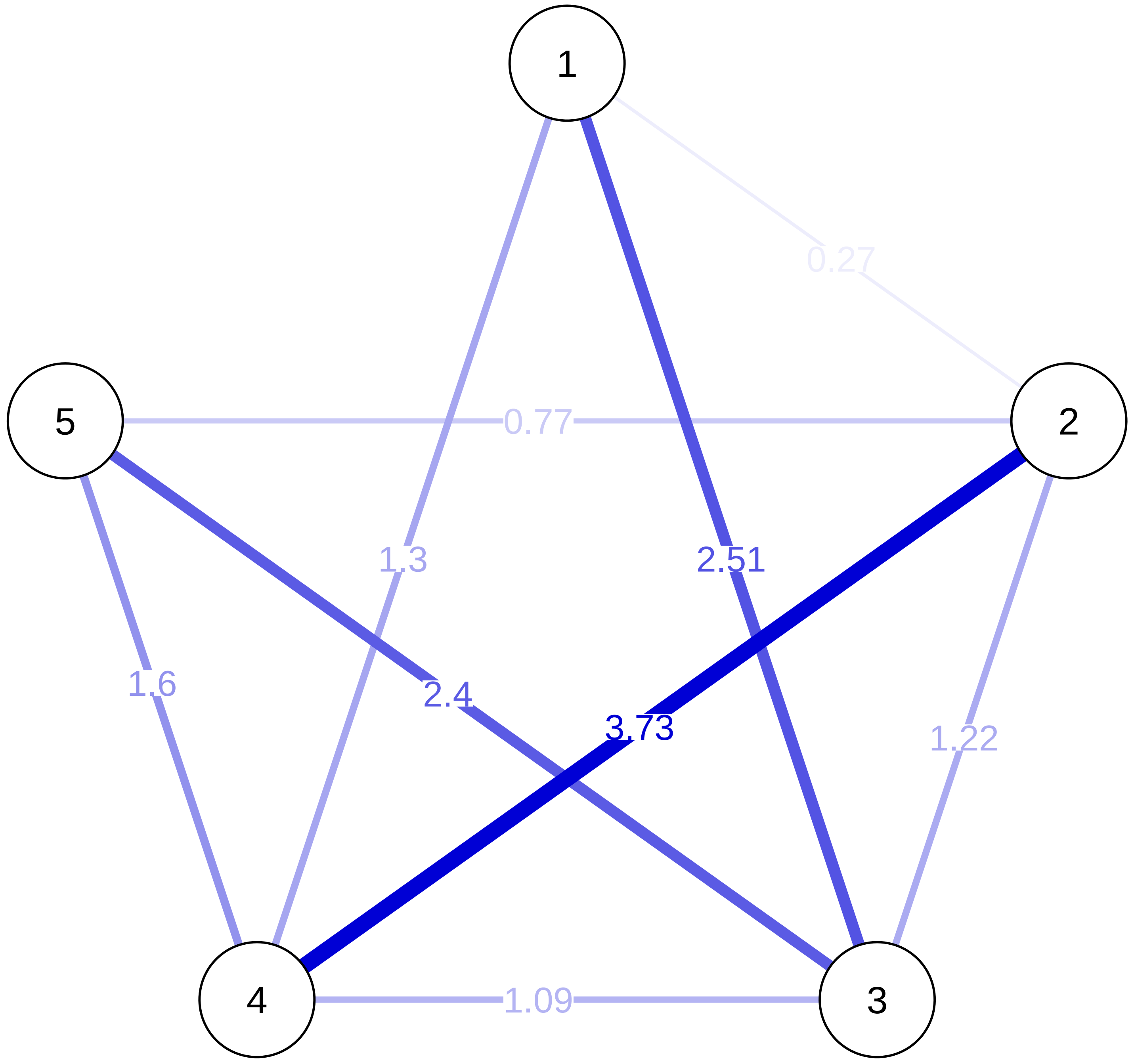

1: anxious; threshold = −3.7974
2: down; threshold = −5.5965
3: not calm; threshold = −2.5674
4: depressed; threshold = −4.8557
5: not happy; threshold = −2.0423

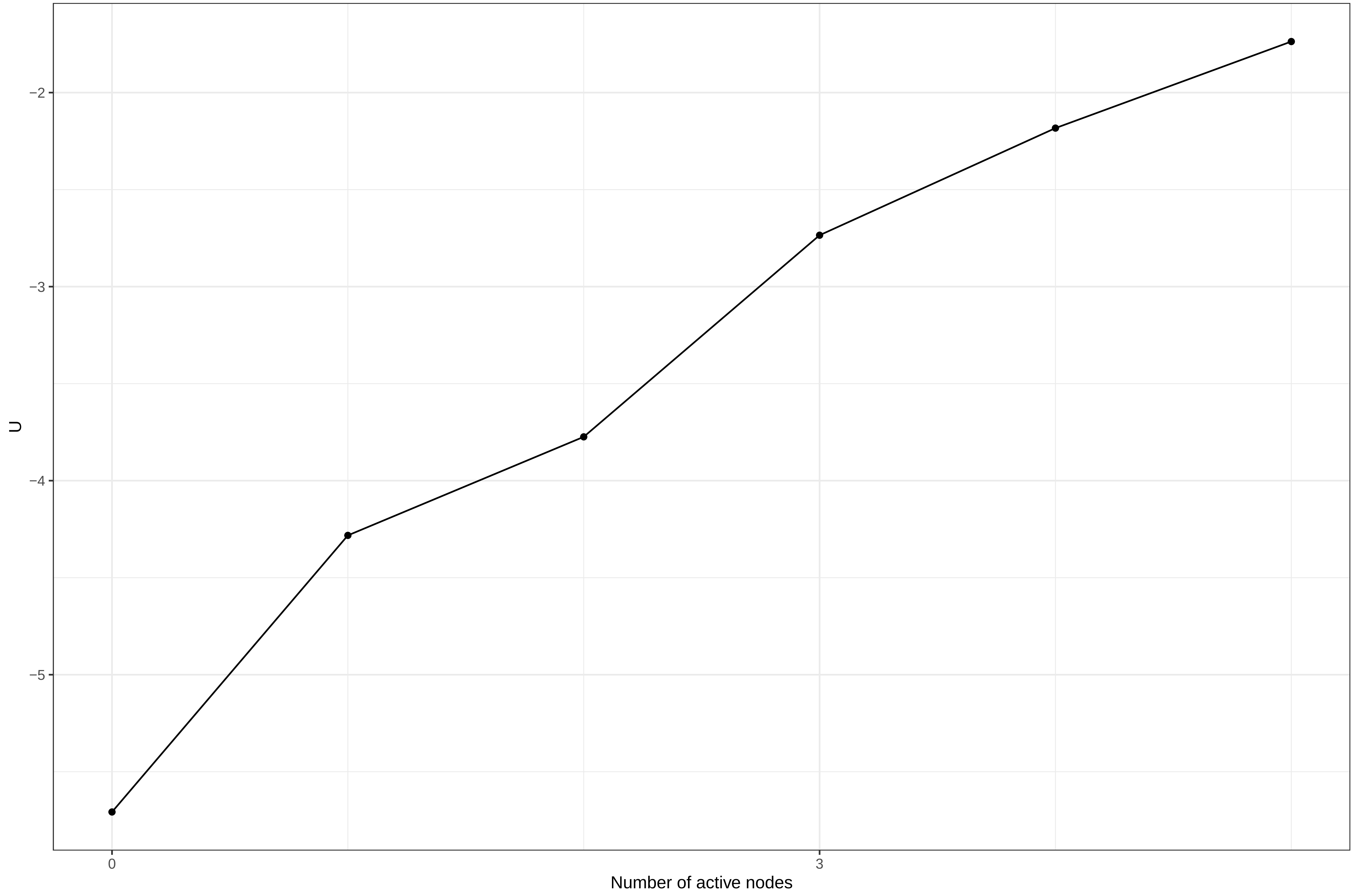
−2
−3
−4
−5
U
0
3
Number of active nodes

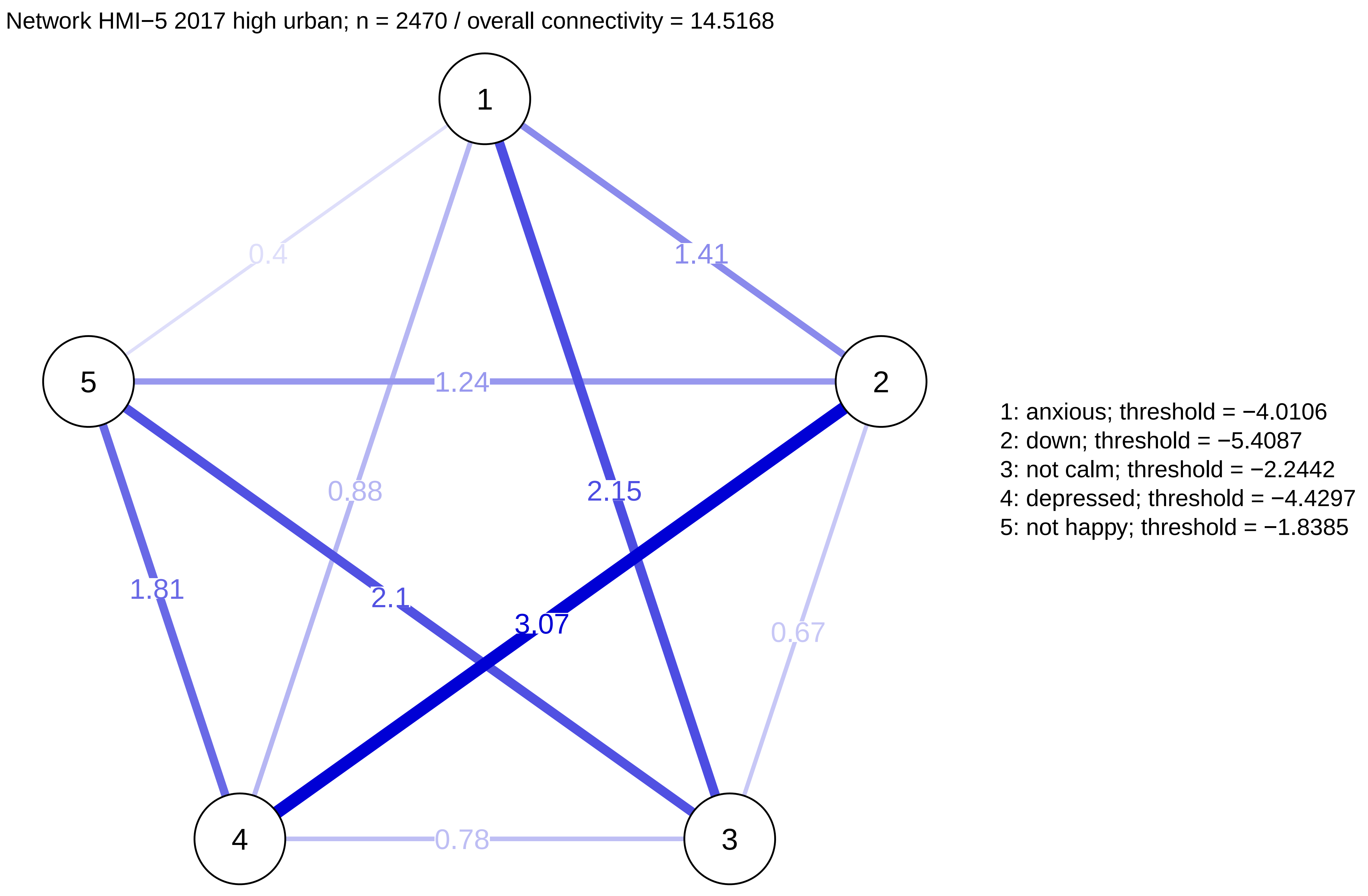
Network HMI−5 2017 high urban; n = 2470 / overall connectivity = 14.5168
1
2
3
4
5
0.4
1.41
1.24
0.88
2.15
1.81
2.1
3.07
0.67
0.78
1: anxious; threshold = −4.0106
2: down; threshold = −5.4087
3: not calm; threshold = −2.2442
4: depressed; threshold = −4.4297
5: not happy; threshold = −1.8385

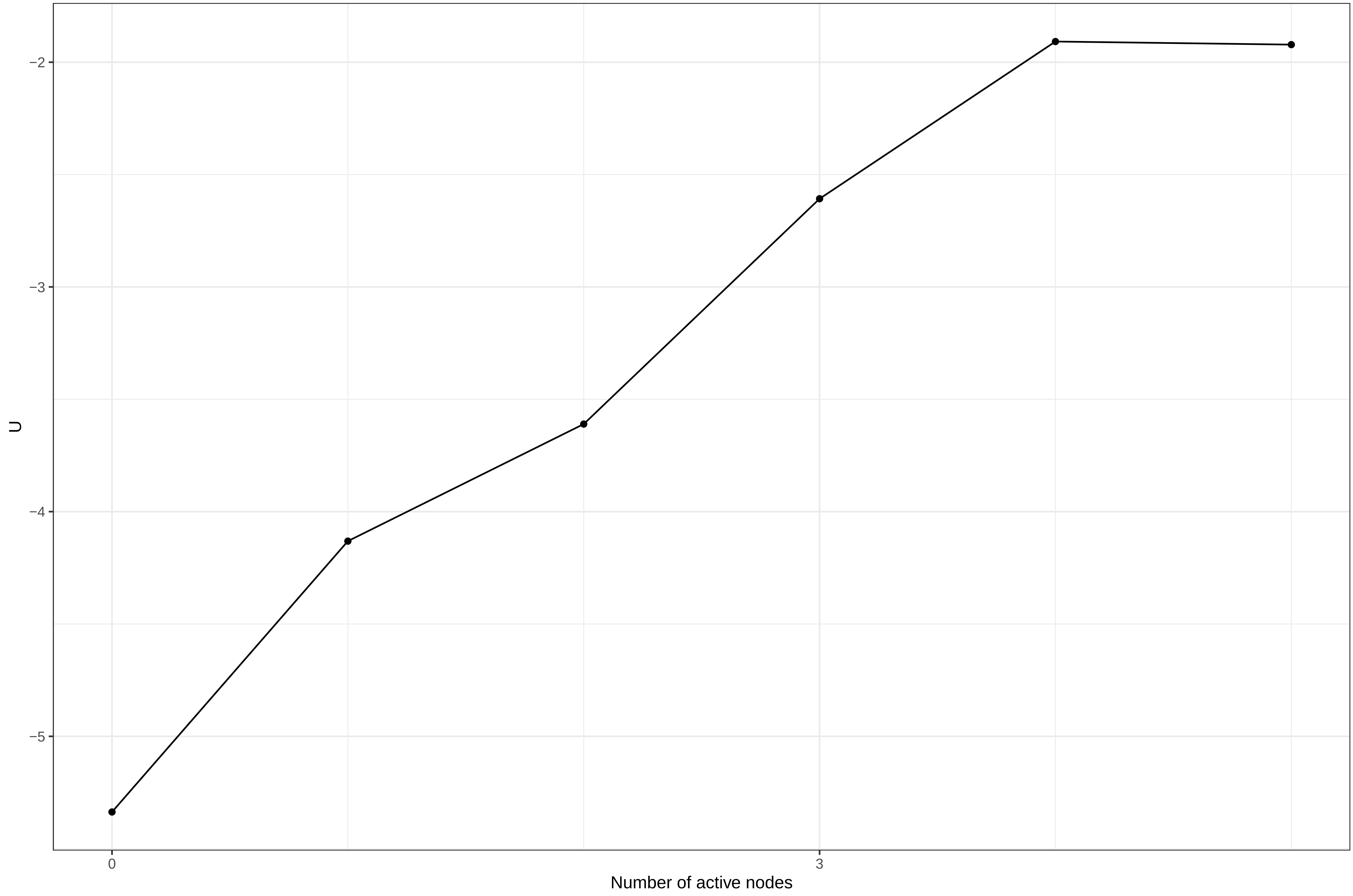

−2
−3
−4
−5
U
0
3
Number of active nodes

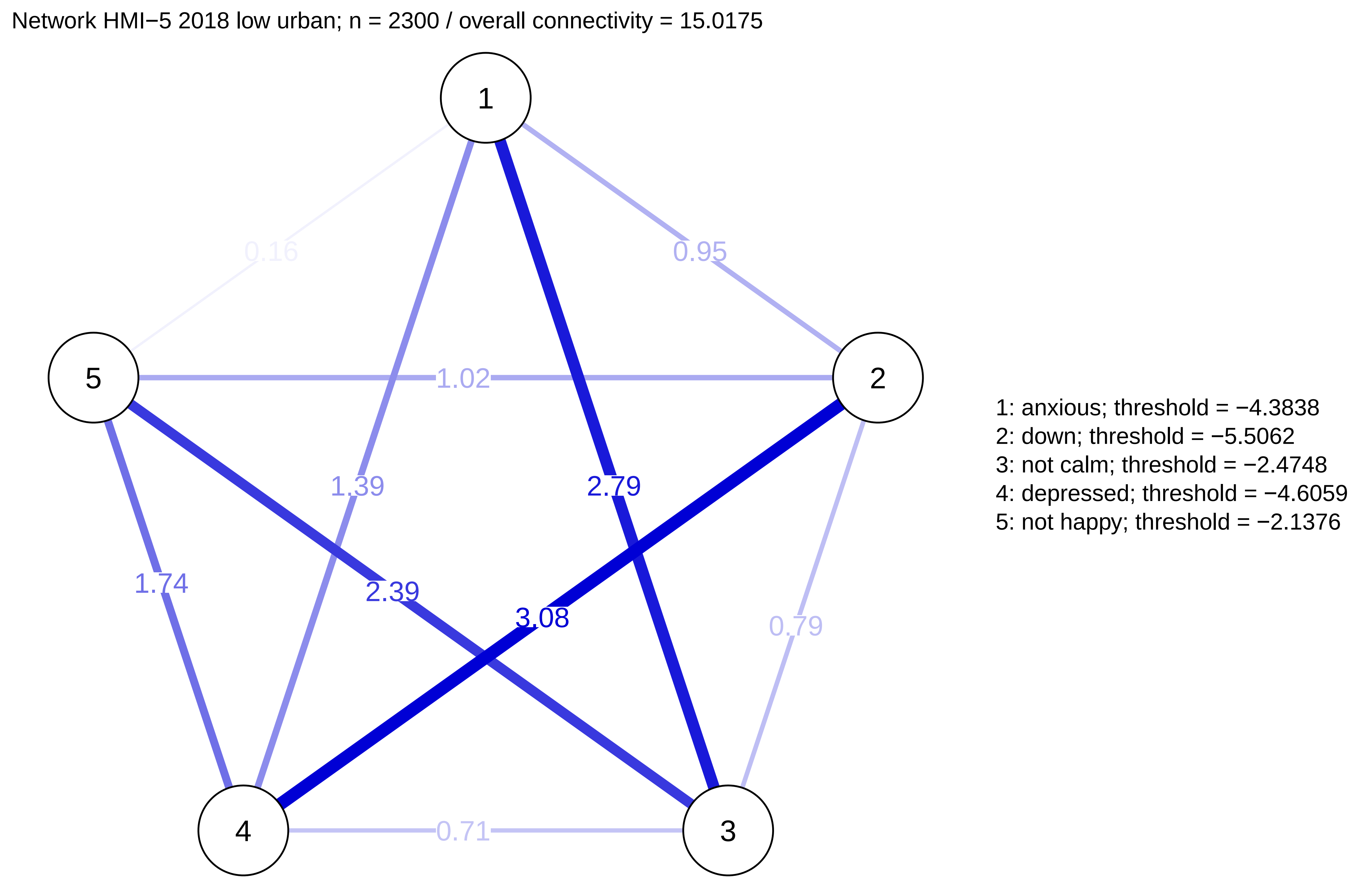
Network HMI−5 2018 low urban; n = 2300 / overall connectivity = 15.0175
1
2
3
4
5
0.16
0.95
1.02
1.39
2.79
1.74
2.39
3.08
0.79
0.71
1: anxious; threshold = −4.3838
2: down; threshold = −5.5062
3: not calm; threshold = −2.4748
4: depressed; threshold = −4.6059
5: not happy; threshold = −2.1376

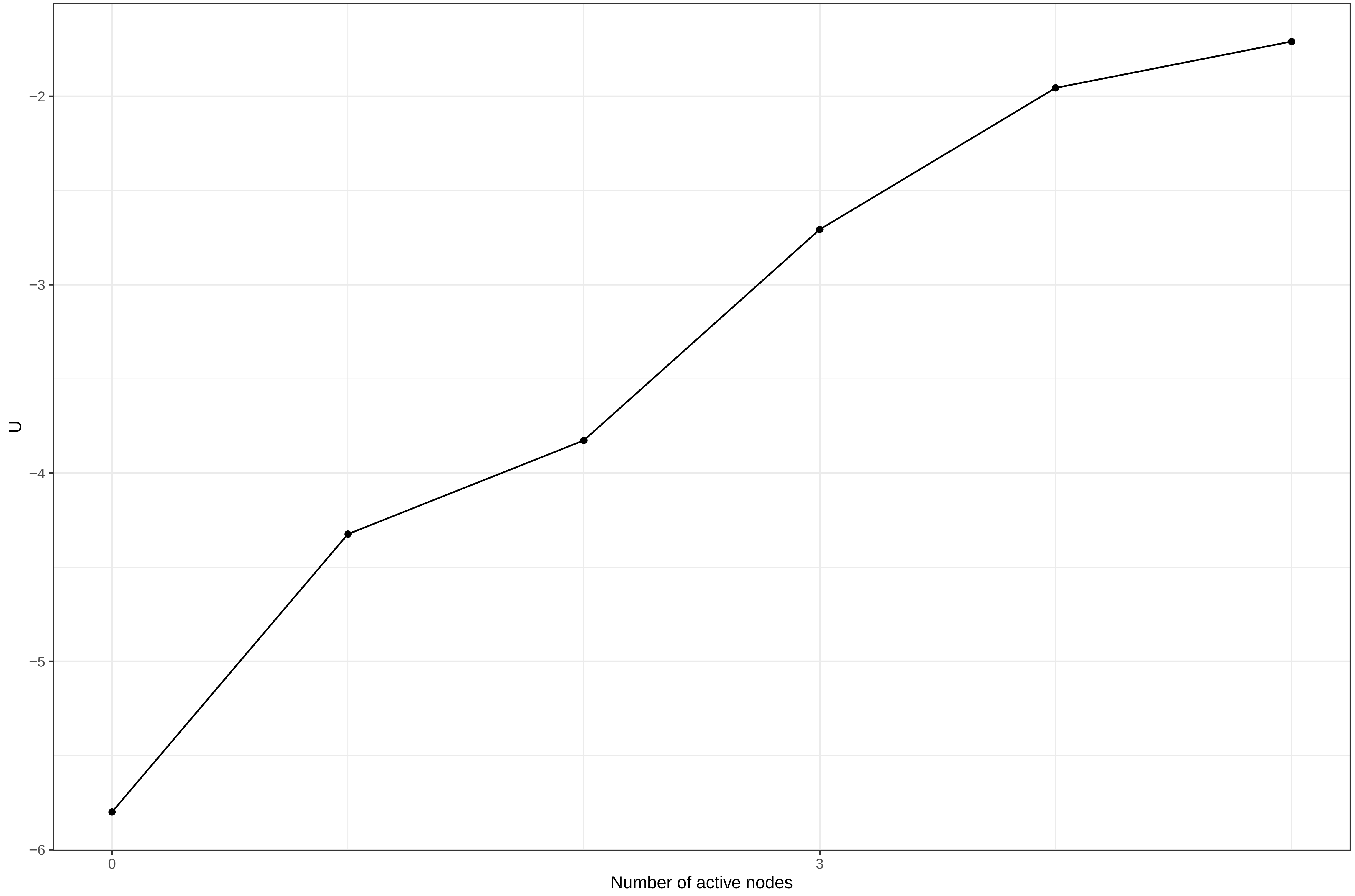

−2
−3
−4
−5
−6
U
0
3
Number of active nodes

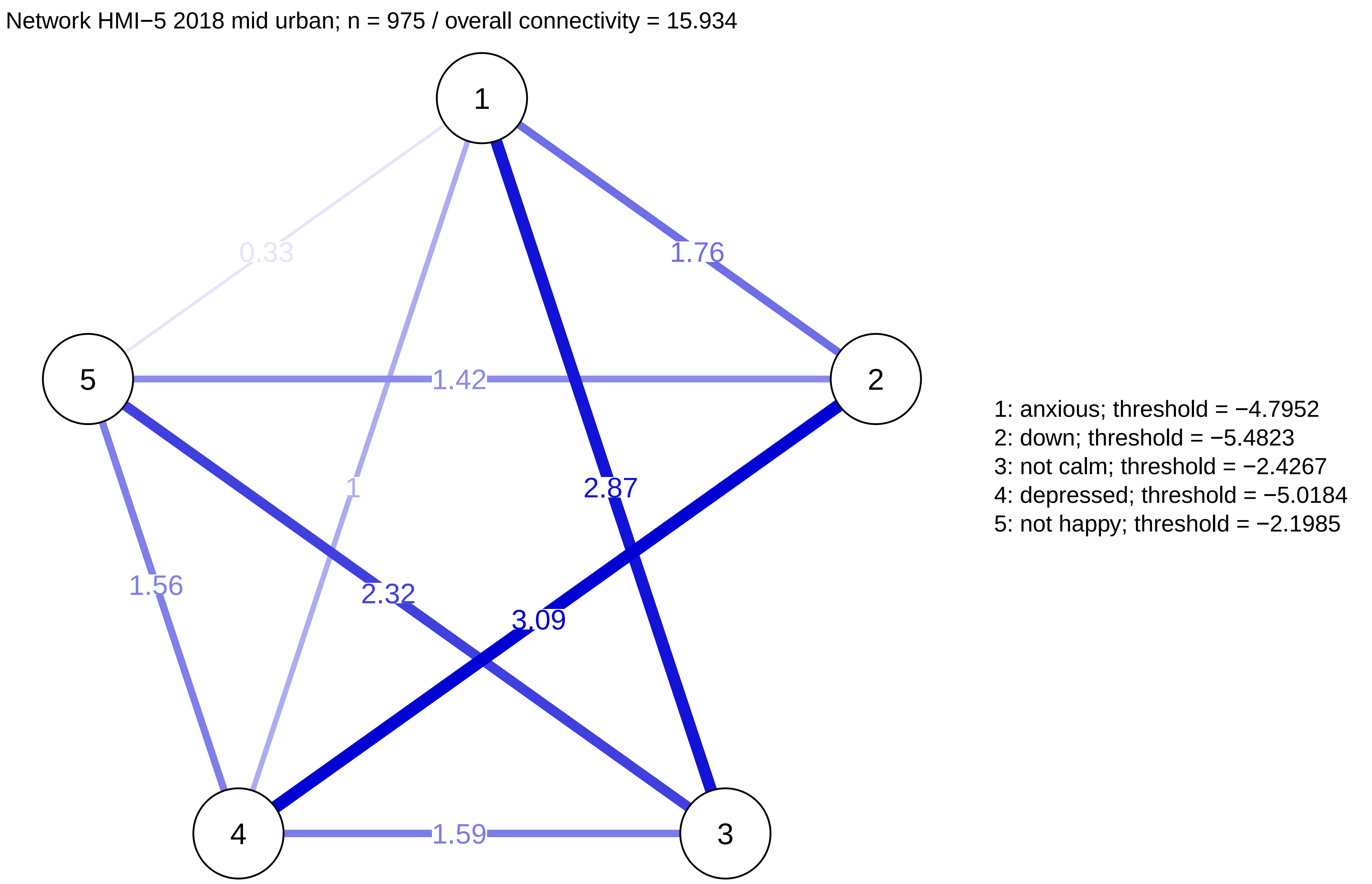
Network HMI−5 2018 mid urban; n = 975 / overall connectivity = 15.934
1
2
3
4
5
0.33
1.76
1.42
1
2.87
1.56
2.32
3.09
1.59
1: anxious; threshold = −4.7952
2: down; threshold = −5.4823
3: not calm; threshold = −2.4267
4: depressed; threshold = −5.0184
5: not happy; threshold = −2.1985

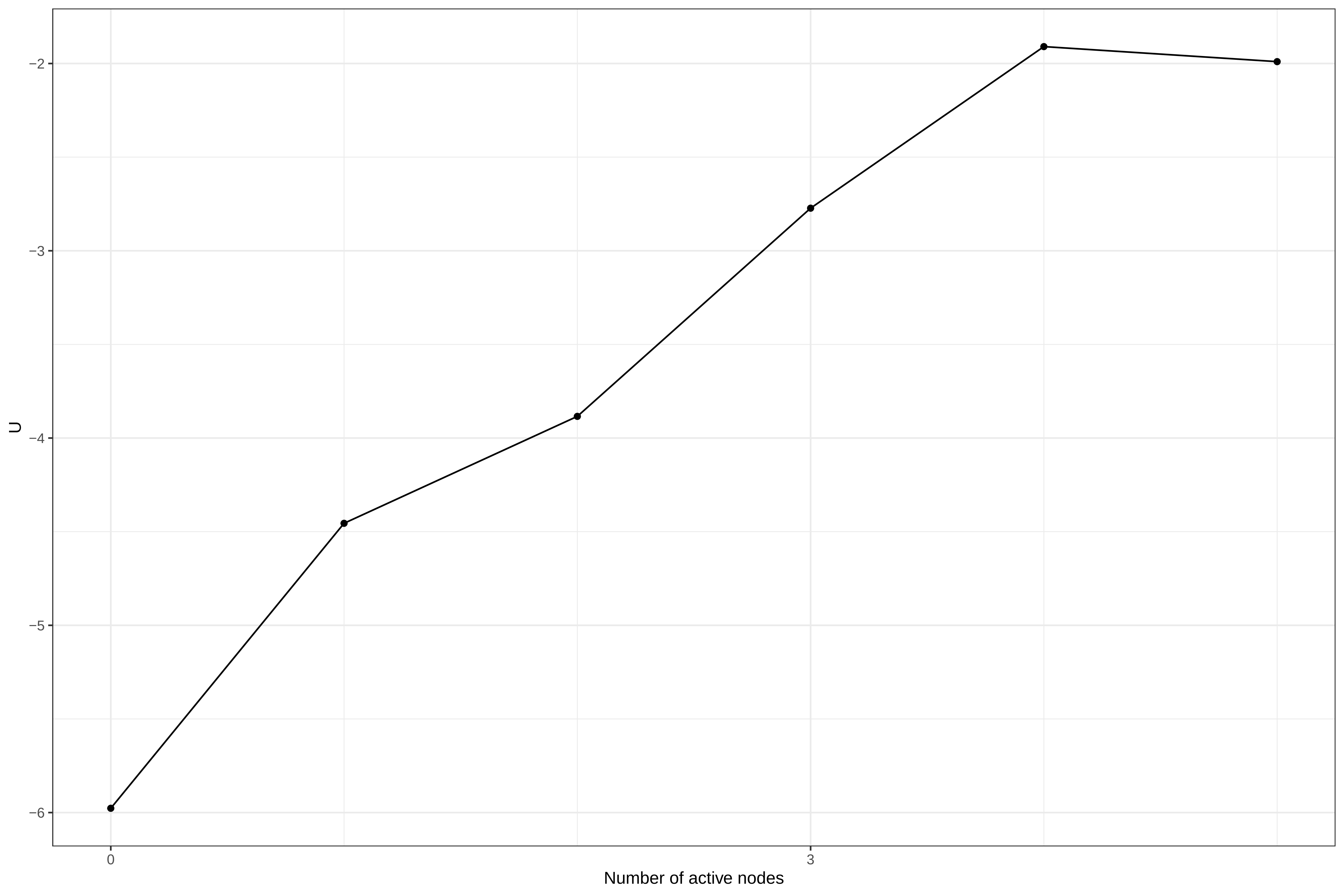

U
−2
−3
−4
−5
−6
0
3
Number of active nodes

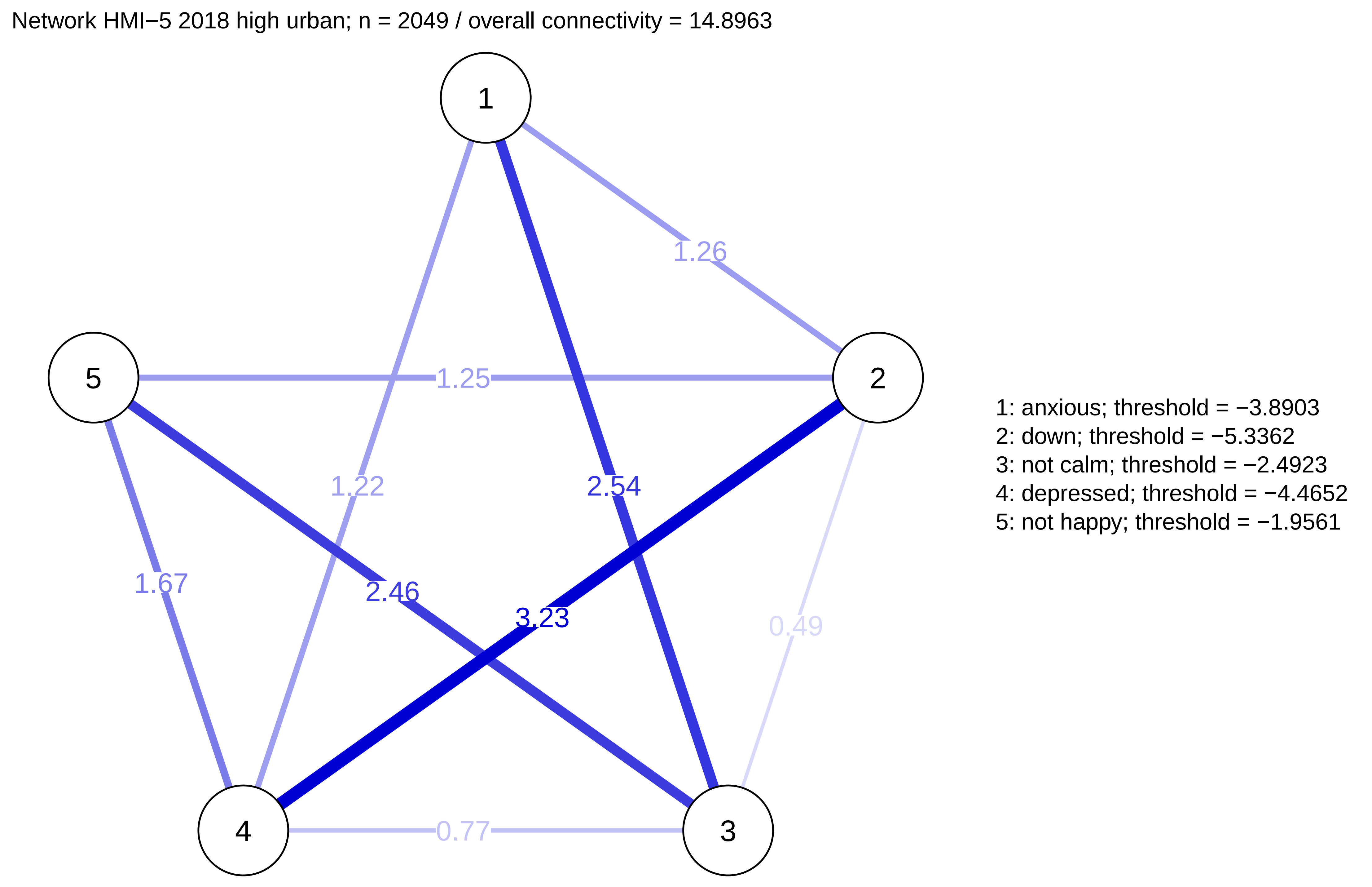
Network HMI−5 2018 high urban; n = 2049 / overall connectivity = 14.8963
1
2
3
4
5
1.26
1.25
1.22
2.54
1.67
2.46
3.23
0.49
0.77
1: anxious; threshold = −3.8903
2: down; threshold = −5.3362
3: not calm; threshold = −2.4923
4: depressed; threshold = −4.4652
5: not happy; threshold = −1.9561

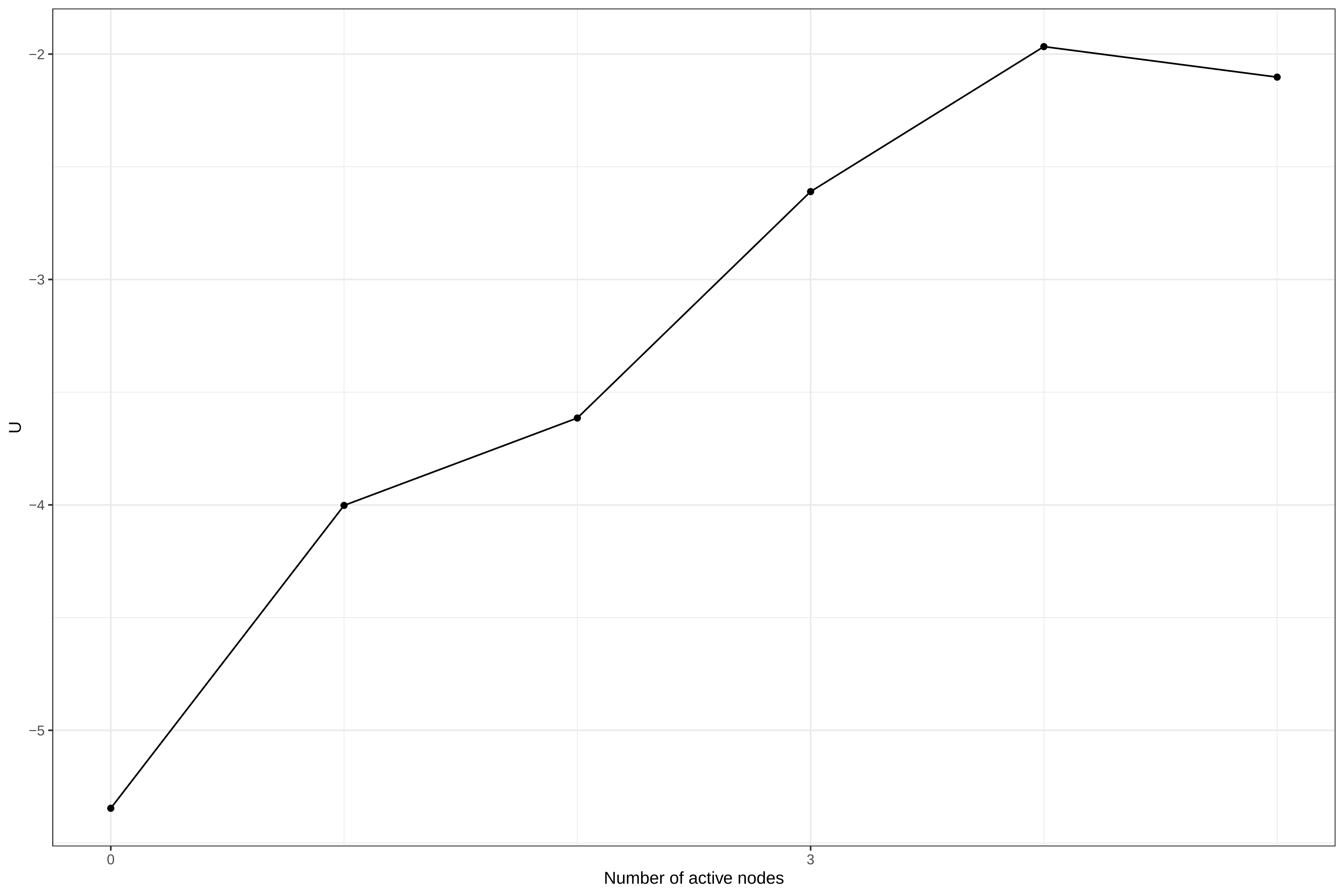

−2
−3
−4
−5
U
0
3
Number of active nodes

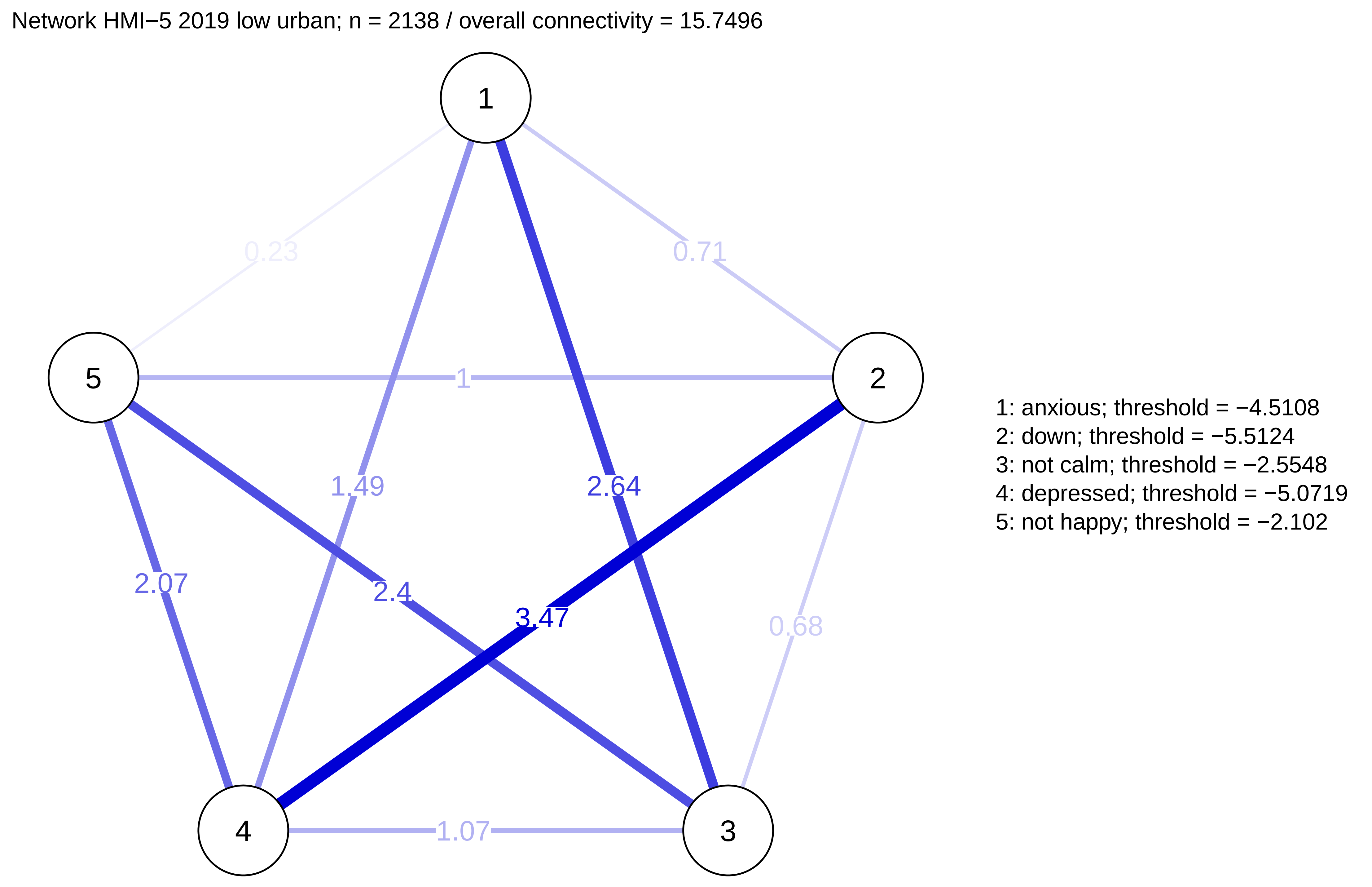
Network HMI−5 2019 low urban; n = 2138 / overall connectivity = 15.7496
1
2
3
4
5
0.23
0.71
1
1.49
2.64
2.07
2.4
3.47
0.68
1.07
1: anxious; threshold = −4.5108
2: down; threshold = −5.5124
3: not calm; threshold = −2.5548
4: depressed; threshold = −5.0719
5: not happy; threshold = −2.102

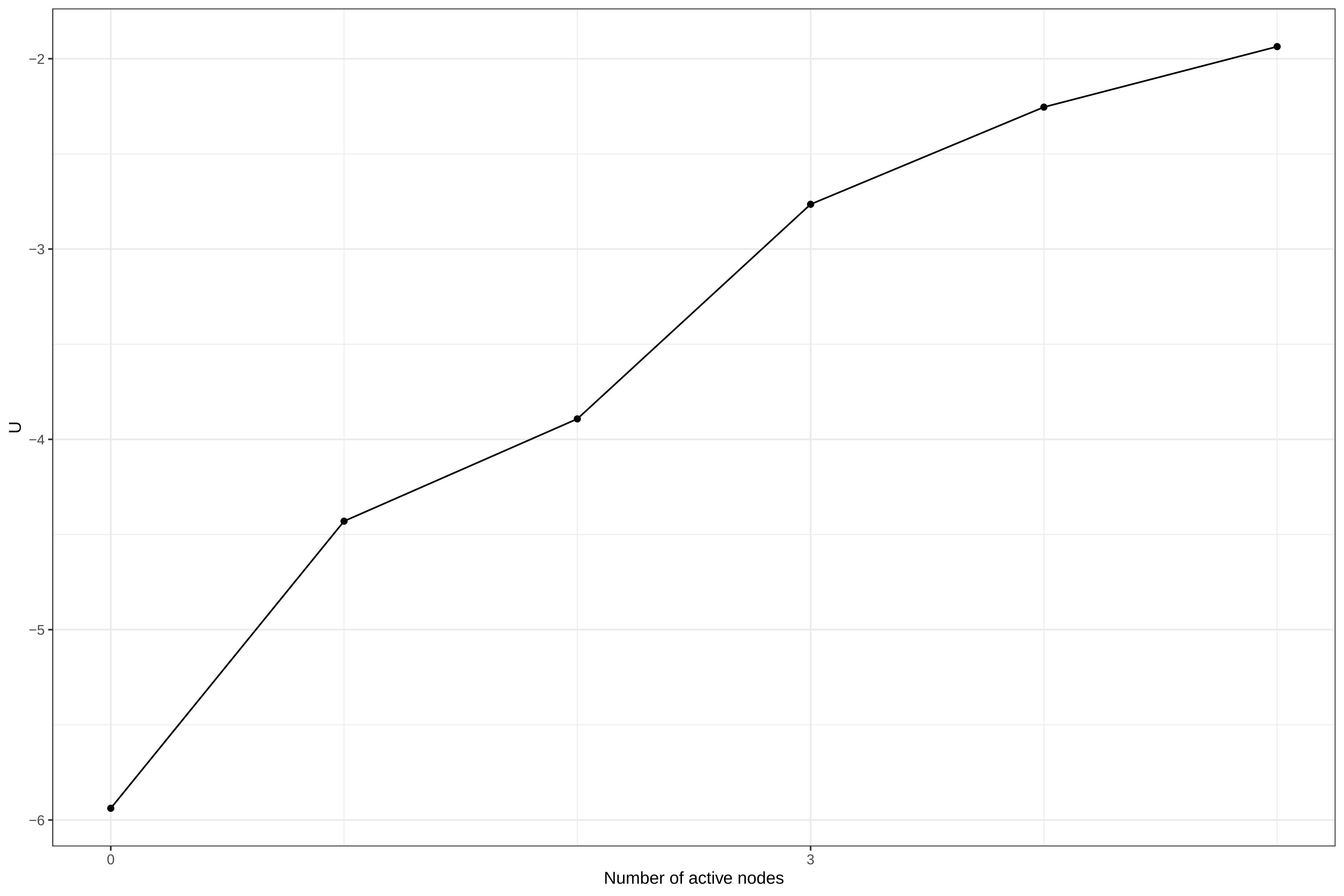

−2
−3
−4
−5
−6
U
0
3
Number of active nodes

Network HMI−5 2019 mid urban; n = 923 / overall connectivity = 15.3303

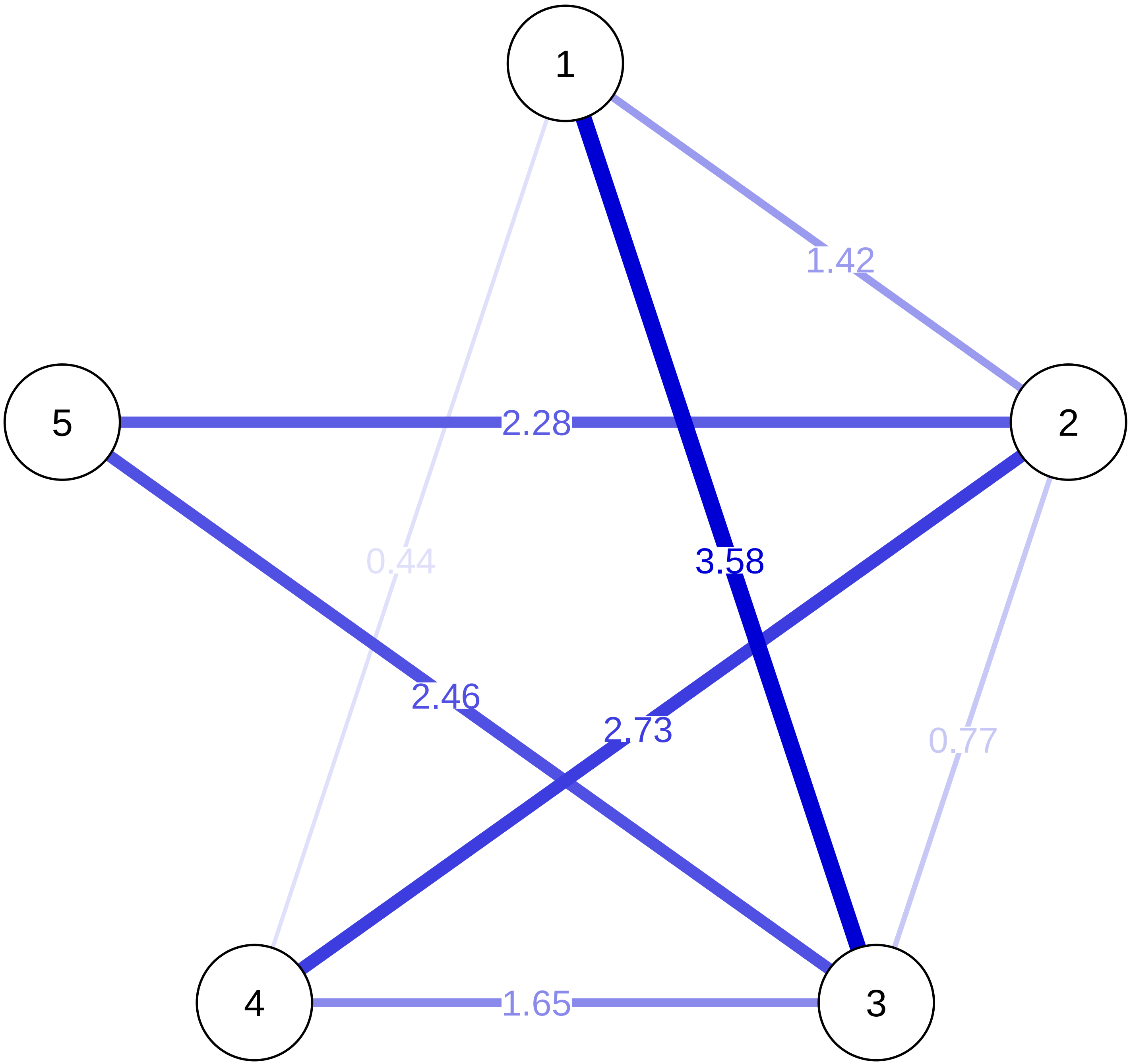

1: anxious; threshold = −4.6169
2: down; threshold = −6.345
3: not calm; threshold = −2.6648
4: depressed; threshold = −3.8738
5: not happy; threshold = −1.9399

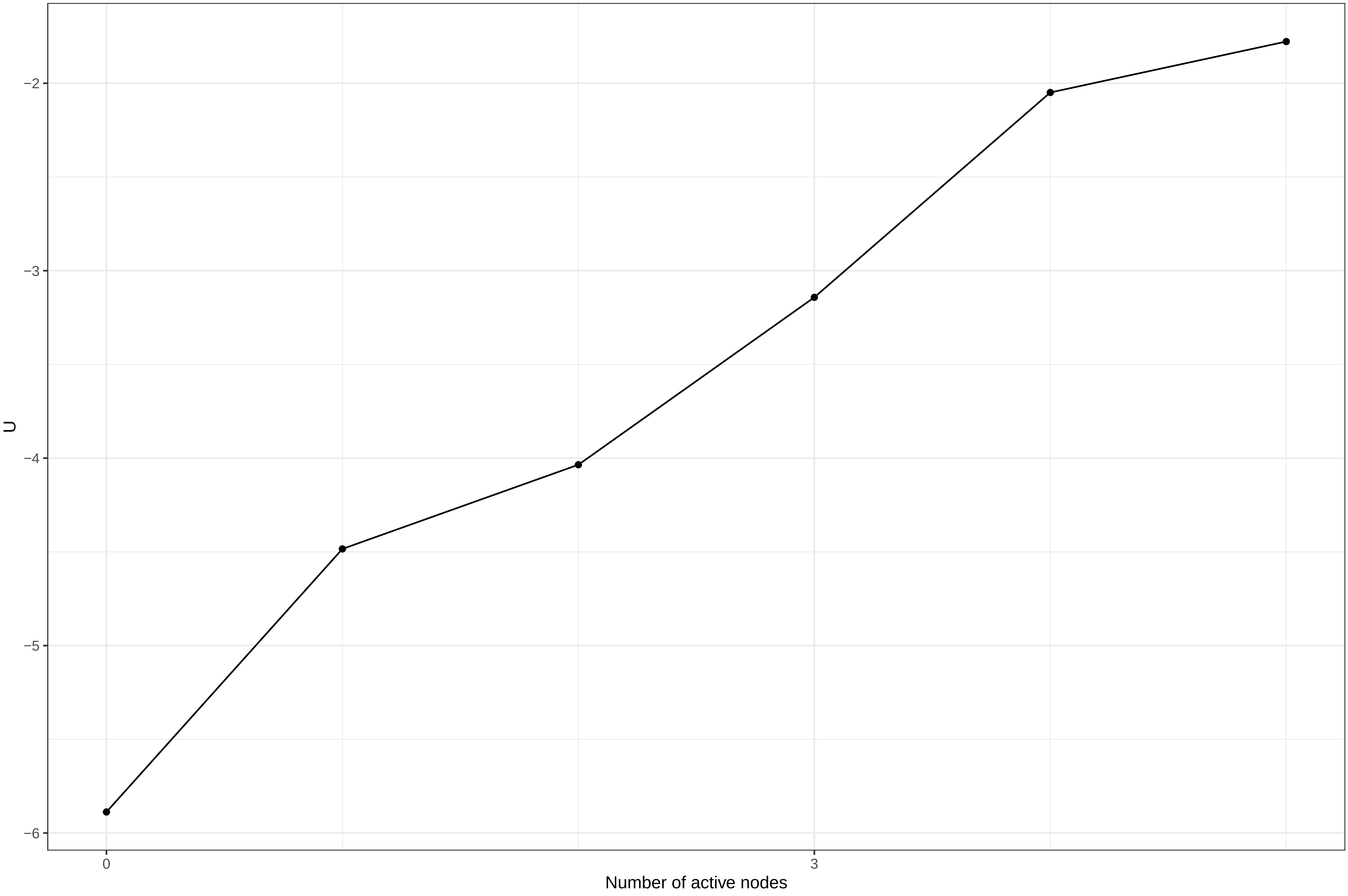

−2
−3
−4
−5
−6
U
0
3
Number of active nodes

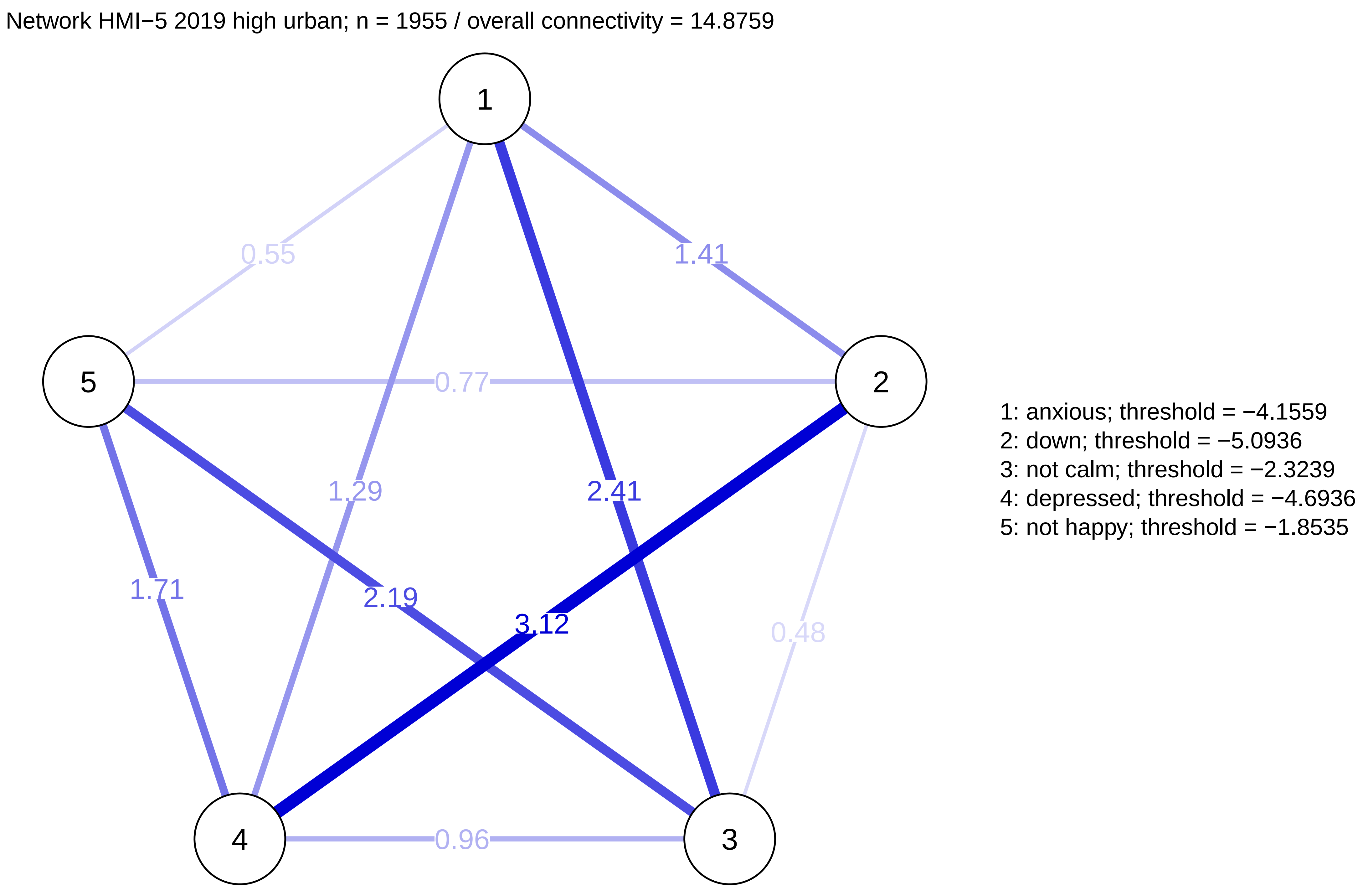

Network HMI−5 2019 high urban; n = 1955 / overall connectivity = 14.8759
1
2
3
4
5
0.55
1.41
0.77
1.29
2.41
1.71
2.19
3.12
0.48
0.96
1: anxious; threshold = −4.1559
2: down; threshold = −5.0936
3: not calm; threshold = −2.3239
4: depressed; threshold = −4.6936
5: not happy; threshold = −1.8535

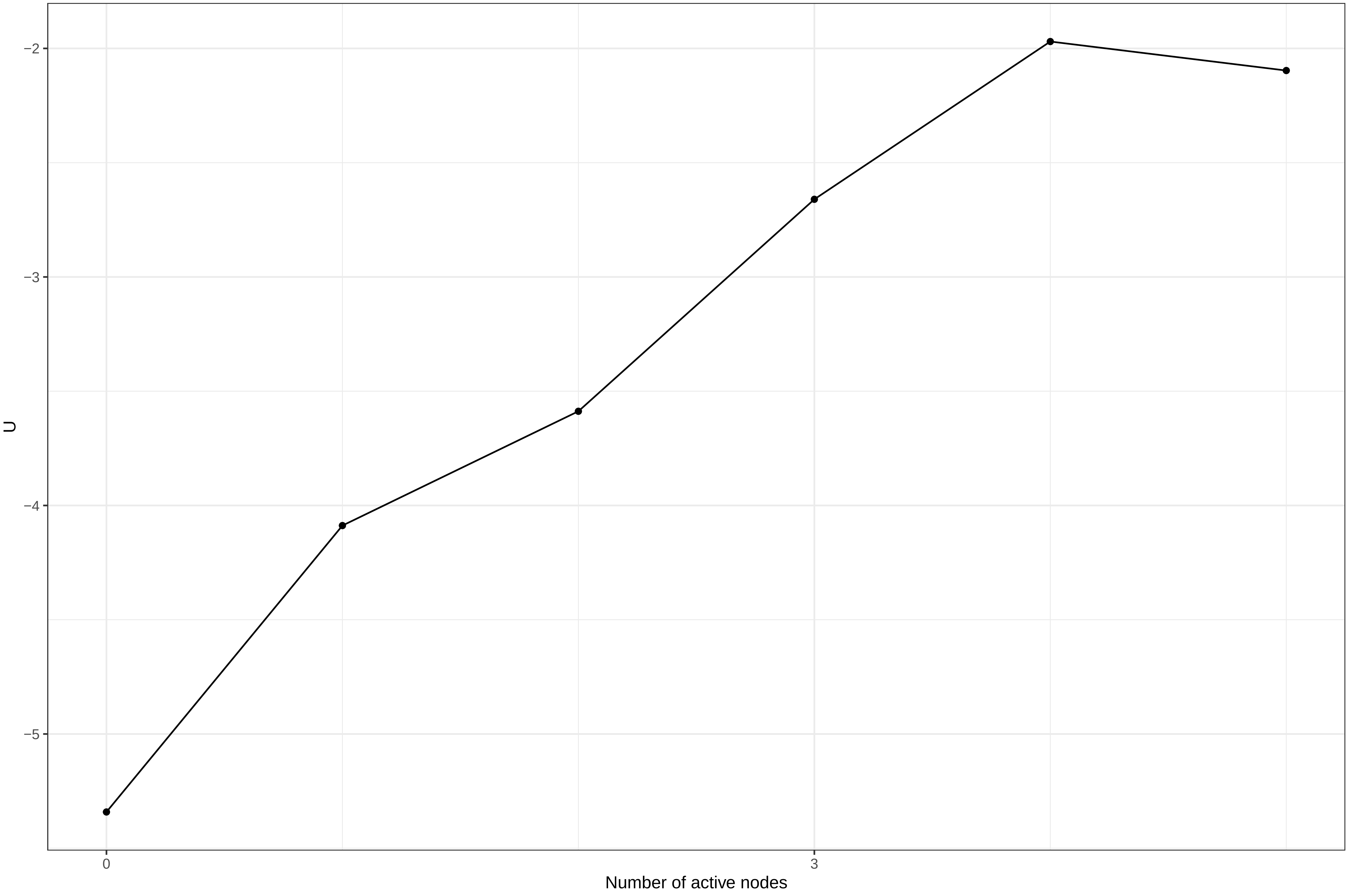

−2
−3
−4
−5
U
0
3
Number of active nodes

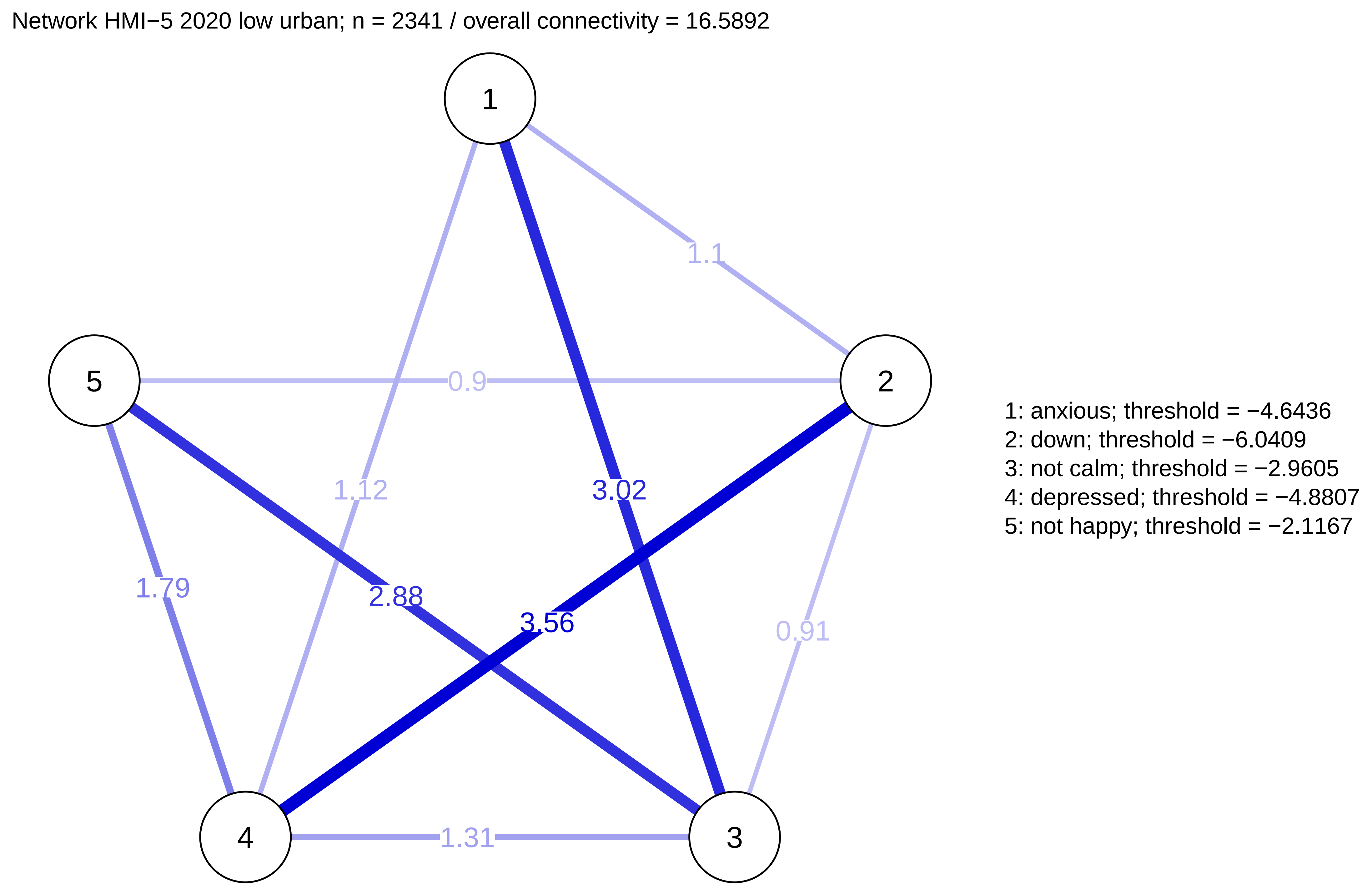
Network HMI−5 2020 low urban; n = 2341 / overall connectivity = 16.5892
1
2
3
4
5
1.1
0.9
1.12
3.02
1.79
2.88
3.56
0.91
1.31
1: anxious; threshold = −4.6436
2: down; threshold = −6.0409
3: not calm; threshold = −2.9605
4: depressed; threshold = −4.8807
5: not happy; threshold = −2.1167

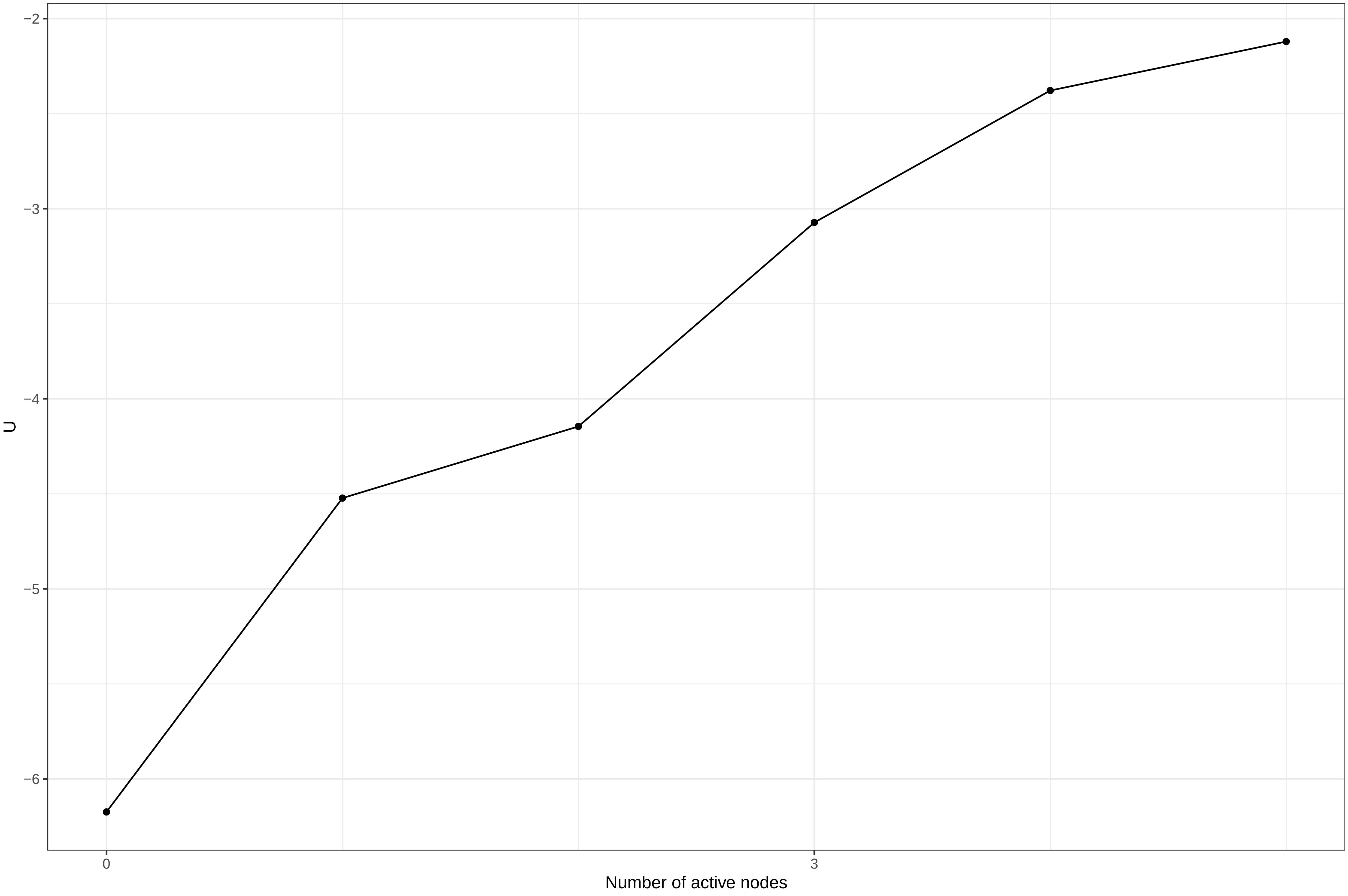
−2
−3
−4
−5
−6
U
0
3
Number of active nodes

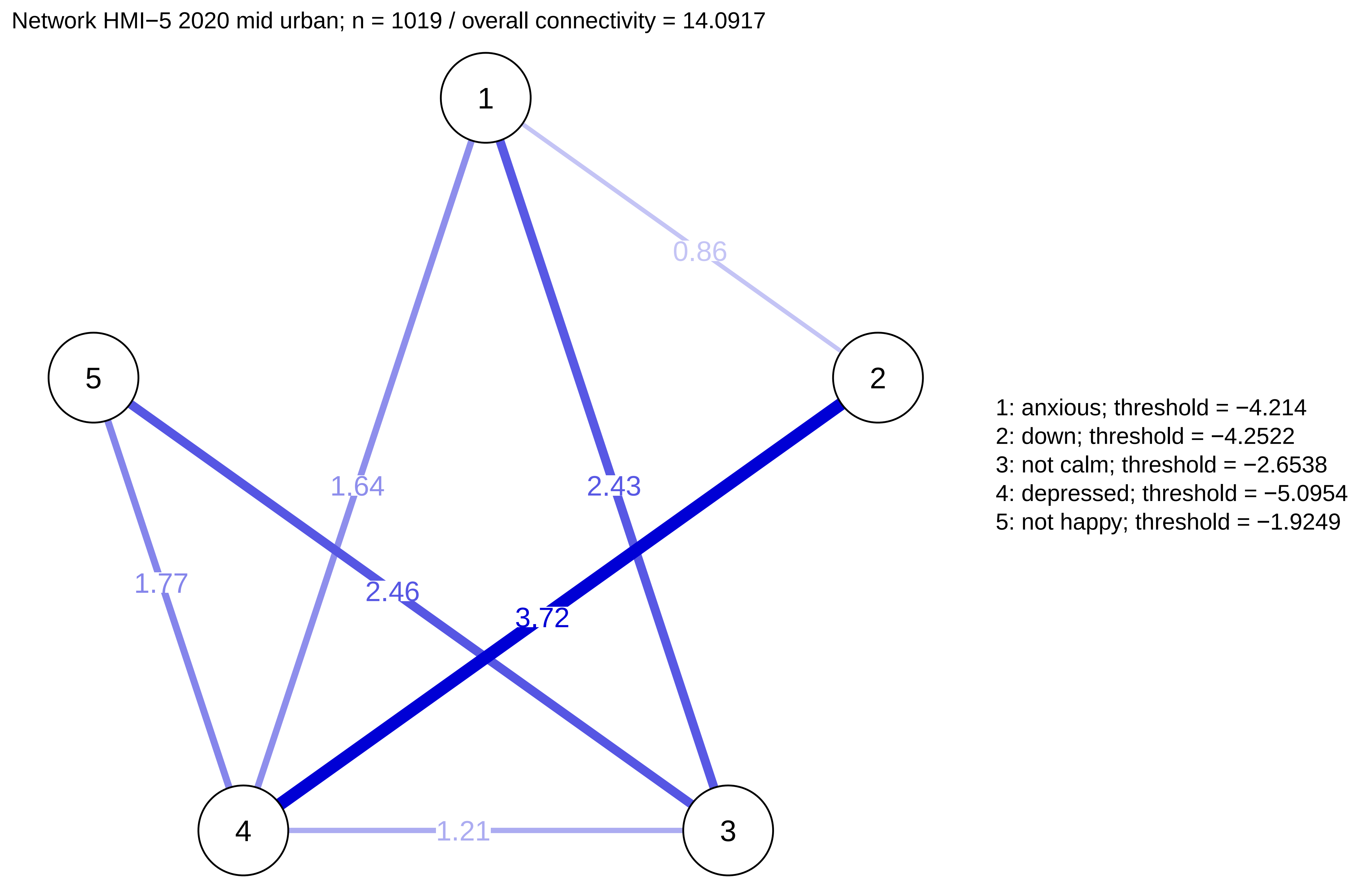
Network HMI−5 2020 mid urban; n = 1019 / overall connectivity = 14.0917
1
2
3
4
5
0.86
1.64
2.43
1.77
2.46
3.72
1.21
1: anxious; threshold = −4.214
2: down; threshold = −4.2522
3: not calm; threshold = −2.6538
4: depressed; threshold = −5.0954
5: not happy; threshold = −1.9249

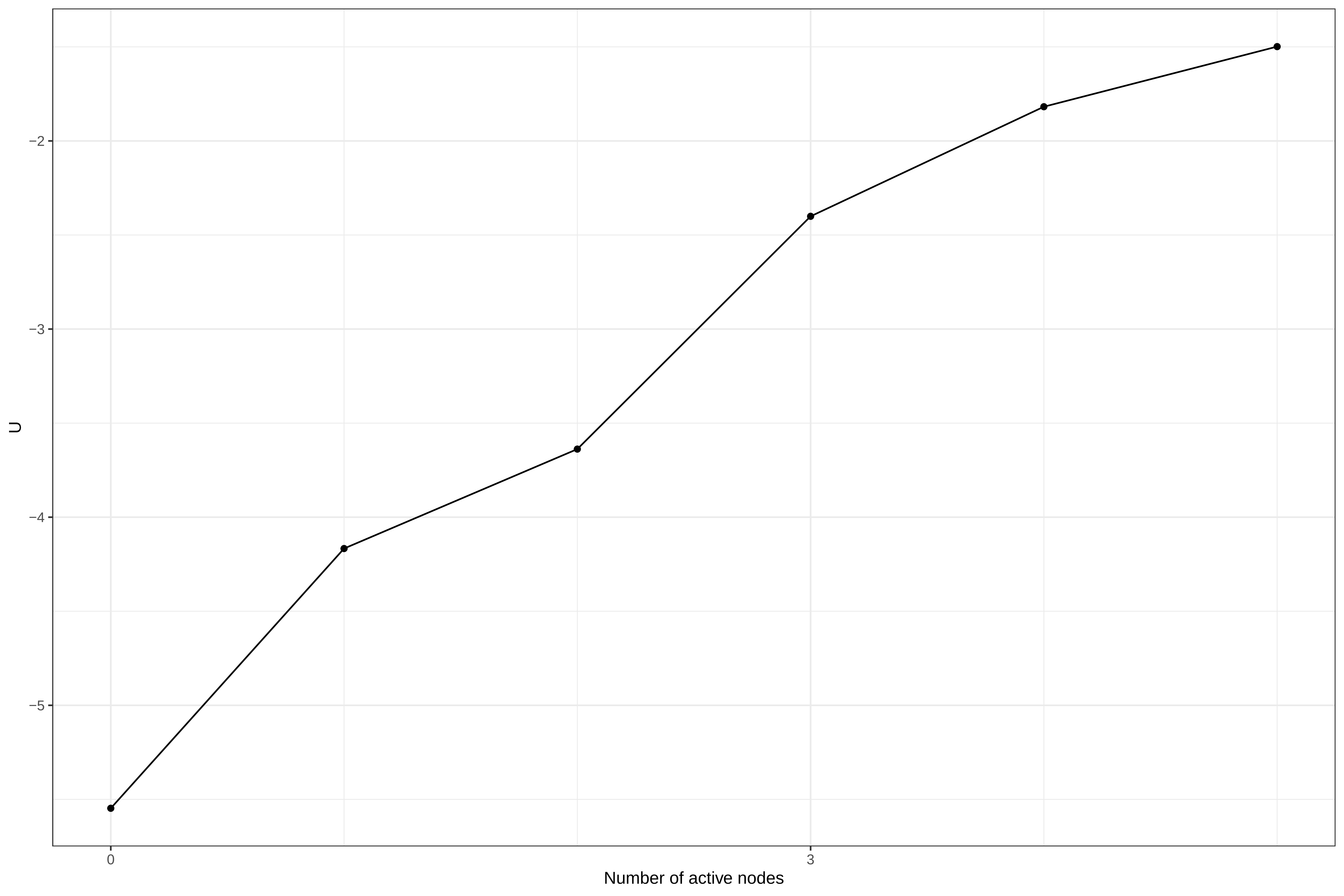
U
−2
−3
−4
−5
0
3
Number of active nodes

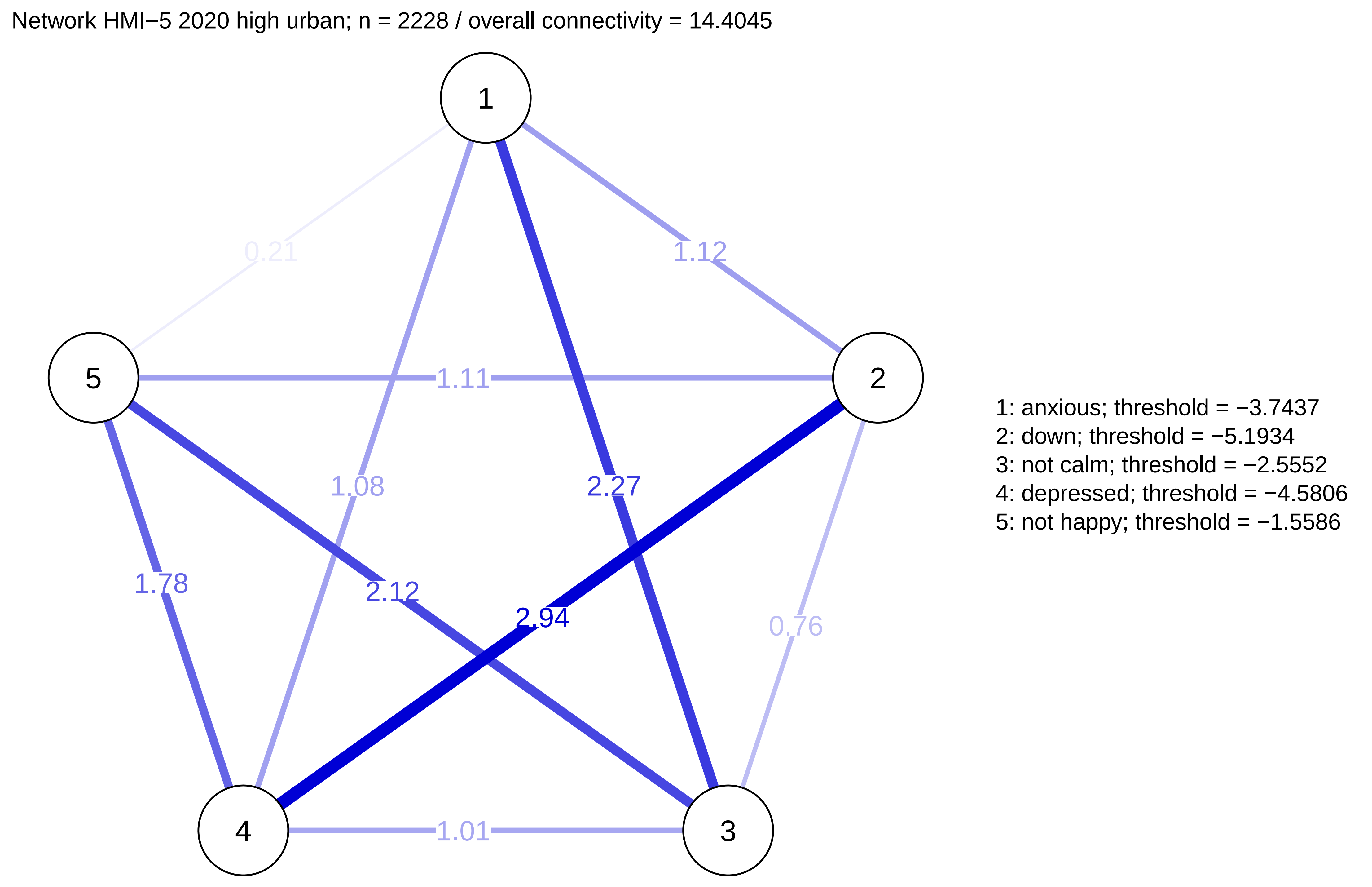
Network HMI−5 2020 high urban; n = 2228 / overall connectivity = 14.4045
1
2
3
4
5
0.21
1.12
1.11
1.08
2.27
1.78
2.12
2.94
0.76
1.01
1: anxious; threshold = −3.7437
2: down; threshold = −5.1934
3: not calm; threshold = −2.5552
4: depressed; threshold = −4.5806
5: not happy; threshold = −1.5586

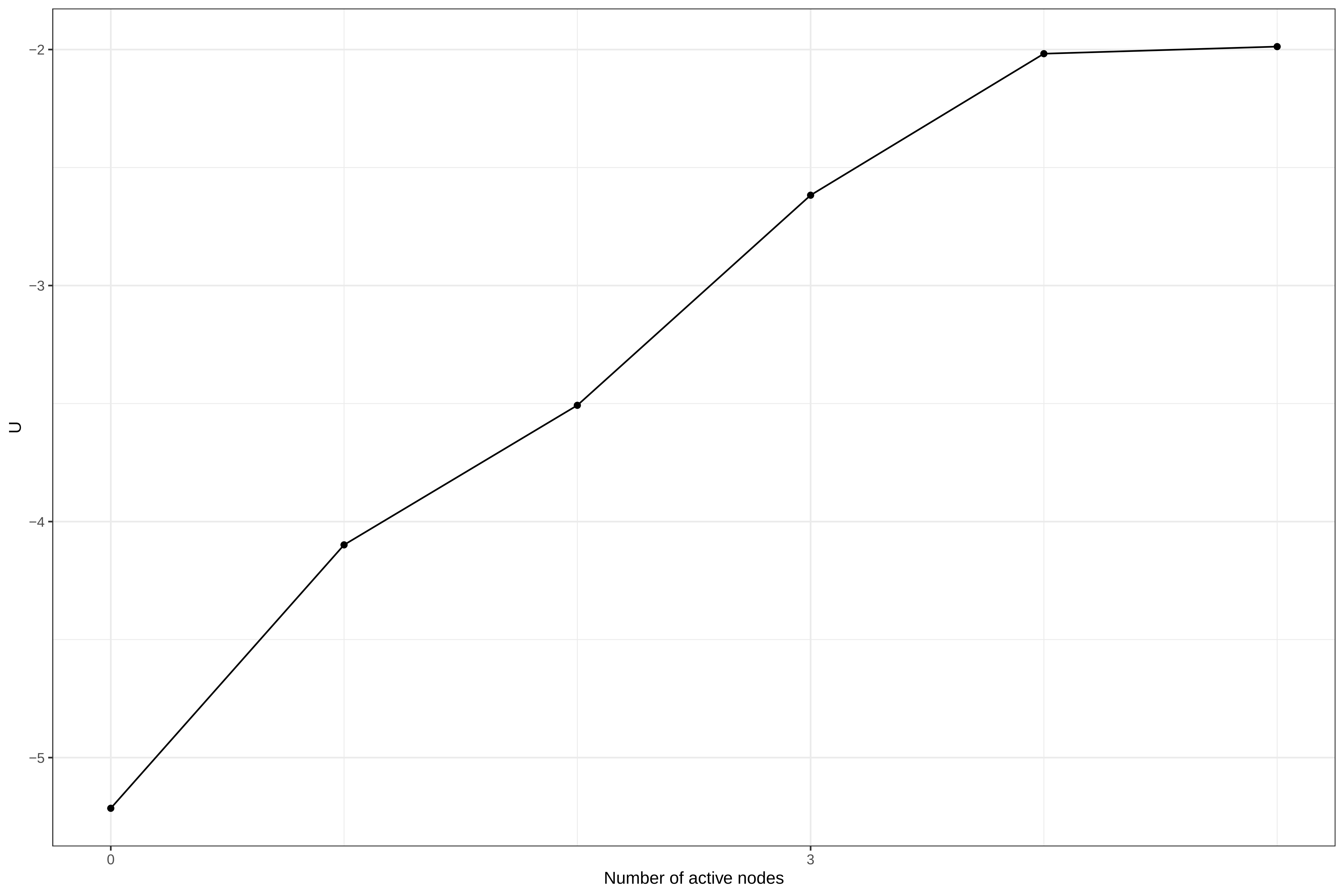

−2
−3
−4
−5
U
0
3
Number of active nodes

Network HMI−5 2021 low urban; n = 2141 / overall connectivity = 14.7861

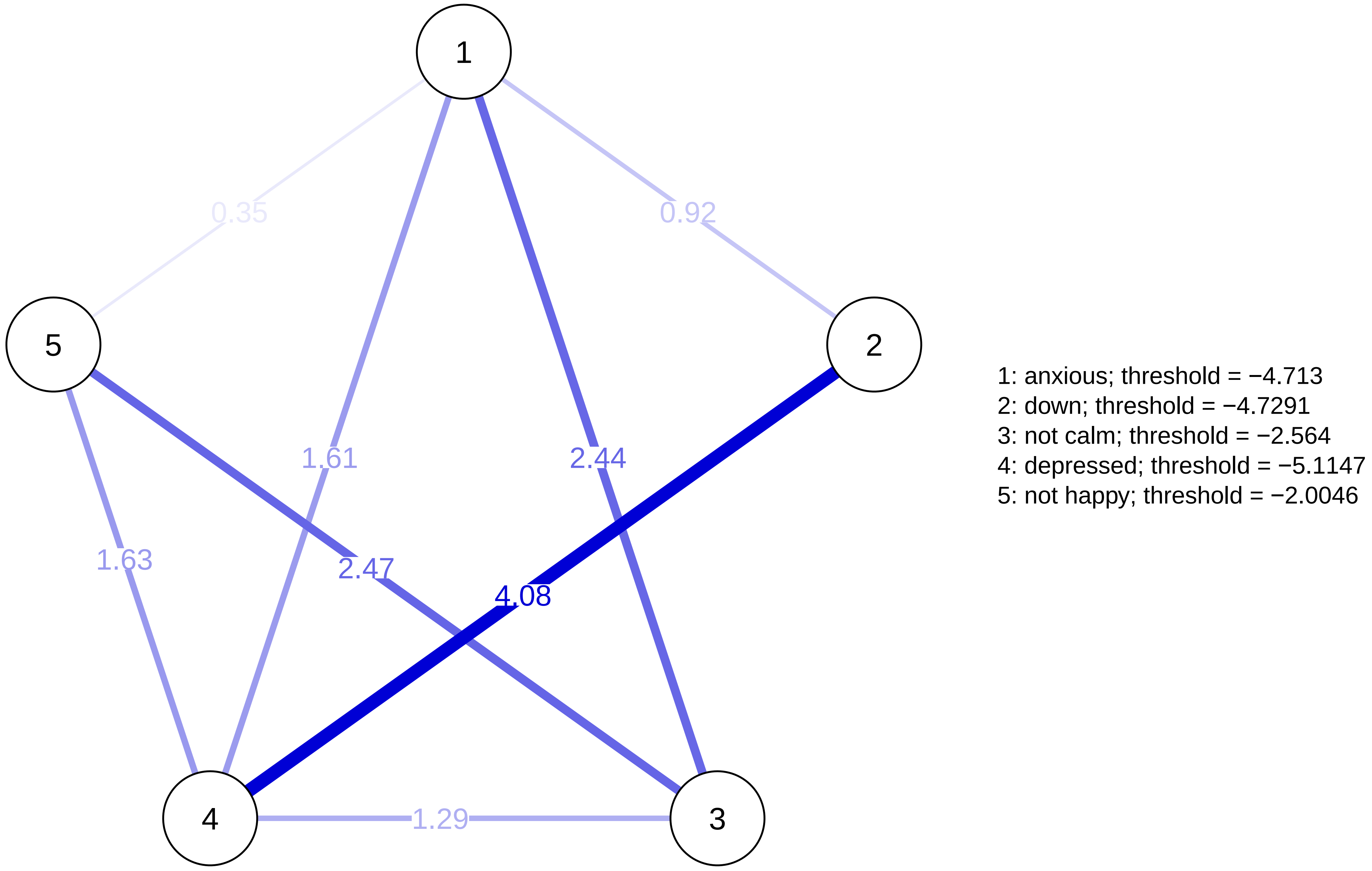

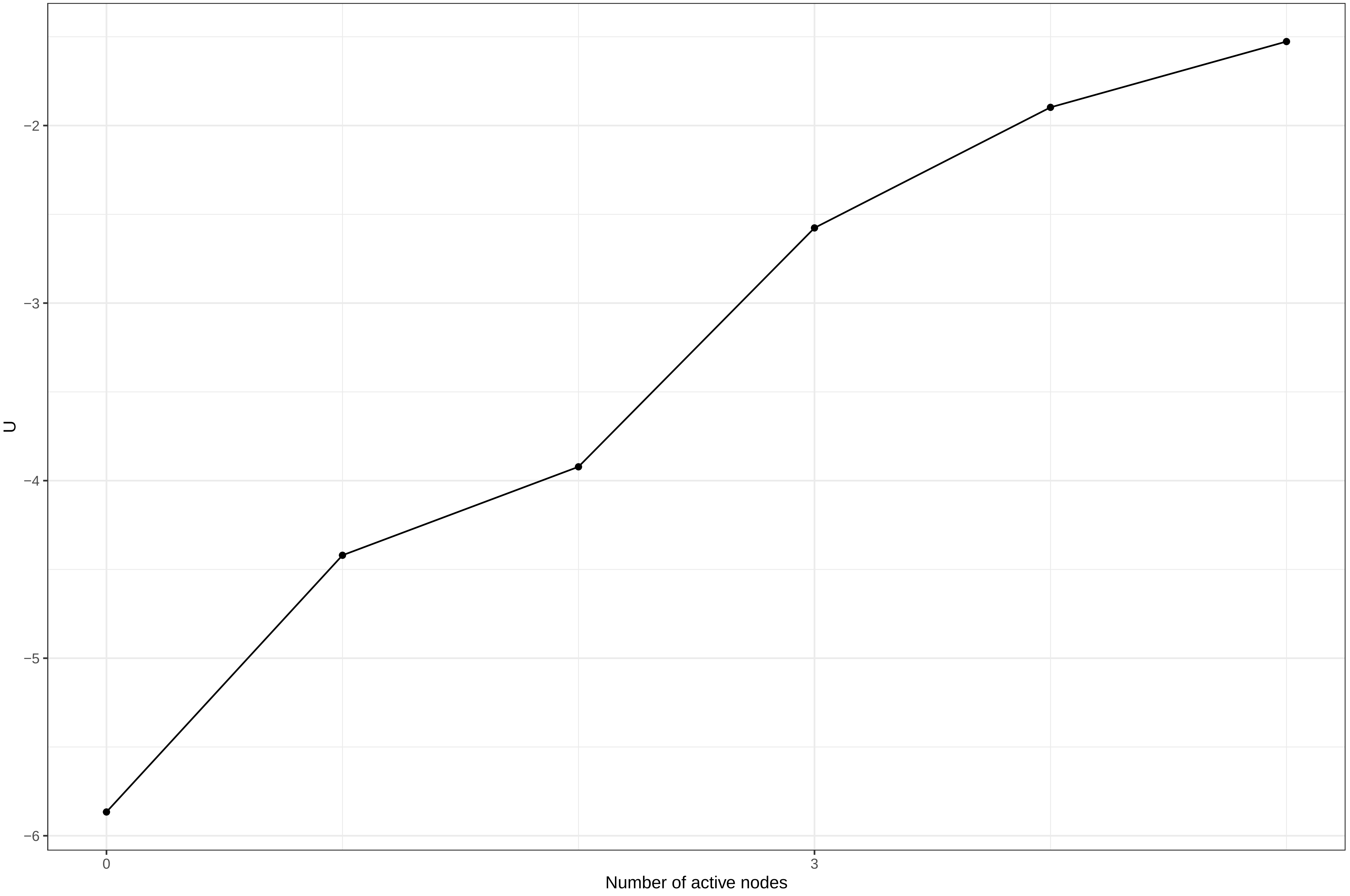

U
−2
−3
−4
−5
−6
0
3
Number of active nodes

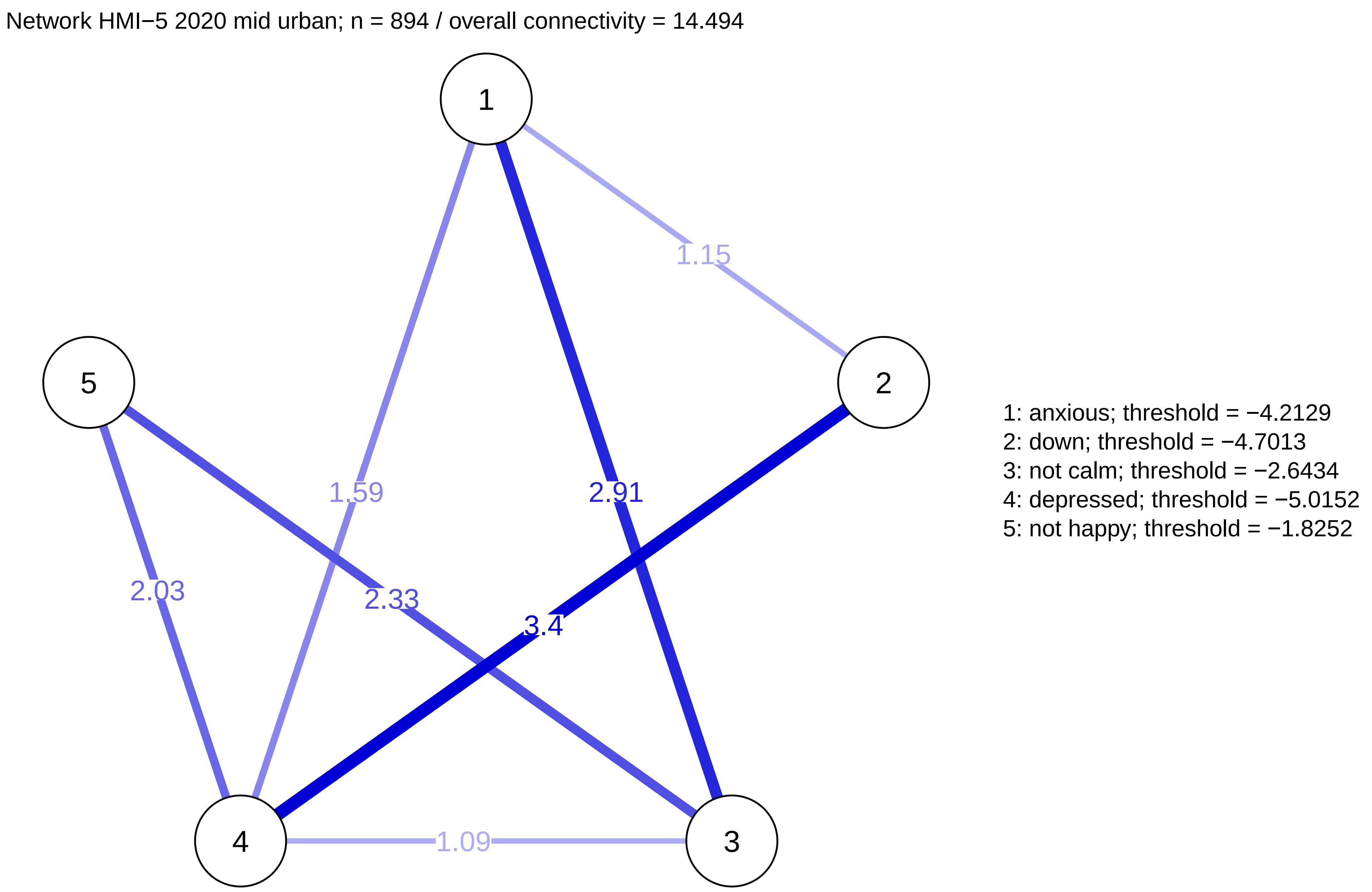
Network HMI−5 2020 mid urban; n = 894 / overall connectivity = 14.494
1
2
3
4
5
1.15
1.59
2.91
2.03
2.33
3.4
1.09
1: anxious; threshold = −4.2129
2: down; threshold = −4.7013
3: not calm; threshold = −2.6434
4: depressed; threshold = −5.0152
5: not happy; threshold = −1.8252

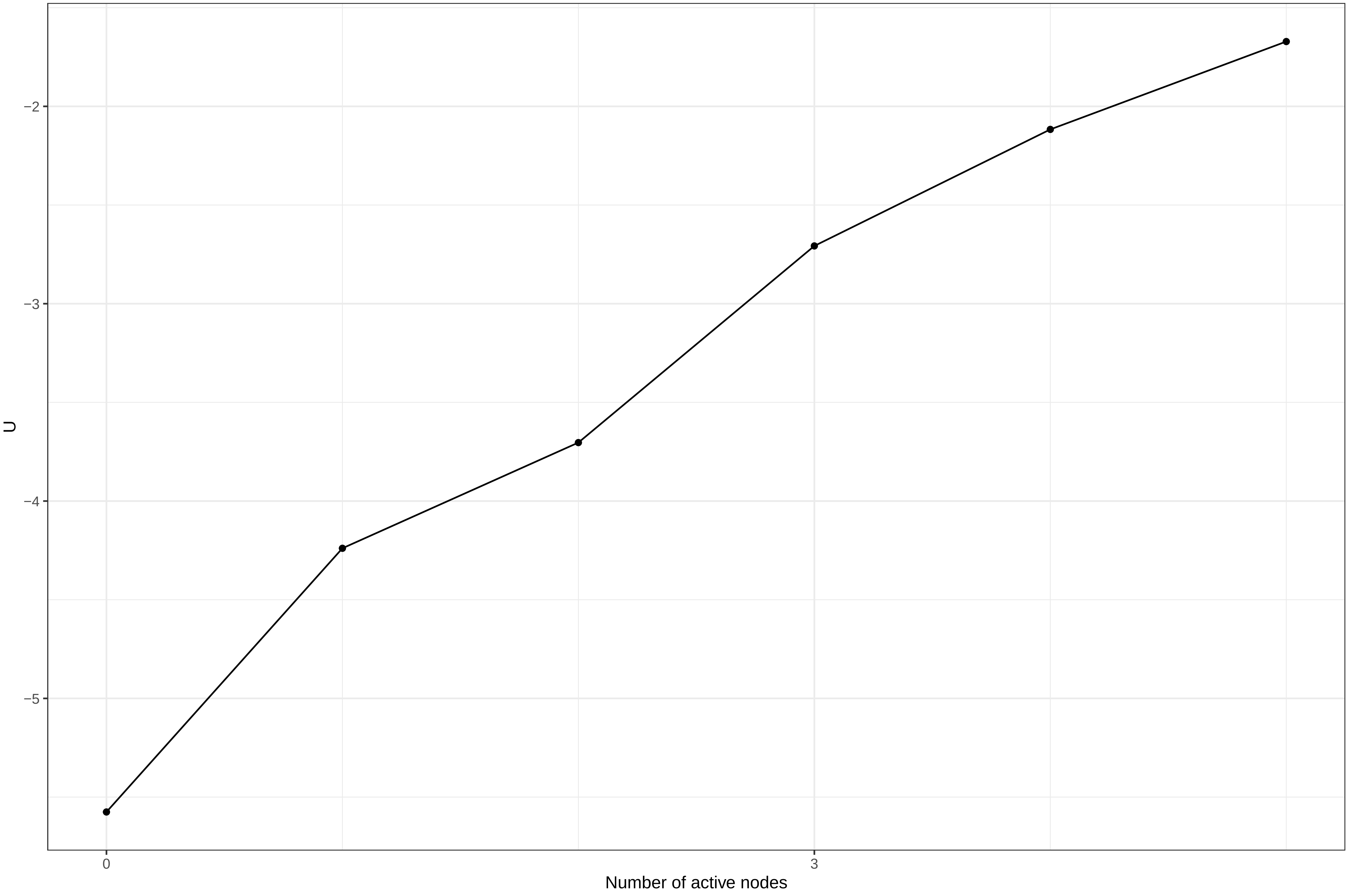

−2
−3
−4
−5
U
0
3
Number of active nodes

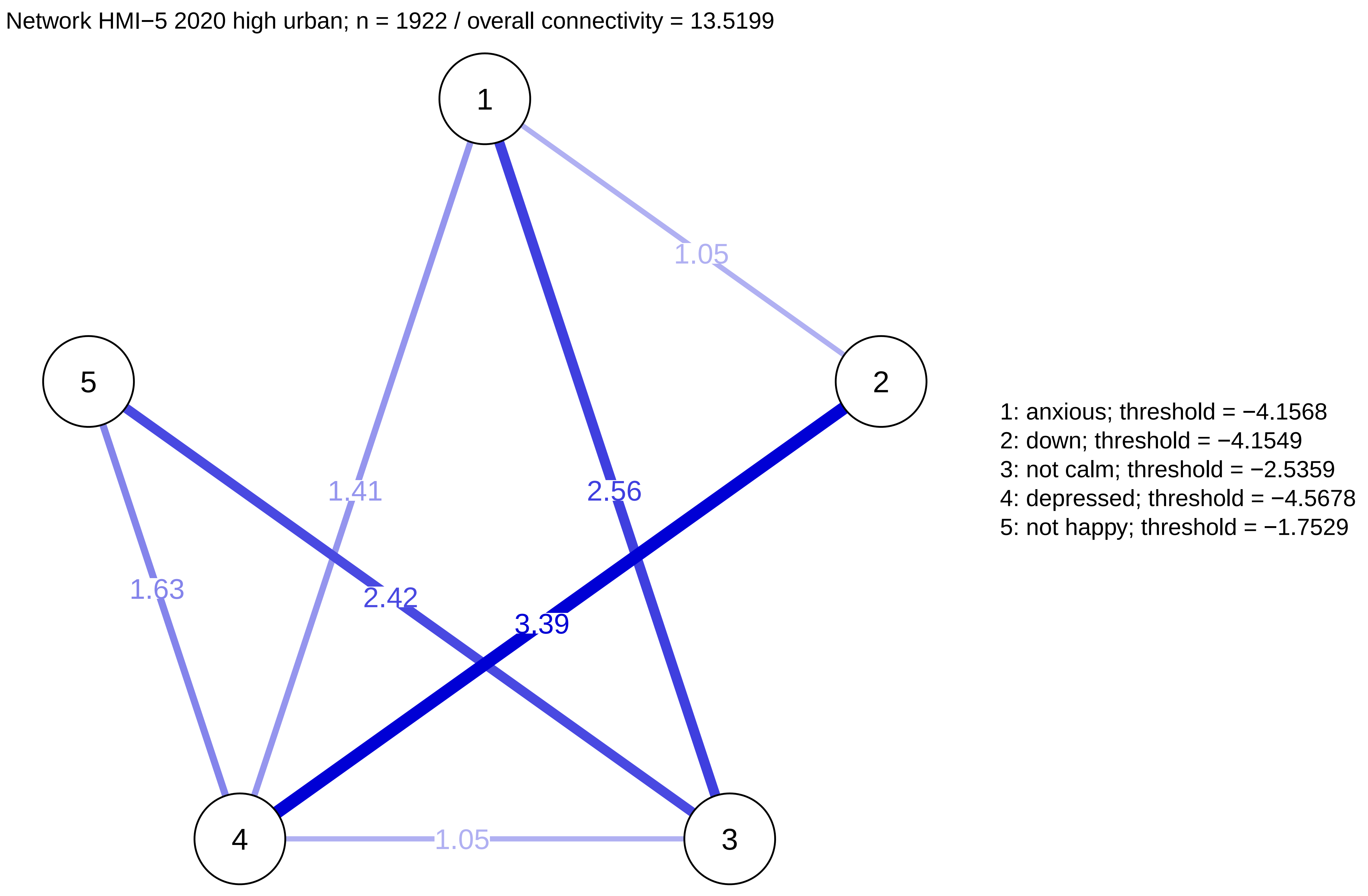
Network HMI−5 2020 high urban; n = 1922 / overall connectivity = 13.5199
1
2
3
4
5
1.05
1.41
2.56
1.63
2.42
3.39
1.05
1: anxious; threshold = −4.1568
2: down; threshold = −4.1549
3: not calm; threshold = −2.5359
4: depressed; threshold = −4.5678
5: not happy; threshold = −1.7529

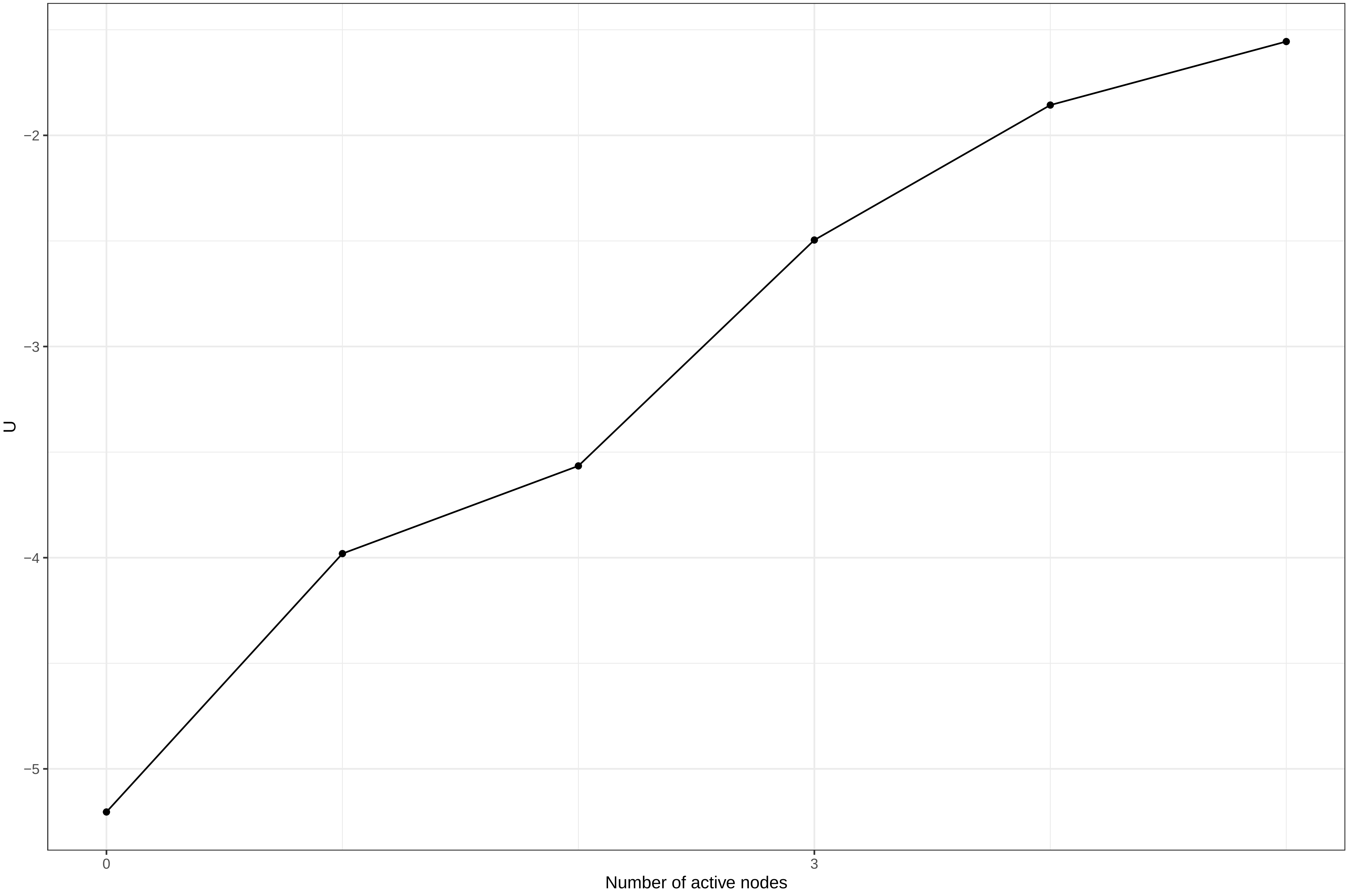

−2
−3
−4
−5
U
0
3
Number of active nodes

Network HMI−5 2022 low urban; n = 1789 / overall connectivity = 16.2355

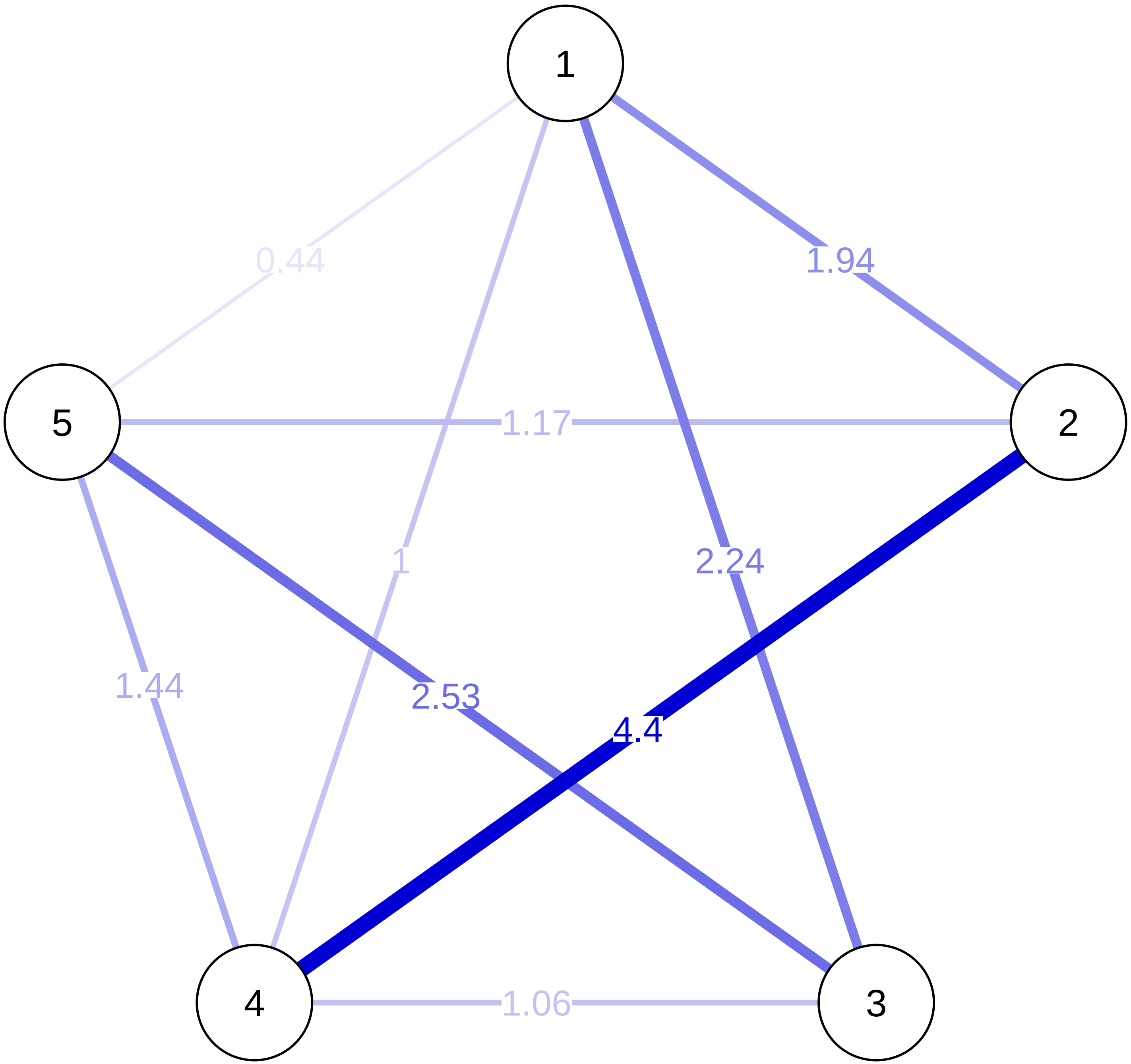

1: anxious; threshold = −4.3042
2: down; threshold = −6.2289
3: not calm; threshold = −2.5251
4: depressed; threshold = −4.8896
5: not happy; threshold = −2.0615

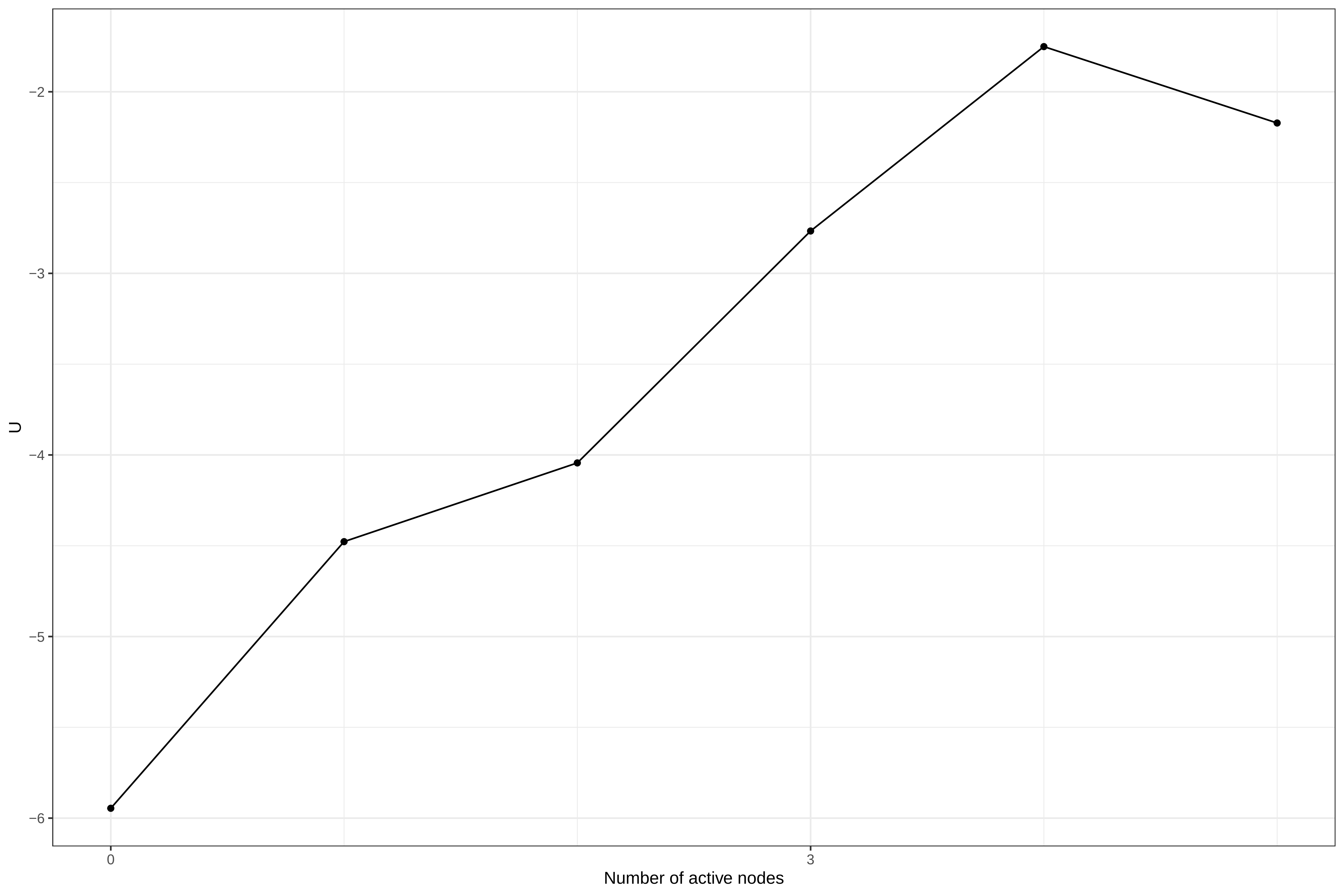

−2
−3
−4
−5
−6
U
0
3
Number of active nodes

Network HMI−5 2020 mid urban; n = 1070 / overall connectivity = 20.2839

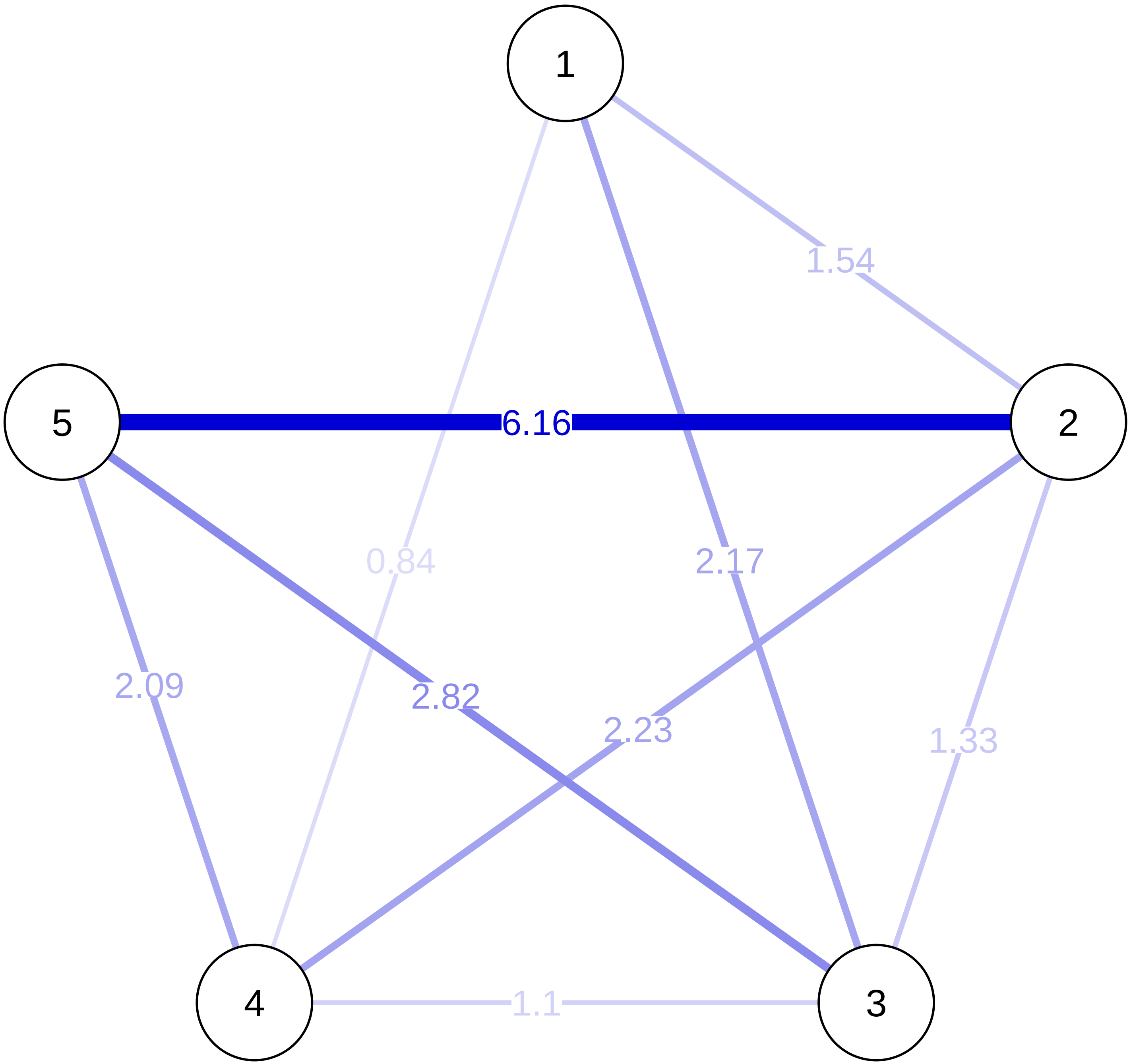

1: anxious; threshold = −3.8196
2: down; threshold = −10.7833
3: not calm; threshold = −2.7386
4: depressed; threshold = −5.0277
5: not happy; threshold = −2.1558

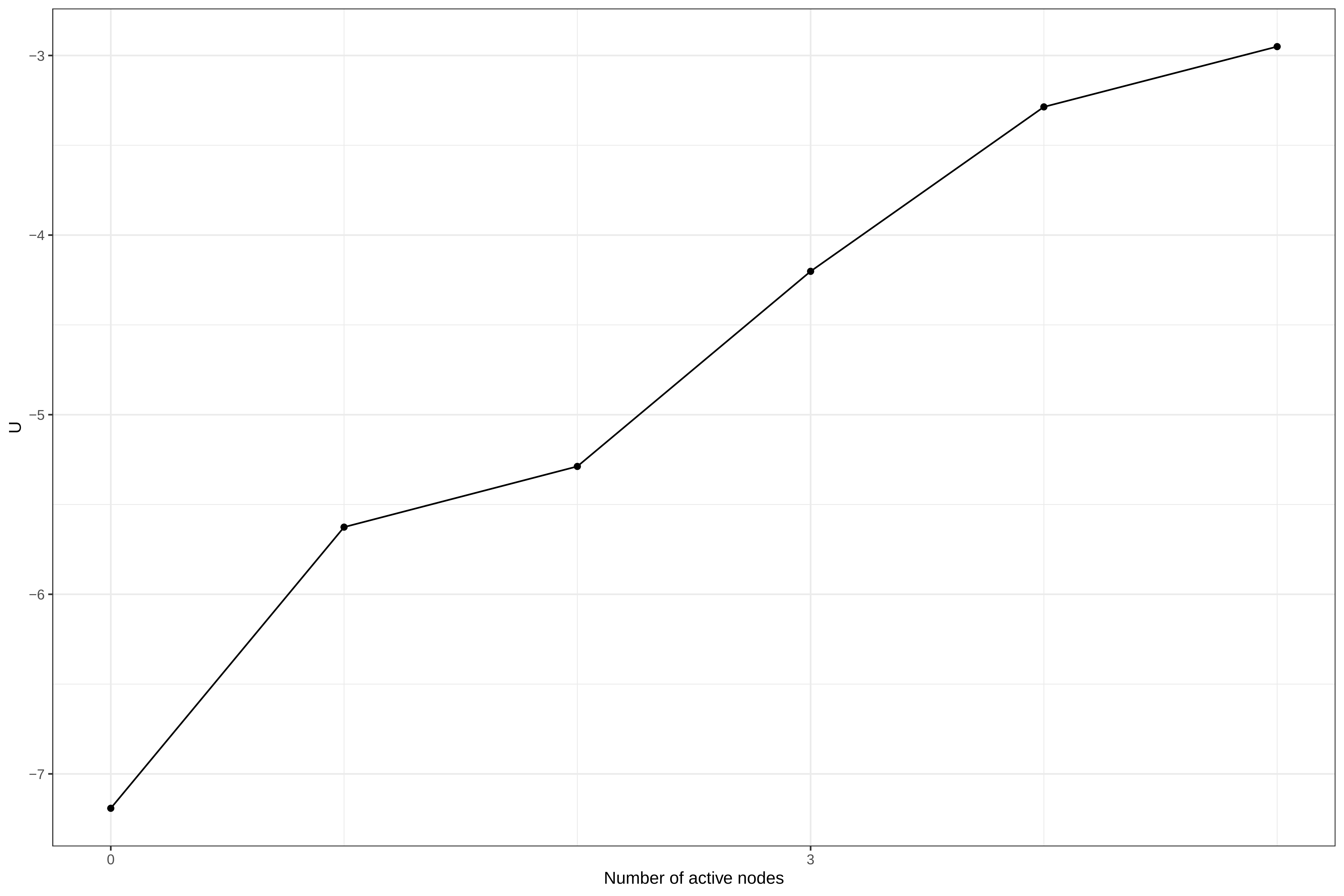

−3
−4
−5
−6
−7
U
0
3
Number of active nodes

Network HMI−5 2020 high urban; n = 2840 / overall connectivity = 14.6596

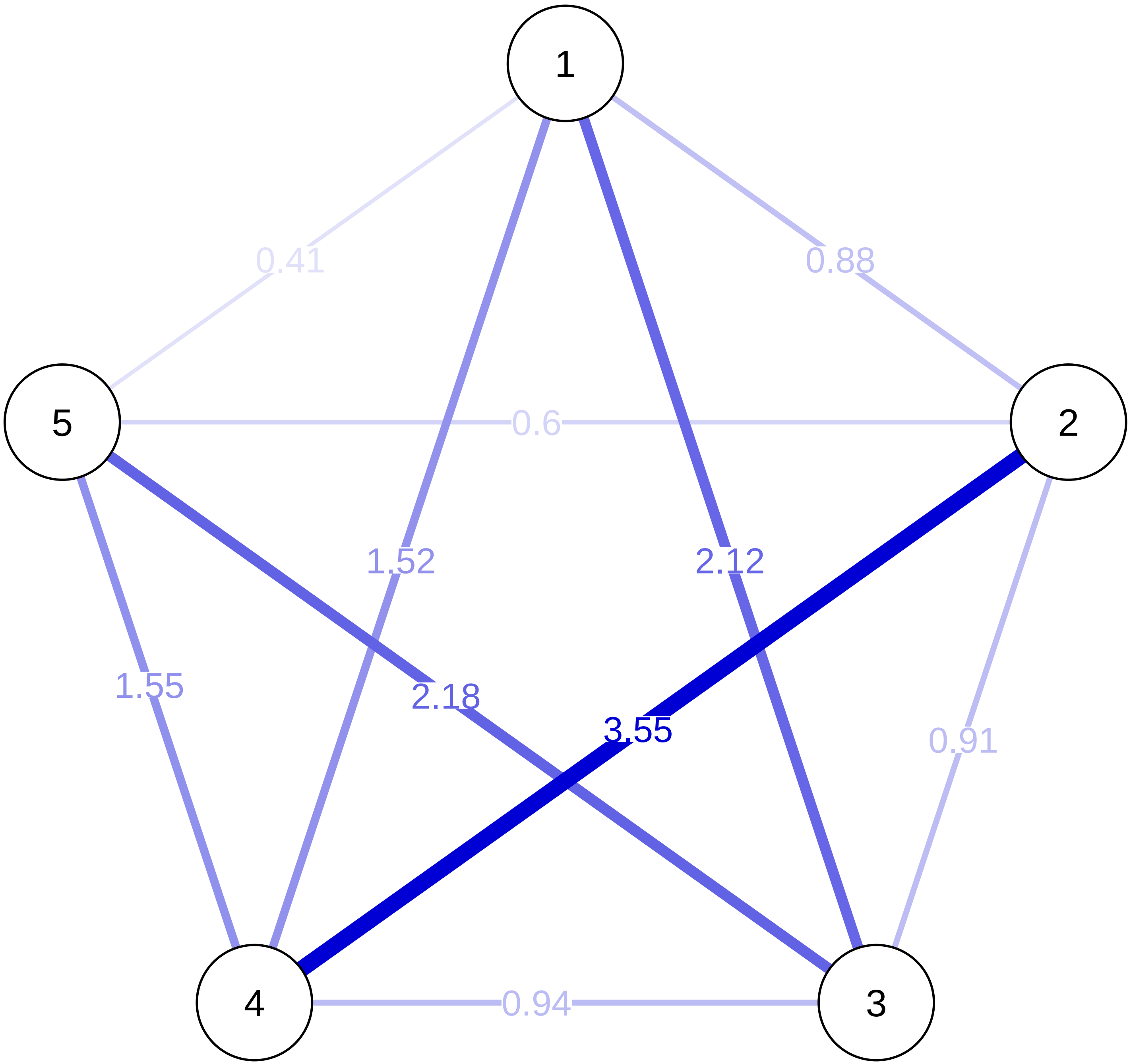

1: anxious; threshold = −3.9369
2: down; threshold = −5.2214
3: not calm; threshold = −2.3956
4: depressed; threshold = −4.768
5: not happy; threshold = −1.6101

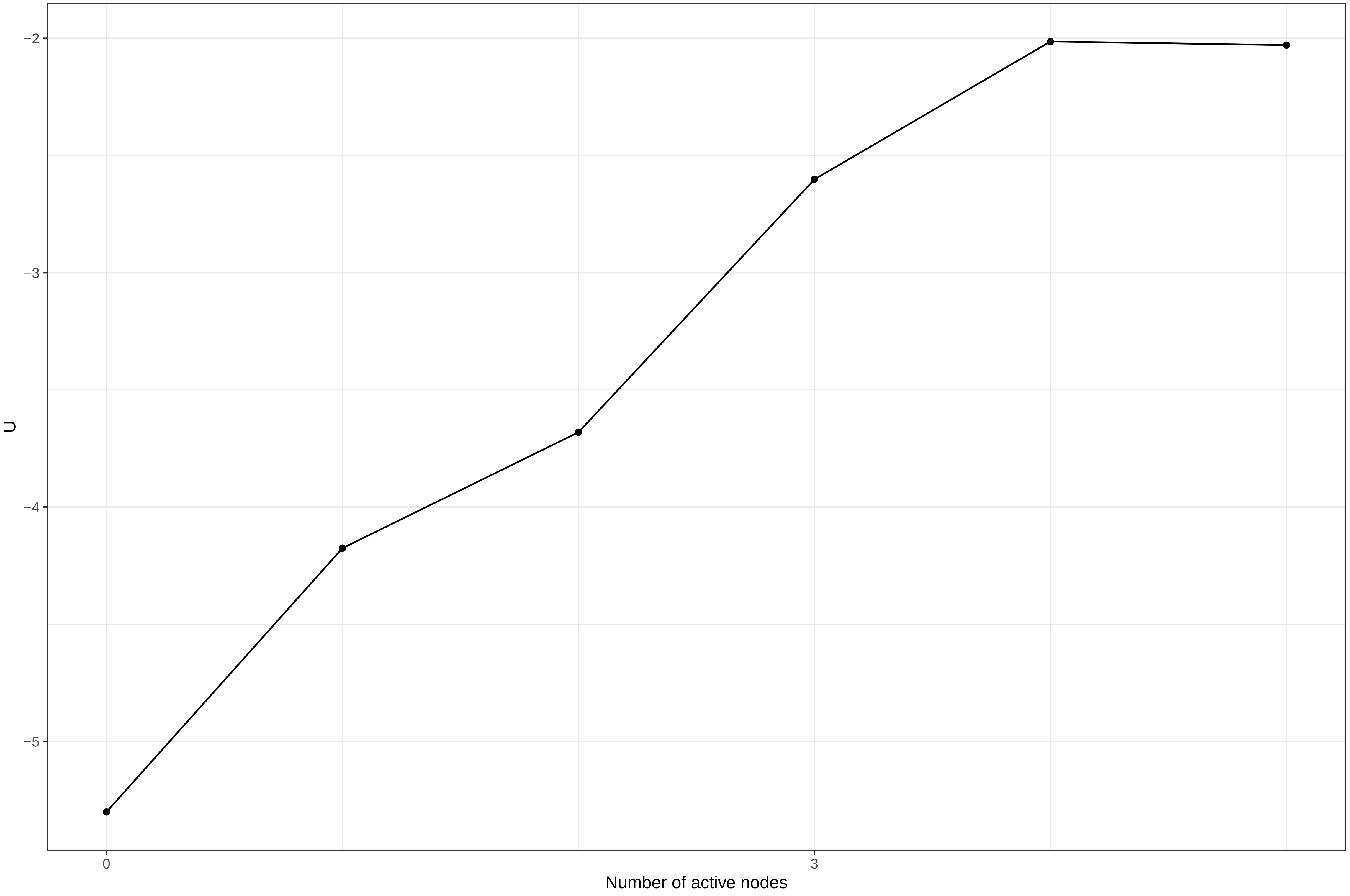

−2
−3
−4
−5
U
0
3
Number of active nodes
