# Supplementary material for: Effect of a national mental health campaign on population mental resilience in the Netherlands: a retrospective longitudinal cohort analysis using a dynamical systems perspective
Source: Lancet Reg Health Eur. 2025 Sep 4;58:101434. doi: 10.1016/j.lanepe.2025.101434 (PMC12624795; doi:10.1016/j.lanepe.2025.101434)
Supplement: Supplementary Figures and Tables [file mmc2.pdf]

Supplementary material for Lancet Regional Health – Europe

Table of content

I. LISS panel recruitment	p. 2
II. Ising model estimations	p. 2
III. Interrupted time-series analyses	p. 3
IV. Differential changes in mental resilience during COVID (2020-2022)	p. 3
Suppl. table 1: variables included from the LISS panel	p. 4
Suppl. table 2: yearly sample attrition and refreshments and response rates in the health questionnaire	p. 5
Suppl. table 3: changes in resilience indicators in males versus females in the period 2012-2022	p. 6
Suppl. table 4: changes in resilience indicators 2012-2022 according to experienced social support	p. 7
Suppl. table 5: changes in resilience indicators in the period 2012-2022 according to level of urbanicity	p. 8
Suppl. table 6: mental healthcare use 2012-2019, entire group	p. 9
Suppl. table 7: mental healthcare use 2012-2019, for males and females separately	p. 10
Suppl. table 8: mental healthcare use 2012-2019, for different levels of social support	p. 11
Suppl. table 9: mental healthcare use 2012-2019, for different levels of urbanicity	p. 12
Suppl. table 10: mental healthcare use 2012-2019, for different levels of education	p. 13
Suppl. table 11: mental healthcare use 2012-2019, different migration backgrounds	p. 15
References	p. 16
Supplementary figure 1	p. 17
Supplementary figure 2	p. 18
Supplementary figure 3	p. 19
Supplementary figure 4	p. 20
Captions for supplementary figures	p. 21

I. LISS panel recruitment

Recruitment for the LISS panel first took place from May until December 2007, through true probability sampling of 10150 households drawn from populations registries by Statistics Netherlands. [1] Self-registration was not possible, and households that would otherwise be unable to participate were provided with a computer and internet connection. Selected households received a letter and an information brochure, as well as a ten Euro banknote as prepaid incentive to increase willingness to participate, after which they were contacted through telephone (if a telephone number was available) or a house visit. Of the initial sample, 48% of households agreed to participate in the panel. Since all member of the household aged 16 years or older were asked to participate, the initial recruited sample amounted to n = 9844 participants. The first refreshment sample drawn in 2009 oversampled single-person households, older adults (aged 65+) and ethnic minorities, since these groups were underrepresented in the initial included sample. Throughout the years, various refreshment samples (either random or stratified) were drawn by Statistics Netherlands to compensate for the yearly 10% household attrition and to ensure representativeness of the panel. More information on attrition and refreshment samples in the LISS panel can be found online. The panel currently consists of about 5000 households, comprising approximately 7500 individuals.[2] Supplementary table 2 provides an overview of yearly sample attrition and refreshment in the current study sample, the number of household members selected to complete the health questionnaire, the dates between which the data was collected per year, as well as the response and completion rate. [3] Data on social variables, including level of education, urbanicity, perceived social support, and migration background, were collected annually in the months of October and November, with the exception of 2012 and 2013 when data collection took place in February and March.

II. Ising model estimations

Since methodology to estimate stability landscapes from psychological networks has only been developed for binary data, we will use binarized values of the MHI-5 score to estimate cross sectional networks with the value 1 indicating a symptom is present (i.e., the node is active) and the value 0 indicating the symptom is absent (i.e., the node is inactive). [4,5] The method to estimate such networks, called eLasso, is based on Ising models used in physics and combines logistic regression with model selection based on a Goodness-of-Fit measure to identify relevant relationships between variables that define connections in a network. Iteratively, one variable is regressed on all the others. This procedure can establish which of the variables in the data are neighbors of a given variable and which are not. To obtain sparsity, an $\ell1$-penalty is imposed on the regression coefficients that determines the level of shrinkage to favor solutions that assign fewer neighbors to any given node. A hyper-parameter called γ is used to determine the strength of the extra penalty. In the present analysis a value of $\gamma = 0{\cdot}25$ is chosen, as simulation studies have shown this value performs best for the Ising Model. The resulting set of conditional dependencies can then be visualized in a network structure. [5]

III. Interrupted time-series analyses

In the mixed models, dependent variables were proportion of participants who (I) were mentally unhealthy (HMI-5 sum score >60), (II) reported to have visited a mental healthcare professional in the last year (i.e., psychiatrist, psychologist, or psychotherapist), or (III) reported to have used medication for depression and/or anxiety in the last year. Outcomes for individual participants had a binomial distribution (either 'yes' or 'no' to the aforementioned outcomes) and thus a logit link function was applied in the models. The independent variable was the month and year in which the outcome was recorded as a way to determine whether the measurement was recorded before or after the start of the campaign, with individual participants as grouping variable. A dummy variable signifying if a measurement was recorded before (coded '0') or after (coded '1') the start of the campaign in September 2016 was introduced in a two-way interaction term in the model, to test for change in trends between the two periods as signified by the slope of this interaction term. In addition, we set out to estimate three-way interaction terms to test for differential impact according to experienced social support and gender and explore differential impact according to level of education, ethnic background, and urbanicity of the living environment. However, upon estimating these three-way interaction terms, issues with regard to model identification and/or convergence kept persisting due to collinearity between the predictors 'year' and 'intervention period', despite use of various optimizer algorithms. We then decided to examine differential impact of the campaign in the sociodemographic subgroups only through a two-way interaction term (i.e., without 'year' as predictor alongside 'intervention period'). Social support, level of education, and urbanicity of the living environment were introduced as continuous predictors, while gender and ethnicity we discreet predictors (see supplementary table 1 for operationalization). Convergence issues in the models with a two-way interaction term could be resolved by applying different optimizer algorithm (i.e., either 'bobya', 'Nelder Mead', or 'nlminb'). [6] In addition, all models were corrected for macro-economic trends using the 'business cycle tracer' computed by Statistics Netherlands to trace the performance of the Dutch economy on a monthly basis based on a variety of economic indicators including GDP, employment rate, and consumer and producer confidence. For a more comprehensive description of how the business cycle tracer is computed, we refer to the website of Statistics Netherlands. In these corrected models, the value of the business cycle tracer for the month the health questionnaire was completed was added as

fixed effect variable/covariate to the model. A p-value of 0.05 was considered to indicate statistical significance. The function glmer() from the package lme4 was used to estimate the generalized linear mixed models. [7]

IV. Differential changes in mental resilience during COVID (2020-2022)

We observed differential changes in mental resilience during COVID, in accordance to experienced social support (supplementary table 3). The low support group showed a low stability difference in 2021 (1·2, range other years 1·5 – 2·1), in keeping with the high proportion of participants who are mentally unhealthy (40.24%, highest value across all years, as the low stability difference reflects a lower tendency of the network to be in the most health state. In the high support group, stability difference decreases considerably in 2022 compared to the previous year (3·3., compared to 4·2 in 2021), while this does not seem to be explained by a higher proportion of mentally unhealthy participants in this group in 2022. We do however observe a second attractor state at the unhealthy end of the spectrum: the HMI-5 network with all symptoms active (i.e., the most unhealthy state) showed higher stability than having four symptoms active.

Supplementary table 1: variables included from the LISS panel	
**Demographic and social characteristics**	
Variable	Measure
Age	Years, based on date of birth
Gender	Self-reported*
Level of education	Highest completed, ordinal scale (1 primary school – 6 university)
Ethnicity	Self-reported, in line with the definition of Statistics Netherlands[8] (response options: Dutch background; First generation foreign, Western background; First generation foreign, non-western background; Second generation foreign, Western background; Second generation, non-westerns background)
Urbanicity of the neighborhood	Based on population density of postal code, ordinal scale composed by Statistics Netherlands (1 rural – 5 extremely urban)
Social support (continuous)	Self-reported perceived social support, ordinal (0 not at all – 10 completely satisfied)
Social support (categorized)	Participants were categorized as experiencing low levels social support (< Q1 score entire group), medium levels of social support (between Q1 – Q3 of the entire group) or high levels of social support (> Q4 of the entire group)·
**Mental health and related healthcare use**	
Variable	Measure
General mental health	5-item Mental Health Inventory (MHI-5)· Items are scored from 1-6, with higher scores indicating poorer health· The sum-score is transformed to 0-100 and a score of >= 60 signifies a person is mentally unhealthy[9]
Use of medication for depression and/or anxiety	Self-reported use of medication for depression and/or anxiety in the last year, yes/no
Visits to psychotherapists, psychologists, or psychiatrist	Self-reported visit and frequency to psychotherapists, psychologists, or psychiatrist in the last year

Abbreviations: Q1, quartile 1; Q3, quartile 3; Q4, quartile 4

*Participants in the years 2012-2021 were only able to select ‘male’ or ‘female’ as response; in 2022 the response ‘other’ was added. In 2022, 20 participants reported to identify neither as male or female; given their small number, there were excluded from analyses.

Supplementary table 2: yearly sample attrition and refreshments and response rates in the health questionnaire						
Year	Sample attrition (i.e., no. of unique drop-out for remainder of study)	Sample refreshment (i.e., no. of unique new participation)	Selected number of participants for health questionnaire	Dates of data collection	Response health questionnaire	Completed health questionnaire
2012	-	-	6769	5-11-2012 up to 31-12-2012	85·4%	84·7%
2013	575	402	6217	4-11-2013 up to 31-12-2013	86·5%	85·9%
2015	681	1365	7126	6-7-2015 up to 25-8-2015	84·3%	83·8%
2016	779	206	6336	7-11-2016 up to 27-12-2016	85·4%	84·7%
2017	663	1311	7487	6-11-2017 up to 26-12 -2017	79·6%	79·2%
2018	690	186	6466	5-11-2018 up to 31-12-2018	85·1%	84·4%
2019	513	154	5954	4-11-2019 up to 31-12-2019	86·7%	86·4%
2020	511	1003	6832	2-11-2020 up to 29-12-2020	84·0%	83·6%
2021	740	124	6274	1-11-2021 up to 28-12-2021	81·4%	81·2%
2022	655	1122	7000	7-11-2022 up to 31-12-2022	83·3%	82·9%

Supplementary table 3: changes in resilience indicators in males versus females in the period 2012-2022										
	**Year (n)**	**Percentage mentally unhealthy[a]**		**Node threshold (total, mean, SD)**		**Network connectivity**		**Stability difference**		
		Males	Females	Males	Females	Males	Females	Males	Females	
**Pre-campaign**	2012 (n = 5644)	14·59%	19·08%	-16·90, -3·38 (1·00)	-15·65, -3·13 (0·97)	13·43	12·35	2·9	2·6	
	2013 (n = 5239)	13·00%	17·8%	-17·59, -3·52 (1·33)	-16·26, -3·25 (1·12)	13·80	12·70	3·3	2·5	
	2015 (n = 4438)	11·96%	15·84%	-19·57, -3·91 (1·57)	-19·98, -4·00 (1·76)	15·03	16·40	3·4	3	
**Start campaign**	2016 (n = 5276)	13·81%	17·99%	-18·98, -3·80 (1·36)	-18·34, -3·67 (1·46)	14·81	14·68	3·3	2·9	
	2017 (n = 5819)	13·86%	17·39%	-19·06, -3·81 (1·55)	-18·32, -3·66 (1·50)	15·11	14·84	3·1	2·7	
	2018 (n = 5365)	13·71%	18·10%	-18·70, -3·74 (1·33)	-18·41, -3·68 (1·55)	14·39	14·78	3·3	2·7	
	2019 (n = 5057)	13·87%	18·74%	-18·73, -3·75 (1·43)	-18·93, -3·79 (1·57)	14·18	15·41	3·4	2·7	
**Campaign/ COVID**	2020 (n = 5615)	15·14%	18·65%	-19·53, -3·91 (1·47)	-18·64, -3·73 (1·49)	15·78	15·19	3·2	2·7	
	2021 (n = 4986)	13·66%	18·33%	-19·00, -3·80 (1·41)	-18·39, -3·68 (1·43)	14·57	14·63	3·2	2·8	
	2022 (n = 5716)	15·09%	19·90%	-19·37, -3·87 (1·60)	-18·68, -3·74 (1·61)	15·52	15·28	3·2	2·6	

Abbreviations: SD, standard deviation; MH, mental health; COVID, coronavirus disease 2019

a: Interrupted time-series analysis 2016-2019 vs. 2012-2015 * gender (female vs. male), beta: -0·15, SE: 0·09, p = 0·08

Supplementary table 4: changes in resilience indicators in the period 2012-2022 according to experienced social support

		Pre-campaign			During campaign / pre-COVID				During campaign and COVID		
	**Social support**	2012	2013	2015	2016	2017	2018	2019	2020	2021	2022
**Mentally unhealthy (%)**	Low	31·78%	33·93%	30·29%	31·84%	33·16%	35·68%	33·81%	37·83%	40·24%	34·64%
	Middle[a]	12·66%	11·25%	10·10%	11·65%	11·40%	10·03%	11·11%	14·07%	12·05%	12·35 %
	High[b]	8·55%	6·89%	7·28%	9·27%	6·70%	7·44%	6·77%	7·85%	7·35%	7·07%
**Node threshold (total, mean, SD)**	Low	-13·45, -2·69 (1·09)	-14·45, -2·89 (1·50)	-16·32, -3·26 (1·69)	-16·68, -3·34 (1·67)	-15·31, -3·06 (1·58)	-15·36, -3·07 (1·45)	-15·16, -3·03 (1·50)	-13·90, -2·78 (1·33)	-14·71, -2·94 (1·64)	-16·15, -3·23 (1·72)
	Middle	-17·19, -3·44 (0·98)	-17·72, -3·54 (1·24)	-21·40, -4·28 (1·83)	-19·11, -3·82 (1·40)	-19·75, -3·95 (1·62)	-20·23, -4·05 (1·54)	-21·21, -4·24 (1·70)	-19·95, -3·99 (1·72)	-19·56, -3·91 (1·59)	-20·60, -4·12 (1·75)
	High	-18·04, -3·61 (0·81)	-18·40, -3·68 (1·00)	-21·67, -4·33 (1·44)	-20·36, -4·07 (1·30)	-19·19, -3·84 (1·06)	-20·54, -4·11 (1·09)	-21·65, -4·33 (1·60)	-22·90, -4·58 (1·85)	-19·67, -3·93 (1·06)	-21·64, -4·33 (1·94)
**Network connectivity**	Low	11·18	12·44	13·74	14·07	12·76	13·12	13·02	11·87	12·95	14·08
	Middle	13·33	13·49	17·01	14·56	15·68	15·683	16·70	15·48	14·64	16·08
	High	13·56	13·70	16·10	15·62	13·65	15·33	17·04	17·91	13·72	17·12
**Stability difference**	Low	1·7	1·5	2·1	1·8	1·6	1·6	1·6	1·5	1·2	1·5
	Middle	3·2	3·3	3·5	3·5	3·2	3·6	3·5	3·1	3·5	3·2
	High	3·7	3·9	3·8	3·9	4·0	3·5	3·7	3·8	4·2	3·3

Abbreviations: SD, standard deviation; COVID, coronavirus disease 2019

a: Interrupted time-series analysis 2016-2019 vs. 2012-2015 * social support (middle vs. low), beta: -0·08, SE: 0·10, p = 0·41

b: Interrupted time-series analysis 2016-2019 vs. 2012-2015 * social support (high vs. low), beta: 0·01, SE: 0·16, p = 0·95

Supplementary table 5: changes in resilience indicators in the period 2012-2022 according to level of urbanicity										
		**Pre-campaign**			**During campaign / pre-COVID**				**During campaign and COVID**		
	**Urbanicity**[a]	2012	2013	2015	2016	2017	2018	2019	2020	2021	2022
Mental unhealthy (%)	Low	15·33%	13·75%	13·00%	13·92%	14·02%	14·08%	14·57%	14·02%	13·94%	15·32%
	Middle	17·35%	15·38%	13·79%	15·45%	15·38%	15·28%	15·40%	16·80%	15·57%	15·15%
	High	18·39%	17·66%	15·26%	17·92%	17·30%	18·68%	19·19%	20·36%	18·85%	20·18%
Node threshold (total, mean)	Low	-16·09, -3·22 (081)	-17·74, -3·55 (129)	-21·86, -4·37 (214)	-18·72, -3·74 (143)	-19·03, -3·81 (159)	-19·11, -3·82 (145)	-19·75, -3·95 (153)	-20·64, -4·13 (157)	-19·12, -3·83 (143)	-20·01, -4·00 (172)
	Middle	-15·63, -3·13 (097)	-16·43, -3·29 (112)	-18·81, -3·76 (149)	-19·23, -3·85 (147)	-18·86, -3·77 (150)	-19·92, -3·98 (155)	-19·44, -3·89 (172)	-18·14, -3·63 (130)	-18·40, -3·68 (138)	-24·52 -4·91 (346)
	High	-15·84, -3·17 (104)	-16·77, -3·35 (121)	-19·50, -3·90 (166)	-18·05, -3·61 (139)	-17·93, -3·59 (151)	-18·14, -3·63 (139)	-18·12, -3·62 (145)	-17·63, -3·53 (148)	-17·17, -3·43 (122)	-17·93, -3·59 (154)
Network connectivity	Low	12·61	14·00	17·71	14·31	15·06	15·02	15·75	16·59	14·79	16·24
	Middle	12·11	12·82	14·67	15·61	14·89	15·93	15·33	14·09	14·49	14·49
	High	12·07	13·41	15·87	14·42	14·52	14·90	14·88	14·40	13·52	14·66
Stability difference	Low	2·9	3·2	3·4	3·3	3·1	3·1	3·2	3·1	3·1	2·8
	Mid	2·8	2·7	3·4	3·1	3·0	3·1	2·7	3·1	2·9	3
	High	2·7	2·7	2·9	2·9	2·7	2·6	2·6	2·6	2·7	2·7

Abbreviations: MH, mental health; COVID, coronavirus disease 2019

a: Interrupted time-series analysis 2016-2019 vs. 2012-2015 * urbanicity (continuous measure), beta: -0·01, SE: 0·03, p = 0·67

Supplementary table 6: mental healthcare use 2012-2019, entire group						
		**MH care visit last 12 months[a]**	**MH care visit last 12 months, if yes how many? (frequency)**			**Medication use for depression and/or anxiety last 12 months[b]**
	**Year (n)**	yes/no (% yes)	1-5	5-10	>10	yes/no (% yes)
**Pre-campaign**	2012 (n = 5644)	6·57%	196	97	76	4·32%
	2013 (n = 5239)	6·89%	208	78	73	4·46%
	2015 (n = 4438)	6·33%	157	74	49	4·60%
**During campaign**	2016 (n = 5276)	7·63%	192	119	89	5·02%
	2017 (n = 5819)	8·15%	242	128	102	5·02%
	2018 (n = 5365)	8·13%	221	107	105	4·77%
	2019 (n = 5057)	8·17%	205	107	100	4·82%

Abbreviations: MH, mental health
a: Interrupted time-series analysis 2016-2019 vs. 2012-2015, beta: -0·01, SE: 0·05, p = 0·82
b: Interrupted time-series analysis 2016-2019 vs. 2012-2015, beta: -0·31, SE: 0·12, p = 0·01

Supplementary table 7: mental healthcare use 2012-2019, for males and females separately							
			**MH care visit last 12 months yes/no**[a]	**MH care visit last 12 months, if yes how many? (frequency)**			**Medication for depression and/or anxiety last 12 months yes/no**[b]
	**Year**	**Gender (n)**	(% yes)	1-5	5-10	>10	(% yes)
**Pre-campaign**	2012	Males (n = 2616)	4·18%	61	27	21	3·31%
		Females (n = 3028)	8·64%	135	70	55	5·19%
	2013	Males (n = 2431)	5·24%	77	26	24	3·30%
		Females (n = 2808)	8·32%	131	52	49	5·48%
	2015	Males (n = 2074)	4·64%	52	30	14	3·33%
		Females (n = 2364)	7·82%	105	55	24	5·71%
**During campaign**	2016	Males (n = 2479)	5·43%	59	42	33	3·48%
		Females (n = 2797)	9·59%	133	77	56	6·38%
	2017	Males (n = 2672)	5·45%	78	38	29	3·39%
		Females (n = 3147)	10·44%	164	90	73	6·04%
	2018	Males (n = 2471)	5·39%	78	40	16	3·36%
		Females (n = 2894)	10·43%	143	75	81	5·97%
	2019	Males (n = 2359)	5·52%	70	34	26	3·23%
		Females (n = 2698)	10·49%	135	75	72	6·21%

Abbreviations: MH, mental health

a: Interrupted time-series analysis 2016-2019 vs. 2012-2015 * gender (female vs. male), beta: 0·07, SE: 0·13, p = 0·59

b: Interrupted time-series analysis 2016-2019 vs. 2012-2015 * gender (female vs. male), beta: 0·37, SE: 0·30, p = 0·22

Supplementary table 8: mental healthcare use 2012-2019, for different levels of social support							
			**MH care visit last 12 months yes/no**[a,b]	**MH care visit last 12 months, if yes how many? (frequency)**			**Medication for depression and/or anxiety last 12 months yes/no**[c,d]
	**Year**	**Level of social support**	(% yes)	1-5	5-10	>10	(% yes)
**Pre-campaign**	2012	Low (n = 1076)	9·94%	58	25	23	6·84%
		Middle (n = 3085)	5·40%	86	50	30	3·68%
		High (n = 867)	4·05%	21	8	6	3·71%
	2013	Low (n = 946)	12·34%	55	27	34	8·07%
		Middle (n 2972)	5·57%	109	28	28	3·61%
		High (n = 856)	3·87%	19	9	5	3·63%
	2015	Low (n = 817)	10·57%	42	26	18	8·57%
		Middle (n = 2608)	5·65%	90	33	24	3·64%
		High (n 618)	2·11%	7	4	2	3·56%
**During campaign**	2016	Low (n = 1047)	13·26%	64	45	29	7·85%
		Middle (n = 3052)	6·23%	94	56	39	4·20%
		High (n = 766)	5·37%	25	8	8	4·31%
	2017	Low (n = 1165)	13·68%	76	40	43	7·89%
		Middle (n = 3386)	6·84%	122	66	43	3·98%
		High (n = 821)	5·87%	25	15	8	4·41%
	2018	Low (n = 1097)	14·44%	72	34	52	8·49%
		Middle (n = 3115)	5·73%	103	46	29	3·31%
		High (n = 699)	6·88%	25	13	10	4·30%
	2019	Low (n = 1060)	14·56%	65	43	46	8·22%
		Middle (n = 2775)	6·09%	97	38	34	3·71%
		High (n = 679)	6·04%	20	12	9	5·01%

Abbreviations: MH, mental health

a: Interrupted time-series analysis 2016-2019 vs. 2012-2015 * social support (middle vs. low), beta: -0·31, SE: 0·15, p = 0·04

b: Interrupted time-series analysis 2016-2019 vs. 2012-2015 * social support (high vs. low), beta: 0·29, SE: 0·23, p = 0·20

c: Interrupted time-series analysis 2016-2019 vs. 2012-2015 * social support (middle vs. low), beta: 0·31, SE: 0·34, p = 0·36

d: Interrupted time-series analysis 2016-2019 vs. 2012-2015 * social support (high vs. low), beta: 0·41, SE: 0·52, p = 0·42

Supplementary table 9: mental healthcare use 2012-2019, for different levels of urbanicity of the living environment							
			MH care visit last 12 months yes/no[a]	MH care visit last 12 months, if yes how many? (frequency)			Medication for depression and/or anxiety last 12 months yes/no[b]
	**Year (n)**	**Urbanicity**	(% yes)	1-5	5-10	>10	(% yes)
**Pre-campaign**	2012	Low (n = 2076)	5·66%	68	30	19	3·77%
		Mid (n = 1345)	6·38%	39	33	13	4·58%
		High (n = 2206)	7·56%	88	34	44	4·70%
	2013	Low (n = 1908)	5·17%	64	17	17	3·57%
		Mid (n = 1256)	6·87%	57	8	21	5·04%
		High (n = 2035)	8·54%	86	48	39	5·03%
	2015	Low (n = 1639)	5·27%	50	29	7	3·66%
		Mid (n = 1067)	5·64%	32	14	14	4·49%
		High (n = 1686)	7·67%	72	31	26	5·52%
**During campaign**	2016	Low (n = 1898)	6·45%	70	32	20	4·11%
		Mid (n = 1193)	6·97%	31	33	19	5·37%
		High (n = 2138)	8·98%	89	53	48	5·55%
	2017	Low (n = 2013)	6·93%	76	36	27	4·24%
		Mid (n = 1294)	6·97%	51	18	21	5·05%
		High (n = 2470)	9·61%	113	70	53	5·15%
	2018	Low (n = 2300)	6·67%	88	35	29	3·83%
		Mid (n = 975)	7·22%	37	19	14	4·21%
		High (n = 2049)	9·91%	93	49	60	5·98%
	2019	Low (n = 2138)	6·34%	69	29	37	3·80%
		Mid (n = 923)	7·17%	37	19	10	4·02%
		High (n = 1955)	10·46%	94	57	53	6·26%

Abbreviations: MH, mental health

a: Interrupted time-series analysis 2016-2019 vs. 2012-2015 * urbanicity (continuous measure), beta: -0·05, SE: 0·05, p = 0·31

b: Interrupted time-series analysis 2016-2019 vs. 2012-2015 * urbanicity (continuous measure), beta: 0·14, SE: 0·11, p = 0·22

Supplementary table 10: mental healthcare use 2012-2019, for different levels of education								
			**Mentally unhealthy (MHI-5 < 60, scale 0 - 100)[a]**	**MH care visit last 12 months yes/no[b]**	**MH care visit last 12 months, if yes how many? (frequency)**			**Medication for depression and/or anxiety last 12 months yes/no[c]**
	**Year**	**Level of education**	Point-prevalence	(% yes)	1-5	5-10	>10	(% yes)
**Pre-camp**	2012	Primary (n = 477)	22·82%	4·31%	12	1	6	4·70%
		Int· sec· (n = 1425)	17·68%	4·92%	35	17	18	5·99%
		High sec· (n = 640)	20·47%	9·28%	36	14	9	5·36%
		Int· voc· (n = 1316)	17·48%	7·49%	51	24	23	3·75%
		High· voc· (n = 1305)	12·87%	7·08%	45	33	13	3·24%
		University (n = 496)	14·52%	6·30%	17	8	6	2·86%
	2013	Primary (n = 385)	21·56%	5·48%	15	3	3	3·65%
		Int· sec· (n = 1315)	16·43%	4·35%	30	13	14	5·72%
		High sec· (n = 578)	18·69%	9·88%	31	14	11	6·60%
		Int· voc· (n = 1243)	16·41%	8·50%	67	17	21	4·12%
		High· voc· (n = 1228)	12·38%	6·87%	45	24	15	3·43%
		University (n = 475)	10·95%	7·66%	20	7	9	2·75%
	2015	Primary (n = 296)	20·60%	5·44%	11	2	3	3·04%
		Int· sec· (n = 1102)	14·52%	5·18%	32	15	10	6·72%
		High sec· (n = 488)	15·16%	8·45%	26	9	6	5·94%
		Int· voc· (n = 1080)	15·00%	6·78%	38	18	17	4·07%
		High· voc· (n = 1056)	10·98%	7·03%	43	21	10	3·79%
		University (n = 409)	11·49%	4·66%	7	9	3	1·96%
**Start camp**	2016	Primary (n = 344)	23·26%	7·31%	14	7	4	4·36%
		Int· sec· (n = 1142)	17·51%	6·06%	35	19	15	7·36%
		High sec· (n = 591)	18·10%	9·39%	27	17	11	5·25%
		Int· voc· (n = 1310)	15·34%	7·53%	47	26	25	4·50%
		High· voc· (n = 1295)	12·74%	8·07%	46	34	24	4·63%
		University (n = 585)	15·21%	8·47%	23	16	10	2·56%
	2017	Primary (n = 366)	20·77%	7·12%	14	4	8	4·37%
		Int· sec· (n = 1193)	17·52%	5·81%	33	20	16	6·79%
		High sec· (n = 646)	16·25%	12·17%	36	23	19	4·95%
		Int· voc· (n = 1413)	16·14%	8·47%	62	32	25	5·31%

		High· voc· (n = 1476)	13·62%	7·33%	58	28	22	3·79%
		University (n = 714)	13·17%	10·02%	38	21	12	2·66%
	2018	Primary (n = 331)	27·49%	6·67%	13	2	7	5·44%
		Int· sec· (n = 1104)	17·66%	6·66%	36	20	17	6·97%
		High sec· (n = 580)	18·13%	11·11%	34	12	18	5·86%
		Int· voc· (n = 1298)	15,94%	7·22%	48	26	19	4·24%
		High· voc· (n = 1363)	12·33%	8·21%	60	23	28	3·82%
		University (n = 676)	13·76%	10·30%	33	20	16	2·81%
	2019	Primary (n = 307)	26·71%	6·19%	12	4	3	5·86%
		Int· sec· (n = 1027)	18·31%	7·11%	34	22	17	6·72%
		High sec· (n = 536)	18·10%	10·49%	23	16	17	5·97%
		Int· voc· (n = 1210)	15·79%	7·63%	50	18	24	4·38%
		High· voc· (n = 1308)	13·91%	8·19%	54	31	22	3·75%
		University (n = 653)	13·63%	9·90%	32	15	17	3·22%

Abbreviations: MH, mental health; MHI-5, 5-item Mental Health Inventory; camp, campaign; primary, primary school; int· sec·, intermediate secondary education; high sec·, higher secondary education; int· voc·, intermediate vocational education; high voc·, higher vocational education

a: Interrupted time-series analysis 2016-2019 vs. 2012-2015 * education (continuous measure), beta: -0·01, SE: 0·03, p = 0·62

b: Interrupted time-series analysis 2016-2019 vs. 2012-2015 * education (continuous measure), beta: -0·002, SE: 0·04, p = 0·96

c: Interrupted time-series analysis 2016-2019 vs. 2012-2015 * education (continuous measure), beta: 0·09, SE: 0·10, p = 0·35

Supplementary table 11: (mental) healthcare use 2012-2019, different migration backgrounds

			mentally unhealthy (MHI-5 < 60, 0 - 100)[a,b]	MH care visit last 12 months yes/no[c,d]	MH care visit last 12 months, if yes how many? (frequency)			Medication for depression and/or anxiety last 12 months yes/no[e,f]
	**Year (n)**	**Migration background[a]**	Point-prevalence	(% yes)	1-5	5-10	>10	(% yes)
**Pre-camp**	2012	Dutch (n = 4840)	15·60%	6·33%	163	81	61	4·11%
		Non-Dutch HIC (n = 423)	21·99%	7·48%	18	6	9	3·80%
		Non-Dutch LMIC (n = 253)	31·62%	7·35%	8	6	4	5·14%
	2013	Dutch (n = 4467)	14·44%	6·81%	176	62	65	4·37%
		Non-Dutch HIC (n = 385)	17·66%	6·51%	14	7	4	3·64%
		Non-Dutch LMIC (n = 239)	28·03%	8·47%	11	6	3	4·42%
	2015	Dutch (n = 3834)	13·02%	5·91%	123	61	42	4·51%
		Non-Dutch HIC (n = 314)	16·88%	7·67%	14	9	1	3·04%
		Non-Dutch LMIC (n = 187)	25·40%	9·04%	10	3	4	3·70%
**Start camp**	2016	Dutch (n = 4343)	14·28%	7·17%	153	91	66	4·95%
		Non-Dutch HIC (n = 459)	19·61%	9·39%	19	17	7	4·36%
		Non-Dutch LMIC (n = 319)	31·35%	9·91%	14	6	11	5·64%
	2017	Dutch (n = 4638)	14·51%	7·41%	180	95	67	4·57%
		Non-Dutch HIC (n = 530)	18·11%	10·59%	27	15	14	4·37%
		Non-Dutch LMIC (n = 476)	24·79%	12·02%	29	12	16	6·09%
	2018	Dutch (n = 4249)	14·19%	7·46%	164	81	70	4·66%
		Non-Dutch HIC (n = 471)	16·77%	8·76%	19	10	12	5·44%
		Non-Dutch LMIC (n = 422)	29·62%	12·65%	28	8	17	5·45%
	2019	Dutch (n = 4065)	14·78%	7·52%	159	77	69	4·72%
		Non-Dutch HIC (n = 438)	20·32%	9·57%	18	10	11	5·86%
		Non-Dutch LMIC (n = 397)	27·20%	13·38%	19	17	17	5·79%

Abbreviations: MH, mental health; MHI-5, 5-item Mental Health Inventory; camp, campaign HIC, high-income country; LMIC, lower- and middle-income countries
a: Interrupted time-series analysis 2016-2019 vs. 2012-2015 * migration background (non-western vs. Dutch), beta: -0·08, SE: 0·16, p = 0·60
b: Interrupted time-series analysis 2016-2019 vs. 2012-2015 * migration background (western vs. Dutch), beta: -0·15, SE: 0·15, p = 0·30
c: Interrupted time-series analysis 2016-2019 vs. 2012-2015 * migration background (non-western vs. Dutch), beta: 0·67, SE: 0·25, p = 0·008
d: Interrupted time-series analysis 2016-2019 vs. 2012-2015 * migration background (western vs. Dutch), beta: -0·25, SE: 0·22, p = 0·24
e: Interrupted time-series analysis 2016-2019 vs. 2012-2015 * migration background (non-western vs. Dutch), beta: 1·00, SE: 0·68, p = 0·14
f: Interrupted time-series analysis 2016-2019 vs. 2012-2015 * migration background (western vs. Dutch), beta: -0·82, SE: 0·47, p = 0·07

References

1. Scherpenzeel A. Data Collection in a Probability-Based Internet Panel: How the LISS Panel Was Built and How It Can Be Used. *Bulletin of Sociological Methodology/Bulletin de Méthodologie Sociologique*. 2011/01/01 2011;109(1):56-61. doi:10.1177/0759106310387713
2. Das M, Knoef M. Experimental and Longitudinal Data for Scientific and Policy Research: Open Access to Data Collected in the Longitudinal Internet Studies for the Social Sciences (LISS) Panel. In: Crato N, Paruolo P, eds. *Data-Driven Policy Impact Evaluation: How Access to Microdata is Transforming Policy Design*. Springer International Publishing; 2019:131-146.
3. Kieruj N. LISS Data Archive Centerdata. Accessed 10-11-2024, 2024. https://www.dataarchive.lissdata.nl/study-units/view/12
4. Cui J, Lunansky G, Lichtwarck-Aschoff A, Mendoza N, Hasselman F. Quantifying the stability landscapes of psychological networks. *PsyArxiv*. 2023;doi:https://doi.org/10.31234/osf.io/nd8zc
5. van Borkulo CD, Borsboom D, Epskamp S, et al. A new method for constructing networks from binary data. *Scientific Reports*. 2014/08/01 2014;4(1):5918. doi:10.1038/srep05918
6. Brown VA. An Introduction to Linear Mixed-Effects Modeling in R. *Advances in Methods and Practices in Psychological Science*. 2021/01/01 2021;4(1):2515245920960351. doi:10.1177/2515245920960351
7. Bates D, Mächler M, Bolker B, Walker S. Fitting Linear Mixed-Effects Models Using lme4. *Journal of Statistical Software*. 10/07 2015;67(1):1 - 48. doi:10.18637/jss.v067.i01
8. Stronks K, Kulu-Glasgow I, Agyemang C. The utility of 'country of birth' for the classification of ethnic groups in health research: the Dutch experience. *Ethnicity & Health* 2009; **14**(3): 255-69.
9. CBS. MHI-5. 2025. https://www.cbs.nl/en-gb/news/2022/50/more-women-than-men-with-psychological-complaints/mental-health (accessed 20-1-2025 2025).

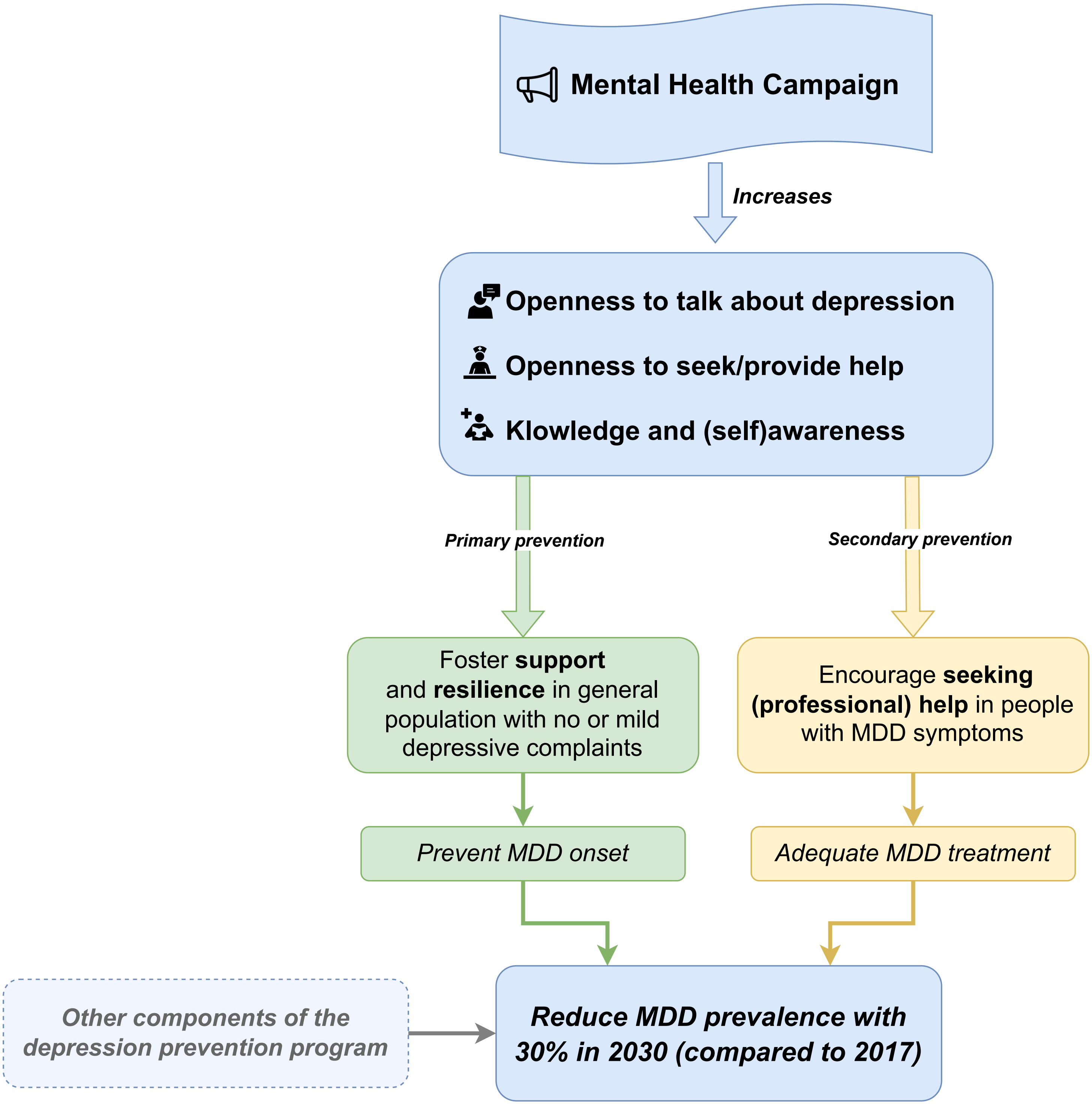
Mental Health Campaign
Increases
Openness to talk about depression
Openness to seek/provide help
Klowledge and (self)awareness
Primary prevention
Secondary prevention
Foster support and resilience in general population with no or mild depressive complaints
Encourage seeking (professional) help in people with MDD symptoms
Prevent MDD onset
Adequate MDD treatment
Other components of the depression prevention program
Reduce MDD prevalence with 30% in 2030 (compared to 2017)

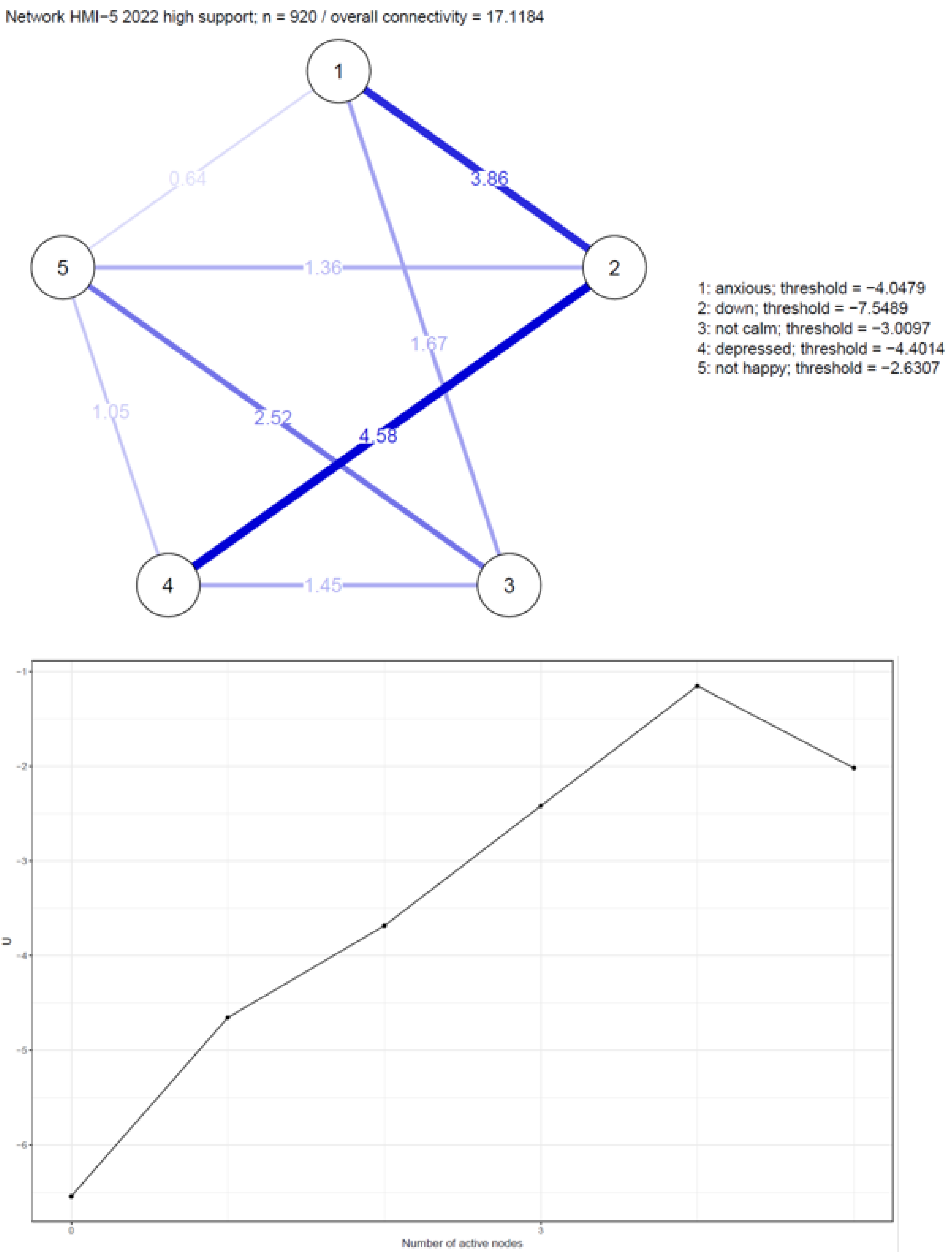

Supplementary figure 1. HMI-5 network and stability landscape for high support group in 2022, unhealthy attractor basin can be seen at the right side of the graph

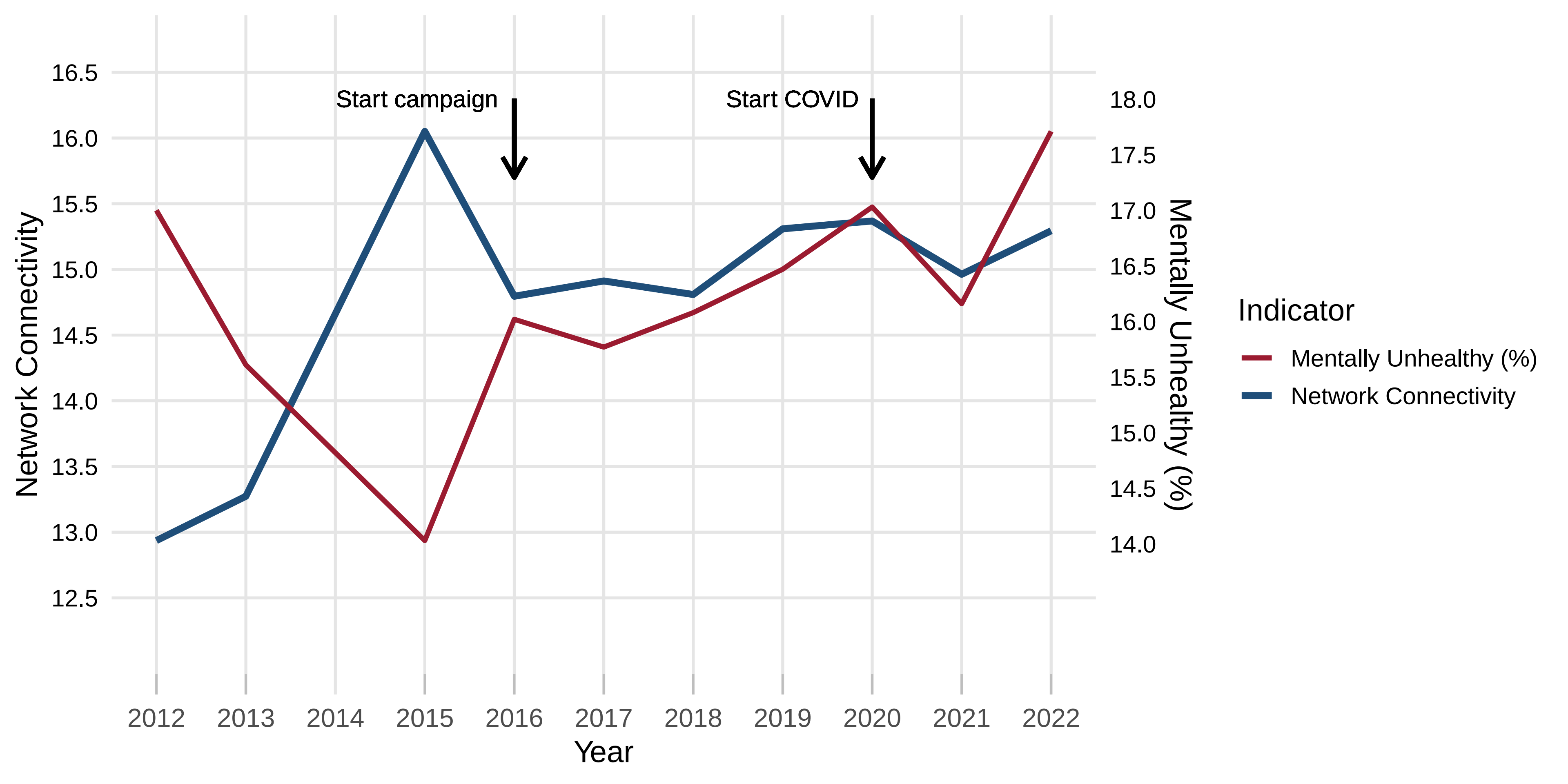

Start campaign
Start COVID
Network Connectivity
Mentally Unhealthy (%)
Year
Indicator
Mentally Unhealthy (%)
Network Connectivity
16.5
16.0
15.5
15.0
14.5
14.0
13.5
13.0
12.5
18.0
17.5
17.0
16.5
16.0
15.5
15.0
14.5
14.0
2012
2013
2014
2015
2016
2017
2018
2019
2020
2021
2022

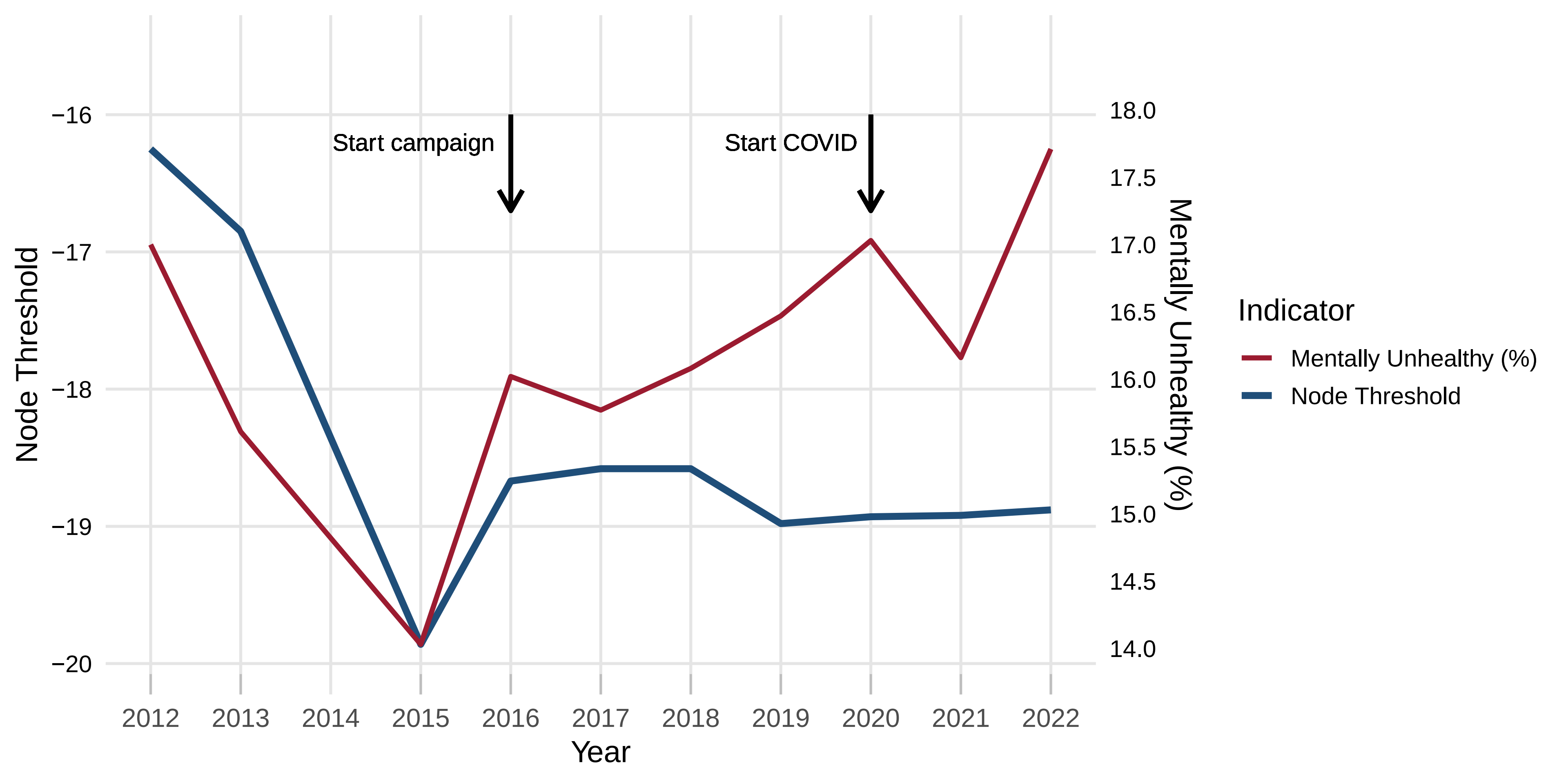

Node Threshold
−16
−17
−18
−19
−20
Start campaign
Start COVID
18.0
17.5
17.0
16.5
16.0
15.5
15.0
14.5
14.0
Mentally Unhealthy (%)
2012
2013
2014
2015
2016
2017
2018
2019
2020
2021
2022
Year
Indicator
Mentally Unhealthy (%)
Node Threshold

Caption for supplementary material

Supplementary figure 1: Proposed mechanisms of mental health campaign in order to contribute to reduction of MDD prevalence

Supplementary figure 2: MHI-5 network and derived stability landscape for the entire group in 2012. Numbers in the connections represent the edge weight of that specific connection. In the graph, number of active nodes is shown in the x-axis and generalized potential function of the state is shown on the y-axis. A lower value on the yaxis represents a higher stability of the network in the state as indicated on the x-axis (i.e., the number of active nodes). In this case, the stability landscape has two local minima (i.e., attractor basins), with most stable attractors being in a state with 0 active nodes (i.e., the most healthy state). We observe a small local attractor basin at the other end as well, as the stability of the network with 5 active nodes (i.e., the most pathological state) is slightly higher than when 4 nodes are active, indicating that it requires more 'energy' to go from 5 to 4 nodes active, than e.g. from 4 to 3 nodes active.

Supplementary figure 3: overview of the connectivity of the population-level network and percentage of participants that was mentally unhealthy for each year (NB no data was collected in 2014).

Supplementary figure 4: overview of the total node threshold of the population-level network and percentage of participants that was mentally unhealthy for each year (NB no data was collected in 2014).
